# Fragment-Based Discovery of Potent RNA-Competitive Inhibitors of the DEAH-Box RNA Helicase DHX8

**DOI:** 10.1021/acs.jmedchem.6c00732

**Published:** 2026-08-04

**Authors:** Benjamin J. Read, Caroline Ewens, Federica Gigante, Jemima Thomas, Catarina Felisberto-Rodrigues, Silvia Alvarez Peres, Catherine Tighe, Edgar de las Heras Ruiz, Kai Schiemann, Andrew G. Malcolm, Peter Craig McAndrew, Mark Stubbs, Harshnira Patani, Helena dos Santos Costa, Katharine Stoodley, Lisa Pickard, Michael Busch, Emma Gunnell, Sara Silva, Alexandra Knopp, Stephen T. Hallett, Martin Augustin, Alfred Lammens, Michael Carter, Mirco Meniconi, Marco Ballarotto, Jon Ainsley, Paul Meister, Debarati Sethi, Rosemary Burke, Andrea Scarpino, Yann-Vaï Le Bihan, Ulrich Grädler, Julian Blagg, Paul Workman, Paul A. Clarke, Andreas Blum, Christina Esdar, Gurdip Bhalay, Rob L. M. van Montfort

**Affiliations:** † Centre for Cancer Drug Discovery, Division of Cancer Therapeutics, 5053 The Institute of Cancer Research, London SM2 5GP, U.K.; ‡ Division of Structural Biology, 5053 The Institute of Cancer Research, London SW3 6JB, U.K.; § 2792 The Healthcare Business of Merck KGaA, Frankfurter Strasse 250, 64293 Darmstadt, Hesse, Germany; ∥ 384733 Proteros Biostructures GmbH, Bunsenstraße 7a, D-82152 Planegg-Martinsried, Germany

## Abstract

Human DHX8 is a spliceosomal DEAH-box RNA helicase involved in releasing mRNA from the spliceosome and crucial in ensuring splicing fidelity. DHX8 was identified as a promising therapeutic oncology target due to its role in regulating stress-adaptive gene expression, including HSF1-dependent transcription, while having broader transcriptional effects in cells under oncogenic stress. We report the discovery of novel RNA-competitive DHX8 inhibitors based on a 2-(phenethylthio)­nicotinic acid scaffold, which were optimized using a structure-guided design approach, following a biophysical fragment screen. This yielded compound **53** with nanomolar biochemical potency, good *in vitro* PK, and activity in a cellular target engagement assay. Optimizing inhibitor binding between Arg647 and the nonconserved His693, coupled with extending into a pocket in the DHX8 Winged-Helix domain, was crucial for potency improvement. By binding in the Winged-Helix domain, these inhibitors restrict the helicase domain’s conformational plasticity, stabilizing a closed, inactive conformation while sterically blocking ssRNA translocation.

## Graphical abstract

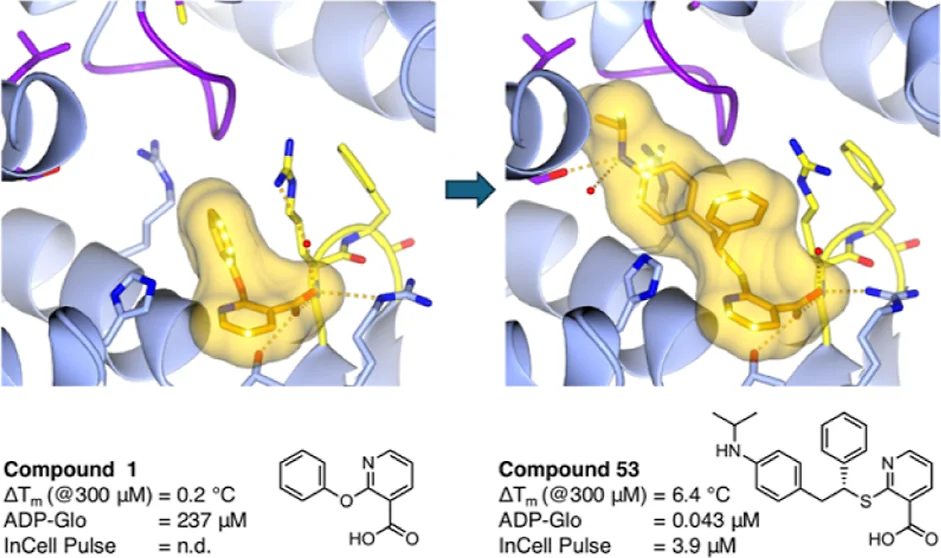

## Introduction

DHX8 is a spliceosomal RNA helicase belonging to the DEAH-box helicase family that couples the hydrolysis of nucleoside triphosphates (NTPs) with RNA translocation to facilitate the release of mature mRNA from the spliceosome.^1–3^ In addition, DHX8 is thought to be essential for splicing fidelity and proof-reading by promoting optimal and rejecting suboptimal 3′ splice sites, as reported for its functional homologue in yeast Prp22.^4,5^ Like other members of the DEAH-box RNA helicase family, DHX8 consists of a nonconserved N-terminal region and a conserved C-terminal helicase domain. Its helicase domain comprises the two canonical RecA domains, RecA1 and RecA2, which contain 12 characteristic sequence motifs important for NTP binding and hydrolysis, RNA binding, and helicase activity. Additionally, the helicase domain contains the C-terminal Winged-Helix, ratchet-like, and oligonucleotide binding (OB)-fold domains, which together with the two RecA domains form the RNA-binding tunnel.^3^ DEAH-box helicases such as DHX8 are characterized by their ability to unwind duplex oligonucleotide substrates with a 3′ single-stranded overhang consistent with a generally preferred 3′ to 5′ unwinding polarity.^6^

We recently identified and validated DHX8 in a functional genomic screen for regulators of Heat Shock Factor (HSF1) – a ligand-less and difficult-to-drug master regulator transcription factor that plays a central role in the cellular response to stress.^7^ HSF1 is also required for oncogene-driven transformation and progression of malignant cells.^8^ We found that DHX8 silencing or degradation resulted in intron retention within HSF1 pre-mRNA, leading to reduced HSF1 protein levels.^7^ This was accompanied by altered processing of HSF1-regulated transcripts associated with poor clinical outcomes,^9^ as well as effects on oncogene-associated gene expression. Mechanistically, DHX8 ATPase activity and RNA binding were critical for proper splicing and expression of HSF1 and other cancer-associated transcripts. We also demonstrated that siRNA-mediated knockdown of DXH8 had a substantially greater impact on the survival of cancer cells compared to untransformed, nonimmortalized cell lines, indicating a potential therapeutic window. Collectively, these findings prompted our initiation of a program to identify small-molecule DHX8 inhibitors as chemical probes and potential drug candidates for the treatment of cancers characterized by a highly activated stress response, or by oncogenic dependencies on alternative splicing, including those associated with tumor progression and therapeutic resistance.

However, helicases are considered challenging drug targets due to the conserved nature of their active sites and the transient nature of their conformational states during their dynamic enzymatic cycles.^10,11^ Fragment-based methods have proved valuable in finding ligands that bind to challenging-to-drug proteins.^12,13^ Therefore, to identify suitable starting points for the design of DHX8 inhibitors, we conducted a parallel surface plasmon resonance (SPR) and thermal shift assay (TSA) fragment screening campaign combined with hit follow-up using SPR concentration-response and ligand-observed nuclear magnetic resonance (LO-NMR) experiments. This yielded four RNA-competitive fragment hits, one of which we optimized, using a structure-guided fragment growing approach, into a series of potent DHX8 inhibitors that showed target engagement in an InCell Pulse assay in human cancer cells. To our knowledge, our study reports the first potent RNA-competitive DEAH-box RNA helicase inhibitors that show cellular target engagement and the first nanomolar inhibitors of DHX8, an RNA helicase integral to the core human spliceosome and mRNA splicing cycle. Therefore, these findings will provide crucial insights into the exploration of DEAH-Box helicases as an underexploited family of potential therapeutic targets and in targeting mRNA splicing with small-molecule inhibitors that act on a different component of the spliceosome than previously reported SF3B1 inhibitors.^14^

## Results and Discussion

### Chemistry

Synthesis of compounds for hit confirmation, hit expansion, and hit optimization are summarized above (Schemes 1, 2, and 3) with further information in the Supporting Information. Route A (Scheme 1) used the S_N_Ar displacement of a range of aromatic halides by a selection of aromatic thiols under basic conditions. Route B (Scheme 1) used 2-mercaptonicotinic acid to generate the corresponding thiolate and react with selected aryl and alkyl bromides under basic conditions. Route C (Scheme 1) was used to synthesize the majority of the extended linker analogues (Tables 4 to 7) using a base-mediated reaction of 2-mercaptonicotinic acid with sulfate esters.

**Scheme 1:**
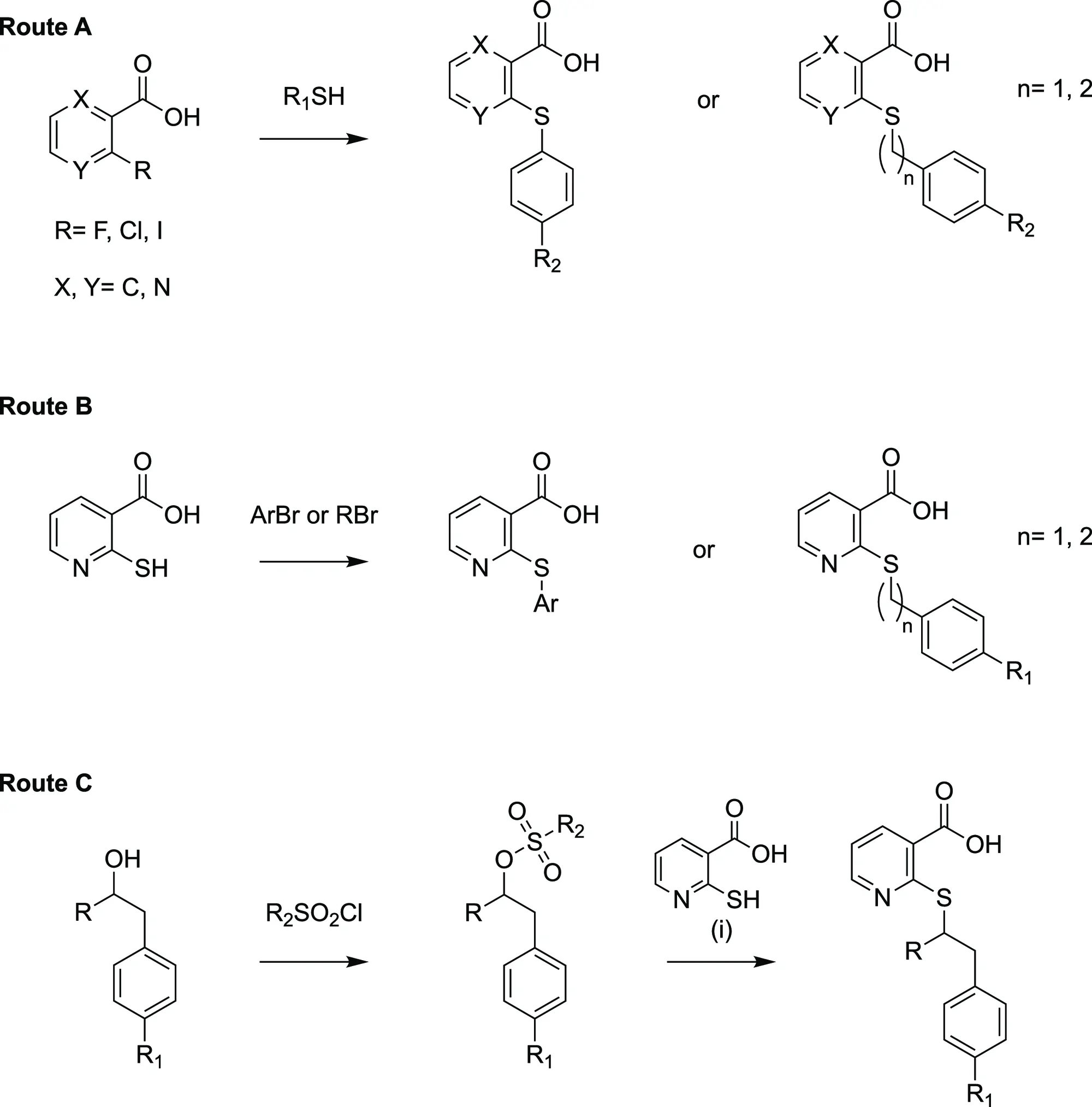
General Synthetic Routes for Thioether Analogues: Route A (Compounds **13**–**17** and **25**); Route B (Compounds **22**, **23**, **36**, **37**, and **39**); and Route C (Compounds **29**–**35**, **38**, **46**–**47**, **50**–**53**)

**Scheme 2:**
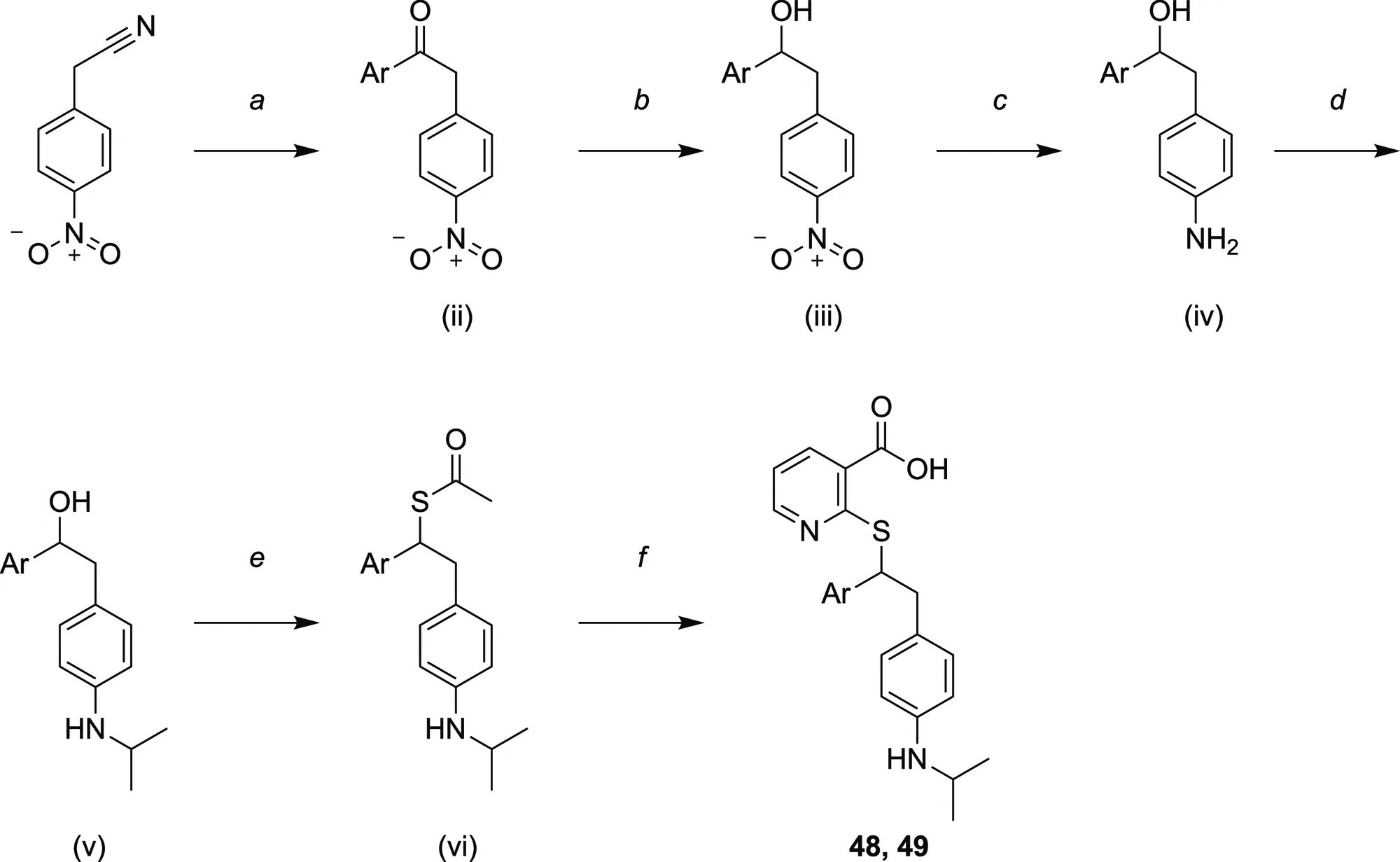
Synthesis of Aromatic Analogues **48**, **49**. Reagents: *a*: ArB­(OH)_2_, Pd­(OAc)_2_, TFA, H_2_O/THF, 80 °C, 13 h; *b*: NaBH_4_, MeOH, rt, 13 h; *c*: Fe, NH_4_Cl, EtOH/H_2_O, 80 °C, 2 h; *d*: Acetone, NaBH­(OAc)_3_, AcOH, DCM, rt, 13 h; *e*: Thioacetic Acid, PPh_3_, DIAD, THF (0.30 mL, 1.513 mmol), rt, 13 h; and *f*: 2-Iodonicotinic Acid, Pd_2_(dba)_3_, XantPhos, Cs_2_CO_3_, 1,4-Dioxane, 120 °C, 16 h

**Scheme 3:**
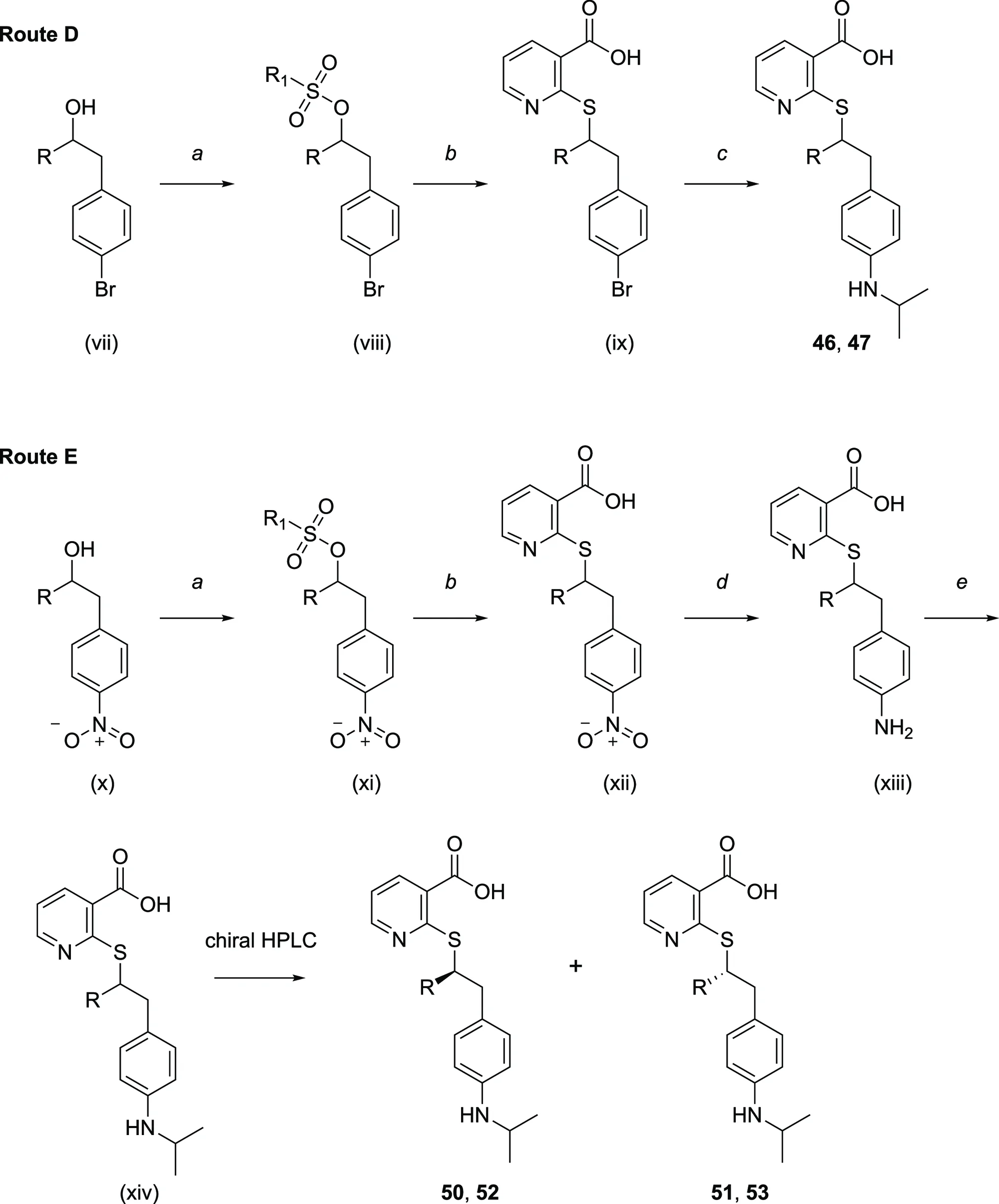
*a*: R_1_SO_2_Cl, Pyridine, DCM, rt, 13 h; *b*: 2-Mercaptonicotinic Acid, K_2_CO_3_, EtOH, 80 °C, 13 h; *c*: Isopropylamine, JohnPhos, Pd_2_(dba)_3_, *t*-BuONa, 1,4-Dioxane, 80 °C, 13 h; *d*: Fe, NH_4_Cl, EtOH/H_2_O, 80 °C, 2 h; and *e*: Acetone, NaBH­(OAc)_3_, AcOH, DCM, rt, 1 h

Schemes 2 and 3 show the synthetic routes used to access compounds shown in Table 7. Compounds **48** and **49** (Scheme 2) were synthesized using a Pd-catalyzed reaction of an aryl boronic acid and 4-nitrophenylacetonitrile to give the ketone (ii). Reduction of ketone (ii) gave the expected alcohol (iii) as a racemic mixture. An iron-mediated reduction of the aryl nitro group gave amine (iv), which was followed by a reductive amination reaction with acetone to install the isopropylaniline moiety in (v). Nucleophilic displacement of alcohol (v) with thioacetic acid under Mitsunobu reaction conditions gave thioester (vi) and subsequent palladium-catalyzed reaction with 2-iodonicotinic acid gave desired compounds (**48** and **49**).

Synthesis of **46**–**47** and **50**–**53** (Table 7) were made as depicted in route D or route E (Scheme 3). The R-group in these final compounds was introduced using a variety of methods (details are provided in the Supporting Information). Both routes (Scheme 3) involve the conversion of the secondary alcohols (vii, x) to the corresponding sulfate esters (viii, xi) and their subsequent base-mediated nucleophilic displacement with 2-mercaptonicotinic acid to give intermediates (ix) and (xii). Installation of the isopropylaniline moiety was achieved through a Buchwald coupling (Route D, step c) or via reductive alkylation with acetone (Route E, step e). Racemic mixtures were purified by chiral HPLC to give the desired single enantiomers **50**, **51** and **52**, **53**.

### DHX8 Fragment Screening

To identify ligands that bind to DHX8, we conducted parallel biophysical fragment screens of the ICR library of 2000 fragments^15^ using both TSA and SPR (Figure 1). The TSA screen was performed against an N-terminally truncated DHX8 variant containing only the helicase domain (DHX8Δ547), as its recombinant production yield was much higher than that for full-length DHX8.^3^ The screen was carried out at a final fragment concentration of 300 μM (Figure 1). ADP, which stabilizes DHX8Δ547 with a shift in melting temperature (Δ*T*_m_) of ∼ 4.5 °C at 300 μM, was used as a positive control (Figure S1). Because the Δ*T*_m_ values observed in the fragment screen were generally low, hits were defined based on a positive Δ*T*_m_ of at least 1 standard deviation above the mean (Δ*T*_m_ ≥ 1 sd), independently calculated for each assay plate. This yielded 18 initial fragment hits, corresponding to a 0.9% hit rate.

**Figure 1:**
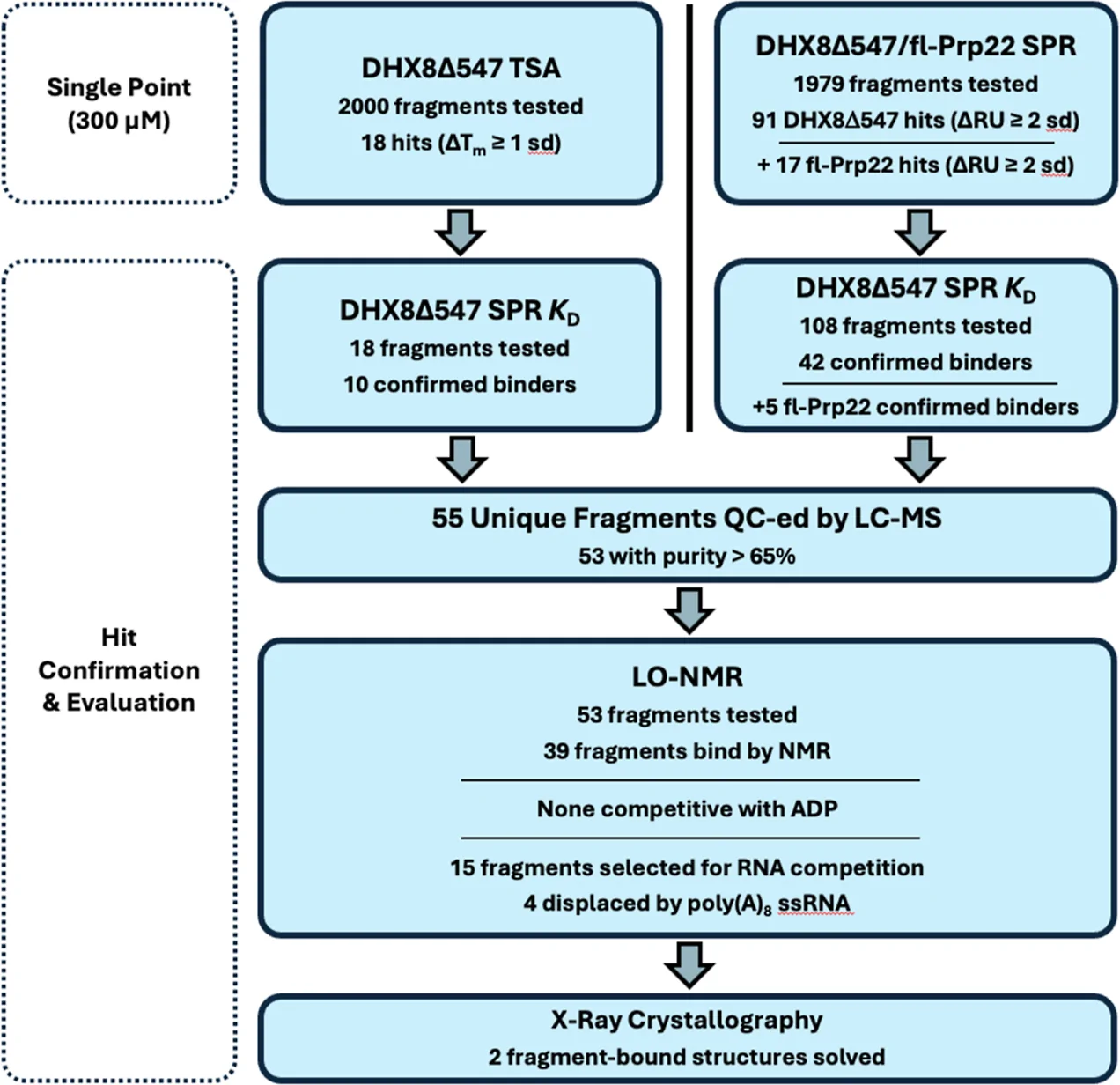
Summary of the DHX8 fragment screening campaign and subsequent hit confirmation. A parallel TSA and SPR fragment screening campaign of the ICR fragment library was carried out against DHX8 to identify suitable starting points for the design of potent DHX8 inhibitors. Fragment hits from both screens were characterized in 8-point SPR concentration-response experiments. Hits confirmed in this step were checked for purity and correct molecular weight using LC-MS and the remaining hits were characterized in LO-NMR experiments using three different methods: CPMG, WaterLOGSY, and STD NMR. Fragments that showed binding in LO-NMR were further analyzed in LO-NMR competition experiments with ADP. No fragment hits were competitive with ADP. Based on chemical diversity and SPR affinity, 15 fragments were selected for LO-NMR competition experiments with a poly­(A)_8_ ssRNA. Fragments competitive with RNA were selected for binding mode elucidation by X-ray crystallography.

To identify fragments binding to the N-terminal as well as the helicase domain of DHX8, we attempted to set up SPR fragment screening against both full-length DHX8 (fl-DHX8) and the truncated DHX8Δ547. Although we were able to immobilize fl-DHX8 on the SPR chip, the initial data were less robust than for DHX8Δ547. As immobilization of the full-length yeast DHX8 homologue Prp22 (fl-Prp22) provided better initial data, the SPR screen of the ICR fragment library was carried out against DHX8Δ547 and fl-Prp22. Twenty-one fragments that bound to reference cells in previous in-house SPR screens^16^ were excluded. The remaining 1979 fragments were screened at a single concentration of 300 μM. ADP and poly­(A)_10_ ssRNA were used as positive controls (Figure S2). Fragments with a binding level of at least two standard deviations from the mean value (ΔRU ≥ 2 sd) were considered primary hits. The SPR screen yielded 91 unique hits that bound DHX8Δ547, of which 63 also bound fl-Prp22. In addition, the screen identified 17 fragments that bound strongly to fl-Prp22 (ΔRU ≥ 2 sd) and also showed weak binding to DHX8Δ547 (1 sd ≤ ΔRU ≤ 2 sd). Combining the strong DHX8Δ547 and fl-Prp22 hits gave a total of 108 primary hits which equates to a hit rate of 5.5% (Figure 1).

### Fragment Hit Confirmation

All TSA and SPR fragment hits were characterized in 8-point concentration-response SPR experiments against DHX8Δ547 and fl-Prp22 (Figure 1). Of the 18 TSA hits, 10 were confirmed in SPR and showed concentration-dependent binding to both DHX8Δ547 and fl-Prp22. Of the 108 SPR hits, 42 showed concentration-dependent binding to both DHX8Δ547 and fl-Prp22, while an additional 5 fragments displayed binding to fl-Prp22 only. Interestingly, only 2 of the identified hits were found in both the TSA and SPR screens, giving a total number of 55 unique hits and highlighting the value of a multifaceted biophysical fragment screening approach to identify hits against challenging targets.^17,18^ Of the 55 confirmed hits, 10 yielded SPR *K*_D_ values, which ranged between 270 and 930 μM, all others were above the limit of quantification of 1000 μM. LC-MS quality control of the stock solutions confirmed that 53 fragments had the correct molecular weight and a purity greater than 65%.

To build further confidence in the hits, we used repurchased fresh stocks to characterize their binding to DHX8Δ547 in three LO-NMR methods: a Carr-Purcell-Meiboom-Gill (CPMG) relaxation edited ^1^H NMR experiment,^19^ a Water Ligand Observed via Gradient SpectroscopY (WaterLOGSY) method,^20^ and a Saturation Transfer Difference (STD) approach.^21^ Of the 53 hits, 39 showed clear binding in all three methods. To investigate if the fragments bound in the DHX8 ATP-binding site, we carried out competition experiments by incubating the 39 fragment/DHX8Δ547 solutions with ADP. However, the peak intensities of none of the 39 fragments could be recovered in the presence of ADP in any of the three LO-NMR methods, therefore, they were unlikely to bind in the DHX8 ATP-binding site. Based on their affinity by SPR, sensorgram quality, size of peak reduction in the initial CPMG experiment, and diversity in chemical structure, we selected 15 fragments for similar competition experiments with poly­(A)_8_ ssRNA. For compounds **1**–**3** (Figure 2), displacement by RNA was observed with all three LO-NMR methods, although for fragment **3** only partial competition was observed (Figure S3). For a fourth fragment, compound **4**/ibuprofen (Figure 2) partial competition was observed with only the CPMG method (Figure S3).

**Figure 2:**
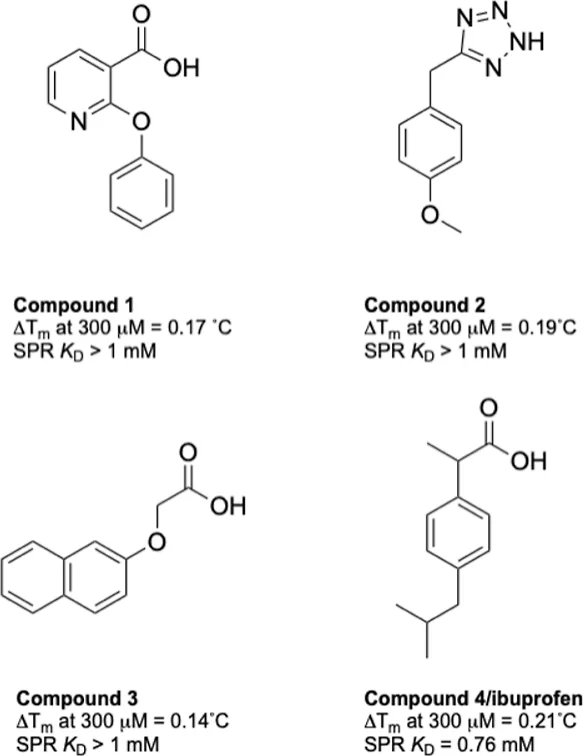
Chemical structures of DHX8 fragment hits. Fragments **1**–**4** were identified as hits in parallel TSA and SPR fragment screening campaigns against the helicase domain of DHX8 (DHXΔ8547) and were confirmed by SPR and LO-NMR experiments. Thermal shifts (Δ*T*_m_) and SPR *K*_D_ values are indicated.

### Determining the Fragment Binding Mode

To obtain insight into the binding mode of the four RNA-competitive fragments, we carried out soaking experiments with compounds **1**–**4** in DHX8Δ547 crystals grown in conditions we previously used to determine the ADP-bound structure of DHX8Δ547.^3^ Although no electron density was observed for compounds **2** and **3**, we successfully determined the crystal structures of DHX8Δ547 bound to ADP and compounds **1** and **4** at a resolution of 2.65 Å and 2.55 Å, respectively (Figure 3A,B). Consistent with the LO-NMR experiments, fragments **1** and **4** did not replace the bound ADP but rather in fact both bind inside the RNA-binding tunnel near the conserved hook-turn motif in the DHX8 RecA1 domain — a motif important for RNA translocation. Superposition of the two fragment-bound structures with the RNA-bound structure of DHX8Δ547 ^3^ shows that both fragments are anchored with their carboxylic acid at the base of the hook-turn motif, mimicking binding of the RNA backbone phosphate from the A6 nucleotide by forming hydrogen bonds with the backbone amide of Arg647 and the side chains of Thr662 and Arg620 (Figure 3C–F). In addition, both fragments contain a phenyl ring which binds in the RNA-binding tunnel, overlapping with the binding location of the A6 ribose moiety, and sandwiched between the side chains of His693 and Arg647 (Figure 3E,F). The isobutyl aliphatic tail of **4** points further into the RNA-binding tunnel toward a small hydrophobic pocket in the Winged-Helix domain, formed by a loop comprising residues Ala981 to Pro987 and the side chains of Leu930, Ser931, and Cys990; a bound DMSO molecule is also observed in this pocket (Figure 3F).

**Figure 3:**
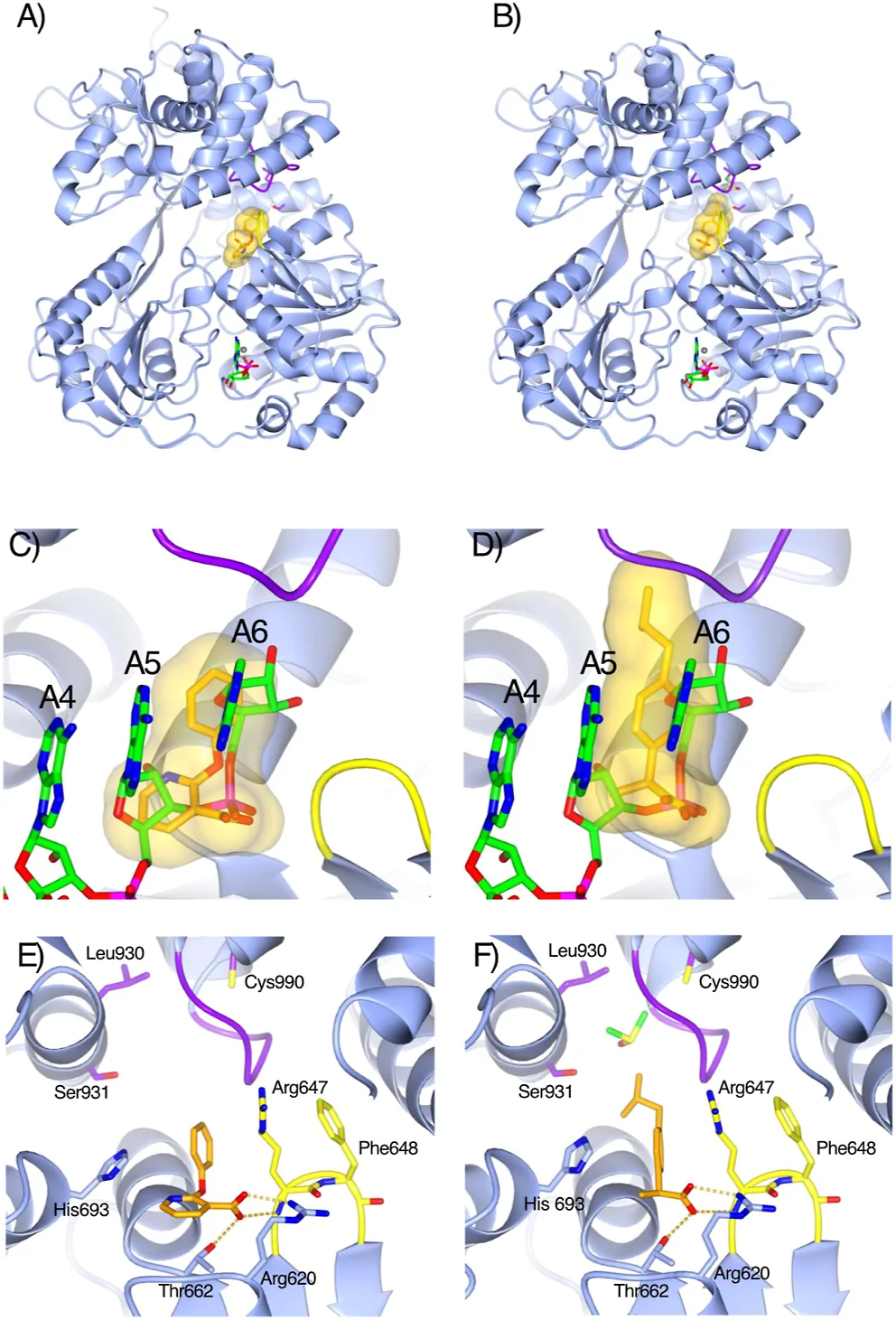
Binding mode of fragments **1** (PDB ID 28ZI) and **4** (PDB ID 28ZJ). Fragment **1** (A,C,E) and **4** (B,D,F) both bind in the RNA-binding tunnel of the DHX8 (DHX8Δ547) helicase domain (blue), close to the conserved hook-turn motif (yellow). The fragments are shown in cylinder representation with carbon atoms in orange superimposed with a semitransparent orange molecular surface. The bound ADP molecules are shown in cylinder representation with carbon atoms in green and residues defining the Winged-Helix pocket are shown in purple. Magnesium ions interacting with the ADP are displayed as gray spheres. (C,D) Superposition of the fragment **1**- and **4**-bound DHX8Δ547 structures and RNA DHX8Δ547 (PDB code 6HYU) showing that their carboxylic acid moieties mimic the binding of the RNA backbone phosphate of the A6 nucleotide and that a phenyl group in each fragment overlaps with the ribose moiety of the A6 RNA nucleotide. The RNA is colored by atom type with carbon atoms in green. (E,F) Close up of the interactions of fragment **1** and **4** showing the polar interactions with DHX8 residues Arg620, Arg647, and Thr662. Hydrogen bonds are shown as dark yellow lines. A bound DMSO molecule is shown in atom colors with carbons in green.

Characterization of fragments **1** and **4** in an ADP-Glo ATP hydrolysis assay and RNA FA (fluorescence anisotropy) binding assay, adapted from our previously reported assays,^3^ yielded respective IC_50_ values of 237 μM and 180 μM for **1** and 144 μM and 57 μM for **4**. This further confirmed them as DHX8 RNA-tunnel binders and, with respective ADP-Glo assay-based ligand efficiencies (LEs) of 0.32 and 0.34, ligand-efficient inhibitors of DHX8 ATPase activity. We pursued the optimization of both fragment hits, but in this study, we will focus on the elaboration of **1** into potent DHX8 inhibitors, while the optimization of **4** will be described elsewhere.

### Optimizing the Fragment Binding Mode

Our next aim was to confirm consistency of the binding mode of fragment **1** using an analogue-by-catalogue approach, which was complemented by mining the ICR and Merck KGaA compound collections and in-house synthesis of bespoke matched pairs (Table 1). This approach yielded fragment **5**, which showed 6- and 9-fold improvements in IC_50_ values as measured in the respective RNA FA and ADP-Glo assays (Table 1). A 2.64 Å crystal structure of **5** bound to DHX8 confirmed a similar binding mode to **1** with the chlorine substitution in the para-position of the B-ring of **5** pointing further into the RNA-binding tunnel in the same general direction as the isobutyl tail in **4** (Figure 4A). This substitution improved the potency and ligand efficiency of **5** and highlighted that growing fragments into this space could access binding hotspots within the RNA-binding tunnel.

**Table 1:**
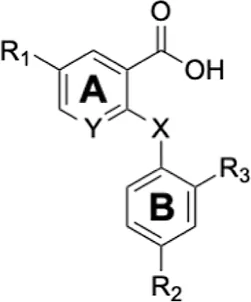

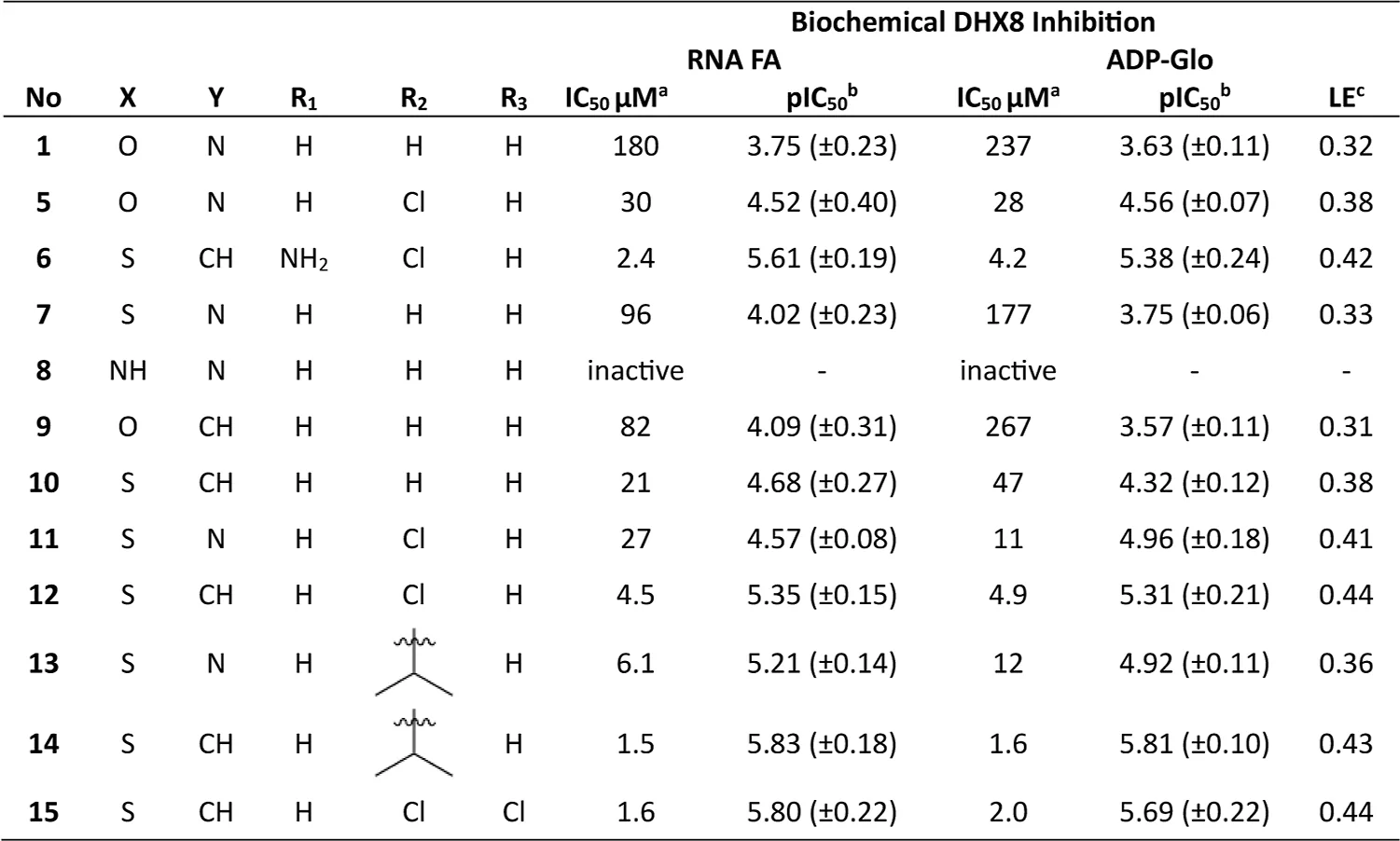
Optimizing the Fragment Binding Mode^a^

a DHX8 fragment binding was confirmed using a fluorescence anisotropy assay based on a fluorescently labeled poly­(A) ssRNA substrate. The effect of fragment binding on DHX8 ATP hydrolysis was characterized using an ADP-Glo assay. Data represent the geometric mean IC_50_ (^a^) or arithmetic mean pIC_50_ ± standard deviation (^b^) of at least three replicates. Ligand Efficiency (^c^) was calculated based on the ADP-Glo pIC_50_ data, using the formula LE = (1.4pIC_50_)/(number of non-hydrogen atoms).

**Figure 4:**
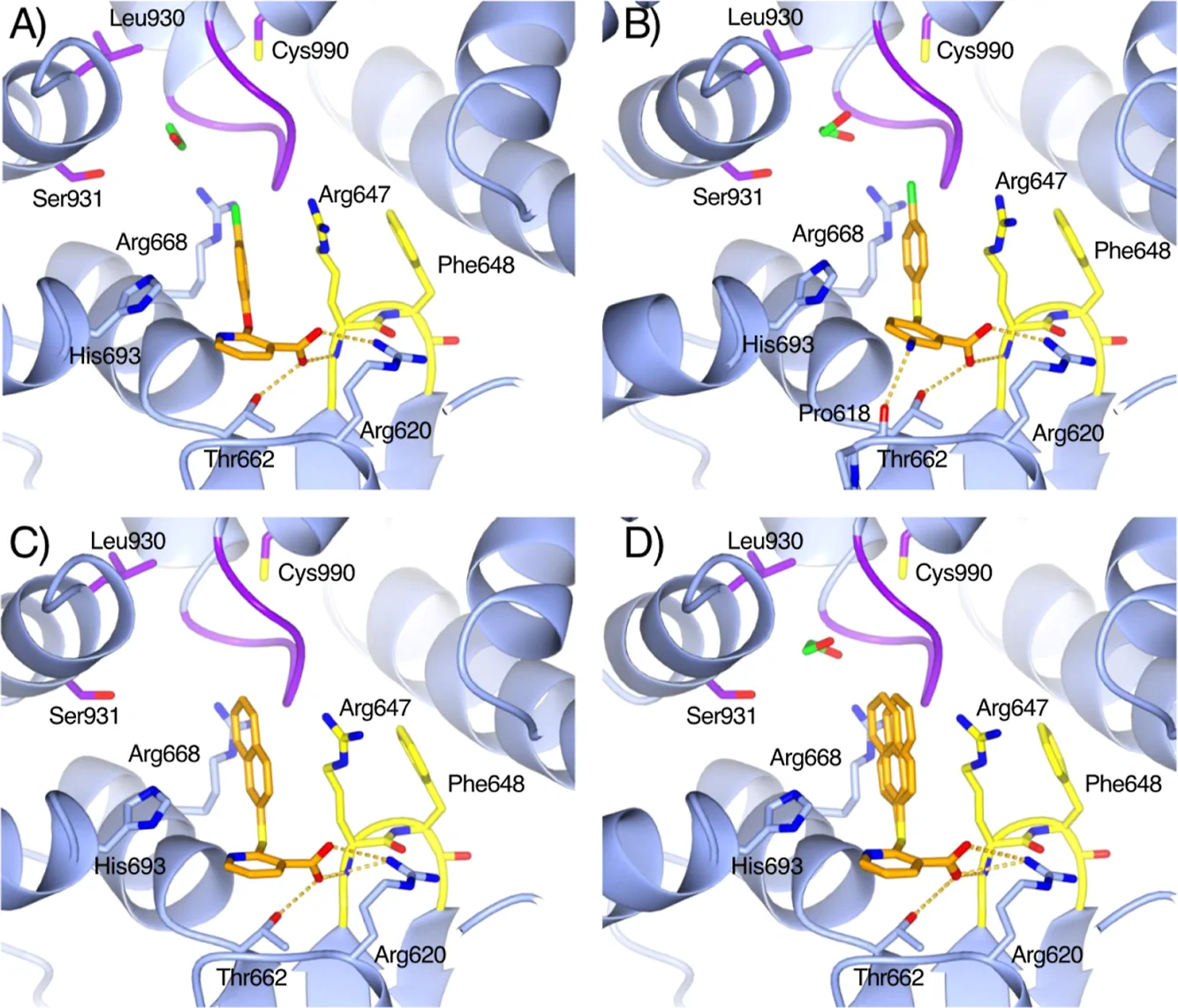
Confirmation of the binding mode for compounds **5**, **6**, and **21**. (A) X-ray structure of DHX8 bound to compound **5** (PDB ID 28ZK). (B) X-ray structure of DHX8 bound to compound **6** (PDB ID 28ZL). (C) X-ray structure of DHX8 bound to the compound **21** (PDB ID 28ZM) with the naphthyl group adopting a single conformation (Chain C). (D) X-ray structure of DHX8 bound to the compound **21** with the naphthyl group adopting two conformations (Chain A). Compounds are shown in cylinder representation with carbon atoms in orange. The hook-turn motif is shown in yellow and the loop and residues defining the pocket in the Winged-Helix domain are shown in purple. Hydrogen bonds are shown as yellow dotted lines. The ethylene glycol molecule occupying the Winged-Helix pocket is shown in atom colors with carbon atoms in green. Key residues are labeled.

The potential of this fragment series was further confirmed by **6**, a key fragment analogue from the ICR HTS library, which showed 75- and 57-fold improvements in the respective RNA FA and ADP-Glo assays as compared to **1**, resulting in an increase in LE to 0.42 (Table 1). A 2.69 Å crystal structure of **6** bound to DHX8 showed that in addition to the interactions observed for **1** and **5**, the exocyclic primary amine on the A-ring of **6** formed an interaction with the backbone carbonyl atom of Pro618 (Figure 4B); however, the long distances (between 3.1 and 3.3 Å for the 4 independent molecules in the asymmetric unit) and suboptimal directionality resulted in a modest potency gain (1.8- and 1.2-fold in the RNA FA and ADP-Glo assays, respectively) between **6** and its matched pair **12** (Table 1). In light of this, and the potential detrimental effect of the addition of an extra primary amine to the cell permeability and other drug-like properties of the compounds, this exocyclic amine was not retained in further analogues. Instead of the ether linker in **1** and **5**, the A- and B-ring of **6** are connected via a sulfur atom, highlighting that this variation in the type of linker might be beneficial to potency. Indeed, comparing **1** and **5** with their respective matched pairs **7** and **11** shows a slight improvement in potency for the sulfur-linked compounds. By contrast, **8**, which has an NH-linker, was inactive in both the RNA FA and ADP-Glo assays (Table 1). In addition, the A-ring of **6** is a phenyl instead of a pyridine. Exploring the effect of a pyridine versus the phenyl A-ring with direct matched pairs **1** and **9** and **7** and **10** showed comparable potencies between **1** and **9** and an approximate 4-fold improvement of the phenyl-containing **10** compared to the pyridine-containing **7**, demonstrating that the pyridine nitrogen is not essential (Table 1). However, the pyridine provides an option to lower lipophilicity which may benefit both solubility and *in vitro* clearance in later optimization. Exploring the B-ring para-position, as exemplified by **11**–**14**, confirmed that a para-substituent led to a general improvement in potency and ligand efficiency compared to **7** or **10**, respectively. Substitution of the B-ring ortho-position was also tolerated as demonstrated by **15**.

Interestingly, in the fragment-bound DHX8 structures of **1**, **5**, and **6**, the carboxylic acid anchor adopts a twisted conformation with a dihedral angle of 58–74° with respect to the A-ring, which we hypothesized to be important for binding. To investigate this hypothesis, a quantum mechanical analysis was conducted on **1** and its matched pairs **7** and **8**, which differ only in the linker heteroatom (X = S and NH, respectively). This evaluation showed that the lowest-energy conformer of **8** assumes an almost completely planar conformation, stabilized by an intramolecular hydrogen bond between the NH linker and the carboxylic acid. By contrast, **1** and **7** are not conformationally locked by the intramolecular hydrogen bond, with both the B-ring and the carboxylic acid group able to engage in productive protein-ligand interactions. Moreover, in the lowest-energy conformers of both compounds, the carboxylic acid assumes a twisted conformation consistent with the conformation observed in the crystal structures (81° and 28.5°, respectively, Figure S4A). Both the coplanarity of the diaryl system and the lack of a twisted carboxylate in the lowest energy conformation of **8** result in a larger computed conformational penalty of binding (∼2 kcal/mol) compared to **1** and **7**, providing a rationale for the lack of activity of the NH-linked fragments (Figure S4B). To potentially favor an out-of-plane conformation of the carboxylic acid in the S-linked fragments, we introduced a nitrogen (compound **16**) and explored a fluorine substituent at the 6-position of the A-ring (compounds **18** and **19**). The latter was supported by quantum mechanical-level dihedral scan calculations confirming that the fluorine stabilizes a twisted conformation close to the experimentally observed dihedral angles (Figure S5). Unfortunately, these modifications did not improve potency, as exemplified by matched pair comparisons **16** with **14**, **17** with **13**, respectively, and matched pairs **18** versus **10**, and **19** versus **12** (Tables 1 and 2). Moreover, the larger chlorine substituent in **20** was detrimental, suggesting that substitutions at the 6-position may introduce unfavorable interactions that offset the desired preconfiguration.

**Table 2:**
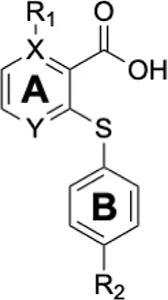

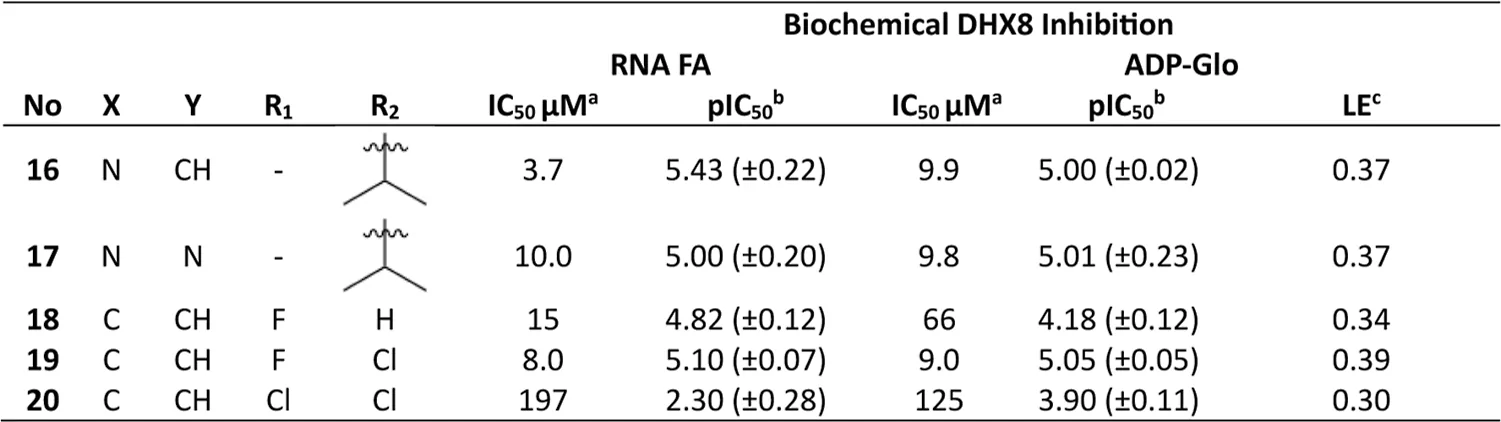
Investigating the Carboxylic Acid Conformation^a^

a DHX8 fragment binding was confirmed using a fluorescence assay based on a fluorescently labeled poly­(A) ssRNA substrate. The effect of fragment binding on DHX8 ATP hydrolysis was characterized using an ADP-Glo assay. Data represent the geometric mean IC_50_ (^a^) or arithmetic mean pIC_50_ ± standard deviation (^b^) of at least three replicates. Ligand Efficiency (^c^) was calculated based on the ADP-Glo pIC_50_ data, using the formula LE = (1.4pIC_50_)/(number of non-hydrogen atoms).

### Extending into the RNA-Binding Tunnel

We investigated further B-ring optimization and discovered that increasing its size to a naphthyl, as in **21**, significantly improved potency compared to its matched pair **7** (Table 3). A crystal structure of **21** bound to DHX8 at 2.85 Å resolution showed the A-ring binding in the same manner as observed before (Figure 4C). The naphthyl moiety adopts a single conformation in two of the independent DHX8 molecules in the asymmetric unit (Figure 4C), whereas in the other two DHX8 molecules, it can be modeled in two distinct conformations (Figure 4D). Regardless of its conformation, the naphthyl group binds between the side chains of His693 and Arg647, like the B-ring of the other fragments, orienting the Arg647 side chain for a face-to-face interaction.

**Table 3:**
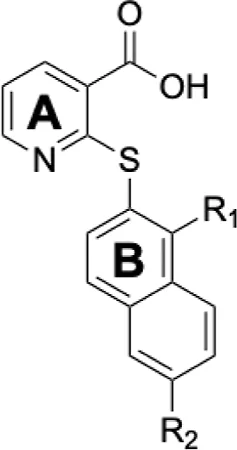

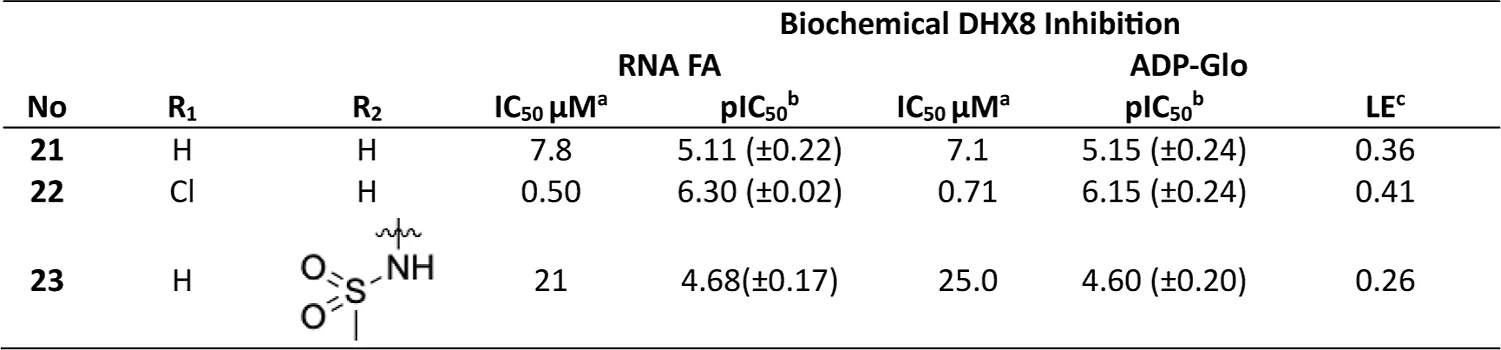
Exploring the Fragment B-Ring^a^

a DHX8 fragment binding was confirmed using a fluorescence assay based on a fluorescently labeled poly­(A) ssRNA substrate. The effect of fragment binding on DHX8 ATP hydrolysis was characterized using an ADPGlo assay. Data represent the geometric mean IC_50_ (^a^) or arithmetic mean pIC_50_ ± standard deviation (^b^) of at least three replicates. Ligand Efficiency (^c^) was calculated based on the ADP-Glo pIC_50_ data, using the formula LE = (1.4pIC_50_)/(number of non-hydrogen atoms).

In addition, the second ring of the naphthyl group stacks against the main chain of Pro984 and Leu985 of a loop lining the small hydrophobic pocket in the Winged-Helix domain. To strengthen these interactions, we introduced a chlorine ortho substituent on the naphthyl ring yielding **22**, which was the first compound in this series with submicromolar potency in both biochemical assays. As the tip of the naphthyl group in **21** is located close to the entrance of the Winged-Helix pocket, which in our ligand-bound DHX8 structures typically binds an ethylene glycol or DMSO molecule, we attempted to mimic a bound DMSO molecule by introducing a methyl-sulfonamide substituent, as in **23**. However, this was not beneficial (Table 3). Despite extensive efforts, **22** remained our best compound, and all further attempts to improve the potency of this fragment series were unsuccessful.

To overcome the potency plateau, we reanalyzed our fragment analogues and identified **24** (Table 4), a ligand efficient (LE = 0.35) compound from the ICR HTS library, which can be viewed as a deconstruction of **21** with a more flexible phenethyl group replacing the directly linked B-ring motif. It is also similar to compound **7**, with the sulfur linker extended by two CH_2_ groups. Compared to **7**, **24** showed a 3- and 6-fold potency increase in the respective RNA-FA and ADP-Glo assays (Table 4). A **24**-bound DHX8 crystal structure at 2.97 Å resolution confirmed the familiar A-ring binding mode (Figure 5A). The extended linker in **24** binds between the side chains of Arg647 and His693, positioning the B-ring deeper into the RNA-binding tunnel, close to the Winged-Helix pocket. Compound **25**, a matched pair of **24** with a phenyl instead of a pyridine A-ring, confirmed the potency of the extended linker compounds with the potential to more fully occupy the RNA-binding tunnel; therefore, we decided to investigate their potential to provide a lead series of potent DHX8 inhibitors.

**Table 4:**
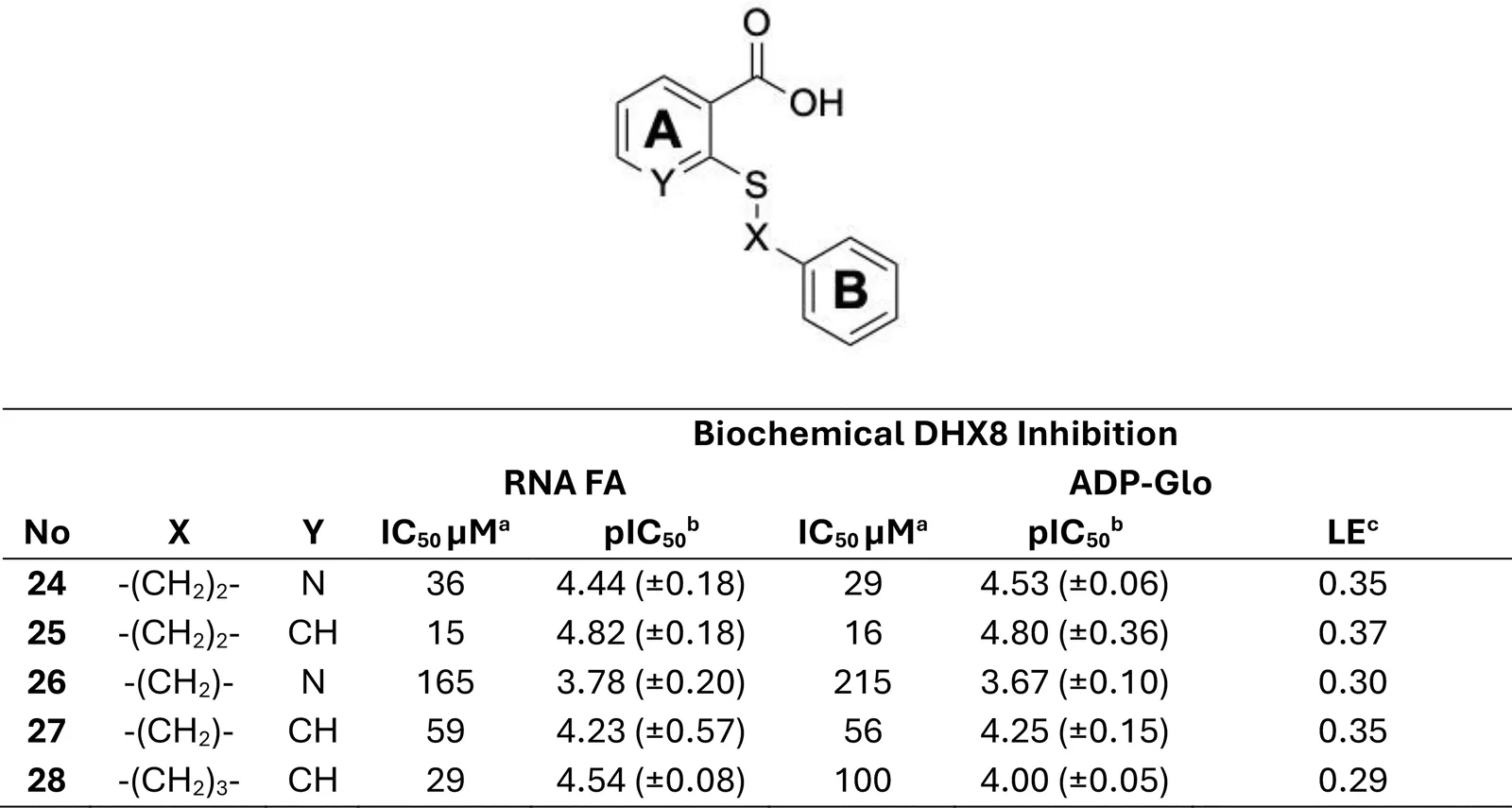
Extended Linker Series, Exploring Linker Length^a^

a DHX8 binding was confirmed using a fluorescence assay based on a fluorescently labeled poly­(A) ssRNA substrate. The effect of compound binding on DHX8 ATP hydrolysis was characterized using an ADP-Glo assay. Data represent the geometric mean IC_50_ (^a^) or arithmetic mean pIC_50_ ± standard deviation (^b^) of at least three replicates. ^c^Ligand Efficiency was calculated based on the ADP-Glo pIC_50_ data, using the formula LE = (1.4pIC_50_)/(number of non-hydrogen atoms)

**Figure 5:**
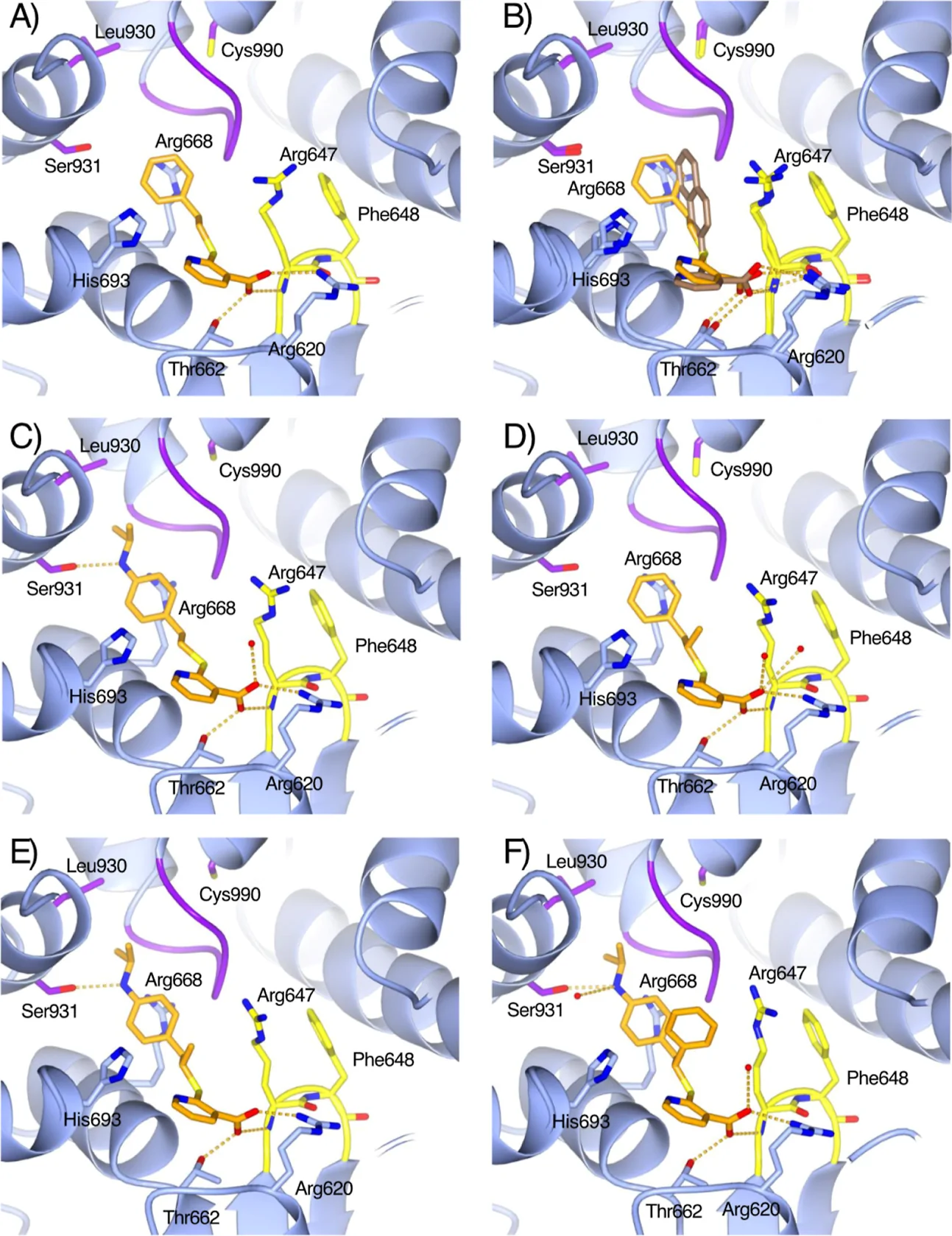
Extended linker series. (A) X-ray structure of DHX8 bound to compound **24** (PDB ID 28ZO). (B) Overlay of DHX8 structures bound to **21** and **24**. (C) X-ray structure of DHX8 bound to compound **34** (PDB ID 28ZP). (D) X-ray structure of DHX8 bound to compound **43** (PDB ID 28ZQ). (E) X-ray structure of DHX8 bound to compound **51** (PDB ID 28ZR). (F) X-ray structure of DHX8 bound to compound **53** (PDB ID 28ZS). The compounds are shown in cylinder representation with carbon atoms in orange, except for compound **21**, which is displayed with carbon atoms in brown. The hook-turn motif is shown in yellow and the loop and residues defining the pocket in the Winged-Helix domain are shown in purple. Hydrogen bonds are shown as yellow dotted lines and water molecules are displayed as red spheres. Key residues are labeled.

First, we assessed the impact of the linker length on the potency (Table 4). Matched pairs **24**/**26** and **25**/**27** showed that shortening the linker by one CH_2_-group was detrimental. Extending the linker with an additional CH_2_-group as in compound **28** also led to a drop in potency and ligand efficiency in the ATPase assay, although not in the RNA FA assay. We therefore concluded that neither linker shortening nor extension was beneficial and retained the ethanethiol linker.

Our next aim was to improve the potency of **24**, which displayed favorable physicochemical properties (Table S1). While we recognized the carboxylic acid as a key binding element, it could in principle limit cellular permeability, tissue distribution, and cause toxicity through metabolic activation.^22^ As risk mitigation, we employed a design strategy that restricted molecular weight to ≤400 Da, targeted a defined lipophilicity range [log*D*_7.4_ (1 – 3)], and routinely used a standard suite of *in vitro* ADME assays to monitor progress. A comparison of the **21**- and **24**-bound DHX8 structures shows an approximately 70° rotation of the B-ring in **24** as compared to the naphthyl group of **21** (Figure 5B). This results in a less productive interaction with the main chain of Pro984 and Leu985, which might explain the lower potency of **24**. However, the **24** B-ring forms a better hydrophobic interaction with the side chain of Arg668, and more importantly, the tip of the phenyl B-ring is positioned close to the entrance of the Winged-Helix pocket, making substitution of its para-position an attractive option to further extend into it.

Using the Viper ligand design software,^23^ an automated fragment scanning method for structure-based lead optimization, we identified the side chain of Ser931 at the entrance of the Winged-Helix pocket as a potential binding hotspot that could be targeted with short aliphatic ether or NH-containing B-ring para-substituents containing hydrogen bond donors or acceptors (Figure S6). Based on the Viper analysis, compounds **29**–**31** with NH-containing substituents aimed at targeting the Ser931 side chain showed improved potency compared to **24** (Table 5). The benefit of hydrogen bond donors in this position was further supported by the reduced affinity of compound **32**, which cannot form the Ser931 H-bond interaction. Compound **33**, with a larger cyclopentylamine para-substituent intended to better fill the Winged-Helix pocket, also had improved potency compared to **24**. With IC_50_ values of 1.2 and 2.3 μM in the respective RNA FA and ATPase assays, one of the best compounds was **34**, which contained an isopropylamino para-substituent on the B-ring, identified by the Viper analysis. The 2.57 Å X-ray structure of **34** bound to DHX8 confirmed an overall binding mode similar to **24** and highlighted the excellent fit of the isopropylamino para-substituent in the Winged-Helix pocket (Figures 5C, S7). In addition, the X-ray crystal structure revealed that the NH-moiety formed the hypothesized hydrogen bond with the hydroxyl side chain of Ser931 at the entrance of the pocket (Figure 5C). Moreover, the lower affinity of **35**, which has an oxygen-containing isopropyl ether C11 substituent, confirmed that an NH-containing para-substituent is optimal to exploit this interaction. Pleasingly, compound **34** showed high solubility and high permeability in a Caco-2 cell permeability assay and low to medium intrinsic clearance in human and mouse hepatocytes, respectively (Table S1).

**Table 5:**
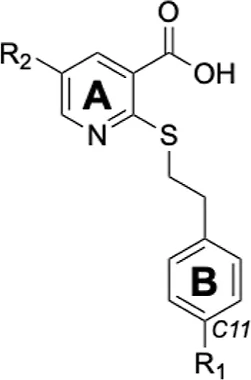

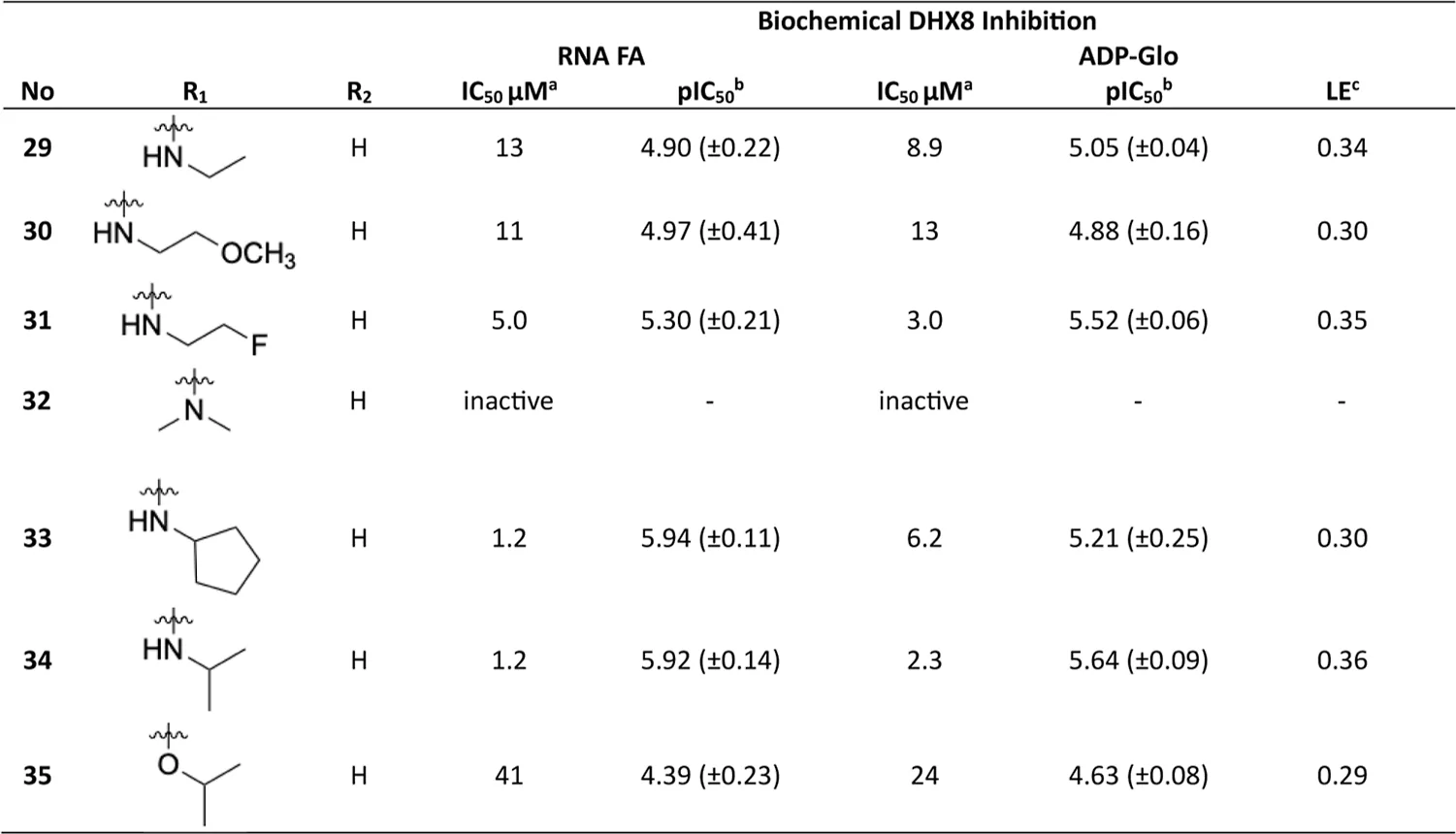
Accessing the Pocket in the Winged-Helix Domain^a^

a DHX8 binding was confirmed using a fluorescence anisotropy assay based on a fluorescently labeled poly­(A) ssRNA substrate. The effect of compound binding on DHX8 ATP hydrolysis was characterized using an ADP-Glo assay. Data represent the geometric mean IC_50_ (^a^) or arithmetic mean pIC_50_ ± standard deviation (^b^) of at least three replicates. ^c^Ligand Efficiency was calculated based on the ADP-Glo pIC_50_ data, using the formula LE = (1.4pIC_50_)/(number of non-hydrogen atoms).

### Substituting the Extended Linker

In parallel with the efforts to grow **24** into the Winged-Helix pocket, we explored substitutions on the carbon atoms of the ethanethiol linker (Table 6). A simple methyl substitution at the C7 position, as in **36**, proved detrimental to affinity, which is consistent with the limited space in this part of the binding site observed in the **24**-bound DHX8 crystal structure. By contrast, substitution of the linker C6 position to access the space between the side chains of His693 and Arg647 was more successful. A similar methyl substituent at the C6 position, as in **37**, yielded 14- and 7-fold improvements in potency in the ATPase and RNA FA assays, respectively. Further substitution with different hydrophobic and hydrophilic groups yielded compounds **38**–**40**, all with single digit micromolar IC_50_ values in both the ATPase and RNA FA assays. However, double substitution of the C6 position, as in the spirocycle **41**, resulted in a significant drop in potency compared to the single C6 substituents, most likely caused by steric clashes with the protein. This led us to re-examine our compounds with a single C6 substitution that created a chiral center, as they had been initially tested as racemic mixtures. Indeed, compared to the racemic methyl-substituted **37**, its single *S*-enantiomer **43** yielded comparable potencies, whereas the potency of the *R*-enantiomer **42** was 6- and 4-fold weaker in the two assays (Table 6). This is consistent with the 2.20 Å crystal structure of the *S*-enantiomer **43** bound to DHX8, which showed the methyl group sandwiched between the side chains of His693 and Arg647 with the conformationally flexible Arg647 side chain packing against the methyl group in an orientation also observed in the naphthyl-containing **21** (Figure 5D). Assuming the same overall binding mode, the methyl substituent in the *R*-enantiomer **42**, would most likely cause a steric clash with the main chain of Gly664 and the side chain of His693, thus explaining the difference in potency between the two enantiomers. This was further substantiated by compounds **44** and **45**, the single enantiomers of the racemic **38** with a phenyl C6 substituent. Based on the crystal structure of *S*-enantiomer **43**, the larger phenyl group in **45** would point into the available space of the RNA-binding tunnel whereas the phenyl in the opposite enantiomer **44** would cause a clash with the protein similar to the one postulated for **42**. This is substantiated by the potency difference between **44**, which has a potency in the double-digit micromolar range, and **45** which has IC_50_ values of 0.23 μM and 0.53 μM in the RNA-FA and ADP-Glo assays, respectively.

**Table 6:**
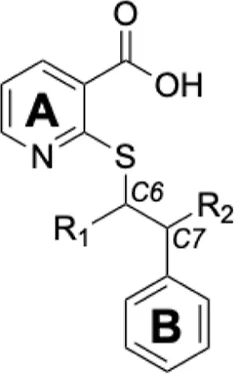

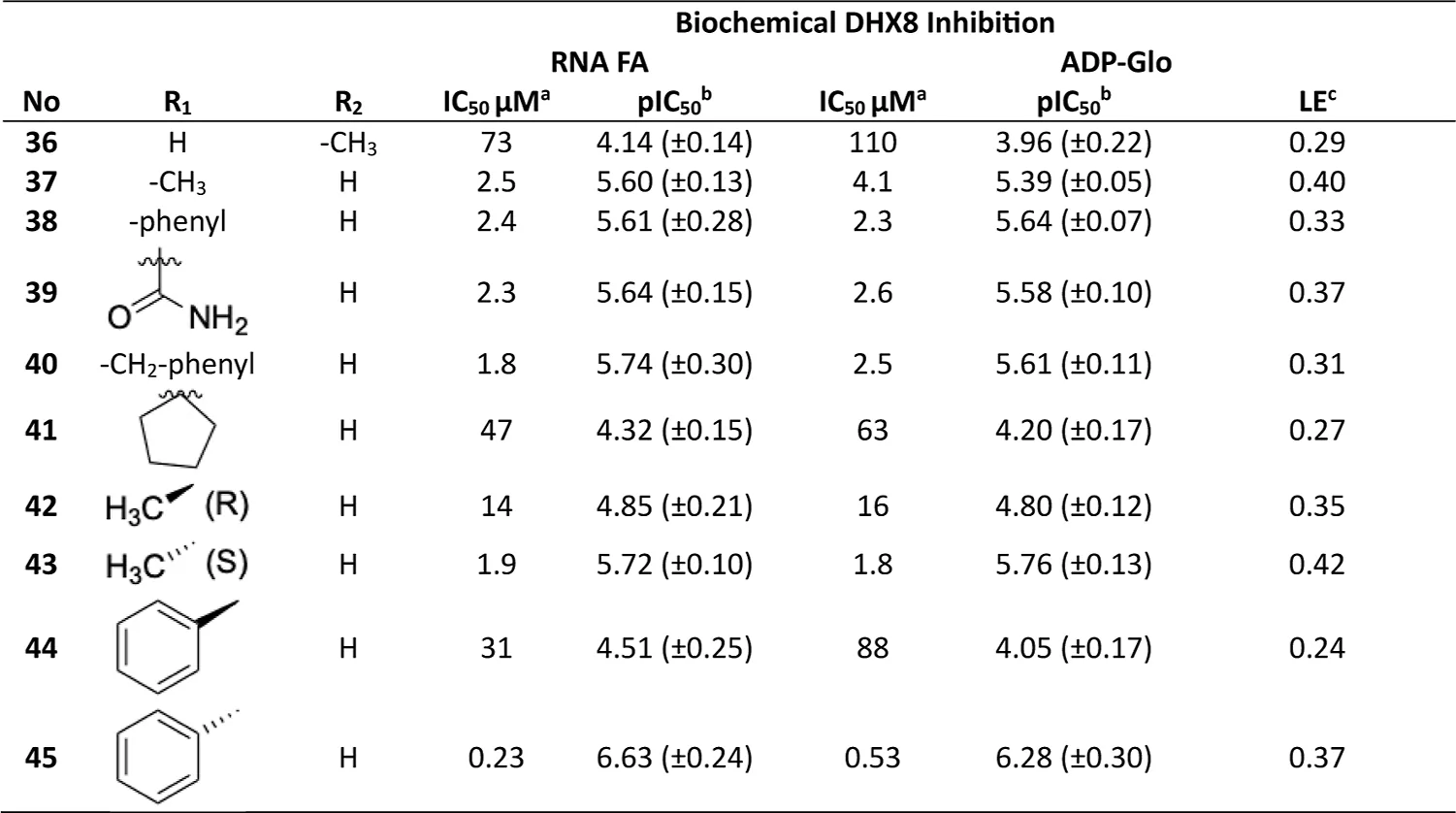
Accessing the Pocket in the Winged-Helix Domain^a^

a DHX8 binding was confirmed using a fluorescence anisotropy assay based on a fluorescently labeled poly­(A) ssRNA substrate. The effect of compound binding on DHX8 ATP hydrolysis was characterized using an ADP-Glo assay. Data represent the geometric mean IC_50_ (^a^) or arithmetic mean pIC_50_ ± standard deviation (^b^) of at least three replicates. ^c^Ligand Efficiency was calculated based on the ADP-Glo pIC_50_ data, using the formula LE = (1.4pIC_50_)/(number of non-hydrogen atoms).

### Potent DHX8 Inhibitors Showing Cellular Target Engagement

Our next step was to combine the optimal isopropylamino moiety in the para-position of the B-ring with additional modifications at C6 to increase potency by exploiting the available space deep within the RNA-binding tunnel. Combining the isopropylamino group with an ethyl group at C6 yielded **46**, which has a similar potency compared to the isopropylamino substituted **34** and to **37** with the single C6 methyl substitution (Table 7). Notably, compounds **47**–**49**, with larger C6 substituents, all yielded IC_50_ values less than 0.5 μM in both the biochemical RNA FA and ATPase assays and despite their increased size retained a ligand efficiency better than 0.30.

**Table 7:**
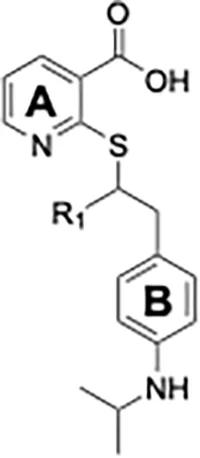

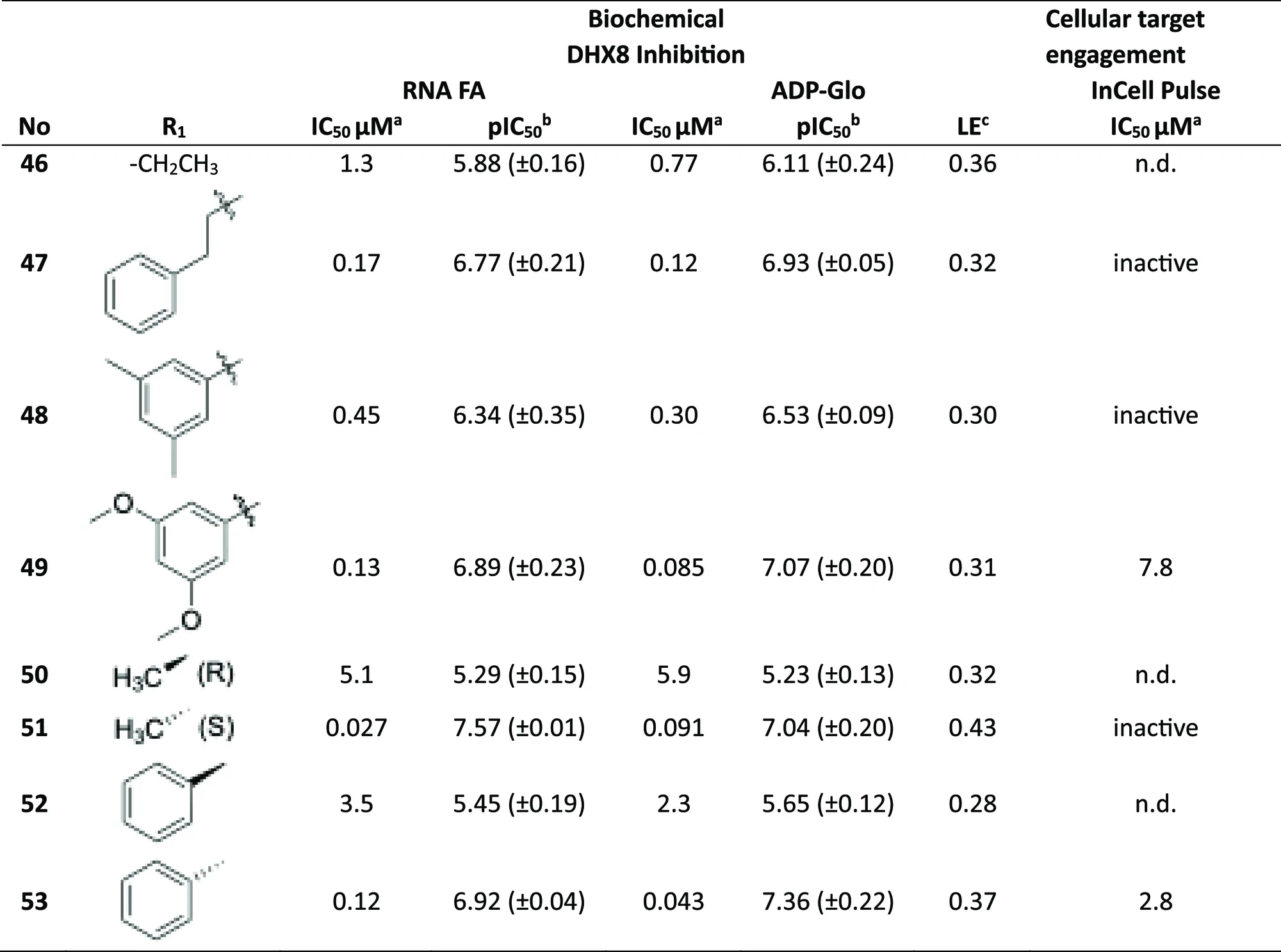
Potent DHX8 Inhibitors^a^

a DHX8 binding was confirmed using a fluorescence anisotropy assay based on a fluorescently labeled pol­(A)­ssRNA substrate. The effect of compound binding on DHX8 ATP hydrolysis was characterized using an ADPGlo assay. Cellular target engagement of DHX8 inhibitors was measured in H1299 lung cancer cells using an InCell Pulse assay (n.d. = not determined). Data represent the geometric mean IC_50_ (^a^) or arithmetic mean pIC_50_ ± standard deviation (^b^) of at least three replicates. ^c^Ligand Efficiency was calculated based on the ADP-Glo pIC_50_ data, using the formula LE = (1.4pIC_50_)/(number of non-hydrogen atoms). n.d. = not determined.

For expediency, **46**–**49** were generated as racemic mixtures but we previously established that the C6 substituted *S*-enantiomers in this series showed better potency than their *R*-enantiomeric counterparts, which is most likely due to steric clashes of the C6 substituents in the *R*-enantiomers with DHX8. This trend is also observed in the combined compounds and exemplified by *R*-enantiomer **50**, which was more than 180-fold less potent in the RNA-FA assay and 60-fold less potent in the ATPase assay compared to its *S*-enantiomer **51**, which gave respective IC_50_ values of 27 and 91 nM (Table 7). The 2.45 Å crystal structure of **51** bound to DHX8 confirmed the expected binding mode with the linker C6 methyl substituent sandwiched between His693 and Arg647 and the B-ring para-isopropylamino group forming a hydrogen bond with Ser931 and occupying the Winged-Helix pocket (Figure 5E). In addition, compound **51** showed high aqueous solubility, high symmetrical permeability in Caco-2, and low to medium intrinsic clearance in human and mouse hepatocytes, respectively (Table S1). Combining a phenyl C6 substituent, as in compounds **44** and **45**, with the isopropylamino para-substituent yielded **52** and its more potent enantiomer **53**. Relative to **51**, the introduction of the aryl ring in **53** increased lipophilicity (log*D*_7.4_ + 0.8) without negatively affecting the high aqueous solubility (Table 8). However, permeability in Caco-2 decreased from high to medium and showed signs of efflux (efflux ratio of 5.0). Intrinsic clearance in human hepatocytes remained low, whereas it was high in mouse hepatocytes. The 2.50 Å crystal structure of **53** bound to DHX8 confirmed its binding mode with the phenyl group binding between the side chains of His693 and Arg647, orienting the arginine’s side chain, and pointing into the wider space of the RNA-binding tunnel (Figure 5F).

**Table 8:**
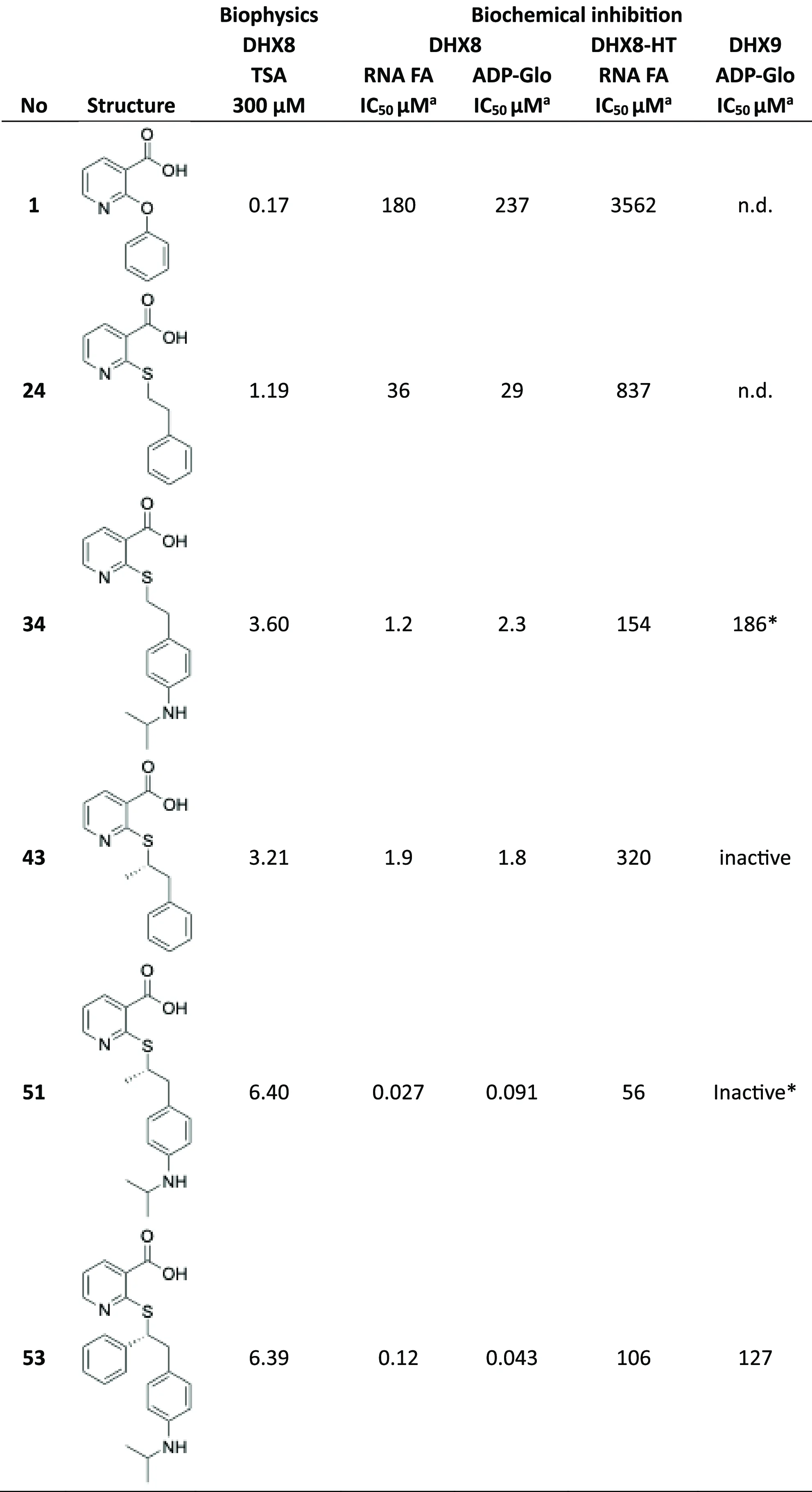
Effect of Inhibitor Binding on DHX8 Stabilization, Potency against a DHX8 Hook-Turn Mutant, and DHX9 Compared to Wild-Type DHX8^a^

a Comparison of thermal stabilization of the DHX8 helicase domain DHX8Δ547 was carried out using a thermal shift assay at different inhibitor concentrations. The effect of compound binding to a hook-turn mutant DHX8 helicase domain was explored using a fluorescently labeled poly­(A) ssRNA substrate. The effect of compound binding on full length DHX9 was explored using an ADP-Glo assay. The values for the respective assays against DHX8 helicase domain are shown for reference. ^a^Data represent the geometric mean of at least three replicates, unless otherwise stated. *Data is *n* = 2.

To test if the high biochemical potency of the advanced extended linker compounds translated into cellular target engagement, compounds with IC_50_ values better than 0.3 μM, which included our two biochemically most potent compounds **51** and **53**, were profiled in a DHX8 InCell Pulse cellular target engagement assay (Table 7) that we established in H1299 human non-small cell lung cancer (NSCLC) cells, which we previously reported to be DHX8-dependent.^7^ The InCell Pulse assay utilizes DHX8Δ547 fused with a β-galactosidase fragment tag.^24,25^ After treatment with a heat pulse, an enzyme acceptor is added to detect β-galactosidase activity and allow the quantification of ligand-induced thermal stabilization of the target in a cellular context. To our surprise, **51** was inactive but, pleasingly, the racemic compound **49** showed DHX8 cellular target engagement in the 10 μM range. Overall, the enantiomerically pure **53**, which like **49** has a large C6 linker substituent, was our best compound with biochemical IC_50_ values of 120 nM and 43 nM in the respective RNA FA and ATPase assays, demonstrating cellular target engagement in H1299 cancer cells with an IC_50_ of 2.8 μM in the InCell Pulse assay.

### Mode of Action of RNA-Competitive DHX8 Inhibitors

To rationalize the mode of action of our DHX8 inhibitors, we conducted an analysis of publicly available structures of spliceosomal DEAH-box RNA helicases. In all DEAH-box helicases, the helicase core undergoes a series of conformational rearrangements during RNA translocation, which involves the opening and closing of the RNA-binding tunnel.^26^ A study on yeast Prp43, the functional homologue of human DHX15, demonstrated that in its postcatalytic ADP-bound and RNA/ATP-bound states the helicase forms an enclosed RNA-binding tunnel.^27^ By contrast, a structure representing the Prp43 ATP-bound state, revealed a conformation of the helicase core in which the C-terminal ratchet-like and OB-fold domains are shifted by more than 10 Å (Figure S8A). This significant movement changes the RNA-binding site from an enclosed tunnel to an open RNA-binding groove and is accompanied by smaller changes in the Winged-Helix domain, which acts as a hinge between the open and closed conformations of the helicase core.

Although the Prp43 structures representing the ADP- and RNA/ATP-bound states both adopt a closed conformation, the RNA-bound structure is slightly more open with differences in the conformations of the ratchet-like and OB-fold domains and a backward shift of the Winged-Helix hinge domain by approximately 3 Å (Figure S8B). Similar differences are present between the ADP-, or compound-bound structures, as compared to the RNA-bound structure of human DHX8 (Figure S8C). Indeed, a superimposition of the **53**- and RNA-bound DHX8 structures also shows an ∼3.3 Å backward shift in residues lining the Winged-Helix domain pocket. The location of the pocket in the more open RNA-bound DHX8 structure is incompatible with binding of the amino-isopropyl moiety of DHX8 inhibitors such as **53** (Figure 6A). This suggests that extended linker inhibitors like **53** stabilize a closed conformation of DHX8 by restricting the motions of the Winged-Helix hinge domain and blocking the opening of the RNA-binding tunnel. This hypothesis is supported by thermal shift assay data, which shows a clear correlation between the increase in inhibitor potency resulting from successful fragment elaboration into the Winged-Helix pocket and DHX8 stabilization (Table 8). Particularly, the addition of the isopropylamino group to compound **24**, yielding **34**, resulted in an increase in DHX8 thermal stabilization of 2.4 °C.

**Figure 6:**
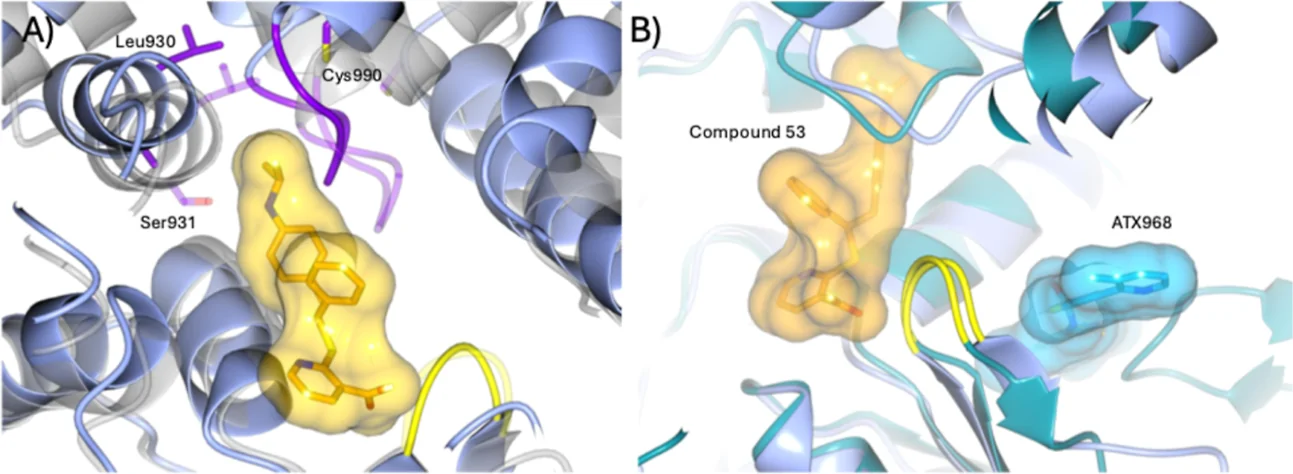
Mode of action of DHX8 and DHX9 RNA helicase inhibitors. (A) Movement of the Winged-Helix pocket in different DHX8 states in the RNA translocation cycle. Superposition of DHX8 structures bound to **53** (PDB ID 28ZS) and ssRNA (PDB code 6HYU). In the DHX8 **53**-bound structure DHX8 is shown in gray, **53** is shown in cylinder representation with carbon atoms in orange with a semitransparent orange solvent-accessible surface superimposed. DHX8 in the RNA-bound structure is shown in blue. The ssRNA substrate has been omitted for clarify. In both structures, loop and key residues of the Winged-Helix pocket are shown in purple, highlighting the movement of the pocket between the two DHX8 structures. (B) Superposition of the crystal structures of DHX8 (blue) structure bound to **53** (orange) and DHX9 (turquoise) bound to ATX968 (cyan) showing that both inhibitors interact with the conserved hook-turn motif (yellow), with the DHX8 inhibitor **53** binding inside the RNA-binding tunnel and ATX968 outside the tunnel in an allosteric pocket on the protein surface.

Moreover, a 2.0 °C increase in stabilization was observed between **24** and **43** further highlighting that substituents that optimize inhibitor binding between His693 and the side chain of Arg647 within the hook-turn motif are important for their postulated mode of action. Indeed, characterization of our DHX8 inhibitors in an RNA-FA assay using the previously reported DHX8Δ547-HT mutant, in which the hook-turn residues Arg647 and Phe648 were mutated to glycine residues, showed a drop-off in IC_50_ values between 20 and 2000-fold, compared to the RNA FA assay for wild-type DHX8 (Table 8).

### Comparison with Other DExH-Box Helicase Inhibitors

We next compared our DHX8 inhibitors with the most advanced and only other structurally characterized DExH-box RNA helicase inhibitor, the DHX9 inhibitor ATX968.^28,29^ DHX9 is a DExH-box helicase involved in the unwinding of double-stranded RNA and DNA substrates, RNA/DNA hybrids such as R-loops, and G-quadruplexes. It is a validated target for patients with tumors characterized by a high level of microsatellite instability (MSI-H) and deficient DNA mismatch repair. ATX968 is an allosteric inhibitor that only partially inhibits DHX9 ATP-hydrolysis in a biochemical ADP-Glo assay. We synthesized ATX968 based on a patent describing DHX9 inhibitors^30^ and confirmed its potent but partial inhibition of DHX9 in our ATPase assay (Table S2). Consistent with its reported allosteric mode of action, ATX968 did not displace the FP-probes in our ATP- and RNA-binding site assays adapted for DHX9 (Table S2). Since at the time, the patent did not describe the DHX9 binding site of ATX968, we cocrystallized ATX968 with a published truncated DHX9 variant (residues 150 – 1150) comprising the N-terminal dsRNA-binding domain 2 (dsRBD2), the minimal transactivation domain (MTAD), and the complete helicase domain.^31^ As reported, crystallization of human DHX9 is extremely challenging, generally yielding only weakly diffracting crystals. Nevertheless, a diffraction screen of hundreds of crystals yielded a single 3.4 Å data set from which we determined an ATX968-bound human DHX9 crystal structure (Figure S9A). By contrast, a parallel cryoEM approach quickly yielded a 2.7 Å human DHX9 cryoEM structure in which both ATX968 and ADP were clearly defined from the first sets of grids (Figure S9B). Importantly, this illustrates that in SBDD cases where protein-ligand crystallography of the target is challenging, a cryoEM approach can provide impactful structural insights in protein-inhibitor binding and should be tried in parallel.

In both DHX9 structures, the binding of ATX968 is nearly identical to that in the recently published ATX968-bound human DHX9 crystal structure (PDB ID 9MFT).^29^ As reported, ATX968 binds in an allosteric pocket between the RecA1 and MTAD domains to a closed conformation of the DHX9 helicase core.^29^ It packs against the base of the conserved hook-turn motif on the outside of the RNA-binding tunnel and binds via an extensive network of hydrogen bonds and hydrophobic interactions with residues from both domains (Figures S9, 6B). DHX8 does not contain an MTAD domain, which explains why ATX968 is inactive against DHX8 (Table S2). Conversely, our DHX8 inhibitors, which also bind to the hook-turn motif, but inside the RNA-binding tunnel (Figure 6B), showed a steep drop-off in IC_50_ values when tested in the DHX9 ADP-Glo ATPase assay (Table 8). This is perhaps surprising, given the conserved nature of the inhibitor binding site. However, this is consistent with the corresponding residue of His693 in DHX8 being an asparagine (Asn519) in DHX9. It is therefore possible that the Arg474/Asn519 pair in DHX9 is not as effective in locking the DHX8 inhibitors onto its RecA1 domain as the Arg647/His693 pair in DHX8. As His693 is not conserved across the DExH-box helicase family (Figure S10), our results suggest that biochemical selectivity between individual DEAH/DExH-box family members can be achieved with RNA-competitive inhibitors.

## Conclusions

As far as we are aware, this study describes the discovery of the first RNA-competitive DEAH-box RNA helicase inhibitors and the first DHX8 inhibitors with nanomolar potency showing target engagement in cells. The initial hit **1** identified in parallel TSA and SPR biophysical fragment screens against the spliceosomal DEAH-box RNA helicase DHX8 was successfully confirmed by SPR concentration-response and ligand-observed NMR experiments. Structure determination of **1** bound to the DHX8 helicase domain confirmed its binding in the RNA-binding tunnel near the conserved hook-turn motif. The binding mode of **1** mimics the binding of an RNA nucleotide from a bound ssRNA substrate with its carboxylic acid group anchored in the binding site of the nucleotide’s RNA backbone phosphate group and its hydrophobic scaffold overlapping with the binding site of the nucleotide’s ribose moiety.

Crucial to improving the potency of our initial fragment hit was a structure-guided fragment-growing approach across the RNA-binding tunnel, which informed the expansion of fragment hits into a small pocket in the DHX8 Winged-Helix domain. Further potency improvements were achieved by optimizing π-stacking aromatic interactions between the side chains of Arg647 and the nonconserved His693, both located in the RecA1 domain. In combination, this yielded nanomolar DHX8 inhibitors with good *in vitro* PK and activity in an InCell Pulse cellular target engagement assay.

We hypothesize that by binding into both the Winged-Helix hinge domain pocket and to the conserved hook-turn motif, our DHX8 inhibitors restrict the conformational plasticity of the helicase domain necessary for processive translocation of ssRNA and stabilize it in a closed inactive conformation. In addition, they bind in the RNA-binding tunnel in a manner that sterically blocks the translocation of an ssRNA substrate.

A structural comparison with the allosteric DHX9 inhibitor ATX968 showed that both the DHX8 and DHX9 inhibitors bind close to the hook-turn motif. However, our DHX8 inhibitors bind inside the DHX8 RNA-binding tunnel, whereas the DHX9 inhibitor ATX968 binds outside the RNA-binding tunnel in an allosteric pocket between the DHX9 RecA1 and MTAD domains. Thus, these compounds inhibit their respective helicase targets in a different manner while both stabilize a closed confirmation of the helicase core. Importantly, the absence of the MTAD domain in DHX8 and other DEAH-box RNA helicases explains the selectivity of ATX968 for DHX9. Moreover, the importance of the poorly conserved His693 residue in the binding of our optimized DHX8 inhibitors suggests that biochemical selectivity between individual DEAH-box family members is achievable with RNA-competitive inhibitors.

## Experimental Section

### General Synthetic Chemistry

Reactions were carried out under N_2_ at room temperature unless otherwise stated. All anhydrous solvents and reagents were obtained from commercial suppliers and used without further purification. Compounds **1**–**12**, **18**–**21**, **24**, and **26**–**28** were sourced from commercial suppliers. Evaporation of solvents was carried out using a rotary evaporator under reduced pressure at a water bath temperature of up to 50 °C. Flash column chromatography was carried out on Biotage purification systems using prepacked Sfär Silica HC D cartridges. For the reversed-phase mode Sfar̈ C18D cartridges were used. Prep-HPLC purification was carried out on a Shimadzu LC-20AD, equipped with either XSelect CSH C18 OBD, XBridge OBD C18, or XBridge Shield RP18 OBD Prep Columns. Chiral separation was carried out on a Shimadzu LC-20AT. Microwave-assisted reactions were performed using a Biotage initiator microwave system. Final compounds were purified to ≥ 95% purity by HPLC analysis. NMR data were collected either on a Bruker AVANCE 500 spectrometer equipped with a 5 mm BBO/QNP probe, or on a Bruker AVANCE Neo 600 spectrometer equipped with a 5 mm TCI Cryo-Probe, or on a Bruker AVANCE III HD 400 MHz. NMR data are presented in the form of chemical shift δ (multiplicity, coupling constants, integration) for major diagnostic protons, given in parts per million relative to tetramethylsilane, referenced to the internal deuterated solvent. High-resolution mass spectrometry (HRMS) was assessed using either an Agilent 1200 series HPLC and a diode array detector coupled to a 6120 time of a flight mass spectrometer with a dual multimode APCI/ESI source, or on a Waters Acquity UHPLC and a diode array detector coupled to a Waters G2 QToF mass spectrometer fitted with a multimode ESI/APCI source, or a Shimadzu LCMS-2020. The synthesis of intermediates for compounds **29**–**32**, **34**, **38**, **40**, **46**–**50**, and **52** is described in the Supporting Information, whereas the final synthetic preparations for each compound are included below.

### Preparation of Compounds

#### 2-((4-Isopropylphenyl)­thio)­nicotinic Acid (**13**)

To a solution of methyl 2-chloronicotinate (80.0 μL, 0.613 mmol, 1.0 equiv) in DMA (1.30 mL, 0.47 M) in a microwave vial were added 4-isopropylbenzenethiol (0.110 mL, 0.735 mmol, 1.2 equiv) and Cs_2_CO_3_ (299 mg, 0.919 mmol, 1.5 equiv). The reaction mixture was stirred at 75 °C for 1.5 h. The reaction mixture was diluted with water and washed with EtOAc. The aqueous layer was separated, acidified with aqueous 1 M HCl solution and extracted with EtOAc. The organic phase was separated, washed with brine, dried over MgSO_4_, filtered, and concentrated under reduced pressure. The crude material was purified by reversed-phase column chromatography (Biotage Isolera One, 12 g Sfär C18 silica column, H_2_O + 0.1% formic acid/MeOH + 0.1% formic acid, 20–80%) to isolate 2-((4-isopropylphenyl)­thio)­nicotinic acid (82.0 mg, 47%). ^1^H NMR (600 MHz, DMSO-*d*_6_): δ 8.41 (dd, *J* = 4.7, 1.8 Hz, 1H), 8.22 (dd, *J* = 7.7, 1.9 Hz, 1H), 7.43–7.38 (m, 2H), 7.33–7.29 (m, 2H), 7.22 (dd, *J* = 7.8, 4.7 Hz, 1H), 2.94 (p, *J* = 6.9 Hz, 1H), 1.24 (d, *J* = 6.9 Hz, 6H). ^13^C NMR (151 MHz, DMSO-*d*_6_): δ 166.4, 161.1, 152.0, 149.1, 139.0, 135.7, 127.6, 127.1, 123.1, 119.6, 33.2, 23.7. HRMS (*m*/*z*): [M + H]^+^ calcd for C_16_H_15_NO_2_S, 274.0896; found, 274.0902.

#### 2 2-((4-Isopropylphenyl)­thio)­benzoic Acid (**14**)

To a solution of 2-iodobenzoic acid (100 mg, 0.403 mmol, 1.0 equiv) in DMF (1.00 mL, 0.4 M) were added 4-isopropylbenzenethiol (56.4 μL, 0.484 mmol, 1.2 equiv), CuI (7.68 mg, 0.040 mmol, 0.1 equiv), and Cs_2_CO_3_ (262 mg, 0.806 mmol, 2.0 equiv) and the reaction was stirred at 80 °C for 1.5 h. After this time, the reaction mixture was diluted with EtOAc and washed with water, the aqueous layer was separated, acidified with aqueous 1 M HCl solution, and washed with EtOAc. The organic layers were combined, washed with brine and dried over MgSO_4_, then filtered and concentrated under reduced pressure. The crude material was purified by reversed-phase column chromatography (Biotage Isolera One, 12 g Sfär C18 silica column, eluted with H_2_O + 0.1% formic acid/MeOH + 0.1% formic acid, 20–80%) to isolate 2-((4-isopropylphenyl)­thio)­benzoic acid (50.0 mg, 43%). ^1^H NMR (600 MHz, DMSO-*d*_6_): δ 13.17 (s, 1H), 7.91 (dd, *J* = 7.7, 1.6 Hz, 1H), 7.46 (d, *J* = 8.2 Hz, 2H), 7.40–7.35 (m, 3H), 7.19 (td, *J* = 7.5, 1.1 Hz, 1H), 6.72 (dd, *J* = 8.2, 1.1 Hz, 1H), 2.96 (p, *J* = 6.9 Hz, 1H), 1.25 (d, *J* = 6.9 Hz, 6H). ^13^C NMR (151 MHz, DMSO-*d*_6_): δ 167.4, 149.9, 142.3, 135.3, 132.4, 130.9, 128.8, 128.8, 126.5, 124.5, 33.2, 23.7. HRMS with negative ionization mode not available.

#### 2-((2,4-Dichlorophenyl)­thio)­benzoic Acid (**15**)

2-Iodobenzoic acid (100 mg, 0.403 mmol, 1.0 equiv), 2,4-dichlorobenzenethiol (50.0 μL, 0.403 mmol, 1.0 equiv), copper (2.56 mg, 0.040 mmol, 0.1 equiv), and potassium hydroxide (115 mg, 2.02 mmol, 5.0 equiv) were combined together in water (3.00 mL). The reaction mixture was stirred at 110 °C for 15 h forming a cream precipitate. The reaction mixture was acidified with aqueous 1 M HCl and extracted with CH_2_Cl_2_ (2 × 15 mL), washed with brine (25 mL), dried over MgSO_4_, filtered, and concentrated to a white solid. The crude mixture was purified by silica gel chromatography (eluted with cyclohexane/EtOAc, 0–100% EtOAc) to afford 2-((2,4-dichlorophenyl)­thio)­benzoic acid (90.0 mg, 71%) as a white solid. ^1^H NMR (500 MHz, DMSO-*d*_6_): δ 13.33 (s, 1H), 7.95 (dd, *J* = 7.8, 1.5 Hz, 1H), 7.87 (d, *J* = 2.2 Hz, 1H), 7.65 (d, *J* = 8.3 Hz, 1H), 7.54 (dd, *J* = 8.3, 2.3 Hz, 1H), 7.42 (ddd, *J* = 8.1, 7.4, 1.6 Hz, 1H), 7.28 (td, *J* = 7.6, 1.1 Hz, 1H), 6.68 (dd, *J* = 8.1, 1.1 Hz, 1H). ^13^C NMR (151 MHz, DMSO): δ 167.4, 138.9, 138.7, 138.1, 135.1, 132.8, 131.1, 130.7, 130.1, 128.8, 128.2, 127.0, 125.5. HRMS Mass ion not detected as product fragments.

#### 3-((4-Isopropylphenyl)­thio)­picolinic Acid (**16**)

To a solution of 3-fluoropyridine-2-carboxylic acid (100 mg, 0.709 mmol, 1.0 equiv) in DMF (1.50 mL, 0.47 M) were added 4-isopropylbenzenethiol (0.130 mL, 0.850 mmol, 1.2 equiv) and Cs_2_CO_3_ (346 mg, 1.06 mmol, 1.5 equiv) and the reaction was stirred at 75 °C overnight. The reaction mixture was diluted with water and washed with EtOAc. Layers were separated, the aqueous was acidified with aqueous 1 M HCl solution and washed with EtOAc. The organic was separated, washed with brine and dried over MgSO_4_, filtered, and concentrated under reduced pressure. The crude mixture was purified by reversed-phase column chromatography (Biotage Isolera One, liquid load, 12g Sfär C18 column, H_2_O + 0.1% formic acid/MeOH + 0.1% formic acid, 20–80%) to afford 3-((4-isopropylphenyl)­thio)­picolinic acid (30.0 mg, 15%). ^1^H NMR (600 MHz, DMSO-*d*_6_): δ 8.41 (dd, *J* = 4.5, 1.4 Hz, 1H), 7.47 (d, *J* = 8.2 Hz, 2H), 7.44 (dd, *J* = 8.3, 4.5 Hz, 1H), 7.40 (d, *J* = 8.2 Hz, 2H), 7.20 (dd, *J* = 8.3, 1.4 Hz, 1H), 2.96 (p, *J* = 6.9 Hz, 1H), 1.24 (d, *J* = 6.9 Hz, 6H). ^13^C NMR (151 MHz, DMSO-*d*_6_): δ 167.1, 150.6, 145.7, 138.3, 135.9, 135.4, 128.8, 128.2, 127.1, 33.7, 24.1. HRMS (*m*/*z*): [M + H]^+^ calcd for C_15_H_15_NO_2_S [M + H]^+^, 274.0896; found, 274.0909.

#### 3-((4-Isopropylphenyl)­thio)­pyrazine-2-carboxylic Acid (**17**)

To a solution of methyl 3-chloropyrazine-2-carboxylate (100 mg, 0.580 mmol, 1.0 equiv) in DMF (1.20 mL, 0.48 M) were added 4-isopropylbenzenethiol (0.110 mL, 0.695 mmol, 1.2 equiv) and Cs_2_CO_3_ (283 mg, 0.869 mmol, 1.5 equiv) and the reaction was stirred at 75 °C for 1.5 h. After this time, the reaction mixture was diluted with water and washed with EtOAc. Layers were separated, the aqueous was acidified with aqueous 1 M HCl solution and washed with EtOAc. The organic was separated, washed with brine, dried over MgSO_4_, filtered, and concentrated under reduced pressure. The crude mixture was purified by reversed-phase column chromatography (Biotage Isolera One, 12g Sfär C18 column, eluted with H_2_O + 0.1% formic acid/MeOH + 0.1% formic acid, 20–80%) to afford 3-((4-isopropylphenyl)­thio)­pyrazine-2-carboxylic acid (50.0 mg, 30%). ^1^H NMR (600 MHz, DMSO-*d*_6_): δ 13.82 (s, 1H), 8.50 (d, *J* = 2.4 Hz, 1H), 8.44 (d, *J* = 2.3 Hz, 1H), 7.45–7.42 (m, 2H), 7.35–7.32 (m, 2H), 2.95 (p, *J* = 6.9 Hz, 1H), 1.24 (d, *J* = 6.9 Hz, 6H). ^13^C NMR (151 MHz, DMSO-*d*_6_): δ 166.4, 150.1, 146.6, 139.8, 136.0, 127.9, 126.7, 33.7, 24.2. HRMS (*m*/*z*): [M + H]^+^ calcd for C_14_H_14_N_2_O_2_S [M + H]^+^, 275.0849; found, 275.0883.

#### 2-((1-Chloronaphthalen-2-yl)­thio)­nicotinic Acid (**22**)

To a solution of 2-sulfanylpyridine-3-carboxylic acid (64.3 mg, 0.414 mmol, 1.0 equiv) and 2-bromo-1-chloro-naphthalene (100 mg, 0.414 mmol, 1.0 equiv) in DMF (1.50 mL) were added K_2_CO_3_ (146 mg, 1.04 mmol, 2.5 equiv) and copper (13.2 mg, 0.207 mmol, 0.5 equiv). The reaction mixture was stirred at 130 °C overnight. After this time, the reaction mixture was cooled to room temperature, filtered through a Si-Thiol cartridge to remove copper, the cartridge was washed with MeOH, and the filtrate was concentrated under reduced pressure. The crude mixture was purified by reversed-phase column chromatography (Biotage Isolera One, liquid load, 12g Sfär C18 column, eluted with H_2_O + 0.1% formic acid/MeOH + 0.1% formic acid, 20–100%) to afford 2-((1-chloronaphthalen-2-yl)­thio)­nicotinic acid (25.0 mg, 18%) as a pale orange solid. ^1^H NMR (600 MHz, DMSO-*d*_6_): δ 13.65 (s, 1H), 8.34 (dd, *J* = 4.7, 1.8 Hz, 1H), 8.28 (ddd, *J* = 9.5, 8.0, 1.6 Hz, 2H), 8.07 (dd, *J* = 7.9, 1.5 Hz, 1H), 7.97 (d, *J* = 8.5 Hz, 1H), 7.76–7.67 (m, 3H), 7.25 (dd, *J* = 7.8, 4.7 Hz, 1H). ^13^C NMR (151 MHz, DMSO): δ 167.0, 160.2, 152.7, 139.7, 137.0, 134.6, 133.9, 131.2, 129.0, 128.9, 128.46, 127.8, 125.0, 120.5. HRMS (*m*/*z*): [M + H]^+^ calcd for C_16_H_10_ClNO_2_S, 316.0194; found, 316.0210.

#### 2-((6-(Methylsulfonamido)­naphthalen-2-yl)­thio)­nicotinic Acid (**23**)

To a solution of 2-sulfanylpyridine-3-carboxylic acid (70.0 mg, 0.451 mmol, 1.0 equiv) and N-(6-bromonaphthalen-2-yl)­methanesulfonamide (135 mg, 0.451 mmol, 1.0 equiv) in DMF (2.00 mL) were added K_2_CO_3_ (117 mg, 0.833 mmol, 1.8 equiv) and copper (13.2 mg, 0.208 mmol, 0.5 equiv). The reaction mixture was stirred at 125 °C overnight. After this time, the resulting mixture was filtered through a Si-Thiol cartridge to remove copper, the cartridge was washed with MeOH, and the filtrate was concentrated under reduced pressure. The crude mixture was purified by reversed-phase column chromatography (Biotage Isolera One12 g Sfär C18 column, eluted with H_2_O + 0.1% formic acid/MeOH + 0.1% formic acid, 20–80%) to afford 2-((6-(methylsulfonamido)­naphthalen-2-yl)­thio)­nicotinic acid (47.0 mg, 26%). ^1^H NMR (600 MHz, DMSO-*d*_6_): δ 13.57 (s, 1H), 10.07 (s, 1H), 8.34 (dd, *J* = 4.7, 1.9 Hz, 1H), 8.24 (dd, *J* = 7.8, 1.9 Hz, 1H), 8.06 (d, *J* = 1.8 Hz, 1H), 7.92 (d, *J* = 8.8 Hz, 1H), 7.86 (d, *J* = 8.6 Hz, 1H), 7.72 (d, *J* = 2.2 Hz, 1H), 7.47 (dd, *J* = 8.6, 1.8 Hz, 1H), 7.41 (dd, *J* = 8.8, 2.2 Hz, 1H), 7.22 (dd, *J* = 7.8, 4.7 Hz, 1H), 3.09 (s, 3H). ^13^C NMR (151 MHz, DMSO-*d*_6_): δ 166.9, 161.5, 152.5, 139.5, 137.6, 134.9, 134.1, 133.8, 130.7, 129.6, 128.1, 127.6, 123.8, 121.0, 120.3, 115.3. HRMS (*m*/*z*): [M + H]^+^ calcd for C_17_H_14_N_2_O_4_S_2_, 375.0468; found, 375.0488.

#### 2-(Phenethylthio)­benzoic Acid (**25**)

To a solution of 2-fluorobenzoic acid (500 mg, 3.57 mmol, 1.0 equiv) and Cs_2_CO_3_ (2.34 g, 7.14 mmol, 2.0 equiv) in DMSO (25.0 mL) was added 2-phenylethanethiol (0.720 mL, 5.35 mmol, 1.5 equiv). The reaction mixture was stirred at 80 °C for 15 h. After cooling to room temperature, the reaction mixture was quenched with 1 M HCl aqueous solution (15 mL), then extracted with EtOAc (3 × 15 mL). The combined organic layers were dried over MgSO_4_, filtered, and concentrated under reduced pressure. The residue was purified by silica gel column chromatography (eluted with cyclohexane/EtOAc, 0–50% EtOAc) to afford 2-(phenethylthio)­benzoic acid (47.2 mg, 5%). ^1^H NMR (500 MHz, CDCl3): δ 8.07 (dd, *J* = 7.9, 1.6 Hz, 1H), 7.42 (ddd, *J* = 8.2, 7.3, 1.6 Hz, 1H), 7.31 (dd, *J* = 8.2, 1.1 Hz, 1H), 7.28–7.22 (m, 2H), 7.21–7.12 (m, 4H), 3.16–3.09 (m, 2H), 2.95 (dd, *J* = 9.4, 6.7 Hz, 2H). ^13^C NMR (126 MHz, CDCl3): δ 171.5, 142.7, 140.3, 133.3, 132.7, 128.8, 128.6, 126.7, 126.6, 125.8, 124.2, 34.8, 33.8. LCMS (*m*/*z*): [M – H]^+^, 257.0661.

#### 2-((4-(Ethylamino)­phenethyl)­thio)­nicotinic Acid Hydrochloride (**29**)

2-((4-((*tert*-Butoxycarbonyl)­(ethyl)­amino)­phenethyl)­thio)­nicotinic acid (75.0 mg, 0.177 mmol, 1.0 equiv) was dissolved in 4 M HCl in dioxane (1.87 mL, 7.48 mmol, 42 equiv). The reaction mixture was stirred at room temperature for 15 h. After this time, the resulting mixture was concentrated under reduced pressure to afford 2-((4-(ethylamino)­phenethyl)­thio)­nicotinic acid hydrochloride (55.0 mg, 98%) as an orange oil. ^1^H NMR (500 MHz, DMSO-*d*_6_): δ 11.19 (s, 1H), 8.65 (dd, *J* = 4.7, 1.9 Hz, 1H), 8.20 (dd, *J* = 7.7, 1.9 Hz, 1H), 7.49 (d, *J* = 8.5 Hz, 2H), 7.45 (d, *J* = 8.5 Hz, 2H), 7.24 (dd, *J* = 7.7, 4.7 Hz, 1H), 3.38–3.34 (m, 2H), 3.29 (q, *J* = 7.2 Hz, 2H), 2.99–2.94 (m, 2H), 1.24 (t, *J* = 7.2 Hz, 3H). ^13^C NMR (151 MHz, DMSO): δ 166.8, 161.0, 152.5, 142.1, 139.5, 134.6, 130.3, 124.2, 123.6, 119.3, 46.5, 34.6, 30.8, 11.3. HRMS (*m*/*z*): [M + H]^+^ calcd for C_16_H_18_N_2_O_2_S, 303.1162; found, 303.1159.

#### 2-((4-((2-Methoxyethyl)­amino)­phenethyl)­thio)­nicotinic Acid Hydrochloride (**30**)

2-((4-((*tert*-Butoxycarbonyl)­(2-methoxyethyl)­amino)­phenethyl)­thio)­nicotinic acid (70.0 mg, 0.162 mmol, 1.0 equiv) was dissolved in 4 M HCl in dioxane (1.71 mL, 6.86 mmol, 42 equiv) and the mixture was stirred at room temperature for 15 h. The reaction mixture was concentrated affording 2-((4-((2-methoxyethyl)­amino)­phenethyl)­thio)­nicotinic acid hydrochloride (60.0 mg, 95%) as a white solid. ^1^H NMR (600 MHz, DMSO-*d*_6_): δ 8.65 (dd, *J* = 4.7, 1.8 Hz, 1H), 8.20 (dd, *J* = 7.7, 1.8 Hz, 1H), 7.41 (s, 4H), 7.24 (dd, *J* = 7.7, 4.7 Hz, 1H), 3.60 (t, *J* = 5.2 Hz, 2H), 3.43 (t, *J* = 5.1 Hz, 2H), 3.38–3.33 (m, 2H), 3.31 (s, 3H), 2.98–2.93 (m, 2H). ^13^C NMR (151 MHz, DMSO): δ 166.8, 161.0, 152.5, 141.9, 139.5, 136.2, 130.2, 124.2, 122.8, 119.3, 67.3, 58.6, 34.5, 30.9. HRMS (*m*/*z*): [M + H]^+^ calcd for C_17_H_21_N_2_O_3_S, 333.1267; found, 333.1258.

#### 2-((4-((2-Fluoroethyl)­amino)­phenethyl)­thio)­nicotinic Acid Hydrochloride (**31**)

2-((4-((*tert*-Butoxycarbonyl)­(2-fluoroethyl)­amino)­phenethyl)­thio)­nicotinic acid (40.0 mg, 0.095 mmol, 1.0 equiv) was dissolved in 4 M HCl in dioxane (1.01 mL, 4.03 mmol, 42 equiv). The reaction mixture was stirred at room temperature for 15 h. After this time, the mixture was concentrated affording 2-((4-((2-fluoroethyl)­amino)­phenethyl)­thio)­nicotinic acid hydrochloride (34.0 mg, 95%) as a white solid. ^1^H NMR (600 MHz, DMSO-*d*_6_): δ 8.65 (dd, *J* = 4.7, 1.8 Hz, 1H), 8.20 (dd, *J* = 7.7, 1.8 Hz, 1H), 7.30 (d, *J* = 7.6 Hz, 2H), 7.23 (dd, *J* = 7.7, 4.7 Hz, 1H), 7.18 (br s, 2H), 4.73 (t, *J* = 4.6 Hz, 1H), 4.65 (t, *J* = 4.6 Hz, 1H), 3.59–3.55 (m, 1H), 3.52 (t, *J* = 4.3 Hz, 1H), 3.35–3.30 (m, 2H), 2.89 (t, *J* = 7.6 Hz, 2H). ^13^C NMR (151 MHz, DMSO): δ 166.8, 161.1, 152.5, 139.5, 130.1, 124.2, 119.3, 80.1, 66.8, 48.5, 34.5, 31.1 (C–H peak ortho to amine and 2 quaternary carbon peaks of the phenyl ring not observed). HRMS (*m*/*z*): [M + H]^+^ calcd for C_16_H_17_FN_2_O_2_S, 321.1068; found, 321.1050.

#### 2-((4-(Dimethylamino)­phenethyl)­thio)­nicotinic Acid (**32**)

2-Mercaptonicotinic acid (70.0 mg, 0.451 mmol, 1.0 equiv), 4-(dimethylamino)­phenethyl 4-methyl-benzenesulfonate (144 mg, 0.451 mmol, 1.0 equiv), and K_2_CO_3_ (69.6 mg, 0.496 mmol, 1.1 equiv) were combined in EtOH (1.40 mL, 0.32 M). The reaction mixture was stirred at 80 °C for 15 h. After this time, the reaction mixture was quenched by the addition of 1 M HCl to acidify to pH 5, extracted with EtOAc (2 × 20 mL), washed with water (25 mL), dried over MgSO_4_, filtered, and concentrated under reduced pressure. The crude product was purified by reversed-phase column chromatography (Biotage Isolera One, 12 g Sfär C18 column, eluted with H_2_O + 0.1% formic acid/MeOH + 0.1% formic acid, 10–100%) to afford 2-((4-(dimethylamino)-phenethyl)-thio)-nicotinic acid (35.0 mg, 23%) as a pale-yellow solid. ^1^H NMR (500 MHz, DMSO-*d*_6_): δ 13.38 (s, 1H), 8.64 (dd, *J* = 4.7, 1.9 Hz, 1H), 8.19 (dd, *J* = 7.7, 1.9 Hz, 1H), 7.22 (dd, *J* = 7.7, 4.7 Hz, 1H), 7.09 (d, *J* = 8.7 Hz, 2H), 6.67 (d, *J* = 8.7 Hz, 2H), 3.30–3.24 (m, 2H), 2.85 (s, 6H), 2.82–2.77 (m, 2H). ^13^C NMR (151 MHz, DMSO): δ 166.9, 161.4, 152.4, 149.6, 139.4, 129.4, 128.8, 124.3, 119.2, 113.1, 40.9, 34.2, 31.7. HRMS (*m*/*z*): [M + H]^+^ calcd for C_16_H_18_N_2_O_2_S, 303.1162; found, 303.1162.

#### 2-((4-(Cyclopentylamino)­phenethyl)­thio)­nicotinic Acid Hydrochloride (**33**)

2-((4-((*tert*-Butoxycarbonyl)­(cyclopentyl)­amino)­phenethyl)­thio)­nicotinic acid (70.0 mg, 0.158 mmol, 1.0 equiv) was dissolved in 4 M HCl in dioxane (1.68 mL, 6.70 mmol, 42 equiv). The reaction mixture was stirred at room temperature for 15 h. After this time, the reaction mixture was concentrated affording 2-[2-[4-(cyclopentylamino)­phenyl]­ethylsulfanyl]­pyridine-3-carboxylic acid hydrochloride (55.0 mg, 87%) as a white solid. ^1^H NMR (500 MHz, DMSO-*d*_6_): δ 13.32 (s, 1H), 10.97 (s, 2H), 8.65 (dd, *J* = 4.7, 1.9 Hz, 1H), 8.21 (dd, *J* = 7.7, 1.9 Hz, 1H), 7.40 (s, 4H), 7.24 (dd, *J* = 7.7, 4.7 Hz, 1H), 3.88–3.82 (m, 1H), 3.38–3.32 (m, 2H), 2.95 (t, *J* = 7.6 Hz, 2H), 1.91–1.83 (m, 2H), 1.78–1.65 (m, 4H), 1.57–1.51 (m, 2H). ^13^C NMR (151 MHz, DMSO): δ 166.8, 161.0, 152.5, 139.5, 134.8, 130.3, 124.1, 123.5, 119.3, 62.6, 34.6, 30.8, 29.7, 23.9. HRMS (*m*/*z*): [M + H]^+^ calcd for C_19_H_22_N_2_O_2_S, 343.1475; found, 343.1459.

#### 2-[2-(4-Isopropylamino-phenyl)-ethylsulfanyl]-nicotinic Acid (**34**)

To a stirred solution of 2-[[2-(4-aminophenyl)­ethyl]­sulfanyl]­pyridine-3-carboxylic acid (100 mg, 0.270 mmol, 1.0 equiv), NaBH­(OAc)_3_ (120 mg, 0.539 mmol, 2.0 equiv), and AcOH (68.2 mg, 1.08 mmol, 4.0 equiv) in CH_2_Cl_2_ (6.00 mL) was added propan-2-one (33.0 mg, 0.539 mmol, 2.0 equiv) dropwise at 0 °C. The mixture was allowed to warm to room temperature and was stirred for 2 h. After this time, the reaction mixture was quenched with water, extracted with CH_2_Cl_2_ (3 × 100 mL), and the organic layer was separated and concentrated under reduced pressure. The crude product was purified by Prep-HPLC (XSelect CSH C18 OBD Prep Column, eluted with H_2_O + 0.05% TFA/ACN, 15–35% ACN) to afford 2-[(2-[4-[(propan-2-yl)­amino]­phenyl]­ethyl)-sulfanyl]­pyridine-3-carboxylic acid (34.0 mg, 39%) as a yellow solid. ^1^H NMR (400 MHz, DMSO-*d*_6_): δ 8.69–8.63 (m, 1H), 8.25–8.18 (m, 1H), 7.40 (d, *J* = 8.0 Hz, 2H), 7.29–7.21 (m, 3H), 3.72–3.59 (m, 1H), 3.40–3.32 (m, 2H), 2.99–2.91 (m, 2H), 1.21 (d, *J* = 6.4 Hz, 6H). HRMS (*m*/*z*): [M + H]^+^ calcd for C_17_H_20_N_2_O_2_S, 317.1; found, 317.1.

#### 2-((4-Isopropoxyphenethyl)­thio)­nicotinic Acid (**35**)

To a 100 mL round-bottom flask were added 4-isopropoxyphenethyl 4-methylbenzene-sulfonate (200 mg, 0.157 mmol, 1.0 equiv), 2-sulfanylpyridine-3-carboxylic acid (38.4 mg, 0.235 mmol, 1.5 equiv), K_2_CO_3_ (45.6 mg, 0.313 mmol, 2.0 equiv), and EtOH (50 mL). The resulting mixture was stirred overnight at 80 °C. The reaction mixture was concentrated under reduced pressure, then diluted with water (50 mL), and acidified to pH 3 with aqueous HCl solution. The aqueous layer was extracted with CH_2_Cl_2_ (3 × 100 mL), the resulting mixture was concentrated under reduced pressure. The crude product was purified by Prep-HPLC (XBridge Prep OBD C18 Column, eluted NH_4_HCO_3_ (5 mM in H_2_O)/ACN, 13–43% ACN) to afford 2-((4-isopropoxyphenethyl)­thio)­nicotinic acid (20.0 mg, 40%) as a white solid. ^1^H NMR (400 MHz, DMSO-*d*_6_): δ 8.59–8.52 (m, 1H), 8.17–8.10 (m, 1H), 7.23–7.08 (m, 3H), 6.88–6.80 (m, 2H), 4.63–4.49 (m, 1H), 3.29–3.21 (m, 2H), 2.86–2.78 (m, 2H), 1.25 (d, *J* = 6.0 Hz, 6H). HRMS (*m*/*z*): [M + 23]^+^ calcd for C_17_H_19_NO_3_S, 318.1; found, 318.1.

#### 2-((2-Phenylpropyl)­thio)­nicotinic Acid (**36**)

2-Mercaptonicotinic acid (70.0 mg, 0.451 mmol, 1.0 equiv), (1-bromopropan-2-yl)­benzene (89.8 mg, 0.451 mmol, 1.0 equiv), and K_2_CO_3_ (69.6 mg, 0.496 mmol, 1.1 equiv) were combined in EtOH (1.40 mL, 0.32 M), heated to 80 °C and stirred for 15 h. The reaction mixture was cooled to room temperature, acidified to pH 3 with the addition of aqueous 1 M HCl and extracted with EtOAc (2 × 20 mL). The combined organic layers were washed with water (25 mL), dried over MgSO_4_, filtered, and concentrated under reduced pressure. The crude product was purified by silica gel column chromatography (10 g Sfär silica column, CH_2_Cl_2_/EtOH, 0–10% EtOH) to afford 2-((2-phenylpropyl)­thio)­nicotinic acid (56.0 mg, 43%) as a pale-yellow solid. ^1^H NMR (600 MHz, DMSO-*d*_6_): δ 13.36 (s, 1H), 8.62 (dd, *J* = 4.7, 1.7 Hz, 1H), 8.18 (dd, *J* = 7.7, 1.8 Hz, 1H), 7.32–7.28 (m, 4H), 7.23–7.19 (m, 2H), 3.40 (dd, *J* = 13.1, 7.2 Hz, 1H), 3.32–3.29 (m, 1H), 3.03 (h, *J* = 7.0 Hz, 1H), 1.32 (d, *J* = 7.0 Hz, 3H). ^13^C NMR (151 MHz, DMSO): δ 166.8, 161.3, 152.3, 146.4, 139.4, 128.8, 127.4, 126.7, 124.2, 119.2, 39.1, 37.5, 22.1. HRMS (*m*/*z*): [M + H]^+^ calcd for C_15_H_15_NO_2_S, 274.0896; found, 274.0906.

#### 2-((1-Phenylpropan-2-yl)­thio)­nicotinic Acid (**37**)

To a 250 mL round-bottom flask were added (2-bromopropyl)­benzene (1.50 g, 7.16 mmol, 1.0 equiv), 2-mercaptonicotinic acid (1.75 g, 10.7 mmol, 1.5 equiv), K_2_CO_3_ (2.08 g, 14.3 mmol, 2.0 equiv), and DMSO (50 mL). The resulting mixture was stirred at 80 °C for 30 min. The reaction was quenched by the addition of water (100 mL) at 0 °C. The mixture was acidified to pH 2 with the addition of aqueous HCl and the aqueous layer was extracted with CH_2_Cl_2_ (3 × 200 mL). The combined organic layers were concentrated under reduced pressure. The residue was purified by silica gel column chromatography (eluted with PE/EtOAc, 1:1) to afford 2-((1-phenylpropan-2-yl)­thio)­nicotinic acid (720 mg, 37%) as a white solid. ^1^H NMR (300 MHz, DMSO-*d*_6_): δ 8.72–8.63 (m, 1H), 8.25–8.16 (m, 1H), 7.31 (d, *J* = 5.3 Hz, 4H), 7.32–7.16 (m, 2H), 4.24–4.09 (m, 1H), 3.14–3.01 (m, 1H), 2.80–2.66 (m, 1H), 1.25 (d, *J* = 6.8 Hz, 3H). HRMS (*m*/*z*): [M + H]^+^ calcd for C_15_H_15_NO_2_S, 274.1; found, 274.1.

#### 2-((1,2-Diphenylethyl)­thio)­nicotinic Acid (**38**)

A mixture of 2-diphenylethyl 4-methylbenzenesulfonate (3.00 g, 2.55 mmol, 1.0 equiv), 2-mercaptonicotinic acid (1.98 g, 12.1 mmol, 4.7 equiv), and K_2_CO_3_ (2.94 g, 20.2 mmol, 7.9 equiv) in EtOH (30.0 mL) was stirred at 80 °C for 16 h. The reaction mixture was concentrated under reduced pressure. The crude product was purified by Prep-HPLC (XBridge Prep OBD C18 Column, H_2_O (10 mmol/L NH_4_HCO_3_ + 0.1% NH_3_ in H_2_O)/ACN, 8–30% ACN) to afford 2-((1,2-diphenylethyl)­thio)­nicotinic acid (100 mg, 12%) as a white solid. ^1^H NMR (400 MHz, Methanol-d4, ppm): δ 8.63–8.57 (m, 1 H), 8.21–8.14 (m, 1 H), 7.36–7.29 (m, 2 H), 7.27–7.19 (m, 2 H), 7.19–7.05 (m, 7 H), 5.37–5.29 (m, 1 H), 3.50–3.40 (m, 1 H), 3.22–3.12 (m, 1 H). HRMS (*m*/*z*): [M + H]^+^ calcd for C_20_H_17_NO_2_S, 336.0; found, 336.0.

#### 2-((1-Amino-1-oxo-3-phenylpropan-2-yl)­thio)­nicotinic Acid (**39**)

To a solution of 2-bromo-3-phenyl-propanamide (162 mg, 0.709 mmol, 1.1 equiv) and 2-mercaptonicotinic acid (100 mg, 0.645 mmol, 1.0 equiv) in acetone (5.00 mL) was added K_2_CO_3_ (180 mg, 1.29 mmol, 2.0 equiv). The reaction mixture was refluxed for 5 h. After this time, the reaction mixture was quenched with aqueous 1 M HCl (50 mL) and extracted with EtOAc (3 × 50 mL). The combined organic layers were dried over MgSO_4_, filtered, and concentrated under reduced pressure. The residue was purified by silica gel chromatography (eluted with cyclohexane/EtOAc, 0–50% EtOAc) to afford 2-((1-amino-1-oxo-3-phenylpropan-2-yl)­thio)­nicotinic acid (4.90 mg, 2%). ^1^H NMR (600 MHz, CDCl_3_): δ 8.58 (dd, *J* = 4.8, 1.8 Hz, 1H), 8.32 (dd, *J* = 7.8, 1.9 Hz, 1H), 7.35–7.30 (m, 4H), 7.25 (tt, *J* = 5.5, 3.2 Hz, 1H), 7.13 (dd, *J* = 7.8, 4.7 Hz, 1H), 4.95 (dd, *J* = 8.6, 6.8 Hz, 1H), 3.34 (dd, *J* = 13.9, 8.6 Hz, 1H), 3.22 (dd, *J* = 13.8, 6.8 Hz, 1H). ^13^C NMR (151 MHz, CDCl_3_): δ 172.7, 168.4, 161.3, 152.4, 139.9, 138.0, 129.1, 128.4, 126.8, 121.9, 118.9, 48.1, 37.9. Not ionizing in HRMS.

#### 2-((1,3-Diphenylpropan-2-yl)­thio)­nicotinic Acid (**40**)

In a 50 mL sealed tube, a mixture of 2-mercaptonicotinic acid (351 mg, 2.20 mmol, 1.5 equiv), *N*’-(1,3-diphenylpropan-2-ylidene)-4-methylbenzenesulfonohydrazide (572 mg, 1.47 mmol, 1.0 equiv), K_2_CO_3_ (528 mg, 3.63 mmol, 2.5 equiv), and EtOH (23.0 mL) was stirred at 80 °C under a nitrogen atmosphere for 16 h. After this time, the reaction mixture was filtered and the filter cake was washed with EtOH (2 × 5 mL). The filtrate was concentrated under reduced pressure. The residue was purified by Prep-HPLC (XBridge Prep OBD C18 Column, H_2_O (10 mmol/L NH_4_HCO_3_ + 0.1% NH_3_ in H_2_O)/ACN, 20–50% ACN) to afford 2-((1,3-diphenylpropan-2-yl)­thio)­nicotinic acid (6.00 mg, 0.1%) as a white solid. ^1^H NMR (400 MHz, DMSO-*d*_6_): δ 8.59–8.54 (m, 1H), 8.10–8.03 (m, 1H), 7.37–7.21 (m, 8H), 7.21–7.08 (m, 3H), 4.40–4.32 (m, 1H), 3.02–2.74 (m, 4H). HRMS (*m*/*z*): [M + H]^+^ calcd for C_21_H_19_NO_2_S, 350.1; found, 350.1.

#### 2-((1-Benzylcyclopentyl)­thio)­nicotinic Acid (**41**)

To a 30 mL sealed tube were added 1-benzylcyclopentan-1-ol (100 mg, 0.539 mmol, 1.0 equiv), 2-mercaptonicotinic acid (100 mg, 0.612 mmol, 1.1 equiv), and TFA (3.00 mL, 26.3 mmol, 75 equiv). The resulting mixture was stirred at 80 °C for 30 min. The reaction was quenched by the addition of water/ice (10 mL) at 0 °C, then NaHCO_3_ was added to reach pH 7. The aqueous layer was extracted with CH_2_Cl_2_ (3 × 100 mL), the combined organic layers were concentrated under reduced pressure. The crude product was purified by Prep-HPLC (Column: XBridge Shield RP18 OBD Column, eluted with H_2_O (10 mmol/L NH_4_HCO_3_ + 0.1% NH_3_ in H_2_O)/ACN, 25–35% ACN) to afford 2-((1-benzylcyclopentyl)­thio)­nicotinic acid (3.50 mg, 2%) as a white solid. ^1^H NMR (300 MHz, DMSO-*d*_6_, ppm): δ 8.50 (s, 1H), 7.99 (s, 1H), 7.22–7.03 (m, 6H), 3.44 (s, 2H), 1.96–1.84 (m, 4H), 1.71–1.59 (m, 4H). HRMS (*m*/*z*): [M + H]^+^ calcd for C_18_H_19_NO_2_S, 314.1; found, 314.2.

#### (*R*)-2-((1-Phenylpropan-2-yl)­thio)­nicotinic Acid (**42**)

**37** 2-((1-Phenylpropan-2-yl)­thio)­nicotinic acid (500 mg, 1.80 mmol) was purified by Chiral-HPLC (Chiralpak AD-H, 2 × 25 cm­(5 μm), Hex (0.1% formic acid)/EtOH; 0–5% EtOH) to afford (*R*)-2-((1-phenylpropan-2-yl)-thio)­nicotinic acid (105 mg, 18%) as a white solid. ^1^H NMR (400 MHz, DMSO-*d*_6_): δ 13.39 (br s, 1H), 8.69–8.63 (m, 1H), 8.21–8.13 (m, 1H), 7.35–7.18 (m, 6H), 4.23–4.10 (m, 1H), 3.11–3.02 (m, 1H), 2.77–2.67 (m, 1H), 1.24 (d, *J* = 6.8 Hz, 3H). HRMS (*m*/*z*): [M + H]^+^ calcd for C_15_H_15_NO_2_S, 274.1; found, 274.1.

#### (*S*)-2-((1-Phenylpropan-2-yl)­thio)­nicotinic Acid (**43**)

**37** 2-((1-Phenylpropan-2-yl)­thio)­nicotinic acid (500 mg, 1.80 mmol) was purified by Chiral-HPLC (Chiralpak AD-H, Hex (0.1%FA)/EtOH; 0–5% EtOH) to afford (*S*)-2-((1-phenylpropan-2-yl)­thio)­nicotinic acid (104 mg, 18%) as a white solid. ^1^H NMR (400 MHz, DMSO-*d*_6_): δ 13.30 (br s, 1H), 8.67–8.61 (m, 1H), 8.19–8.12 (m, 1H), 7.28 (d, *J* = 5.9 Hz, 4H), 7.24–7.14 (m, 2H), 4.21–4.07 (m, 1H), 3.09–2.99 (m, 1H), 2.74–2.64 (m, 1H), 1.22 (d, *J* = 6.9 Hz, 3H). HRMS (*m*/*z*): [M + H]^+^ calcd for C_15_H_15_NO_2_S, 274.1; found, 274.1.

#### (*S*)-2-((1,2-Diphenylethyl)­thio)­nicotinic Acid (**44**)

**38** 2-((1,2-Diphenylethyl)­thio)­nicotinic acid (100 mg, 0.295 mmol) was purified by Chiral-HPLC (N-Column: N-Reg-AD Column, Hex (0.1% formic acid)/EtOH, 93:7) to afford (*S*)-2-((1,2-diphenylethyl)­thio)-nicotinic acid (27.8 mg, 27%) as a white solid. ^1^H NMR (400 MHz, DMSO-*d*_6_): δ 13.39 (s, 1 H), 8.74–8.67 (m, 1 H), 8.23–8.16 (m, 1 H), 7.42–7.35 (m, 2 H), 7.30–7.20 (m, 3 H), 7.23–7.13 (m, 5 H), 7.17–7.07 (m, 1 H), 5.36–5.28 (m, 1 H), 3.43–3.34 (m, 1 H), 3.29–3.18 (m, 1 H). HRMS (*m*/*z*): [M + H]^+^ calcd for C_20_H_17_NO_2_S, 336.1; found, 336.1.

#### (*R*)-2-((1,2-Diphenylethyl)­thio)­nicotinic Acid (**45**)

**38** 2-((1,2-Diphenylethyl)­thio)­nicotinic acid (100 mg, 0.295 mmol) was purified by Chiral-HPLC (N-Column: N-Reg-AD Column, Hex (0.1% formic acid)/EtOH, 93:7) to afford (*R*)-2-((1,2-diphenylethyl)­thio)-nicotinic acid (27.3 mg, 26%) as a white solid. ^1^H NMR (400 MHz, DMSO-*d*_6_): δ 13.39 (s, 1 H), 8.74–8.68 (m, 1 H), 8.23–8.16 (m, 1 H), 7.42–7.35 (m, 2 H), 7.30–7.06 (m, 9 H), 5.36–5.27 (m, 1 H), 3.40–3.36 (s, 1 H), 3.29–3.18 (m, 1 H). HRMS (*m*/*z*): [M + H]^+^ calcd for C_20_H_17_NO_2_S, 336.1; found, 336.1.

#### 2-((1-(4-(Isopropylamino)­phenyl)­butan-2-yl)­thio)­nicotinic Acid (**46**)

To a 10 mL sealed tube were added 2-((1-(4-bromophenyl)­butan-2-yl)­thio)­nicotinic acid (36.0 mg, 0.098 mmol, 1.0 equiv), isopropylamine (13.0 mg, 0.209 mmol, 2.1 equiv), JohnPhos (13.0 mg, 0.041 mmol, 0.4 equiv), Pd_2_(dba)_3_ (10.0 mg, 0.010 mmol, 0.1 equiv), *t*-BuONa (20.0 mg, 0.198 mmol, 2.0 equiv), and dioxane (2.00 mL). The resulting mixture was stirred at 80 °C for 13 h under a nitrogen atmosphere. The reaction mixture was filtered, washed with CH_2_Cl_2_ (4 × 10 mL), and the filtrate was concentrated under reduced pressure. The crude product was purified by prep-HPLC (XBridge Prep OBD C18 Column, eluted with H_2_O (10 mmol/L NH_4_HCO_3_ + 0.1% NH3 in H_2_O)/ACN, 25–50% ACN) to afford 2-((1-(4-(isopropylamino)-phenyl)­butan-2-yl)­thio)­nicotinic acid (10.2 mg, 29%) as a white solid. ^1^H NMR (300 MHz, Methanol-d4): δ 8.44–8.36 (m, 1H), 7.89–7.78 (m, 1H), 7.18–6.94 (m, 3H), 6.70–6.57 (m, 2H), 4.20–4.00 (m, 1H), 3.68–3.47 (m, 1H), 3.07–2.84 (m, 1H), 2.68–2.53 (m, 1H), 1.81–1.60 (m, 1H), 1.60–1.42 (m, 1H), 1.21–1.13 (m, 6H), 1.06–0.92 (m, 3H). HRMS (*m*/*z*): [M + H]^+^ calcd for C_19_H_24_N_2_O_2_S, 345.0; found, 345.0.

#### 2-((1-(4-(Isopropylamino)­phenyl)-4-phenylbutan-2-yl)­thio)­nicotinic Acid (**47**)

To a 10 mL sealed tube were added 2-((1-(4-bromophenyl)-4-phenylbutan-2-yl)­thio)­nicotinic acid (20.0 mg, 0.045 mmol, 1.0 equiv), isopropylamine (6.00 mg, 0.096 mmol, 2.1 equiv), JohnPhos (6.00 mg, 0.019 mmol, 0.4 equiv), Pd_2_(dba)_3_ (9.00 mg, 0.009 mmol, 0.2 equiv), *t*-BuONa (10.0 mg, 0.099 mmol, 2.2 equiv), and dioxane (2.00 mL). The resulting mixture was stirred at 80 °C under a nitrogen atmosphere for 13 h. After this time, the mixture was filtered, washed with CH_2_Cl_2_ (4 × 10 mL), and the filtrate was concentrated under reduced pressure. The crude product was purified by Prep-HPLC (XBridge Shield RP18 OBD Column, eluted H_2_O (10 mmol/L NH_4_HCO_3_ + 0.1% NH_3_ in H_2_O)/ACN, 26–56% ACN) to afford 2-((1-(4-(isopropylamino)­phenyl)-4-phenylbutan-2-yl)­thio)­nicotinic acid (2.00 mg, 11%) as a white solid. ^1^H NMR (500 MHz, DMSO): δ 8.56 (dd, *J* = 4.6, 1.9 Hz, 1H), 8.13 (dd, *J* = 7.6, 1.9 Hz, 1H), 7.22 (t, *J* = 7.5 Hz, 2H), 7.20–7.11 (m, 2H), 7.11–7.05 (m, 2H), 6.97 (d, *J* = 8.3 Hz, 2H), 6.49–6.44 (m, 2H), 5.17 (s, 1H), 4.17–4.08 (m, 1H), 3.48 (p, *J* = 6.3 Hz, 1H), 2.86 (dd, *J* = 13.8, 5.7 Hz, 1H), 2.79 (ddd, *J* = 13.7, 10.6, 5.3 Hz, 1H), 2.71–2.56 (m, 2H), 1.91 (ddt, *J* = 13.7, 10.7, 5.5 Hz, 1H), 1.77 (tdd, *J* = 15.2, 7.8, 5.4 Hz, 1H), 1.10 (d, *J* = 6.2 Hz, 6H). HRMS (*m*/*z*): [M + H]^+^ calcd for C_25_H_28_N_2_O_2_S, 421.0; found, 421.0.

#### 2-((1-(3,5-Dimethylphenyl)-2-(4-(isopropylamino)­phenyl)­ethyl)­thio)­nicotinic Acid (**48**)

To a 30 mL sealed tube were added 2-iodonicotinic acid (108 mg, 0.412 mmol, 1.0 equiv), 1-{[1-(3,5-dimethylphenyl)-2-{4-[(propan-2-yl)­amino]­phenyl}­ethyl]­sulfanyl}­ethan-1-one (180 mg, 0.522 mmol, 1.3 equiv), Pd_2_(dba)_3_ (40.0 mg, 0.041 mmol, 0.1 equiv), XantPhos (50.0 mg, 0.082 mmol, 0.2 equiv), Cs_2_CO_3_ (280 mg, 0.816 mmol, 2.0 equiv), and dioxane (15.0 mL). The resulting mixture was stirred at 120 °C for 13 h under a nitrogen atmosphere. After this time, the reaction mixture was cooled to room temperature and filtered, the filter cake was washed with CH_2_Cl_2_ (4 × 10 mL) and the filtrate was concentrated under reduced pressure. The crude product was purified by Prep-HPLC (XBridge Prep OBD C18 Column, H_2_O (10 mol/L NH_4_HCO_3_)/ACN, 24–51% ACN) to afford 2-((1-(3,5-dimethylphenyl)-2-(4-(isopropylamino)­phenyl)­ethyl)­thio)­nicotinic acid (11.0 mg, 6%) as a white solid. ^1^H NMR (300 MHz, DMSO-*d*_6_): δ 8.56–8.49 (m, 1H), 8.08–8.01 (m, 1H), 7.13–7.04 (m, 1H), 6.95–6.74 (m, 5H), 6.42–6.29 (m, 2H), 5.12–5.01 (m, 1H), 3.47–3.37 (m, 1H), 3.18–3.04 (m, 1H), 3.04–2.90 (m, 1H), 2.19 (s, 6H), 1.11–0.97 (m, 6H). HRMS (*m*/*z*): [M + H]^+^ calcd for C_25_H_28_N_2_O_2_S, 421.0; found, 421.0.

#### 2-((1-(3,5-Dimethoxyphenyl)-2-(4-(isopropylamino)­phenyl)­ethyl)­thio)­nicotinic Acid (**49**)

A mixture of 1-{[1-(3,5-dimethoxyphenyl)-2-{4-[(propan-2-yl)­amino]­phenyl}­ethyl]­sulfanyl}­ethan-1-one (90.0 mg, 0.229 mmol, 1.3 equiv), 2-iodonicotinic acid (45.0 mg, 0.172 mmol, 1.0 equiv), Pd_2_(dba)_3_ (17.0 mg, 0.018 mmol, 0.1 equiv), XantPhos (21.0 mg, 0.034 mmol, 0.2 equiv), and Cs_2_CO_3_ (120 mg, 0.350 mmol, 2.0 equiv) in 1,4-dioxane (2.00 mL) was stirred at 120 °C for 16 h under a nitrogen atmosphere. The reaction mixture was cooled to room temperature, diluted with CH_2_Cl_2_ (20 mL), then concentrated under reduced pressure. The crude product was purified by Prep-HPLC (XSelect, CSH OBD Column, eluted with H_2_O (10 mmol/L NH_4_HCO_3_ + 0.1% NH_3_ in H_2_O)/ACN, 13–38% ACN) to afford 2-((1-(3,5-dimethoxyphenyl)-2-(4-(isopropylamino)­phenyl)­ethyl)­thio)-nicotinic acid (29.2 mg, 37%) as a white solid. ^1^H NMR (400 MHz, DMSO-*d*_6_): δ 8.68–8.62 (m, 1 H), 8.18–8.11 (m, 1 H), 7.23–7.16 (m, 1 H), 6.91–6.85 (m, 2 H), 6.51 (d, *J* = 2.3 Hz, 2 H), 6.42–6.34 (m, 2 H), 6.33–6.28 (m, 1 H), 5.19–5.11 (m, 1 H), 3.69 (s, 6 H), 3.49–3.38 (m, 1 H), 3.19–3.09 (m, 1 H), 3.09–2.98 (m, 1 H), 1.07 (d, *J* = 6.3 Hz, 6 H). HRMS (*m*/*z*): [M + H]^+^ calcd for C_25_H_28_N_2_O_4_S, 453.1; found, 453.1.

#### 2-[[(2*R*)-1-(4-(Isopropylamino)-phenyl)­propan-2-yl]­thio]­nicotinic Acid (**50**)

To a stirred solution of 2-((1-(4-aminophenyl)­propan-2-yl)­thio)­nicotinic acid (300 mg, 1.041 mmol, 1.0 equiv), AcOH (184 mg, 3.07 mmol, 2.9 equiv), and NaBH­(AcO)_3_ (324 mg, 1.53 mmol, 1.5 equiv) in CH_2_Cl_2_ (30 mL) was added acetone (89.0 mg, 1.53 mmol, 1.5 equiv) dropwise at 0 °C and the resulting mixture was stirred at room temperature for 1 h. After this time, the reaction was quenched with water, and the mixture was extracted with CH_2_Cl_2_ (4 × 60 mL). The combined organic layers were dried over anhydrous Na_2_SO_4_, filtered, and concentrated under reduced pressure. The residue was purified by reversed-phase flash chromatography (C18 silica gel, eluted with NH_4_HCO_3_ (5 mM in H_2_O)/ACN, 10–50% ACN) to afford 2-((1-(4-(isopropylamino)-phenyl)­propan-2-yl)­thio)­nicotinic acid (220 mg, 64%) as a white solid. HRMS (*m*/*z*): [M + H]^+^ calcd for C_18_H_22_N_2_O_2_S, 331.0; found, 331.0. The racemic mixture 2-((1-(4-(Isopropylamino)­phenyl)­propan-2-yl)­thio)­nicotinic acid (220 mg, 0.666 mmol) was purified by Chiral-HPLC (Column: Chiralpak AD-H, 2 × 25 cm (5 μm); eluted with Hex (0.1% formic acid)/EtOH; Gradient: 8% EtOH in 13 min; RT1:8.36; RT2:11.368) to give 2-[[(2*R*)-1-(4-(isopropylamino)-phenyl)­propan-2-yl]­thio]­nicotinic acid (20.0 mg, 9%) as a white solid. ^1^H NMR (300 MHz, DMSO-*d*_6_): δ 8.67–8.58 (m, 1H), 8.24–8.10 (m, 1H), 7.28–7.14 (m, 1H), 7.06–6.89 (m, 2H), 6.55–6.41 (m, 2H), 4.09–3.91 (m, 1H), 3.56–3.26 (m, 1H), 2.96–2.77 (m, 1H), 2.54–2.44 (m, 1H), 1.24–1.16 (m, 3H), 1.16–1.05 (m, 6H). HRMS (*m*/*z*): [M + H]^+^ calcd for C_18_H_22_N_2_O_2_S, 331.0; found, 331.0.

#### 2-[[(2*S*)-1-(4-(Isopropylamino)­phenyl)­propan-2-yl]­thio]­nicotinic Acid (**51**)

The racemic mixture 2-((1-(4-(Isopropylamino)­phenyl)­propan-2-yl)­thio)­nicotinic acid (220 mg, 0.666 mmol) was purified by Chiral-HPLC (Column: Chiralpak AD-H, 2 × 25 cm (5 μm); eluted with Hex (0.1% formic acid)/EtOH, Gradient: 8% EtOH in 13 min; RT1:8.36; RT2:11.368) to give 2-[[(2*S*)-1-(4-(isopropylamino)­phenyl)­propan-2-yl]­thio]­nicotinic acid (21.0 mg, 10%) as a white solid. ^1^H NMR (300 MHz, DMSO-*d*_6_): δ 8.65–8.55 (m, 1H), 8.20–8.08 (m, 1H), 7.22–7.10 (m, 1H), 7.00–6.89 (m, 2H), 6.53–6.41 (m, 2H), 4.15–3.91 (m, 1H), 3.60–3.29 (m, 1H), 3.01–2.77 (m, 1H), 2.54–2.44 (m, 1H) 1.23–1.15 (m, 3H), 1.12–1.03 (m, 6H). HRMS (*m*/*z*): [M + H]^+^ calcd for C_18_H_22_N_2_O_2_S, 331.0; found, 331.0.

#### 2-[(*S*)-2-(4-Isopropylamino-phenyl)-1-phenyl-ethylthio]­nicotinic Acid (**52**)

To a 30 mL sealed tube were added 2-((2-(4-aminophenyl)-1-phenylethyl)­thio)­nicotinic acid (100 mg, 0.265 mmol, 1.0 equiv), NaBH­(OAc)_3_ (118 mg, 0.530 mmol, 2.0 equiv), AcOH (67.0 mg, 1.06 mmol, 4.0 equiv), and CH_2_Cl_2_ (6.00 mL). To the above mixture was added acetone (32.4 mg, 0.530 mmol, 2.0 equiv) dropwise at 0 °C under a nitrogen atmosphere and the resulting mixture was stirred at room temperature for 2 h. After this time, the reaction was quenched with water, and the mixture was extracted with CH_2_Cl_2_ (5 × 40 mL). The combined organic extracts were dried over anhydrous Na_2_SO_4_, filtered, and concentrated under reduced pressure. The residue was purified by reversed-phase flash chromatography (C18 silica gel; NH_4_HCO_3_ (5 mM in H_2_O)/ACN, 3–100% gradient) to afford 2-[2-(4-isopropylamino-phenyl)-1-phenyl-ethylthio]­nicotinic acid (38.0 mg, 35%) as a yellow oil. HRMS (*m*/*z*): [M + H]^+^ calcd for C_23_H_24_N_2_O_2_S, 393.0; found, 393.0. The racemic mixture 2-[2-(4-isopropylamino-phenyl)-1-phenyl-ethylthio]­nicotinic acid (38.0 mg, 0.096 mmol) was purified by Chiral HPLC (Column: Chiralpak AD-H, 2 × 25 cm (5 μm); Hex (0.1% formic acid)/IPA; 5% IPA in 26 min; RT1:16.742; RT2:20.816) to give 2-[(*S*)-2-(4-isopropylamino-phenyl)-1-phenyl-ethylthio]­nicotinic acid as the first eluting fraction as a white solid (3.00 mg, 8%). ^1^H NMR (400 MHz, Methanol-d4): δ 8.60–8.54 (m, 1H), 8.17–8.11 (m, 1H), 7.35–7.28 (m, 2H), 7.25–7.17 (m, 2H), 7.17–7.09 (m, 2H), 6.97–6.91 (m, 2H), 6.63–6.55 (m, 2H), 5.33–5.22 (m, 1H), 3.57–3.49 (m, 1H), 3.37–3.30 (m, 1H), 3.11–3.03 (m, 1H), 1.18–1.13 (m, 6H). HRMS (*m*/*z*): [M + H]^+^ calcd for C_23_H_24_N_2_O_2_S, 393.0; found, 393.0.

#### 2-[(*R*)-2-(4-Isopropylamino-phenyl)-1-phenyl-ethylthio]­nicotinic Acid (53)

The racemic mixture 2-[2-(4-isopropylamino-phenyl)-1-phenyl-ethylthio]­nicotinic acid (38.0 mg, 0.096 mmol) was purified by Chiral HPLC (Column: Chiralpak AD-H, 2 × 25 cm (5 μm); Hex (0.1% formic acid)/IPA; 5% IPA in 26 min; RT1:16.742; RT2:20.816) to give 2-[(R)-2-(4-isopropylamino-phenyl)-1-phenyl-ethylthio]­nicotinic acid as the second eluting fraction as a white solid (3 mg, 8%). ^1^H NMR (400 MHz, Methanol-d4): δ 8.59–8.55 (m, 1H), 8.17–8.12 (m, 1H), 7.35–7.28 (m, 2H), 7.25–7.18 (m, 2H), 7.18–7.09 (m, 2H), 6.98–6.90 (m, 2H), 6.65–6.56 (m, 2H), 5.31–5.22 (m, 1H), 3.58–3.49 (m, 1H), 3.37–3.34 (m, 1H), 3.12–3.02 (m, 1H), 1.19–1.10 (m, 6H). HRMS (*m*/*z*): [M + H]^+^ calcd for C_23_H_24_N_2_O_2_S, 393.0; found, 393.0.

## Supplementary Material







## Notes

Docked structures and QM calculations (ZIP)
Molecular formula strings and some data (CSV)
Supplementary experimental details on protein synthesis and purification, biophysical fragment screening and follow-up experiments, biochemical and cellular target engagement assays, computational approaches, crystallographic and cryoEM structured determination methods, and synthetic details of compound intermediates; supplementary figures; and supplementary tables including those listing crystallographic and cryoEM data statistics (PDF)

## Abbreviations

AcOH: acetic acid
aq.: aqueous
CPMG: Carr–Purcell–Meiboom–Gill
cryoEM: cryo-electron microscopy
DEAH-box: aspartate glutamate alanine histidine-box
DExH-box: aspartate glutamate any amino acid histidine-box
DHX8: DEAH-box helicase 8
DHX9: DExH-box helicase 9
DIAD: diisopropyl azodicarboxylate
dsRNA: double-stranded ribonucleic acid
eq: equivalent
ERBB2: erythroblastic oncogene B2
Et_3_N: triethylamine
Et_2_O: diethyl ether
EtOAc: ethyl acetate
EtOH: ethanol
FA: fluorescence anisotropy
fl: full length
Hex: hexane
HRMS: high-resolution mass spectrometry
HSF1: heat shock factor 1
HSP70: heat shock protein 70
HSP90: heat shock protein 90
HT: hook-turn
HTS: high-throughput screen
IPA: isopropyl alcohol
*K*_D_: dissociation constant
LogD_7.4_: logarithm of the distribution coefficient at pH 7.4
LO-NMR: ligand-observed nuclear magnetic resonance
MeOH: methanol
MTAD: minimal transactivation domain
MW: microwave
n.d.: not done
NTP: nucleoside triphosphate
PDFG-B: Platelet-Derived Growth Factor Subunit B
PE: petroleum ether
PK: pharmacokinetics
Prp22: pre-mRNA-splicing factor 22
Prp43: pre-mRNA-splicing factor 43
QC: quality control
RAS: rat sarcoma
RecA1: recombination A1
RecA2: recombination A2
RU: response unit
sat.: saturated
sd: standard deviation
SF3B1: splicing factor 3B subunit 1
siRNA: small interfering ribonucleic acid
S_N_Ar: nucleophilic aromatic substitution
sol.: solution
SPR: surface plasmon resonance
ssRNA: single-stranded ribonucleic acid
STD: saturation transfer difference
*T*_m_: melting temperature
TSA: Thermal shift assay
WaterLOGSY: water ligand observed via gradient spectroscopy
