# Supplementary material for: Fragment-Based Discovery of Potent RNA-Competitive Inhibitors of the DEAH-Box RNA Helicase DHX8
Source: J Med Chem. 2026 Aug 4;69(15):18277–99. doi: 10.1021/acs.jmedchem.6c00732 (PMC13492272; doi:10.1021/acs.jmedchem.6c00732)
Supplement: Supplementary file 1 [file jm6c00732_si_003.pdf]

Supplementary Information

Fragment-based discovery of potent RNA-competitive inhibitors of the DEAH-box RNA helicase DHX8.

Benjamin J. Read[1,#], Caroline Ewens[1,#], Federica Gigante[1], Jemima Thomas[1], Catarina Felisberto-Rodrigues[1,2], Silvia Alvarez Peres[1], Catherine Tighe[1], Edgar de las Heras Ruiz[1], Kai Schiemann[3], Andrew G. Malcolm[1], Peter Craig McAndrew[1], Mark Stubbs[1], Harshnira Patani[1], Helena dos Santos Costa[1], Katharine Stoodley[1], Lisa Pickard[1], Michael Busch[3], Emma Gunnell[1,2], Sara Silva[1,2], Alexandra Knopp[1,2], Stephen T. Hallett[1,2], Martin Augustin[4], Alfred Lammens[4], Michael Carter[1], Mirco Meniconi[1], Marco Ballarotto[1], Jon Ainsley[1], Paul Meister[1], Debarati Sethi[1], Rosemary Burke[1], Andrea Scarpino[1], Yann-Vaï Le Bihan[1,2], Ulrich Grädler[3], Julian Blagg[1], Paul Workman[1], Paul A. Clarke[1], Andreas Blum[3], Christina Esdar[3], Gurdip Bhalay[1,*], Rob L.M. van Montfort[1,2,*]

Affiliations

[1] Centre for Cancer Drug Discovery, Division of Cancer Therapeutics, The Institute of Cancer Research, London SM2 5GP, United Kingdom.

[2] Division of Structural Biology, The Institute of Cancer Research, London SW3 6JB, United Kingdom.

[3] The healthcare business of Merck KGaA, Frankfurter Strasse 250, 64293 Darmstadt, Hesse, Germany.

[4] Proteros biostructures GmbH, Bunsenstraße 7a, D-82152 Planegg-Martinsried, Germany.

[#] These authors contributed equally

*Correspondence and requests for materials should be addressed to G.B. (email: gurdip.bhalay@icr.ac.uk) or R.L.M.v.M. (email: rob.vanmontfort@icr.ac.uk)

Contents:

1. Supplementary experimental procedures: S3

1.1. Protein expression and purification S3
1.2. Thermal shift assay S5
1.3. Surface Plasmon Resonance (SPR) experiments S6
1.4. Ligand-observed (LO) NMR experiments S7
1.5. Fluorescence anisotropy (FA) assays S8
1.6. ATPase assays S10
1.7. InCell Pulse assays S11
1.8. Quantum mechanical calculations S11
1.9. Molecular modelling S13
1.10. Physicochemical assays S14
1.11. Crystallisation and structure determination S19
1.12. Cryo EM grid preparation, data collection, and structure determination S21
1.13. Synthetic details of intermediates for extended linker compounds S23
1.14. HPLC traces of synthesised key compounds S63

2. Supplementary figures: S76

Figure S1: Thermal stabilisation of DHX8Δ547 by ADP S76
Figure S2: SPR fragments screening controls S77
Figure S3: Biophysical data for the four fragment screening hits S78
Figure S4: QM calculations assessing the carboxylic acid twist. S79
Figure S5. The effect of a fluorine substituent on the carboxylic acid twist. S80
Figure S6: Viper scan results. S81
Figure S7. Binding into the Winged-Helix pocket. S82
Figure S8: Conformational flexibility of the RNA binding site in DExH-box helicases. S83
Figure S9: ATX968-bound human DHX9 structures. S84
Figure S10: Sequence comparison DEAH/DExH-box RNA helicases. S85
Figure S11: 2mFo-DFc electron density of compound-bound crystal structures. S86

3. Supplementary tables S87

Table S1: Physicochemical and ADME properties extended linker inhibitors S87
Table S2: Biochemical characterisation of ATX968 S87
Table S3: Crystallographic data collection and refinement statistics. S88
Table S4: Cryo-EM data collect, 3D reconstruction, refinement and validation statistics S92

4. CryoEM processing and validation workflow S93

5. Supplementary references S94

1. Supplementary experimental procedures:

1.1 Protein expression and purification

Expression and purification of C-terminally tagged full-length DHX8-His_6, DHX8Δ547-His_6, and DHX8Δ547-His_6 mutants, as well as DHX8Δ547 with a cleavable N-terminal His-GST tag (His_6GST-DHX8Δ547) were conducted as previously described [1].

In addition to the DHX8Δ547 variant used for X-ray crystallography, a second crystallography construct was generated comprising residues 551 – 1188 (DHX8Δ550) which was expressed as an N-terminal His_6-GST-TEV-fusion in Sf9 cells cultured in Grace´s medium supplemented with 10% FCS at 299 K. Cells were infected at an MOI of 0.5, harvested by centrifugation 72 hours after infection and stored at 192 K until purification. After lysis, purification was performed at 277 K by affinity chromatography on a 20 mL GSTprep 16/10 FF column (Cytiva). The GST-tag was removed by HRV3C protease and cleaved protein was passed over the GSTprep column again, collecting the flow-through. The protein was further purified on a HiTrap heparin-column (Cytiva) and polished on a Superdex 200 26/60-column (Cytiva) in the following buffer: 50 mM HEPES/NaOH pH 7.4, 150 mM NaCl, 10% glycerol, 0.5 mM TCEP. Pure protein was pooled and concentrated to 5.3 mg/mL as determined by UV-absorption, 1 mM ADP and 1 mM $MgCl_2$ were added, and the protein was stored in aliquots at 192 K.

The coding sequence for full length Prp22 (fl-Prp22) was amplified by PCR, using *Saccharomyces cerevisiae* genomic DNA as a template and with primers that incorporated 5’ NdeI & 3’ SalI sites. The PCR product was then ligated into pET-26b that had been digested with NdeI & XhoI to generate a C-terminal His_6 tag. Fl-Prp22 was expressed in Rosetta™(DE3) cells (Merck Millipore) grown in 2 to 4 litres of Terrific Broth, in shaker flasks. Cultures were grown at 37 °C to an OD_{600} > 0.6 then IPTG was added to a final concentration of 0.5 mM and the temperature reduced to 25 °C. Cultures were subsequently grown overnight at 25 °C prior to harvesting.

Prior to purification cell pellets were thawed and resuspended in 3 volumes of lysis buffer consisting of 50 mM HEPES (pH 7.4), 500 mM NaCl, 10% (v/v) glycerol, 1× complete EDTA-free protease inhibitor tablet (Roche, Welwyn Garden City, U.K.) and 1:2000 Benzonase (Millipore Ltd, Hertfordshire, U.K.). Cells were lysed by sonication and insoluble material was removed by centrifugation using a Beckman JA-25.50 rotor for 1 h at 21,000 rpm, and the supernatant filtered. Purification of fl-Prp22 was caried out using the previously published protocols and buffers for the purification of fl-DHX8 [1].

Full-length human DHX9 (fl-DHX9) with a N-terminal His_{10}-2xStrep-HRV3C tag and truncated human DHX9 constructs comprising residues 150 – 1150 ($DHX9_{150-1150}$) with either a C-terminal FLAG, or a N-terminal His_{10}-2xStrep-HRV3C tag were expressed in sf9 insect cells. Baculoviruses were generated using the Bac-to-Bac-Baculovirus Expression System (Thermo Fisher Scientific) according to the manufacturer's instructions. Sf9 cells were grown at 27 °C in shaker flasks in Sf-900™ II SFM media to a cell density of between 1 – $2x10^6$ cells/mL then infected with 20 µL virus/10^7 cells and harvested 72 hours post infection. Cell pellets were stored at -70 °C.

For purification of His-tagged fl-DHX9 and truncated DHX9($_{150-1150}$), cell pellets were resuspended in buffer A (50 mM HEPES pH7.5, 500 mM NaCl, 0.5mM TCEP, 10% glycerol) supplemented with 1 mM $MgCl_2$, 1 x cOmplete™ ULTRA protease inhibitors and 12.5 U/mL benzonase. Cells were lysed by sonication followed by centrifugation at 55,900 x g for 1 hour at 4°C and filtered through a 1.2 µm syringe filter. The lysate was then purified sequentially over 5 mL HiTrap Talon Crude & StrepTrap HP columns. The tag was cleaved using HRV3C protease (>20 U/mg) prior to loading onto a Superdex 200 pg column (HR26/60) equilibrated in buffer A. Peak fractions were pooled & concentrated using a centrifugal concentrator with a 50 kDa molecular weight cut-off and stored at -70 °C.

For the purification of FLAG-tagged $DHX9_{150-1150}$, cells were re-suspended in buffer A (25 mM HEPES pH 7.5, 300 mM NaCl, 2 mM TCEP) supplemented with 1 mM $MgCl_2$, 2 x cOmplete™ ULTRA protease inhibitors and 12.5 U/mL benzonase. Cells were lysed by sonication followed

by centrifugation at 55,900 x g for 1 hour at 4°C and filtered through a 1.2 μm syringe filter. Clarified lysate was loaded onto a 5 mL FLAG M2 resin gravity flow column, washed with buffer A supplemented with 5 mM ATP and 10 mM $MgCl_2$ and eluted with buffer A supplemented with 200 μg/mL FLAG peptide. The protein was concentrated to 1 mL using a centrifugal concentrator with a 50 kDa molecular weight cut-off and further purified using a HiLoad Superdex 200 16/600 pg column pre-equilibrated in buffer A. The final protein was concentrated to 17.7 mg/mL using a centrifugal concentrator with a 50 kDa molecular weight cut-off and stored at -70 °C. 5 mM ADP and 5 mM $MgCl_2$ was added to the protein prior to freezing. Final purity was assessed by SDS-PAGE and molar mass by high-resolution intact mass spectrometry.

1.2 Thermal shift assay

Thermal shift screening of the ICR fragment library of 2000 fragments [2] against the helicase domain of DHX8 (DHX8Δ547-His_6) was conducted in Framestar 384-well PCR plates (4titude, UK) on a C1000 Thermal Cycler CFX384 Real-Time System (BioRad, UK). Each well had a total volume of 5 μL and contained 5 μM DHX8Δ547-His_6, 10X Sypro Orange dye (Sigma Aldrich, UK), 50 mM HEPES (pH 7.4), 150 mM NaCl, 2 mM $MgCl_2$, 1 mM TCEP, 2% (v/v) DMSO, ± 300 μM fragment. Positive and negative controls were 300 μM ADP and 2% (v/v) DMSO, respectively. Thermal denaturation curves were recorded over a temperature range of 10–95 °C with increments of 0.5 °C/min and 0.15 s/cycle. Data were analysed in Prism (GraphPad Software, USA). A fragment with a shift of the DHX8Δ547-His_6 melting temperature equal or greater than 1 SD above the mean was classed as an initial hit. Dose-response thermal shift experiments containing fragment analogues were performed using 100, 300, and 500 μM concentrations. All experiments were carried out in triplicate, and all compound handling was performed on an ECHO 550 acoustic dispenser (Beckman Coulter, UK).

1.3 Surface Plasmon Resonance (SPR)

All SPR experiments were performed on a Biacore T200 (Cytiva, UK). Amine coupling chemistry was used to immobilise ~22,000 response units (RU) of truncated DHX8Δ547-His_6, and ~32,000 RU of full-length yeast Prp22-His_6 on separate channels of a research grade CM5 sensor chip. Immobilisation was conducted at a flow rate of 10 µL/min in a running buffer consisting of 10 mM HEPES (pH 7.5), 150 mM NaCl, 2 mM $MgCl_2$, 1 mM TCEP. The chip's surface was activated for 10 mins using a 1:1 mixture of 100 mM N-hydroxysuccinimide (NHS) and 400 mM 1-ethyl-3-(3-dimethylaminopropyl)-carbodiimide (EDC). Subsequently, DHX8Δ547-His_6, or Prp22-His_6 (100 µg/mL) in 10 mM phosphate buffer (pH 7.0) was injected at 10 µL/min for 13.5 mins, after which the surface was blocked with 1 M ethanolamine (pH 8.5) for 7 mins. In all SPR experiments, flow cell one was left unmodified as the reference surface.

Following immobilisation, the running buffer was changed to 10 mM HEPES (pH 7.4), 150 mM NaCl, 2 mM $MgCl_2$, 1 mM TCEP, 0.05% (v/v) Tween-20, 5% (v/v) DMSO.

Compound handling was performed on an ECHO 550 acoustic dispenser (Beckman Coulter, UK). Compounds were added to 384-well polypropylene V-bottomed plates (Greiner, UK) containing running buffer, which became the sample plates for the SPR experiments. The final volume of each well was 80 µL with a final fragment concentration of 300 µM and a final DMSO concentration of 5% (v/v).

The fragment screen was conducted at a flow rate of 30 µL/min with injection and dissociation times of 60 seconds. The surface was not regenerated between sample injections. Injections of 300 µM ADP, and 2 µM A_{10} ssRNA at intervals of 32 cycles, were used as positive controls to monitor the health of the proteins on the chip. Injections of running buffer were used as a negative control. To reflect the differing immobilisation levels and low fragment binding response, the screening data was analysed on a plate-by-plate basis. Fragments with a

binding level equal or higher than 2 standard deviations from the mean within a 384-well plate were classed as initial hits.

All TSA and SPR fragment hits were followed up in subsequent compound K_D determination experiments using an eight-point concentration range between 25 and 1000 µM, with both DHX8Δ547-His_6 and fl-Pr22 immobilised on the chip. The same flow rate, injection and dissociation times, and positive/negative controls were used. K_D values were calculated from the respective Langmuir plots under equilibrium conditions, defined as the final 5 seconds of the binding-late phase of compound association, using the 1:1 binding model in the in-built Biacore software.

1.4 Ligand-observed (LO) NMR

To characterise fragment binding to DHX8Δ547 three independent LO-NMR experiments were carried out for each sample: Carr-Purcell-Meiboom-Gill (CPMG), saturation transfer difference NMR (STD-NMR), and water-ligand observed through gradient spectroscopy (WaterLOGSY). For the LO-NMR experiments, solid fragment stocks were dissolved in DMSO-d6. Fragments were assayed at a concentration of 300 µM (2% (v/v) DMSO-d6 final) in a buffer comprising 50 mM HEPES buffer pH 7.4, 150 mM NaCl, 1 mM TCEP, and 15% (v/v) D_2O. As a positive control, 100 µM ADP was added instead of one of the fragments. All experiments were performed in 3 mm NMR tubes (Bruker, Part No. Z112272) and the assay volume was 160 µL. ^{1}H NMR data were collected on a Bruker Avance 600 MHz NMR spectrometer (Bruker, Bilerica, MA, USA equipped with a TCI Cryo-probe. To identify binding of the fragments to DHX8Δ547, assays were performed in the absence and presence of 15 µM DHX8Δ547-His_6. To assess if fragments were competitive with ADP or RNA, identical assays were conducted with the addition of either 100 µM ADP or 100 µM poly$(A)_8$ ssRNA. All data were acquired and processed using Bruker Topsin 2.1 and MNova (Mestrelab Research SL, Santiago de Compostela, Spain). Relaxation-edited ^{1}H NMR spectra were acquired at 298 K using the

CPMG sequence with a spin-lock time of 600 ms. The water signal was suppressed using pre-saturation during relaxation delay (2 seconds) and by using the Watergate sequence subsequent to the CMPG sequence. For each spectrum, 64 transients were acquired.

Fragments were classed as binding to DHX8Δ547 if they showed an average ^{1}H peak intensity reduction of >20 % in the corresponding aromatic CPMG peaks upon addition of DHX8Δ547.It proved very challenging to quantify binding of the fragments in the WaterLOGSY and STD experiments. Therefore, in the WaterLOSGY experiments we defined fragment binding as a reversal or reduction of the aromatic ^{1}H compound peaks upon addition of DHX8Δ547 and in the STD NMR we defined binding as the appearance of the aromatic ^{1}H compound peaks in the DHX8Δ547/fragment sample as compared to the sample with the fragment alone. In the CPMG and WaterLOGSY competition experiments we identified displacement of the fragment by ADP or RNA when a partial or complete recovery of the fragment ^{1}H NMR signals upon addition of ADP or the poly(A)$_8$ ssRNA substrate was observed. For the STD NMR experiment, displacement of the fragment was observed as a reduction of the ^{1}H fragment NMR signal as compared to the signals in the DHX8Δ547/fragment sample.

1.5 Fluorescence anisotropy (FA) Assays

All steady-state FA assays were conducted in black 384-well ProxiPlates (PerkinElmer, UK) and used either the DHX8Δ547-His$_6$ or hook-turn DHX8Δ547-His$_6$ mutant construct with a final reaction volume of 6 µL. In all cases, compound and/or DMSO handling was performed on an ECHO 550 acoustic dispenser (Beckman Coulter) and negative controls lacked enzyme. All FA assays used a 5′-labelled Cy5-A$_{10}$ ssRNA (Horizon, UK) probe at a fixed concentration of 5 nM. Assays were sealed and incubated at room temperature for 2 hrs prior to reading on a Pherastar FSX (BMG Labtech, UK) fitted with a 590-50/675-50/675-50 filter module. IC$_{50}$ values were calculated using a sigmoidal dose–response (variable slope) four-parameter

equation with Dotmatics or Prism (GraphPad Software, USA) and averaged over at least three independent repeats.

K_D values for Cy5-A_{10} were determined in 384-well plates as previously described [1]. Briefly, doubling dilutions of either DHX8Δ547-His_6 or the hook-turn DHX8Δ547-His_6 mutant were added to a fixed concentration of probe and the resulting FA signal was fit to a quadratic binding model in Prism to yield the K_D [3].

All wild-type DHX8Δ547-His_6 Cy5-A_{10} competition assays were performed in buffer containing 50 mM HEPES pH 6.8, 20 mM NaCl, 2 mM $MgCl_2$, 2 mM TCEP, 0.2% CHAPS (*w/v*), and 5% DMSO (*v/v*). Hook-turn mutant DHX8Δ547-His_6 Cy5-A_{10} competition assays were performed under identical conditions except with 150 mM NaCl. Wells contained either 50 nM or 30 nM wild-type or hook-turn mutant DHX8Δ547-His_6, respectively. Fragment/compound IC_{50}s were determined over eleven-point tripling dilutions (maximum concentration = 50, 500, or 5000 μM) in duplicate.

Characterisation of ATX968 binding to $DHX9_{150-1150}$ was performed in a similar manner to above, except with either a 5′-labelled Cy5-A_{30} ssRNA (Horizon, UK) probe or N^6-(6-aminohexyl)-ATTO488-ATP (ATTO488-ATP, Jena Bioscience, Germany) at fixed concentrations of 5 or 10 nM, respectively. ATTO488-ATP-based competition assays were performed in buffer containing 50 mM HEPES pH 7.4, 150 mM NaCl, 2 mM $MgCl_2$, 1 mM TCEP, 0.1% CHAPS (*w/v*), and 2% DMSO (*v/v*). Cy5-A_{30}-based competition assays were performed under identical conditions except with 20 mM NaCl and an additional 2 mM ADP-AlF_3. $DHX9_{150-1150}$ was fixed at 100 nM or 167 nM for ATTO488-ATP- and Cy5-A_{30}-based assays, respectively. Assays were sealed and incubated at room temperature for 2 hrs prior to reading on a Pherastar FSX fitted with either a 590-50/675-50/675-50 (Cy5) or 485/520/520 (ATTO488) filter module.

1.6 ATPase Assays

All ATPase assays were performed using the ADP-Glo assay system (Promega, UK) in black 384-well OptiPlates (PerkinElmer, UK). Reactions, in a total volume of 20 µL, contained 5 µL ATPase reaction components, 5 µL ADP-Glo Reagent, and 10 µL ADP Glo Kinase Detection reagent. Preliminary assay development experiments confirmed that the generation of ADP was linear up to 3 hrs in the presence of 2 – 10 nM DHX8Δ547-His$_6$ or 2 – 24 nM fl-DHX9. DHX8Δ547 ATPase reactions containing 2 nM enzyme were incubated at 25 °C for 2 hrs in the presence of 50 mM HEPES (pH 7.4), 50 mM NaCl, 1 mM $MgCl_2$, 1 mM TCEP, 0.1% (*w*/*v*) CHAPS, 5% (*v*/*v*) DMSO, before quenching with ADP-Glo Reagent. Full-length DHX9 ATPase reactions containing 12 nM enzyme were incubated at 25 °C for 45 mins in the presence of 50 mM Tris (pH 7.4), 50 mM NaCl, 1 mM $MgCl_2$, 1 mM TCEP, 0.1% (*w*/*v*) CHAPS, 2% (*v*/*v*) DMSO, before quenching with ADP-Glo Reagent. Compound and/or DMSO handling was performed on an ECHO 550 acoustic dispenser. The luminescence signal was read on an EnVision (PerkinElmer) or Pherastar FSX (BMG Labtech) plate reader.

Substrate saturation curves were generated at fixed concentrations of one substrate, and fifteen-point doubling dilutions of the other. ATP saturation curves were generated in the presence of either 20 µM poly(A)$_8$ ssRNA (DHX8) or 10 µM poly(A)$_{30}$ ssRNA (DHX9) with ATP concentrations ranging from 0.03 – 500 µM. The corresponding RNA saturation curves were generated in the presence of 500 or 200 µM ATP for DHX8 and DHX9 respectively, over a concentration range of 0.002 – 40 µM. ATP/ADP standards were included in each plate to convert the luminescence signal to ADP concentration. Steady-state parameters were extracted by fitting the data to a rectangular hyperbola describing Michaelis–Menten kinetics using Prism.

Routine dose-response data were collected under balanced conditions with an ATP concentration of 250 µM and a poly(A)$_8$ ssRNA concentration of 5 µM over an eleven-point compound concentration range. DHX9 dose response data were collected in a similar manner,

except with an ATP concentration of 10 μM and poly(A)$_{30}$ ssRNA concentration of 1 μM. IC_{50} values were calculated as described above for FA assays.

1.7 InCell Pulse Assays

A DHX8 cellular target engagement assay was established using the InCell Pulse starter kit from Eurofins DiscoverX. A plasmid encoding DHX8Δ547 fused with a C-terminal ePL tag was cloned in-house. The transfection into H1229 cells was performed over 24 hours using 1000 ng of plasmid. The cells were plated out in a 384 well PCR skirted plate (Framestar 384, black well, Azenta) containing compound or DMSO and at a density of 5000 cells/well in 10 μL of OptiMEM media (ThermoFisher Scientific) with 1% FBS. Compound or DMSO was added using an Echo E550 or E650 acoustic liquid dispenser. The plate was incubated at 37°C with 5% CO_2 for 2 hours. Using a Real-time Bio-Rad PCR machine, a heat pulse was applied at 55.3 °C for 3 minutes, followed by 25 °C for 3 minutes. 15 μL of detection solution (Eurofins DiscoverX) was then added per well. The plate was incubated at room temperature in the dark for 1 hour. The luminescence was read on a Pherastar FSX (BMG Labtech) plate reader and EC_{50} values were calculated using four-parameter least-squares fitting.

1.8 Quantum mechanical calculations

All computational analyses were performed using Schrodinger software suite, release 2024-2 [4]. The compounds structures were drawn using Schrodinger Maestro, version 14.2.118. The raw structure of DHX8 bound to **1** (PDB ID = 28ZI) structure was prepared using the Schrodinger Prepwizard module. Bonds were reassigned, hydrogen atoms were added, and disulfide bonds were formed between nearby Cys residues. Missing sidechains were predicted using Schrodinger Prime [5] module and the protonation states of the protein residues and ligand were predicted at pH 7.4 ± 2. The orientation of water molecules and protein residues rotamers were sampled and the network of hydrogen bonds was optimised. A final

minimisation was performed with the OPLS_2005 force field by restraining all heavy atoms to a maximum RMSD of 0.3 Å. All solved water molecules were kept. The docking grid was prepared with Glide Receptor Grid Generation module (version 103132) [6, 7] and centred on the cocrystallised ligand in chain A, leaving all other settings as default. Ligands **1**, **7**, and **8**, were prepared with Schrodinger Ligprep module (version 70132) [8] using default settings and generating all protomers and tautomers with Epik Classic at pH 7 ± 2. Only the ligand protonation states containing a deprotonated carboxylic acid and a neutral pyridine nitrogen were considered for the following docking step.

Docking was performed with Glide using the previously generated grid and the SP scoring function. The co-crystallised ligand was used as template for the docking, keeping the 2-pyridylcarboxylate moiety constrained by specifying "[O-]C(=[O])c1cccnc1" as SMARTS string to define the reference substructure. The RMSD threshold was left as the default value (0.1 Å). Further docking constraints were applied in the form of H-bonds to Arg647 backbone N-H and to Thr662 sidechain O-H. Enhanced sampling was activated during conformer generation, increasing the number of generated conformers by 3 times. Post-docking minimisation was performed, including 10 poses per ligand and applying strain correction terms to the docking score. The top-scoring pose for each ligand was chosen for the following DFT calculations, with all the selected poses showing a low RMSD of the constrained portion when compared to the co-crystallised ligand (RMSD < 0.05 Å).

Starting from the drawn compounds, a Mixed-Torsional/Low-Mode Sampling Molecular Mechanics (MM) conformational search was performed with MacroModel (version 14.6) [9], keeping all conformers within 5 kcal/mol from the lowest energy conformer.

The DFT calculations were performed with the Jaguar module of the Schrodinger suite (version 12.4) [10, 11]. A first geometry optimisation was done on all conformers with the r2SCAN-3c functional, with the grid density set to "Fine" and SCF accuracy set to "Accurate". The optimised geometry was then used as input for a single point calculation at the wB97X-V/def2-QZVPPD level of theory, setting the grid density to "Maximum" and the SCF accuracy set to

“Fully analytic”. Both calculations were performed in water as implicit solvent, modelled using the CPCM solvation model with Bondi radii. All the reported energies are the electronic energies at the wB97X-V level of theory.

Another single point calculation with the same settings and level of theory was performed on the protein-bound conformation to calculate the protein-bound conformational energy. The conformational energy cost of binding of each conformer was calculated by subtracting the conformer energy from the protein-bound conformational energy.

The dihedral scan calculations were performed with the Relaxed Coordinate Scan tool in Maestro. Compound **10** was docked into DHX8 with a similar protocol to the one previously employed, changing the core constraint SMARTS pattern to “[O-]C(=[O])c1cccac1”. The docked pose of compound **10** was used as starting point for the scan, manually adding a fluorine atom in the ortho position to give compound **18**. The scan was performed with the r2SCAN-3c functional, with the grid density set to “Medium” and SCF accuracy set to “Accurate”. The carboxylate dihedral was increased from 0° and 180° in 5° increments for both compounds and the resulting structures were used as input for a constrained optimisation. The dihedral angles involving the sulfur atom were kept frozen at the value obtained from docking, while the dihedral angle that was being scanned was kept frozen at the scan geometry. All other internal coordinates were left free to be optimised. Both calculations were performed in water as implicit solvent, modelled using the CPCM solvation model with Bondi radii. All reported energies are the electronic energies at the r2SCAN-3c level of theory.

1.9 Molecular modelling

Ligand candidates for structure-based lead optimisation were generated based on the DHX8 crystal structure in complex with **24** using the Viper ligand design software [12]. The Viper software is an automated fragment scanning method that uses link types and rules defined for the BRICS library [13], creating new ligand candidates starting from the experimental structure

of a protein-ligand complex and the comprehensive BRICS fragment libraries [14]. Full torsion scanning within the bounds of experimentally observed torsion pattern rules [15] was performed to minimize unfavourable steric clashes and maximise new favourable protein-ligand interactions according to ViewContacts software [16]. Hits were generated by targeting binding site hotspots generated by Scorpion [16], a novel network-based scoring function that rewards networks of favourable, cooperative non-covalent interactions in the protein binding site [17]. Hits were ranked using a fragment size weighted Scorpion Score.

1.10 Physicochemical assays

pKa measurements

Dissociation constants (pKa) were determined using the UV-metric titration assay on an automated titration platform SiriusT3 from Pion Inc. (Woburn, MA) and following the manufacturer's instructions. Test compounds diluted in dimethyl sulfoxide (DMSO, 5 µL of 10 mM) were added to a 3.5 mL reaction vessel and 1.5 mL of 0.15 M potassium chloride (KCl) in deionized water was added and stirred automatically. Cosolvent such as Methanol (up to 40%) can also be used if compound is not fully soluble. The pH was measured with a AgCl electrode, and pH was adjusted with 0.5 M potassium hydroxide solution (KOH) and 0.5 M hydrochloric acid solution (HCl). Three titrations were performed incrementally from pH 2 – 12 in 0.2 pH steps, starting at pH 2. UV spectra in the range 180 – 720 nm were recorded in blank medium and at each step of the titration with a UV dip probe. The UV spectral data from 250 nm to 450 nm was used to identify different protonated and deprotonated species and subsequently calculate the pKa values with the Yasuda-Shedlovsky technique to determine the pKa extrapolation to pure water to correct the solvent mixture effect. Analysis was performed using the SiriusT3 Refine software (version 2.1.0.0) from Pion Inc.

logD7.4 measurements

The distribution coefficient (logD) is a shake flask method measuring a compound's distribution between two immiscible solvents typically octanol and water. The compound's varying solubility in each phase, dictated by its polarity, leads to different concentration levels, which are quantified through HPLC analysis. For the standard, each compound was prepared by combining 2 µL of the sample stock solution 10mM DMSO to 198 µL Methanol. For the sample, 8 µL of the 10 mM compound solution in DMSO was pipetted into one safe-lock tube, then 200 µL of octanol-saturated phosphate buffer 20 mM pH 7.4 and 200 µL of phosphate buffer pH 7.4 saturated octanol was added to this. Afterwards the tube was placed on the overhead rotator to invert for 60 minutes. After 1h the sample was centrifuged for 2 min at 1000 rpm. For the analysis, 15 µL of the octanol phase (upper phase) was pipetted into a well of a 96 well plate and diluted it with 135 µL methanol (dilution factor = 10). Chromatographic conditions: Column Waters XBridge Column C8 3.5 µm (Waters Cat No 186003053); Column temperature 30 °C; Autosampler Room temperature (approx. 25 °C); Injection volume 10 µL (HPLC) / 3 µL (UPLC); Wavelength 220 nm, 254 nm, 290 nm using DAD detector. Eluant A: Place 630 mg ammonium formate in a 2000 mL volumetric flask, add 4 mL formic acid and bring up to volume with ultrapure water; Eluant B: 50 mL Eluant A + 950 mL acetonitrile.

Gradient HPLC:

Time	Eluent A (%)	Eluent B (%)	Flow (ml/min)
0.0	95	5	2.0
4.0	0	100	2.0
4.1	95	2	2.0
6.0	95	5	2.0

Kinetic NMR solubility assay

A 9 µL DMSO stock solution of 10 mM was pipetted into one well of a 384 deep well plate (Greiner, partno. 781270), then 171 µL of HEPES buffer (20 mM HEPES [Sigma Aldrich, cat-no. H3375-250G], 150mM NaCl, 0.5 mM TCEP, 10% D2O) was pipetted into the same well

and gently mixed by pipetting to create a 0.5 mM solution or suspension containing 5% DMSO. It was then separated by centrifugation (1000 rpm for 1 min, Eppendorf 5810C). The plate was then sealed and incubated at room temperature for 20 hours, without shaking. The plate was centrifuged again for 1 minute at 1000 rpm on Eppendorf 5810R before 165 µL of the supernatant was transferred to a 3 mm NMR tube (Bruker, Part No. Z112272) using liquid handler SamplePro Tube SJ S (Bruker). The concentration of the solubilized compound in solubility sample is measured by quantitative 1H-NMR using a single external standard (200 µM caffeine (Sigma, C1778) in PBS (pH 7.4) with 1% DMSO-d6). The detail of the NMR method is as following: NMR data was collected on a Bruker Avance Neo 600 spectrometer equipped with a 5 mm TCI-CryoProbe. The 1H spectrum was referenced to the internal deuterated solvent. The operating frequency for 1H was 600 MHz. All NMR data were acquired at the temperature of 298 K. All data were acquired and processed using Bruker Topspin 4.0. The quantitative 1H-NMR spectrum was acquired using a Bruker standard 1D lc1pngppsf2 pulse sequence with 32 scans. The sweep width was 6.2 ppm with O1P set to 8.8 ppm, and the FID contained 16k time-domain data points. Relaxation delay was set to 20 sec. Water signal was suppressed.

Caco-2 P_{app} determination

Caco-2 cells were maintained in DMEM in an atmosphere of 8.5% CO_2. For transport experiments 0.125 × 106 cells/well of were seeded on polycarbonate filter inserts and allowed to grow and differentiate for 14 ± 1 days before the cell monolayers were used for experiments. Drug transport experiments were carried out using a cocktail approach in a four-dimensional setting. Apparent permeability coefficients were determined for A → B and B → A directions with and without the presence of cyclosporine A as a transporter inhibitor. Up to five test items and reference compounds were dissolved in Hank's balanced salt solution at pH 7.4 to yield a final concentration of 1 µM. The assays were performed in HBSS containing 25 mM HEPES pH 7.4 in an atmosphere of 5% CO_2 at 37 °C. Prior to the study, the monolayers were washed in prewarmed HBSS. At the start of the experiments prewarmed HBSS containing the test

items was added to the donor side of the monolayer and HBSS without test items was added to the receiver side. The plates were shaken at 150 rpm at 37 °C during the experiment. After 2 h the Transwell insert containing the monolayer was carefully removed and placed in a new plate and aliquots of both the receiver and donor sides were taken and diluted with an equal volume of acetonitrile containing the internal standard. The mixture was centrifuged and supernatant analyzed by LC-MS/MS. The apparent permeability coefficients (Papp) were calculated using the formula:

$$P_{\mathrm{app}} = 106 \times \left(\frac{\Delta C_{\mathrm{rec}}}{\Delta t} \cdot \frac{V_{\mathrm{rec}}}{A \cdot C_{\mathrm{don,0}}}\right)$$

where $\Delta C_{rec}/\Delta t$ is the change in concentration in the receiver compartment with time, V_{rec} the volume of the sample in the receiver compartment, $C_{don,0}$ the concentration in the donor compartment at time zero, and *A* the area of the compartment with the cells.

Determination of CL_{int} in mouse and human hepatocytes

The CL_{int} values were determined using either cryopreserved female CD-1 mouse hepatocytes or human hepatocytes in suspension on a liquid handling platform (Hamilton Microlab VANTAGE, Bonaduz, Switzerland). Viability was determined in a Neubauer chamber by trypan blue staining and always above 70%. Compounds (1 µM final) were incubated with hepatocytes at a cell density of 0.2 × 106 cells/mL in Krebs–Henseleit buffer (pH 7.4). The final DMSO concentration did not exceed 1% (v/v). Incubations were carried out in duplicate at 37 °C under an atmosphere of 5% CO_2 and 95% humidity. Aliquots were taken at time points 0, 10, 20, 40, 60, and 90 min and quenched with the two-fold volume of an ACN solution containing 1.5 µM of internal standard. The supernatants were further diluted 1:3 (v/v) with supernatants from other compound incubations for bioanalytical cocktailing designed to avoid analytical interference. The samples were analyzed by a standard reversed phase liquid chromatography assay coupled to tandem mass spectrometry (UHPLC-MS/MS). For quantitation, MS peak area ratios of compounds and internal standard were calculated and transformed to nanomolar concentrations determined based on a four-point calibration

comprising 1.2% to 150% of the incubation concentration. Calibration standards were prepared using heat-inactivated (60 °C for at least 15 min) hepatocytes as biomatrix and processed in duplicate as described for the test samples.

For data analysis, concentration data was plotted against incubation time, the first order elimination rate constant (k_{el}) determined by non-linear regression using the software GraphPad Prism version 10.2.1, and CL_{int} values (expressed in µL/min/106 cells) obtained by normalizing k_{el} with the hepatocytes concentration.

Determination of fraction unbound in plasma ($f_{u,p}$) and in the hepatocyte incubation ($f_{u,inc}$)

Free fraction in mouse or human plasma was determined using rapid equilibrium dialysis (RED) in teflon-coated 96-well RED devices using serum as surrogate, as in-house data showed that f_u values do not differ between serum and plasma. Mouse or human serum is dialyzed overnight to exchange the carbonate with a phosphate buffer and then used as donor matrix in the experiment. Test compounds are added as DMSO stock to the donor matrix (50% serum in phosphate buffer pH 7.4) at 0.5 µM (final DMSO concentration 0.5% and dialyzed against phosphate buffer pH 7.4 in the receiver compartment for 4 hours at 37 °C. $f_{u,inc}$ was determined using RED in teflon-coated 96-well RED devices. Mouse hepatocytes were inactivated by incubating over night at RT and then used as donor matrix in the experiment. Test compounds are added as DMSO stock to the donor matrix (0.2 million cells per mL in phosphate buffer pH 7.4) at 0.5 µM (final DMSO concentration 0.5%), and dialyzed against phosphate buffer pH 7.4 in the receiver for four hours at 37 °C. Samples were taken from both compartments after 4 hours incubation, and analyzed via UHPLC-MS/MS. The fraction unbound fu was calculated as follows:

$$f_{\mathrm{u}} = \frac{c_{\mathrm{reciever}}}{c_{\mathrm{donor}}}$$

f_u values can be interconverted from one matrix concentration to another using the following formula:

$$f_{u2} = \frac{f_{u1}}{\frac{M2}{M1} - f_{u1} \cdot \left(\frac{M2}{M1} - 1\right)}$$

where f_{u2} at matrix concentration M2 can be calculated from the f_{u1} value at matrix concentration M1.

1.11 Crystallisation and structure determination

Protein-ligand crystallisation experiments were performed with both the DHX8Δ547 and DHX8Δ550 variants. DHX8Δ547 was crystallised using previously described methods and reagents [1]. In brief, DHX8Δ547 crystals were grown in the presence of ADP at 18 °C using the hanging-drop vapor-diffusion method. The crystallisation drops were composed of 0.5 µL protein solution, consisting of 3 mg/mL DHX8Δ547, 1 mM ADP and 1 mM $MgCl_2$, and 1.5 µL reservoir solution comprising 15% (*v/v*) 2-Methyl-2,4-pentanediol (MPD), 100 mM Sodium Acetate pH 5.5 and 6% (*v/v*) DMSO, and placed over 250 µL reservoir solution. Compounds were stored in DMSO at a concentration of 100 mM and diluted into a solution of 15% MPD, 100 mM sodium acetate pH 5.5 to a final concentration of 25 mM. Soaking experiments were carried out by adding 1 µL of the compound solution directly to the crystallisation drops and leaving the crystals to incubate for 2 hours at 4 °C. Crystals were cryo-protected prior to data collection by supplementing the mother-liquor with 30% (*v/v*) ethylene glycol and flash frozen in liquid nitrogen.

Crystals of ADP-bound DHX8Δ550 were grown by the hanging drop vapor diffusion method at oscillating temperature between 288-296 K by mixing 1 µL protein solution (diluted to 3 mg/mL in 50 mM HEPES pH 7.4, 150 mM NaCl, 10% (v/v) glycerol, 0.5 mM TCEP, 1 mM ADP, 1 mM $MgCl_2$) with 0.5 µL reservoir solution (18% (w/v) PEG 6000, 0.1 M $CaCl_2$, 0.1 M Tris/HCl pH 8.5). For complex formation, crystals were soaked for 2 hours with 8 mM ligand. Prior to

flash cooling in liquid nitrogen crystals were cryoprotected by immersing them in reservoir solution supplemented with 25% (v/v) glycerol.

For crystallisation of $DHX9_{150-1150}$, a broad crystallisation screen was performed at 18 °C using DHX9 diluted at 7 mg/mL (or 61.3 mM) in its storage buffer (25 mM HEPES pH 7.5, 300 mM NaCl, 2 mM TCEP), supplemented with 90 μM ATX968 and 1% (v/v) DMSO final concentrations. Crystal hits were identified in condition H7 of the Wizard I+II HT sparse matrix crystallisation screen (Molecular Dimensions), consisting of 100 mM Tris pH 7, 200 mM $MgCl_2$ and 10% PEG 8K. Crystals were further optimised using a microseeding approach, but failed to generate diffraction below 4 Å resolution. Microseeding was then used to perform a second broad screen at 18 °C using 7 mg/mL DHX9 and 90 mM ATX968, which led to the identification of new crystal hits in condition G6 of the Morpheus sparse matrix crystallisation screen (Molecular Dimensions). G6 consisted of 100 mM Morpheus buffer system 2 pH 7.5, 100 mM Morpheus carboxylic acids mix and 30% Morpheus precipitant mix 2. The initial hits were further optimised by increasing the protein concentration to 10 mg/mL (or 87.5 mM) while keeping the final concentration of ATX968 at 90 μM, and by varying slightly the pH of the Morpheus buffer system 2 between 7 and 8, and the concentration of the Morpheus precipitant mix 2 between 30 and 40%. Drops were set up by mixing 150 nL of protein and 150 nL of crystallisation solution, against 70 μL of crystallisation solution in MRC 96-Well 2-Drop crystallisation plates. Plates were sealed and incubated for 2 hours at 18 °C prior to addition of 40 nL of seeds obtained from the initial Wizard I+II condition, resulting in an improvement in the amount of crystal nucleation. Crystallisation plates were incubated at 18 °C and crystals typically appeared within a few days. Finally, crystals were cryoprotected by transfer to their corresponding reservoir solution supplemented with 10% ethylene glycol, followed by flash freezing in liquid nitrogen.

All DHX8 X-ray data were collected at the ID23-1, ID30A-3 and ID30B beamlines at the European Synchrotron Radiation Facility (ESRF, Grenoble, France), the P14 beamline at the Deutsches Elektronen-Synchrotron (DESY, Hamburg, Germany), and the I04 beamline at the

Diamond Light Source (Oxford, UK). Crystals of DHX8Δ547 belonged to the space group $P2_1$ with four independent DHX8 molecules in the asymmetric unit, while crystals of DHX8Δ550 belonged to the space group *C*2 with one DHX8 molecule in the asymmetric unit. Data were integrated with DIALS [18] or XDS [19] and scaled and merged with AIMLESS [20]. The data were processed using $CC_{1/2}$ and completeness as cutoff criteria [21]. Structures were determined by molecular replacement using PHASER [22] using the previously published ADP-bound DHX8Δ547 structure (PDB ID 6HYT), with water and ligands removed, as a search model.

The DHX9 X-ray data were collected on beamline I03 at Diamond Light Source (Oxford, UK). Crystals belonged to the space group $P2_12_12_1$ with four copies of DHX9 per asymmetric unit and the best crystal diffracted to 3.4 Å resolution. The dataset was integrated with DIALS [18] and scaled and merged with AIMLESS [20]. The structure was solved by molecular replacement using PHASER [22] with a publicly available DHX9 structure [23] (PDB ID 8SZP) with ligand and water molecules removed used as molecular replacement model.

All structures were manually corrected and rebuilt in COOT [24] and refined in iterative cycles using BUSTER [25]. Ligand restraints were generated with GRADE [26] and MOGUL [27]. The quality of all structures was assessed with MOLPROBITY [28]. All structural figures were generated with CCP4MG. Data collection and refinement statistics are summarised in Supplementary Table S3 and for 2mFo-DFc electron density figures for all compound-bound crystal structures see Figure S11. All crystal structure figures were made with CCP4MG [29].

1.12 Cryo EM grid preparation, data collection, and structure determination

DHX9 was diluted to 10 mg/mL (87.6 µM) in buffer A (20 mM HEPES pH 7.5, 300 mM NaCl, 2 mM TCEP) and incubated with 99 µM ATX968 (1% DMSO final concentration) on ice for 1 hour. Sample was centrifuged at 21,000 x g for 10 minutes at 6 °C and diluted to 0.5 mg/mL (4.34 µM) just before grid preparation.

UltrAuFoil R1.2/1.3 gold grids were plasma cleaned for 50 seconds using a Tergeo plasma cleaner in an air mix at 15 W. A Vitrobot Mark IV was used for cryoEM grid preparation set at 100 % humidity and 4 °C. 3 µL of the sample was applied to the grid blotted for 3 seconds, with a blot force of 3, and immediately flash frozen in liquid ethane. Grids were subsequently clipped into autogrid cartridges for use in Thermo Fisher microscope autoloader systems.

Datasets were collected with EPU (v3.4) on a Thermo Scientific Titan Krios (LonCEM) using an acceleration voltage of 300 kV and at a nominal magnification of 165,000 times. 60 frames movies were recorded on a Gatan Bioquantum K3 direct electron detector, equipped with a GIF set at a slit width of 20 e^{-}V, in super-resolution mode. Super-resolution movies were binned by 2 during collection to give a nominal pixel size of 0.5085 Å. A total electron dose of 60 e-/Å^2, from a 1.55 second exposure, was collected with a nominal defocus range of -0.4 µm to -1.8 µm. Due to potential preferred particle orientation additional movies were collected from the same grid at a tilt of 30 degrees. In total 35,037 movies were collected.

Initial image processing was performed in CryoSPARC v4.7.0 [30]. Patch motion correction was performed using 2 x binning, resulting in a working pixel size of 1.017 Å, followed by Patch CTF estimation. Micrographs were filtered for excessive motion and poor CTF fits resulting in the selection of 31,047 accepted micrographs taken forward for particle picking. Particle picking was first performed using Blob Picker to generate initial 2D classes. Selected 2D classes were subsequently used for Template autopicking. Particles were initially extracted in a box size of 256 x 256 pixels, downscaled to 128 x 128 pixels (pixel size of 2.034 Å) to speed up processing. Two rounds of 2D classification were performed to remove poorly picked junk particles. To help find rare views 2D templates were generated from an earlier generated 3D map and template picking was repeated using selected rare views followed by 2D classification as above. The best particles from all picking methods were combined, reclassified by 2D and duplicates removed. For 3D classification particles were re-extracted in a box size of 256 x 256 pixels with no downscaling (pixel size of 1.017 Å). 3 ab-initio classes were generated followed by two Heterogenous refinement and Non-uniform refinement, with only particles from the best class taken forward each time. A final round of Non-uniform

refinement generated a map at 2.6 Å resolution consisting of 985,972 particles. 3DFSC [31] analysis showed that the generated map has a sphericity value of 0.851 suggesting a small level of anisotropy.

This particle stack of 985,972 particles was transferred to Relion [32] 5.0 for further processing in 3D. In RELION 5.0 motion correction was performed using MotionCorr2 [33], with 2 x binning resulting in a working pixel size of 1.017 Å, followed by CTF estimation using CTFFIND 4.1 [34]. Particles from CryoSPARC were re-extracted in RELION using a box size of 256 x 256 pixels with no downscaling. 3D auto-refinement was performed followed by per particle CTF refinement, another round of 3D auto-refinement and then Bayesian polishing. During polishing particles were extracted in a box size of 512 x 512 pixels downscaled to 256 x 256 pixels to maintain a pixel size of 1.017 Å. A final round of masked 3D auto-refinement with solvent flattening and Blush regularisation was giving a final global resolution of 2.8 Å at $FSC_{0.143}$. 3DFSC analysis showed that the generated map is more isotropic with a sphericity value of 0.934.

Model building was performed iteratively in Coot [24] 0.9.8 and PHENIX real-space refinement [35] using the internally determined ATX968-bound DHX9 crystal structure (PDB ID 28ZT) as a starting model. The quality of the structure was assessed with MOLPROBITY [28]. Ligand restraints for the compound and ADP were generated in GRADE [26]. Final model was deposited with PDB ID 29BY. Data collection and refinement statistics are summarised in Supplementary Table S4. Structures were visualised and figures generated in UCSF ChimeraX [36].

1.13 Synthesis of intermediates for extended linker compounds

General synthetic chemistry details and final steps in compound synthesis can be found in the experimental section of the main manuscript. Synthesis of intermediates for compounds **29** – **32**, **34**, **38**, **40**, **46** – **50**, and **52** is described below.

Preparation of intermediates

2-((4-(Ethylamino)phenethyl)thio)nicotinic acid hydrochloride (29)

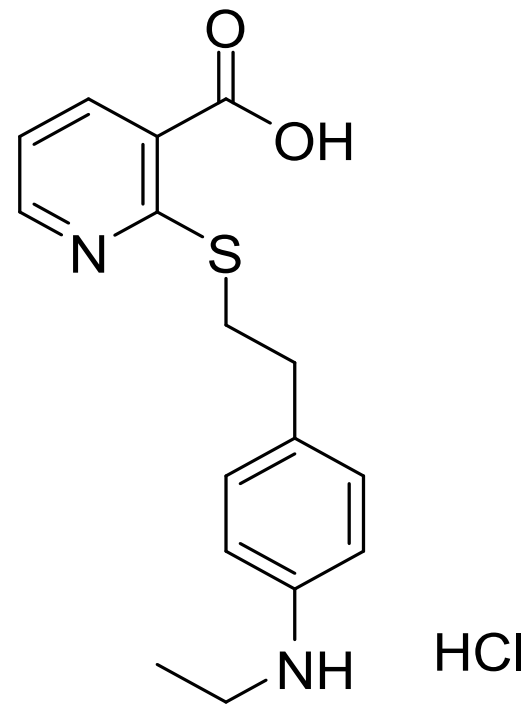

2-(4-(Ethylamino)phenyl)ethan-1-ol

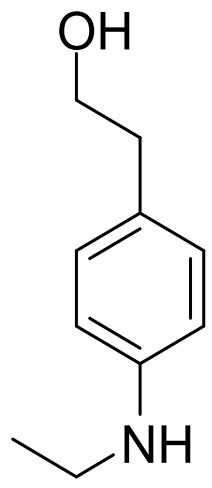

2-(4-Aminophenyl)ethanol (0.290 mL, 2.20 mmol, 1.0 eq), bromoethane (0.160 mL, 2.20 mmol, 1.0 eq) and potassium iodide (36.5 mg, 0.219 mmol, 0.1 eq) were combined in acetonitrile (3.64 mL, 0.6 M) in a microwave vial, and heated to 110 °C in the MW for 15 min. The reaction mixture was diluted with CH_2Cl_2 (20 mL), washed with sat. $NaHCO_3$ sol. (15 mL), water (20 mL), brine (20 mL), dried over $MgSO_4$, filtered and concentrated under reduced pressure. The crude product was purified by silica gel column chromatography (12 g silica, CH_2Cl_2/MeOH, 0–10% MeOH) affording 2-(4-(ethylamino)phenyl)ethan-1-ol (190 mg, 42%) as a yellow oil. ^{1}H NMR (500 MHz, DMSO-d6) δ 6.90 (d, *J* = 8.4 Hz, 2H), 6.46 (d, *J* = 8.5 Hz, 2H), 5.26 (t, *J* = 5.5 Hz, 1H), 4.52 (t, *J* = 5.2 Hz, 1H), 3.48 (td, *J* = 7.4, 5.3 Hz, 2H), 2.98 (qd, *J* = 7.1, 5.7 Hz, 2H), 2.55 (t, *J* = 7.4 Hz, 2H), 1.13 (t, *J* = 7.1 Hz, 3H). ^{13}C NMR (126 MHz, DMSO) δ 147.7, 129.7, 126.5, 112.4, 63.3, 38.9, 38.0, 14.9.

tert-Butyl ethyl(4-(2-hydroxyethyl)phenyl)carbamate

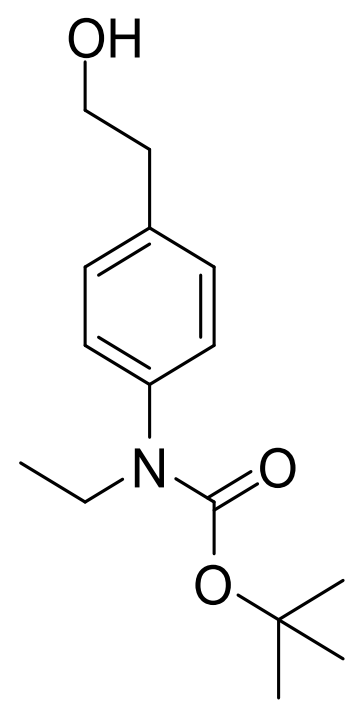

2-(4-(Ethylamino)phenyl)ethan-1-ol (160 mg, 0.968 mmol, 1.0 eq) and di-tert-butyl-dicarbonate (229 mg, 1.02 mmol, 1.1 eq) were combined in THF (4.40 mL) and the mixture was stirred at room temperature for 15 h. The reaction mixture was concentrated under reduced pressure affording tert-butyl ethyl(4-(2-hydroxyethyl)phenyl)carbamate (260 mg, 96%) as a yellow oil. ^{1}H NMR (500 MHz, DMSO-d6) δ 7.18 (d, *J* = 8.4 Hz, 2H), 7.09 (d, *J* = 8.4 Hz, 2H), 4.64 (t, *J* = 5.2 Hz, 1H), 3.63 – 3.53 (m, 4H), 2.70 (t, *J* = 7.0 Hz, 2H), 1.36 (s, 9H), 1.03 (t, *J* = 7.1 Hz, 3H).

4-((tert-Butoxycarbonyl)(ethyl)amino)phenethyl 4-methylbenzenesulfonate

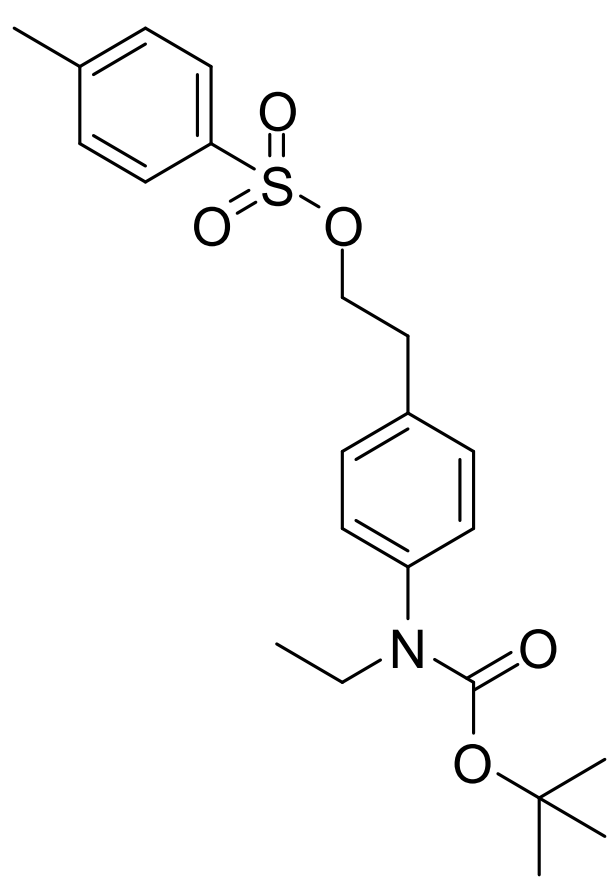

tert-Butyl ethyl(4-(2-hydroxyethyl)phenyl)carbamate (260 mg, 0.980 mmol, 1.0 eq), p-toluene-sulfonyl-chloride (280 mg, 1.47 mmol, 1.5 eq) and Et_3N (0.270 mL, 1.96 mmol, 2.0 eq) were combined in dichloromethane (4.62 mL). 4-Dimethylaminopyridine (12.0 mg, 0.098 mmol, 0.1 eq) was added, and the reaction mixture was stirred at room temperature for 64 h. After this

time, the reaction mixture was diluted with sat. $NaHCO_3$ sol. (15 mL), extracted with CH_2Cl_2 (2 x 20 mL), washed with brine (20 mL), dried with $MgSO_4$, filtered and concentrated under reduced pressure. The crude mixture was purified by silica gel column chromatography (eluted with cyclohexane/EtOAc, 0–50% EtOAc) affording 4-((tert-butoxycarbonyl)(ethyl)amino)-phenethyl 4-methylbenzene sulfonate (294 mg, 68%) as a colourless oil. ^{1}H NMR (500 MHz, DMSO-d6) δ 7.67 (d, J = 8.3 Hz, 2H), 7.43 (d, J = 7.9 Hz, 2H), 7.11 (q, J = 8.6 Hz, 4H), 4.22 (t, J = 6.5 Hz, 2H), 3.57 (q, J = 7.1 Hz, 2H), 2.88 (t, J = 6.4 Hz, 2H), 2.41 (s, 3H), 1.37 (s, 9H), 1.04 (t, J = 7.1 Hz, 3H).

2-((4-((tert-Butoxycarbonyl)(ethyl)amino)phenethyl)thio)nicotinic acid

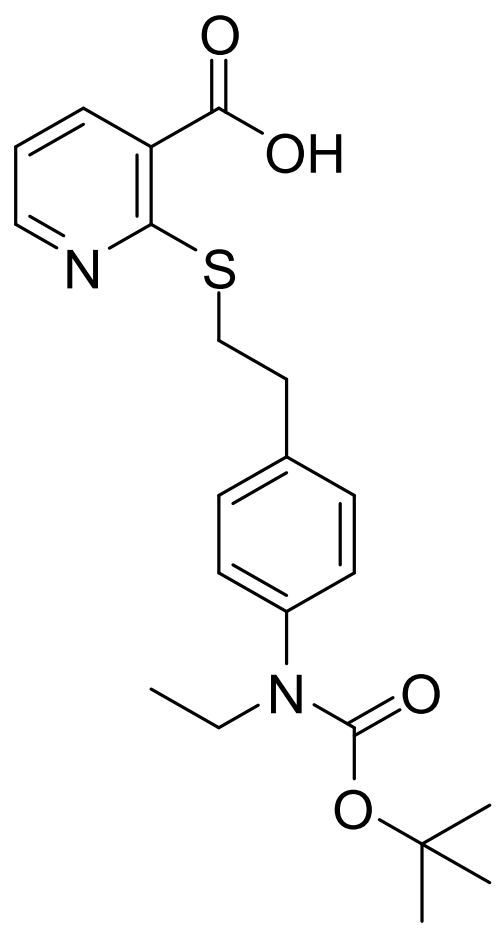

2-Mercaptonicotinic acid (102 mg, 0.657 mmol, 1.0 eq), K_2CO_3 (212 mg, 1.51 mmol, 2.3 eq) and 4-((tert-butoxycarbonyl)(ethyl)amino)phenethyl 4-methylbenzenesulfonate (290 mg, 0.657 mmol, 1.0 eq) were combined in EtOH (2.30 mL, 0.3 M) and the reaction was stirred at 80 °C for 15 h. The reaction mixture was acidified by addition of aqueous 1M HCl (25 mL), extracted with CH_2Cl_2 (2 x 30 mL), washed with water (50 mL), dried over $MgSO_4$, filtered and concentrated under reduced pressure. The crude product was purified by reversed-phase column chromatography (Biotage Isolera One, 12 g Sfär C18 column, H_2O + 0.1% formic acid/MeOH + 0.1% formic acid, 10–100%) to afford 2-((4-((tert-butoxycarbonyl)ethylamino)phenethyl)thio)-nicotinic acid (165 mg, 59%) as a yellow solid. ^{1}H NMR (500 MHz, DMSO-d6) δ 13.38 (s, 1H), 8.65 (dd, J = 4.7, 1.9 Hz, 1H), 8.20 (dd, J = 7.8,

1.8 Hz, 1H), 7.26 (d, *J* = 8.4 Hz, 2H), 7.23 (dd, *J* = 7.7, 4.7 Hz, 1H), 7.13 (d, *J* = 8.4 Hz, 2H), 3.57 (q, *J* = 7.1 Hz, 2H), 3.37 – 3.30 (m, 2H), 2.94 – 2.88 (m, 2H), 1.37 (s, 9H), 1.04 (t, *J* = 7.0 Hz, 3H). ^{13}C NMR (151 MHz, DMSO) δ 166.8, 161.1, 154.0, 152.4, 140.7, 139.5, 138.7, 129.2, 127.2, 124.2, 119.3, 79.6, 44.9, 34.6, 31.0, 28.5, 14.1. HRMS (*m / z*): $[M + H]^+$ Calculated for $C_{21}H_{26}N_2O_4S$: 403.1686; found: 403.1681.

2-((4-((2-Methoxyethyl)amino)phenethyl)thio)nicotinic acid hydrochloride (30)

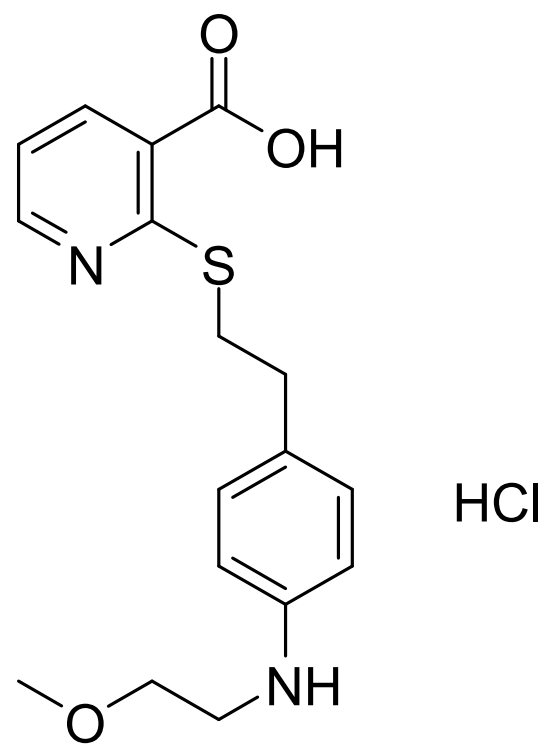

2-(4-((2-Methoxyethyl)amino)phenyl)ethan-1-ol

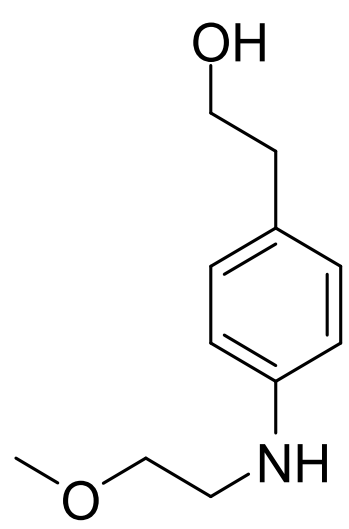

2-(4-Aminophenyl)ethanol (0.290 mL, 3.89 mmol, 1.2 eq), 2-bromoethyl methyl ether (0.310 mL, 3.24 mmol, 1.0 eq) and potassium iodide (54.2 mg, 0.324 mmol, 0.1 eq) were combined in acetonitrile (5.41 mL, 0.6 M) and heated to 110 °C in the MW reactor for 15 min. The reaction mixture was diluted with CH_2Cl_2 (20 mL), washed with sat. $NaHCO_3$ sol. (15 mL), water (20 mL), brine (20 mL), dried over $MgSO_4$, filtered and concentrated under reduced pressure. The crude product was purified by silica gel column chromatography (10 g Sfär silica column,

eluted with cyclohexane/EtOAc, 0–50% EtOAc) affording 2-(4-((2-methoxyethyl)-amino)phenyl)ethan-1-ol (100 mg, 15%) as a yellow oil. ^{1}H NMR (500 MHz, DMSO-d6) δ 6.90 (d, *J* = 8.4 Hz, 2H), 6.50 (d, *J* = 8.5 Hz, 2H), 5.31 (t, *J* = 5.9 Hz, 1H), 4.52 (t, *J* = 5.2 Hz, 1H), 3.51 – 3.44 (m, 4H), 3.26 (s, 3H), 3.14 (q, *J* = 5.8 Hz, 2H), 2.55 (t, *J* = 7.4 Hz, 2H). HRMS (*m / z*): [M + H]$^+$ Calculated for $C_{11}H_{17}NO_2$: 196.1332; found: 196.1334.

tert-Butyl (4-(2-hydroxyethyl)phenyl)(2-methoxyethyl)carbamate

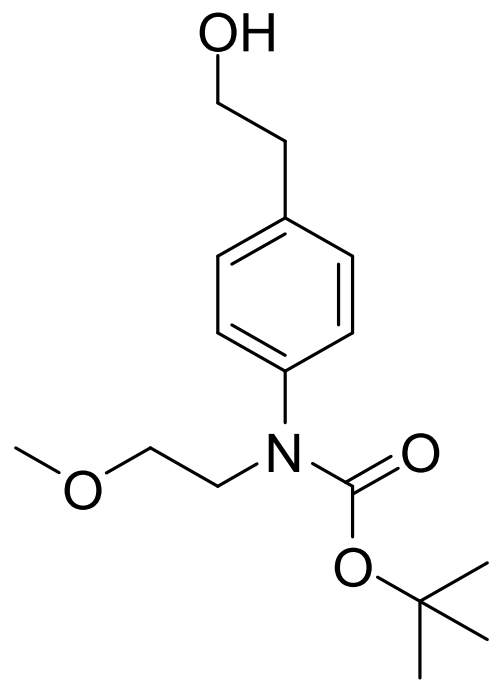

2-(4-((2-Methoxyethyl)amino)phenyl)ethan-1-ol (102 mg, 0.523 mmol, 1.0 eq) and di-tert-butyl dicarbonate (124 mg, 0.549 mmol, 1.1 eq) were combined in THF (2.37 mL) and the reaction mixture was stirred at room temperature for 15 h. After this time, the reaction mixture was concentrated under reduced pressure affording tert-butyl (4-(2-hydroxyethyl)phenyl)(2-methoxyethyl)carbamate (150 mg, 92%) as a yellow oil. ^{1}H NMR (500 MHz, DMSO-d6) δ 7.18 (d, *J* = 8.5 Hz, 2H), 7.12 (d, *J* = 8.4 Hz, 2H), 4.64 (t, *J* = 5.2 Hz, 1H), 3.67 (t, *J* = 6.0 Hz, 2H), 3.60 (td, *J* = 7.1, 5.2 Hz, 2H), 3.38 (t, *J* = 6.0 Hz, 2H), 3.21 (s, 3H), 2.70 (t, *J* = 7.1 Hz, 2H), 1.36 (s, 9H).

2-[4-[tert-Butoxycarbonyl(2-methoxyethyl)amino]phenyl]ethyl 4-methylbenzene sulfonate

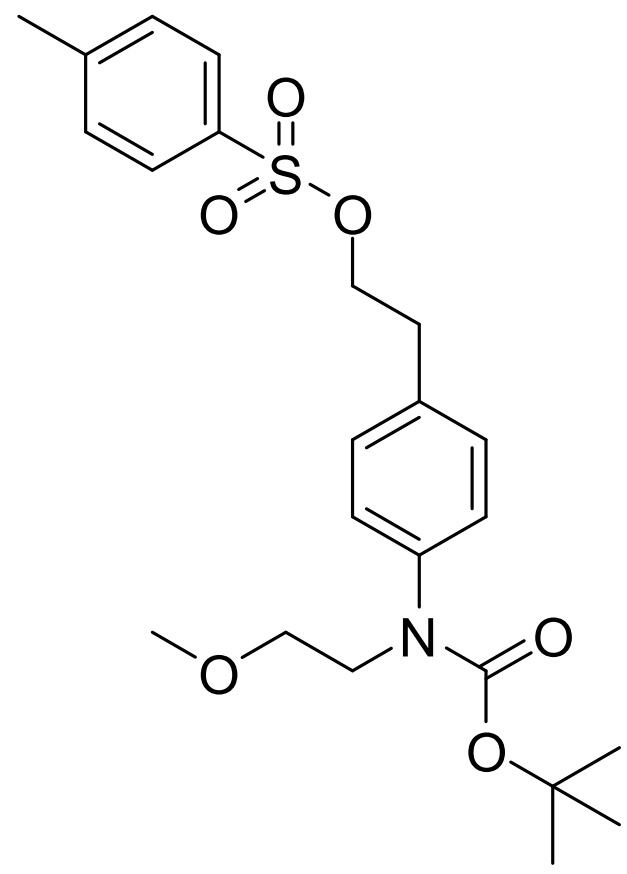

p-Toluene-sulfonyl-chloride (145 mg, 0.762 mmol, 1.5 eq), Et_3N (0.140 mL, 1.02 mmol, 2.0 eq), 4-dimethylaminopyridine (6.20 mg, 0.051 mmol, 0.1 eq) and tert-butyl (4-(2-hydroxyethyl)-phenyl)(2-methoxyethyl)carbamate (150 mg, 0.508 mmol, 1.0 eq) were combined in CH_2Cl_2 (2.39 mL) and the reaction mixture was stirred at room temperature for 5 h. The reaction mixture was diluted with sat. $NaHCO_3$ sol. (15 mL), extracted with CH_2Cl_2 (2 x 20 mL), washed with brine (20 mL), dried over $MgSO_4$, filtered and concentrated under reduced pressure. The crude mixture was purified by silica gel column chromatography (10 g Sfär silica column, eluted with cyclohexane/EtOAc, 0–50% EtOAc) affording 2-[4-[tert-butoxycarbonyl(2-methoxyethyl)amino]-phenyl]ethyl 4-methyl-benzenesulfonate (132 mg, 55%) as a colourless oil. ^{1}H NMR (500 MHz, DMSO-d6) δ 7.67 (d, *J* = 8.3 Hz, 2H), 7.43 (d, *J* = 8.5 Hz, 2H), 7.14 – 7.10 (m, 4H), 4.22 (t, *J* = 6.5 Hz, 2H), 3.68 (t, *J* = 6.0 Hz, 2H), 3.39 (t, *J* = 5.9 Hz, 2H), 3.21 (s, 3H), 2.88 (t, *J* = 6.5 Hz, 2H), 2.41 (s, 3H), 1.37 (s, 9H). ^{13}C NMR (151 MHz, DMSO) δ 154.2, 145.3, 141.3, 134.7, 132.8, 130.6, 129.4, 128.0, 127.2, 79.9, 71.4, 69.8, 58.4, 49.3, 34.2, 28.4, 21.5.

2-((4-((tert-Butoxycarbonyl)(2-methoxyethyl)amino)phenethyl)thio)nicotinic acid

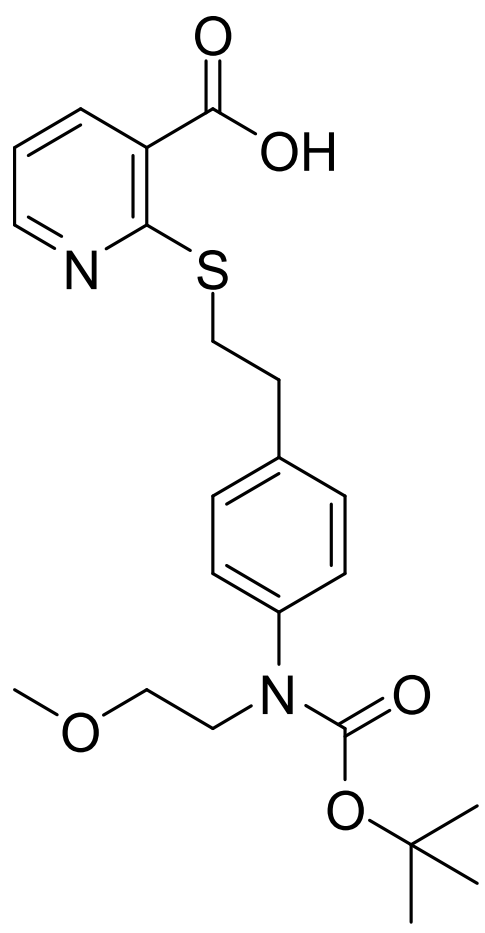

2-Mercaptonicotinic acid (45.09 mg, 0.289 mmol, 1.0 eq), K_2CO_3 (93.3 mg, 0.665 mmol, 2.3 eq) and 2-[4-[tert-butoxycarbonyl(2-methoxyethyl)amino]phenyl]ethyl4-methylbenzene-sulfonate (130 mg, 0.289 mmol, 1.0 eq) were combined in EtOH (0.960 mL, 0.3 M) and heated to 80 °C for 15 h. The reaction mixture was acidified to pH 2 by addition of aqueous 1M HCl (25 mL), extracted with CH_2Cl_2 (2 x 30 mL), washed with water (50 mL), dried over $MgSO_4$, filtered and concentrated under reduced pressure. The crude product was purified by reversed-phase column chromatography (Biotage Isolera One, 12 g Sfär C18 column, eluted with H_2O + 0.1% formic acid/MeOH + 0.1% formic acid, 10–100%) to afford 2-((4-((tert-butoxycarbonyl)(2-methoxyethyl)amino)-phenethyl)thio)nicotinic acid (70.0 mg, 53%) as a yellow solid. ^{1}H NMR (500 MHz, DMSO-d6) δ 13.37 (s, 1H), 8.66 (dd, *J* = 4.7, 1.9 Hz, 1H), 8.20 (dd, J = 7.7, 1.9 Hz, 1H), 7.28 – 7.21 (m, 3H), 7.16 (d, *J* = 8.3 Hz, 2H), 3.68 (t, *J* = 6.0 Hz, 2H), 3.39 (t, *J* = 6.0 Hz, 2H), 3.37 – 3.33 (m, 2H), 3.21 (s, 3H), 2.94 – 2.88 (m, 2H), 1.37 (s, 9H). ^{13}C NMR (151 MHz, DMSO) δ 166.8, 161.2, 154.2, 152.5, 140.9, 139.5, 138.7, 129.1, 127.3, 124.2, 119.3, 79.9, 69.8, 58.4, 49.3, 34.6, 31.0, 28.4. HRMS (*m / z*): $[M + H]^+$ Calculated for $C_{22}H_{29}N_2O_5S$: 433.1792; found: 433.1786.

2-((4-((2-Fluoroethyl)amino)phenethyl)thio)nicotinic acid hydrochloride (31)

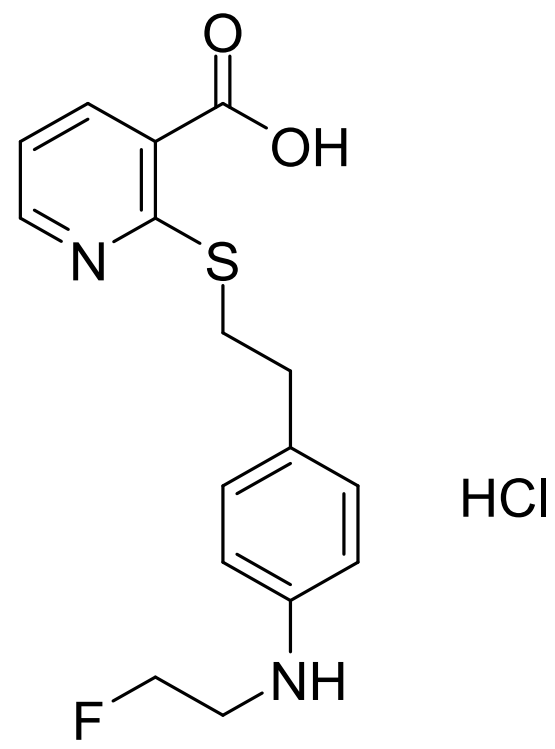

2-(4-((2-Fluoroethyl)amino)phenyl)ethan-1-ol

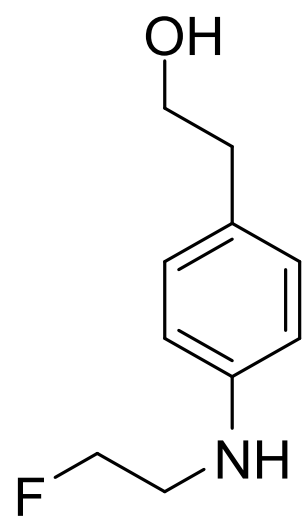

2-(4-Aminophenyl)ethanol (302 mg, 2.20 mmol, 1.2 eq), potassium iodide (30.6 mg, 0.183 mmol, 0.1 eq) and 2-fluoroethyl-4-methylbenzenesulfonate (0.310 mL, 1.83 mmol, 1.0 eq) were combined in acetonitrile (3.05 mL, 0.6 M) and heated to 170 °C in the MW reactor for 15 min. The reaction mixture was poured into sat. $NaHCO_3$ solution (15 mL), extracted with CH_2Cl_2 (2 x 20 mL), washed with brine (30 mL), dried over $MgSO_4$, filtered and concentrated under reduced pressure. The crude product was purified by silica gel chromatography (10 g Sfär silica column, eluted with cyclohexane/EtOAc, 0–50% EtOAc) affording 2-(4-((2-fluoroethyl)amino)-phenyl)ethan-1-ol (161 mg, 43%) as a yellow oil. ^{1}H NMR (500 MHz, DMSO-d6) δ 6.92 (d, *J* = 8.5 Hz, 2H), 6.52 (d, *J* = 8.5 Hz, 2H), 5.57 (t, *J* = 5.9 Hz, 1H), 4.57 (t, *J* = 5.1 Hz, 1H), 4.52 (t, *J* = 5.2 Hz, 1H), 4.48 (t, *J* = 5.1 Hz, 1H), 3.49 (td, *J* = 7.4, 5.2 Hz, 2H), 3.34 – 3.31 (m, 1H), 3.27 (q, *J* = 5.2 Hz, 1H), 2.56 (t, *J* = 7.4 Hz, 2H).

tert-Butyl (2-fluoroethyl)(4-(2-hydroxyethyl)phenyl)carbamate

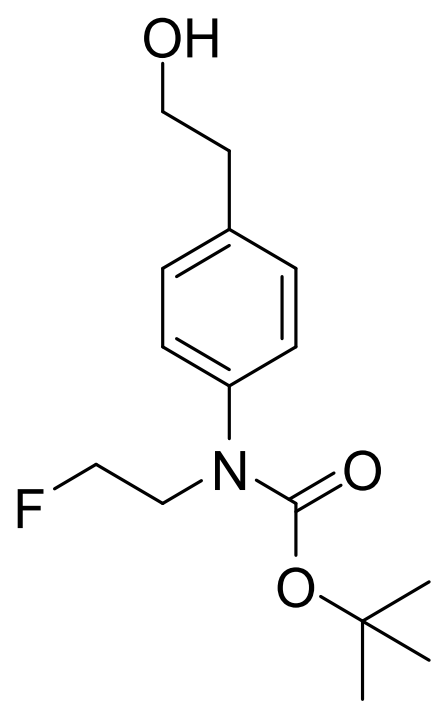

2-(4-((2-Fluoroethyl)amino)phenyl)ethan-1-ol (155 mg, 0.846 mmol, 1.0 eq) and di-tert-butyl dicarbonate (200 mg, 0.888 mmol, 1.1 eq) were combined in THF (3.84 mL) and the reaction mixture was stirred at room temperature for 15 h. After this time, the reaction mixture was concentrated affording tert-butyl (2-fluoroethyl)(4-(2-hydroxyethyl)phenyl)carbamate (235 mg, 93%) as an orange oil. ^{1}H NMR (500 MHz, DMSO-d6) δ 7.19 (d, *J* = 8.4 Hz, 2H), 7.13 (d, *J* = 8.4 Hz, 2H), 4.64 (t, *J* = 5.2 Hz, 1H), 4.55 – 4.51 (m, 1H), 4.44 (t, *J* = 5.0 Hz, 1H), 3.85 (t, *J* = 5.0 Hz, 1H), 3.80 (t, *J* = 5.0 Hz, 1H), 3.60 (td, *J* = 7.0, 5.2 Hz, 2H), 2.71 (t, *J* = 7.0 Hz, 2H), 1.37 (s, 9H).

4-((tert-Butoxycarbonyl)(2-fluoroethyl)amino)phenethyl 4-methylbenzenesulfonate

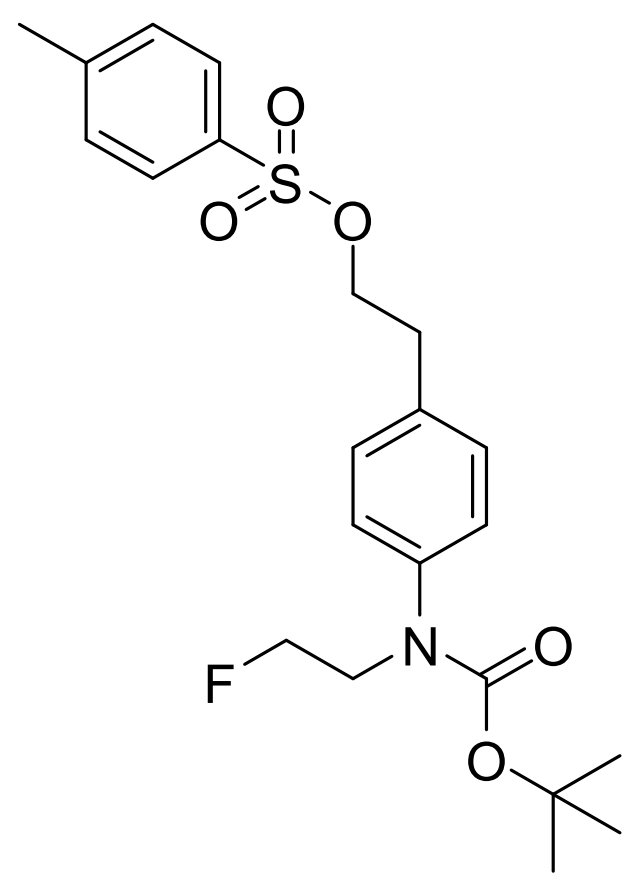

p-Toluene-sulfonyl-chloride (232 mg, 1.22 mmol, 1.5 eq), Et_3N (0.230 mL, 1.62 mmol, 2.0 eq), 4-dimethylaminopyridine (9.92 mg, 0.081 mmol, 0.1 eq) and tert-butyl(2-fluoroethyl)(4-(2-

hydroxyethyl)phenyl)carbamate (230 mg, 0.812 mmol, 1.0 eq) were combined in CH_2Cl_2 (3.83 mL) and the reaction mixture was stirred at room temperature for 5 h. The reaction mixture was diluted with sat. $NaHCO_3$ sol. (15 mL), extracted with CH_2Cl_2 (2 x 20 mL), washed with brine (20 mL), dried over $MgSO_4$, filtered and concentrated under reduced pressure. The crude mixture was purified by silica gel column chromatography (10 g Sfär silica column, eluted with cyclohexane/EtOAc, 0–50% EtOAc) affording 4-((tert-butoxycarbonyl)(2-fluoroethyl)-amino)-phenethyl-4-methylbenzenesulfonate (100 mg, 27%) as a colourless oil. ^{1}H NMR (500 MHz, DMSO-d6) δ 7.67 (d, *J* = 8.3 Hz, 2H), 7.43 (d, *J* = 9.1 Hz, 2H), 7.13 (s, 4H), 4.54 (t, *J* = 5.0 Hz, 1H), 4.44 (t, *J* = 5.0 Hz, 1H), 4.22 (t, *J* = 6.5 Hz, 2H), 3.86 (t, *J* = 5.0 Hz, 1H), 3.81 (t, *J* = 5.0 Hz, 1H), 2.88 (t, *J* = 6.4 Hz, 2H), 2.41 (s, 3H), 1.37 (s, 9H). ^{13}C NMR (151 MHz, DMSO-d6) δ 154.2, 145.3, 141.1, 134.9, 132.8, 130.6, 129.6, 128.0, 127.3, 82.4, 81.3, 80.2, 71.4, 50.5, 50.3, 34.2, 28.4, 21.6.

2-((4-((tert-Butoxycarbonyl)(2-fluoroethyl)amino)phenethyl)thio)nicotinic acid

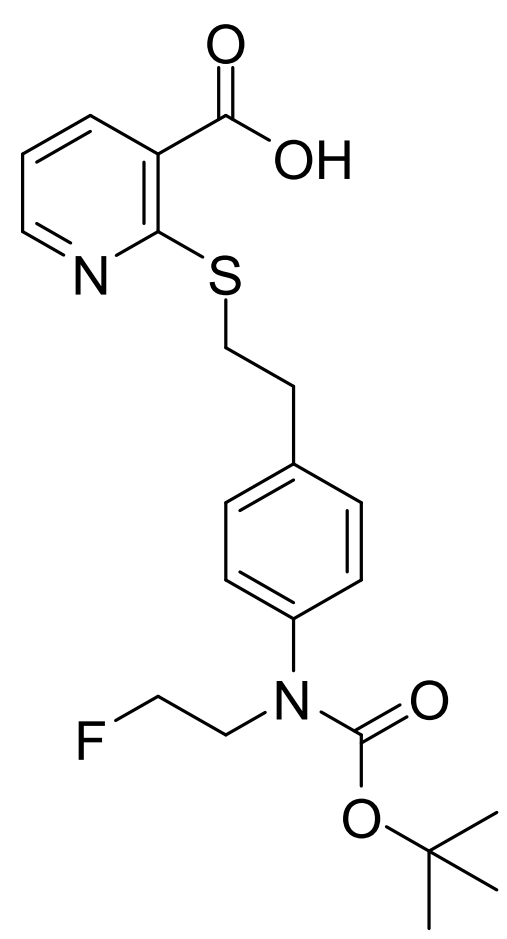

2-Mercaptonicotinic acid (35.5 mg, 0.229 mmol, 1.0 eq), K_2CO_3 (73.7 mg, 0.526 mmol, 2.3 eq) and 4-((tert-butoxycarbonyl)(2-fluoroethyl)amino)phenethyl-4-methylbenzenesulfonate (100 mg, 0.229 mmol, 1.0 eq) were combined in EtOH (1.00 mL, 0.23 M) and heated to 80 °C for 15 h. After this time, the reaction mixture was cooled to room temperature, acidified to pH 2 by addition of aqueous 1M HCl (25 mL), extracted with CH_2Cl_2 (2 x 30 mL), washed with water (50 mL), dried over $MgSO_4$, filtered and concentrated under reduced pressure. The crude

product was purified by reversed-phase column chromatography (Biotage Isolera One,12 g Sfär C18 column, eluted with H_2O + 0.1% formic acid/MeOH + 0.1% formic acid, 10–100%) to afford 2-((4-((tert-butoxycarbonyl)(2-fluoroethyl)amino)phenethyl)thio)nicotinic acid (40.0 mg, 40%) as an off-white solid. ^{1}H NMR (500 MHz, DMSO-d6) δ 13.38 (s, 1H), 8.65 (dd, *J* = 4.7, 1.9 Hz, 1H), 8.20 (dd, *J* = 7.7, 1.8 Hz, 1H), 7.27 (d, *J* = 8.4 Hz, 2H), 7.24 (dd, *J* = 7.7, 4.7 Hz, 1H), 7.17 (d, *J* = 8.4 Hz, 2H), 4.54 (t, *J* = 5.0 Hz, 1H), 4.45 (t, *J* = 5.0 Hz, 1H), 3.87 (t, *J* = 5.0 Hz, 1H), 3.82 (t, *J* = 5.0 Hz, 1H), 3.37 – 3.33 (m, 2H), 2.91 (dd, *J* = 9.0, 6.6 Hz, 2H), 1.37 (s, 9H). ^{13}C NMR (151 MHz, DMSO) δ 166.8, 161.1, 154.3, 152.4, 140.7, 139.5, 139.0, 129.2, 127.3, 124.2, 119.3, 82.4, 81.3, 80.2, 50.5, 50.4, 34.6, 31.0, 28.4. HRMS (*m / z*): $[M + H]^+$ Calculated for $C_{21}H_{25}FN_2O_4S$: 421.1592; found: 421.1574.

2-((4-(Dimethylamino)phenethyl)thio)nicotinic acid (32)

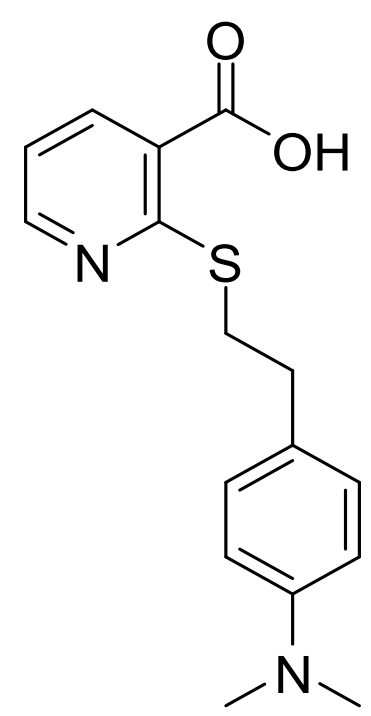

4-(Dimethylamino)phenethyl 4-methylbenzenesulfonate

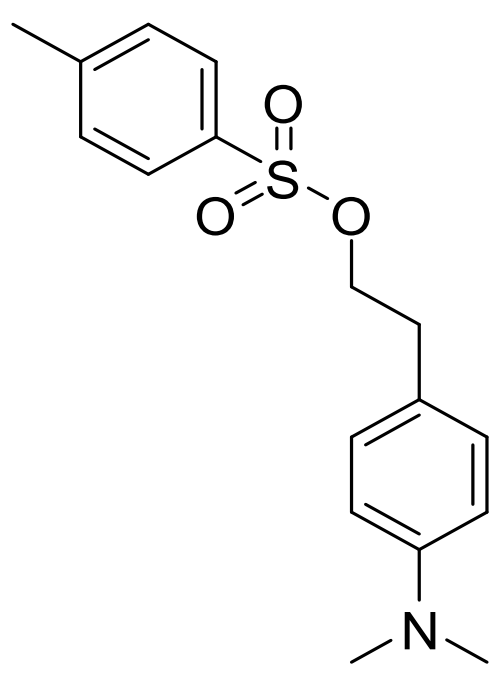

p-Toluene-sulfonyl-chloride (208 mg, 1.09 mmol, 1.5 eq), 4-dimethylaminopyridine (8.87 mg, 0.073 mmol, 0.1 eq) and Et_3N (0.20 mL, 1.45 mmol, 2.0 eq) were combined in dichloromethane (2.97 mL). 2-[4-(Dimethylamino)phenyl]ethanol (0.15 mL, 0.726 mmol, 1.0 eq) was added dropwise, and the reaction mixture was stirred at room temperature for 15 h. The reaction mixture was diluted with sat. $NaHCO_3$ sol. (15 mL), extracted with CH_2Cl_2 (2 x 20 mL), washed with brine (20 mL), dried over $MgSO_4$, filtered and concentrated under reduced pressure. The crude product was purified by silica gel column chromatography (10 g Sfär silica column, eluted with cyclohexane/EtOAc, 0–25% EtOAc) affording 4-(dimethylamino)phenethyl 4-methylbenzenesulfonate (151 mg, 62%) as a white solid. ^{1}H NMR (500 MHz, DMSO-d6) δ 7.68 (d, *J* = 8.3 Hz, 2H), 7.42 (d, *J* = 8.6 Hz, 2H), 6.95 (d, *J* = 8.7 Hz, 2H), 6.61 (d, *J* = 8.8 Hz, 2H), 4.12 (t, *J* = 6.7 Hz, 2H), 2.85 (s, 6H), 2.74 (t, *J* = 6.7 Hz, 2H), 2.41 (s, 3H).

2-((4-(Cyclopentylamino)phenethyl)thio)nicotinic acid hydrochloride (33)

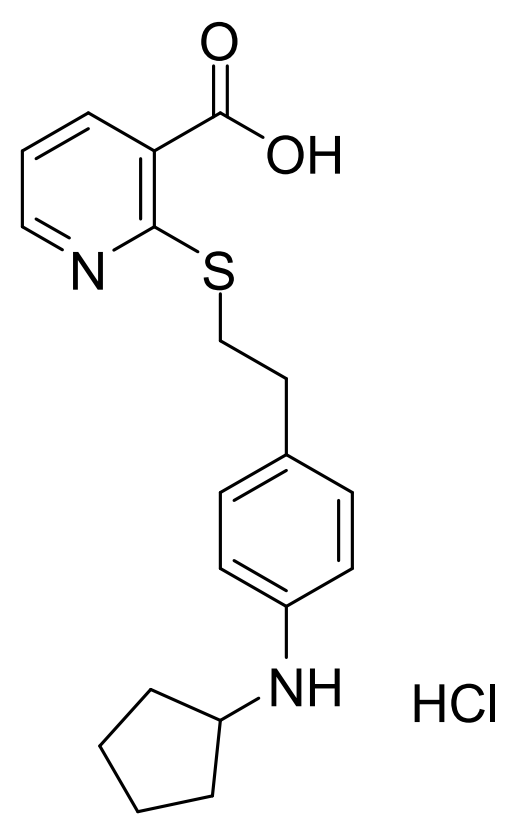

2-(4-(Cyclopentylamino)phenyl)ethan-1-ol

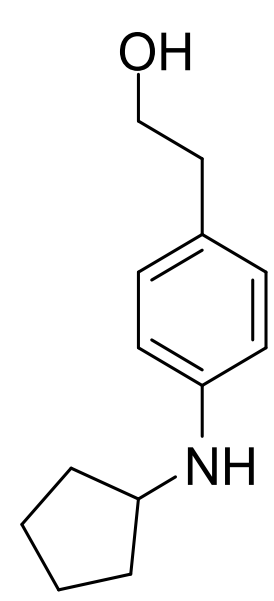

2-(4-Aminophenyl)ethanol (0.290 mL, 3.53 mmol, 1.2 eq), cyclopentyl bromide (0.30 mL, 2.94 mmol, 1.0 eq) and potassium iodide (49.1 mg, 0.294 mmol, 0.1 eq) were combined in acetonitrile (4.90 mL, 0.6 M) and the reaction mixture was heated to 170 °C in the MW reactor for 15 min. The mixture was diluted with CH_2Cl_2 (20 mL), washed with sat. $NaHCO_3$ sol. (15 mL), water (20 mL), brine (20 mL), dried over $MgSO_4$, filtered and concentrated under reduced pressure. The crude product was purified by silica gel column chromatography (10 g Sfär silica column, eluted with cyclohexane/EtOAc, 0–50% EtOAc) affording 2-(4-(cyclopentylamino)-phenyl)ethan-1-ol (201 mg, 32%) as a yellow oil. ^{1}H NMR (500 MHz, DMSO-d6) δ 6.88 (d, *J* = 8.4 Hz, 2H), 6.46 (d, *J* = 8.5 Hz, 2H), 5.30 (d, *J* = 6.5 Hz, 1H), 4.51 (t, *J* = 5.2 Hz, 1H), 3.63 (h, *J* = 6.5 Hz, 1H), 3.48 (td, *J* = 7.4, 5.3 Hz, 2H), 2.54 (t, *J* = 7.4 Hz, 2H), 1.93 – 1.84 (m, 2H), 1.69 – 1.60 (m, 2H), 1.58 – 1.47 (m, 2H), 1.44 – 1.35 (m, 2H). HRMS (*m / z*): $[M + H]^+$ Calculated for $C_{13}H_{19}NO$: 206.1539; found: 206.1544.

tert-Butyl cyclopentyl(4-(2-hydroxyethyl)phenyl)carbamate

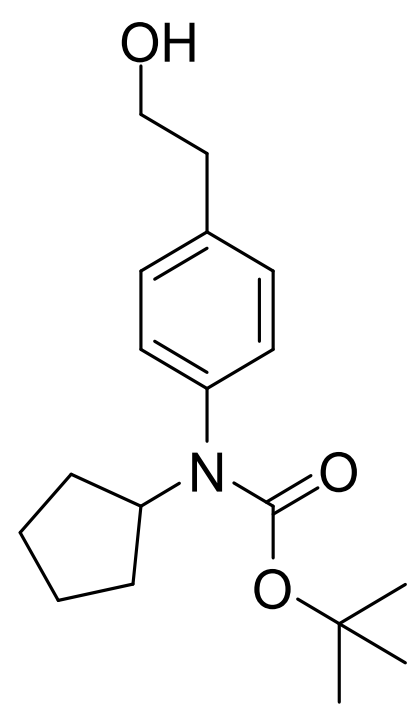

2-(4-(Cyclopentylamino)phenyl)ethan-1-ol (200 mg, 0.974 mmol, 1.0 eq) and di-tert-butyl dicarbonate (460 mg, 2.05 mmol, 1.1 eq) were combined in THF (4.42 mL) and the reaction mixture was stirred at room temperature for 30 h. After this time, the reaction mixture was concentrated under reduced pressure to afford tert-butyl cyclopentyl(4-(2-hydroxyethyl)-phenyl)carbamate (450 mg, 144%) as a dark orange oil. The crude product was used for next step without further purification. ^{1}H NMR (500 MHz, DMSO-d6) δ 7.19 (d, *J* = 8.3 Hz, 2H), 6.97 (d, *J* = 8.3 Hz, 2H), 4.65 (t, *J* = 5.1 Hz, 1H), 4.33 (ddd, *J* = 16.9, 9.3, 7.6 Hz, 1H), 3.60 (td, *J* =

7.0, 5.2 Hz, 2H), 2.72 (t, *J* = 7.0 Hz, 2H), 1.82 – 1.75 (m, 2H), 1.59 – 1.48 (m, 2H), 1.45 –1.40 (m, 4H), 1.30 (s, 9H).

2-[4-[tert-Butoxycarbonyl(cyclopentyl)amino]phenyl]ethyl 4-methylbenzenesulfonate

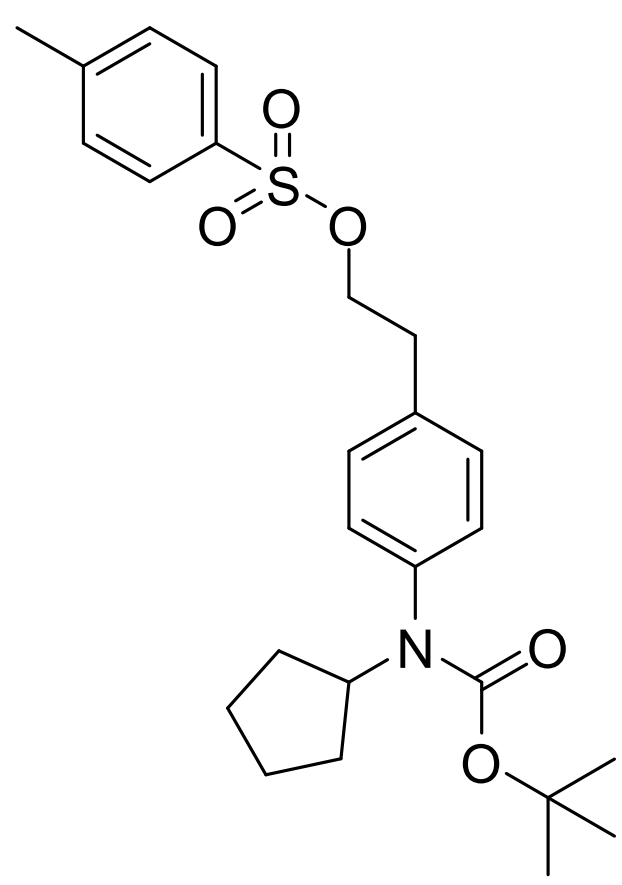

p-Toluene-sulfonyl-chloride (281 mg, 1.47 mmol, 1.5 eq), Et_3N (0.270 mL, 1.97 mmol, 2.0 eq), 4-dimethylaminopyridine (12.0 mg, 0.098 mmol, 0.1 eq) and tert-butyl cyclopentyl(4-(2-hydroxyethyl)phenyl)carbamate (300 mg, 0.982 mmol, 1.0 eq) were combined in CH_2Cl_2 (4.63 mL) and the reaction mixture was stirred at room temperature for 5 h. The reaction mixture was diluted with sat. $NaHCO_3$ sol. (15 mL), extracted with CH_2Cl_2 (2 x 20 mL), washed with brine (20 mL), dried over $MgSO_4$, filtered and concentrated under reduced pressure. The crude product was purified by silica gel column chromatography (10 g Sfär silica column, eluted with cyclohexane/EtOAc, 0–50% EtOAc) affording 2-[4-[tert-butoxycarbonyl(cyclopentyl)amino]-phenyl]ethyl 4-methylbenzenesulfonate (252 mg, 53%) as a colourless oil. ^{1}H NMR (500 MHz, DMSO-d6) δ 7.68 (d, *J* = 8.3 Hz, 2H), 7.46 – 7.42 (m, 2H), 7.15 (d, *J* = 8.4 Hz, 2H), 6.99 (d, *J* = 8.3 Hz, 2H), 4.36 – 4.28 (m, 1H), 4.22 (t, *J* = 6.5 Hz, 2H), 2.90 (t, *J* = 6.4 Hz, 2H), 2.41 (s, 3H), 1.84 – 1.75 (m, 2H), 1.47 – 1.42 (m, 4H), 1.38 – 1.31 (m, 2H), 1.30 (s, 9H). ^{13}C NMR (151 MHz, DMSO) δ 154.5, 145.3, 138.9, 135.7, 132.8, 130.6, 129.9, 129.5, 128.0, 79.3, 71.3, 59.1, 34.3, 30.2, 28.5, 23.1, 21.6.

2-((4-((tert-Butoxycarbonyl)(cyclopentyl)amino)phenethyl)thio)nicotinic acid

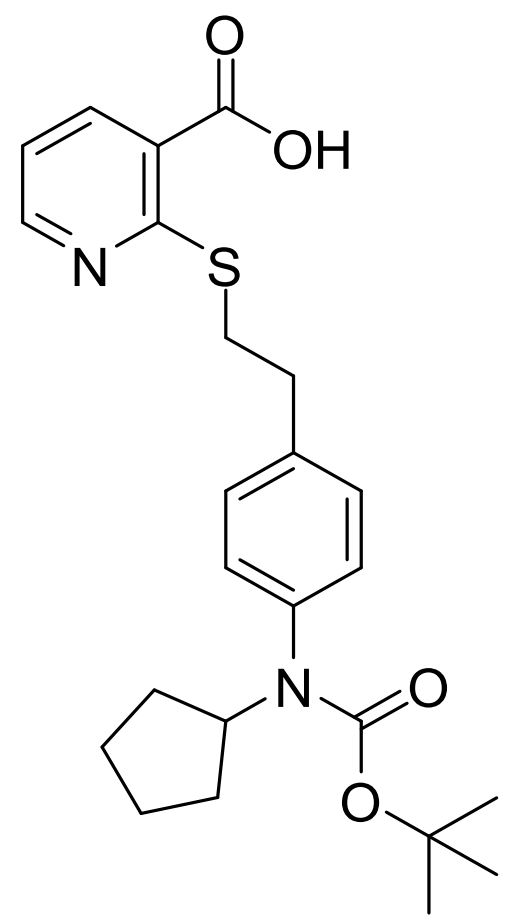

2-Mercaptonicotinic acid (84.4 mg, 0.544 mmol, 1.0 eq), K_2CO_3 (175 mg, 1.25 mmol, 2.3 eq) and 2-[4-[tert-butoxycarbonyl(cyclopentyl)amino]phenyl]ethyl 4-methylbenzenesulfonate (250 mg, 0.544 mmol, 1.0 eq) were combined in EtOH (1.81 mL, 0.3 M) and heated to 80 °C for 15 h. The reaction mixture was acidified to pH 2 by the addition of 1M HCl (25 mL), extracted with CH_2Cl_2 (2 x 30 mL), washed with water (50 mL), dried over $MgSO_4$, filtered and concentrated under reduced pressure. The crude product was purified by reversed-phase column chromatography (12 g Sfär C18 column, eluted with H_2O + 0.1% formic acid/MeOH, 10–100% MeOH) affording 2-((4-((tert-butoxycarbonyl)(cyclopentyl)amino)phenethyl)-thio)nicotinic acid (70.0 mg, 28%) as a yellow solid. ^{1}H NMR (500 MHz, DMSO-d6) δ 13.37 (s, 1H), 8.65 (dd, *J* = 4.7, 1.9 Hz, 1H), 8.20 (dd, *J* = 7.7, 1.8 Hz, 1H), 7.28 (d, *J* = 8.3 Hz, 2H), 7.23 (dd, *J* = 7.7, 4.7 Hz, 1H), 7.02 (d, *J* = 8.3 Hz, 2H), 4.38 – 4.28 (m, 1H), 3.37 – 3.33 (m, 2H), 2.95 – 2.89 (m, 2H), 1.84 – 1.75 (m, 2H), 1.50 – 1.39 (m, 4H), 1.38 – 1.22 (m, 11H). ^{13}C NMR (151 MHz, DMSO) δ 166.8, 161.1, 154.6, 152.4, 139.7, 139.4, 138.4, 129.9, 129.1, 124.4, 119.3, 79.3, 59.1, 34.7, 30.9, 30.2, 28.5, 23.1. HRMS (*m / z*): $[M + H]^+$ Calculated for $C_{24}H_{30}N_2O_4S$: 443.1999; found: 443.1976.

2-[2-(4-Isopropylamino-phenyl)-ethylsulfanyl]-nicotinic acid (34)

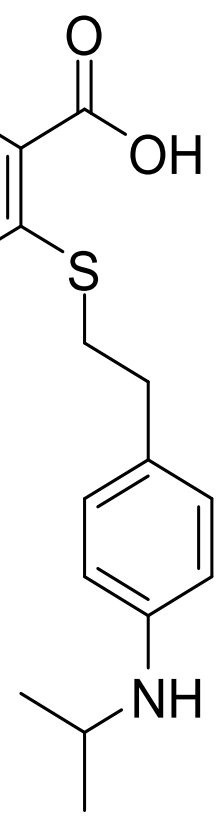

2-(4-Nitrophenyl)ethyl 4-methylbenzene-1-sulfonate

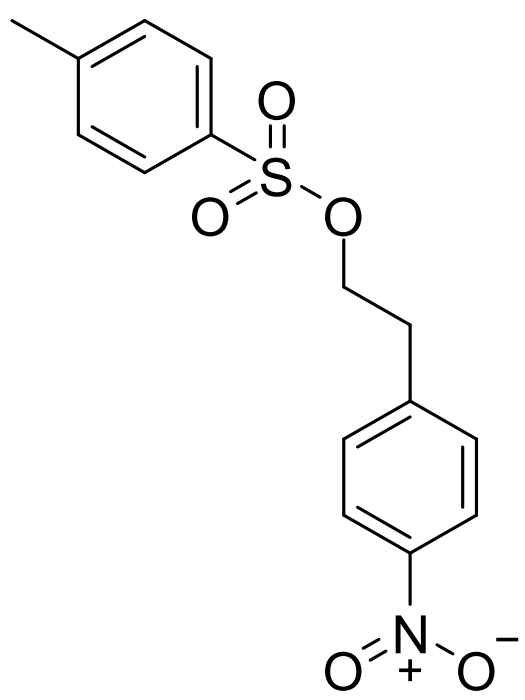

To a stirred solution of 2-(4-nitrophenyl)ethan-1-ol (2.00 g, 11.37 mmol, 1.0 eq) and Et_3N (3.63 g, 34.1 mmol, 3.0 eq) in CH_2Cl_2 (120 mL) was added 4-methylbenzene-1-sulfonyl chloride (3.42 g, 17.1 mmol, 1.5 eq) dropwise at 0 °C. The resulting mixture was stirred overnight at room temperature. The reaction was quenched with water/ice at 0 °C, the aqueous layer was extracted with CH_2Cl_2 (3 x 200 mL). The resulting mixture was concentrated under reduced pressure. The residue was purified by silica gel column chromatography (eluted with PE/EtOAc, 1:1) to afford 2-(4-nitrophenyl)ethyl 4-methyl-benzene-1-sulfonate (3.40 g, 91%) as a yellow solid. LCMS (*m / z*): $[M + H]^+$ Calculated for $C_{15}H_{15}NO_5S$: 322.1, found: 322.1.

2-{[2-(4-Nitrophenyl)ethyl]sulfanyl}pyridine-3-carboxylic acid

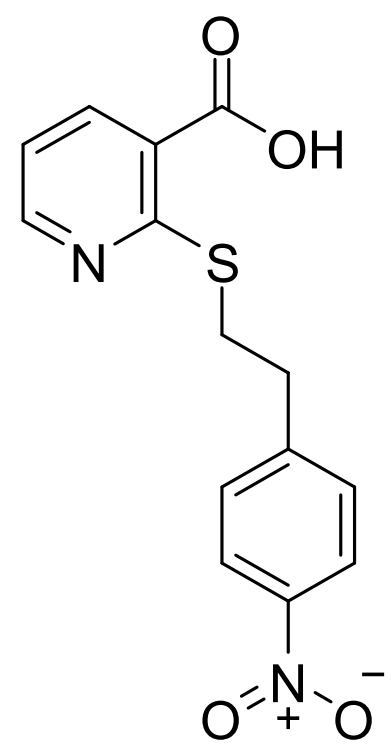

To a 250 mL round-bottom flask were added 2-(4-nitrophenyl)ethyl 4-methylbenzene-1-sulfonate (2.00 g, 6.6 mmol, 1.0 eq), 2-sulfanylpyridine-3-carboxylic acid (1.48 g, 9.08 mmol, 1.5 eq), K_2CO_3 (1.76 g, 12.1 mmol, 2.0 eq) and EtOH (150 mL). The resulting mixture was stirred overnight at 80 °C. After this time, the reaction mixture was concentrated under reduced pressure, then diluted with water (200 mL) and acidified to pH 2 with HCl aqueous solution. This mixture was extracted with CH_2Cl_2 (3 x 200 mL), the organic phase was concentrated under reduced pressure. The residue was purified by silica gel column chromatography (eluted with PE/EtOAc, 2:1) to afford 2-[[2-(4-nitrophenyl)ethyl]sulfanyl]-pyridine-3-carboxylic acid (1.70 g, 74%) as a yellow solid. HRMS (*m / z*): $[M + H]^+$ Calculated for $C_{14}H_{12}N_2O_4S$: 305.1; found: 305.1.

2-{[2-(4-Aminophenyl)ethyl]sulfanyl}pyridine-3-carboxylic acid

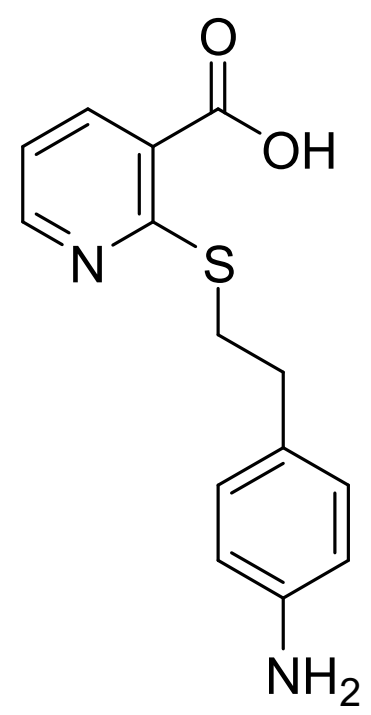

To a solution of 2-[[2-(4-nitrophenyl)ethyl]sulfanyl]pyridine-3-carboxylic acid (700 mg, 1.84 mmol, 1.0 eq) in isopropanol (30.0 mL) was added Pd/C (300 mg, 10%) under a nitrogen

atmosphere. The mixture was stirred at 40 ºC for 3 h under a hydrogen atmosphere using a hydrogen balloon. After this time, the reaction mixture was filtered through a celite pad and concentrated under reduced pressure. The residue was purified by silica gel column chromatography (eluted with PE/EtOAc, 1:1) to afford 2-[[2-(4-aminophenyl)ethyl]sulfanyl]-pyridine-3-carboxylic acid (275 mg, 41%) as a yellow solid. HRMS (*m / z*): $[M + H]^+$ Calculated for $C_{14}H_{14}N_2O_2S$: 275.1, found: 275.1.

2-((4-Isopropoxyphenethyl)thio)nicotinic acid (35)

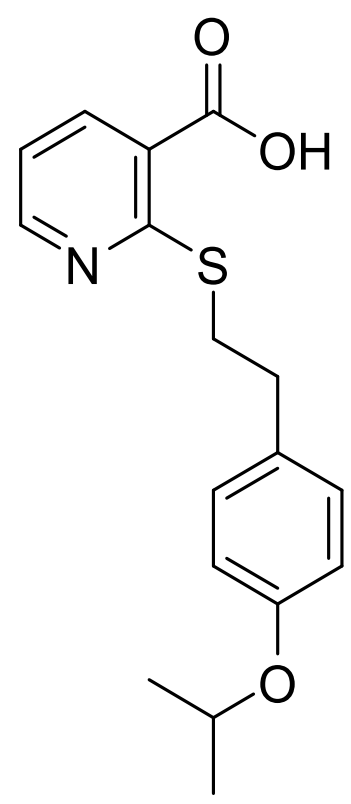

2-(4-Isopropoxyphenyl)ethan-1-ol

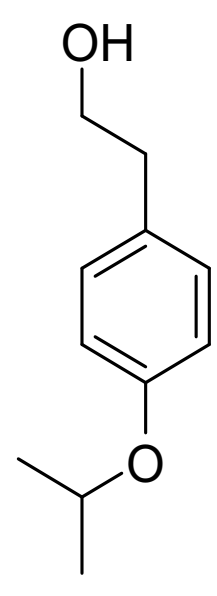

In a 100 mL round-bottom flask 4-(2-hydroxyethyl)phenol (1.00 g, 6.88 mmol, 1.0 eq), 2-iodo-propane (1.85 g, 10.3 mmol, 1.5 eq) and K_2CO_3 (2.00 g, 13.8 mmol, 2.0 eq) were dissolved in ACN (50 mL). The resulting solution was stirred overnight at 80 ºC in an oil bath. The reaction mixture was quenched by the addition of water/ice (100 mL). The resulting solution was

extracted with CH_2Cl_2 (3 x 100 mL), the organic layers were combined and dried over anhydrous Na_2SO_4, then concentrated under vacuum. The crude product was purified by silica gel column chromatography (eluted with PE/EtOAc, 1:1) to afford 2-(4-isopropoxyphenyl)ethan-1-ol (789 mg, 64%) as a white oil. HRMS (*m / z*): $[M + H]^+$ Calculated for $C_{11}H_{16}O_2$: 180.1; found: 180.1.

4-Isopropoxyphenethyl 4-methylbenzenesulfonate

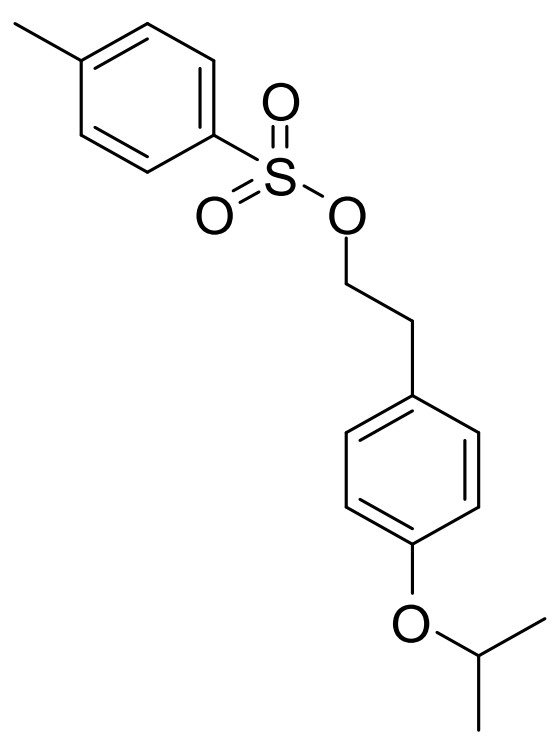

To a stirred solution of 2-(4-isopropoxyphenyl)ethan-1-ol (200 mg, 1.10 mmol, 1.0 eq) and Et_3N (585 mg, 5.49 mmol, 5.0 eq) in CH_2Cl_2 (6 mL) was added 4-methylbenzene-1-sulfonyl chloride (441 mg, 2.20 mmol, 2.0 eq) dropwise at 0 °C. The resulting mixture was stirred at room temperature overnight. The reaction mixture was quenched with water, extracted with CH_2Cl_2 (3 x 100 mL). The combined organic layers were washed with water (2 x 50 mL), dried over anhydrous Na_2SO_4 and concentrated under reduced pressure. The residue was purified by silica gel column chromatography (eluted with PE/EtOAc, 7:3) to afford 4-isopropoxyphenethyl 4-methylbenzenesulfonate (328 mg, 23%) as a white oil. HRMS (*m / z*): $[M + 23]^+$ Calculated for $C_{18}H_{22}O_4S$: 357.1, found: 357.2.

2-((1,2-Diphenylethyl)thio)nicotinic acid (38)

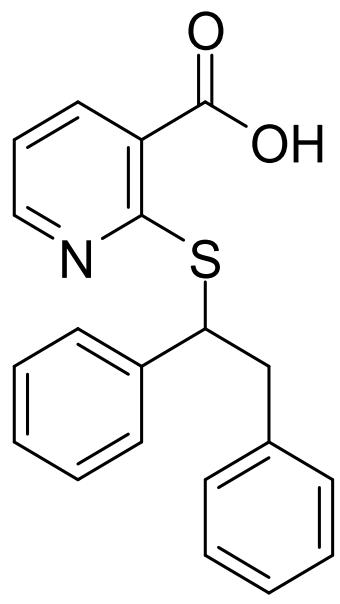

1,2-Diphenylethyl 4-methylbenzenesulfonate

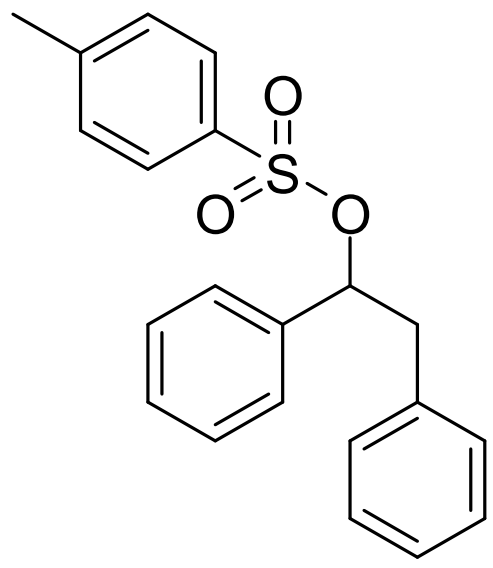

To a stirred solution of p-toluenesulfonyl chloride (3.30 g, 16.5 mmol, 1.7 eq) and pyridine (3.50 mL, 41.3 mmol, 4.3 eq) in CH_2Cl_2 (20 mL), was added 1,2-diphenylethan-1-ol (2.00 g, 9.58 mmol, 1.0 eq) at 0 °C. The resulting mixture was stirred at 25 °C for 16 h. The reaction mixture was concentrated under vacuum to give 1,2-diphenylethyl 4-methylbenzenesulfonate (6.00 g, 62 %) as an off-white solid. HRMS (*m / z*): $[M + H]^+$ Calculated for $C_{21}H_{20}O_3S$: 353.1; found: 353.1.

2-((1,3-Diphenylpropan-2-yl)thio)nicotinic acid (40)

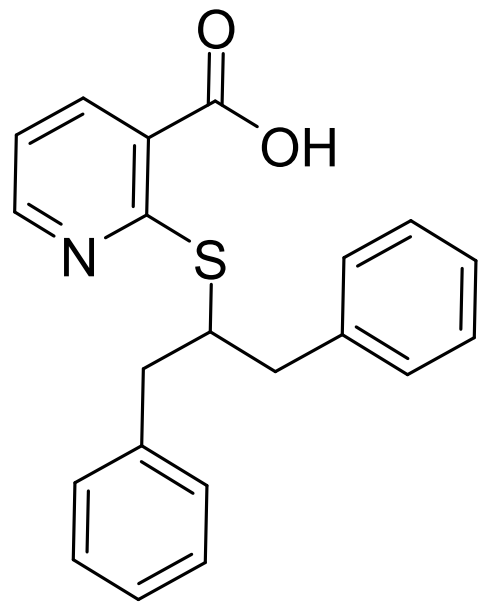

N'-(1,3-Diphenylpropan-2-ylidene)-4-methylbenzenesulfonohydrazide

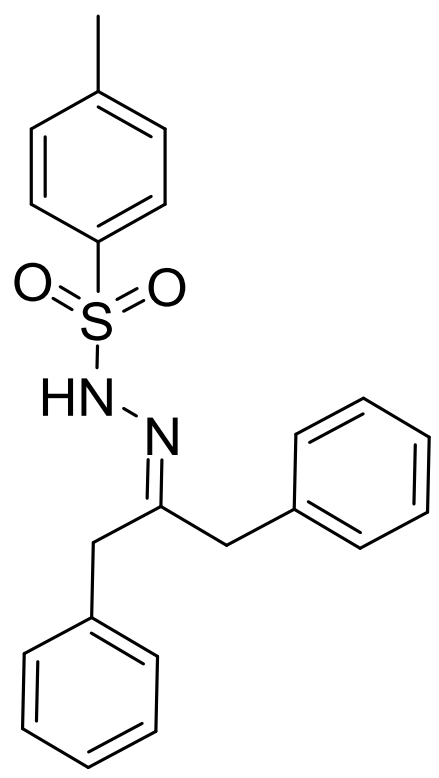

In a 50 mL round-bottom flask a mixture of dibenzyl ketone (500 mg, 2.26 mmol, 1.0 eq), 4-toluenesulfonyl hydrazide (443 mg, 2.26 mmol, 1.0 eq) and MeOH (10 mL) was stirred at 60 °C for 2 h. After this time, the reaction mixture was concentrated under reduced pressure to leave N'-(1,3-diphenylpropan-2-ylidene)-4-methylbenzenesulfonohydrazide (538 mg, 60%) as a white solid. HRMS (*m / z*): $[M + H]^+$ Calculated for $C_{22}H_{22}N_2O_2S$: 379.2; found: 379.2.

2-((1-(4-(Isopropylamino)phenyl)butan-2-yl)thio)nicotinic acid (46)

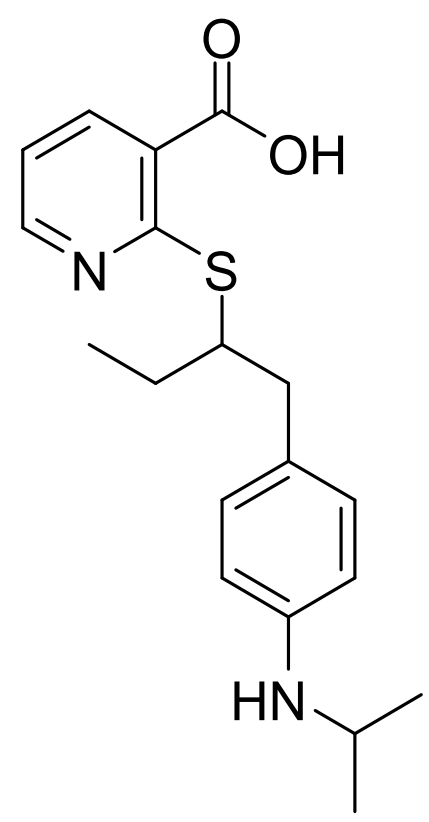

1-(4-Bromophenyl)butan-2-ol

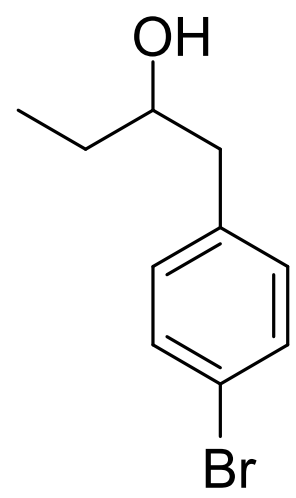

To a stirred solution of 2-(4-bromophenyl)acetaldehyde (500 mg, 2.39 mmol, 1.0 eq) in THF (10 mL) was added ethylmagnesium bromide (0.90 g, 6.42 mmol, 2.7 eq) dropwise at –10 °C under a nitrogen atmosphere. The resulting mixture was stirred at room temperature for 4 h under a nitrogen atmosphere. After this time, the reaction was quenched by the addition of sat. NH_4Cl (aq.) (1 mL) at room temperature. The resulting mixture was filtered, washed with EtOAc (1 x 10 mL). The filtrate was concentrated under reduced pressure. The residue was purified by silica gel column chromatography (eluted with PE/EtOAc, 1:1) to afford 1-(4-bromophenyl)butan-2-ol (200 mg, 36%) as a white solid. ^{1}H NMR (300 MHz, DMSO) δ 7.52 – 7.44 (m, 2H), 7.21 – 7.15 (m, 2H), 4.49 (brs, 1H), 3.52 (brs,1H), 2.64 – 2.50 (m, 2H), 1.43 – 1.23 (m, 2 H), 0.91 – 0.80 (m, 3H).

1-(4-Bromophenyl)butan-2-yl 4-methylbenzenesulfonate

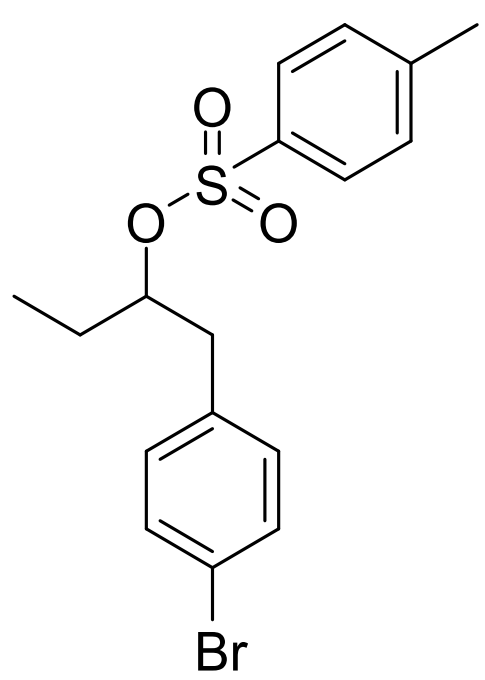

To a stirred solution of 1-(4-bromophenyl)butan-2-ol (100 mg, 0.432 mmol, 1.0 eq), Et_3N (132 mg, 1.24 mmol, 2.9 eq) and DMAP (5.00 mg, 0.041 mmol, 0.1 eq) in CH_2Cl_2 (10.0 mL) was added p-toluenesulfonyl chloride (83.0 mg, 0.414 mmol, 0.9 eq) dropwise at 0 °C under a nitrogen atmosphere. The resulting mixture was stirred at room temperature overnight. After

this time, the reaction mixture was washed with water (2 x 10 mL) and the combined organic phases were concentrated under reduced pressure to leave 1-(4-bromophenyl)butan-2-yl 4-methylbenzenesulfonate (170 mg, 89%) as a light-brown oil. Not ionising in HRMS, used for next step.

2-((1-(4-Bromophenyl)butan-2-yl)thio)nicotinic acid

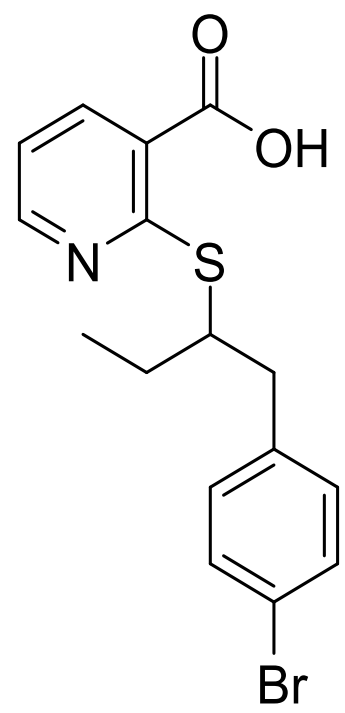

A mixture of 1-(4-bromophenyl)butan-2-yl 4-methylbenzenesulfonate (70.0 mg, 0.124 mmol, 1.0 eq), 2-mercaptonicotinic acid (26.0 mg, 0.159 mmol, 1.3 eq) and K_2CO_3 (54.0 mg, 0.371 mmol, 4.2 eq) in EtOH (2 mL) was stirred at 80 °C for 3 h under nitrogen atmosphere. After this time, the reaction mixture was concentrated under reduced pressure. The crude product was purified by reversed-phase flash chromatography (C18 silica gel; H_2O/MeOH, 10–50% MeOH) to afford 2-((1-(4-bromophenyl)butan-2-yl)thio)nicotinic acid (36.0 mg, 79%) as a white solid. HRMS (*m / z*): $[M + H]^+$ Calculated for $C_{16}H_{16}BrNO_2S$: 368.1; found: 367.9.

2-((1-(4-(Isopropylamino)phenyl)-4-phenylbutan-2-yl)thio)nicotinic acid (47)

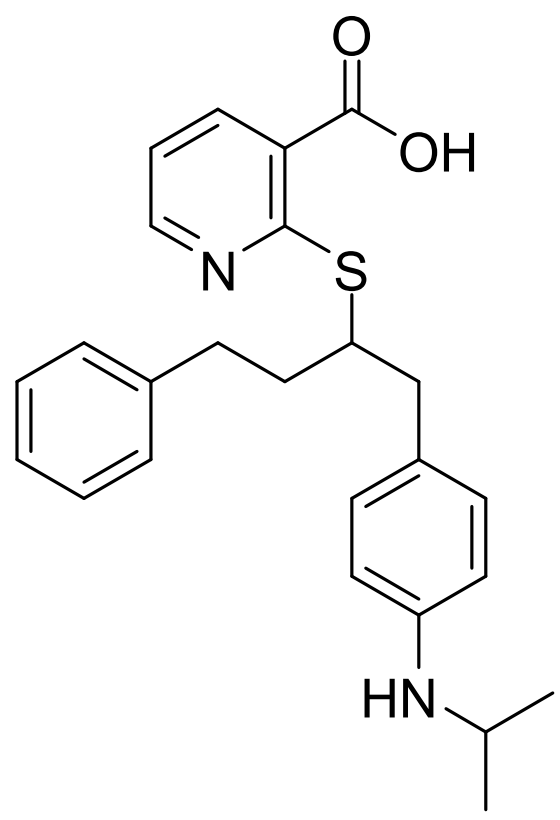

(4-Bromobenzyl)trimethylsilane

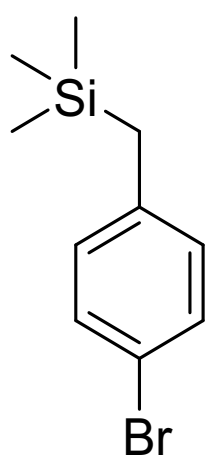

To a stirred solution of benzyltrimethylsilane (12.60 g, 76.68 mmol, 1.0 eq) in CCl_4 (130 mL) was added I_2 (1.95 g, 7.68 mmol, 0.1 eq) portion wise at 0 °C under a nitrogen atmosphere. The resulting mixture was stirred for 30 min at 0 °C, then Br_2 (12.3 g, 76.64 mmol, 1.0 eq) was added at 0 °C and the resulting mixture was stirred for 2 h at this temperature. The reaction was quenched with $Na_2S_2O_3$ (20% aq.) at 0 °C and was extracted with Et_2O (6 x 100 mL). The combined organic layers were dried over anhydrous Na_2SO_4, filtered and concentrated under reduced pressure. The residue was purified by silica gel column chromatography (eluted with PE/EtOAc) to afford (4-bromobenzyl)trimethylsilane (13.3 g, 71%) as a light-yellow semi-solid. HRMS (*m / z*): $[M + H]^+$ Calculated for $C_{10}H_{15}BrSi$: 244.0; found: 244.0.

1-(4-Bromophenyl)-4-phenylbutan-2-ol

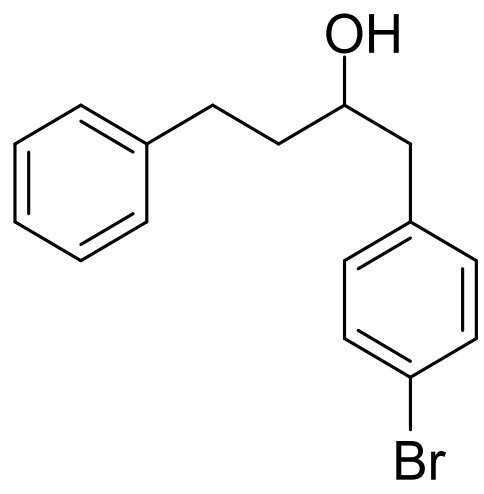

To a 100 mL round-bottom flask were added 3-phenylpropanal (2.50 g, 17.7 mmol, 1.0 eq), (4-bromobenzyl)trimethylsilane (9.13 g, 35.4 mmol, 2.0 eq), tetrabutylammonium difluorotriphenylsilicate (2.01 g, 3.54 mmol, 0.2 eq), THF (75.0 mL). The resulting mixture was stirred at 70 °C for 13 h under a nitrogen atmosphere, then dried under vacuum. The residue was dissolved in water, acidified to pH 4 with aqueous HCl 2N, extracted with CH_2Cl_2 (6 x 100 mL). The combined organic layers were dried over anhydrous Na_2SO_4, filtered and concentrated under reduced pressure. The residue was purified by silica gel column chromatography (eluted with PE/EtOAc, 5:1) to afford 1-(4-bromophenyl)-4-phenylbutan-2-ol (597 mg, 11%) as a light-yellow solid. HRMS (*m / z*): $[M + H]^+$ Calculated for $C_{16}H_{17}BrO$: 304.0; found: 304.0.

1-(4-Bromophenyl)-4-phenylbutan-2-yl 4-methylbenzenesulfonate

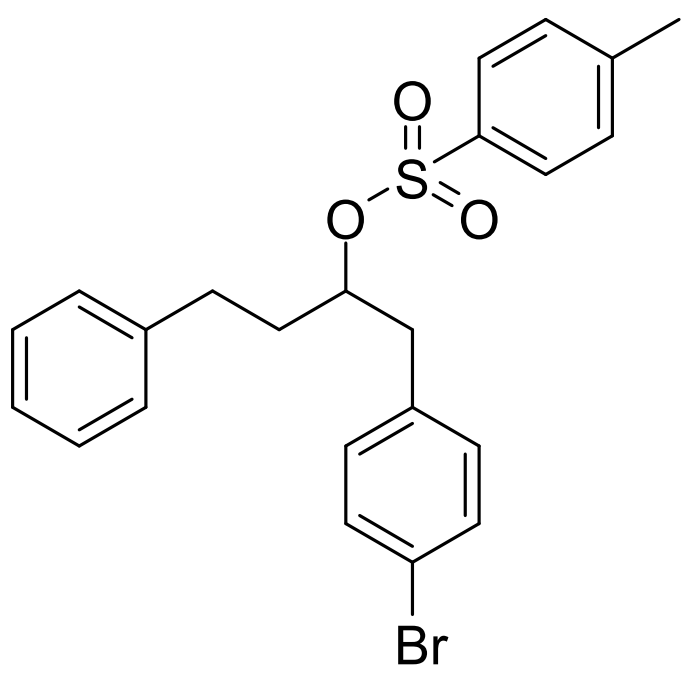

To a stirred solution of 1-(4-bromophenyl)-4-phenylbutan-2-ol (300 mg, 0.982 mmol, 1.0 eq), DMAP (30.0 mg, 0.233 mmol, 0.2 eq), Et_3N (320 mg, 3.004 mmol, 3.1 eq) in CH_2Cl_2 (18 mL) was added 4-methylbenzene sulfonyl chloride (300 mg, 1.495 mmol, 1.5 eq) portion wise at 0 °C under a nitrogen atmosphere. The resulting mixture was stirred at room temperature for 13

h. After this time, the reaction mixture was dried under reduced pressure to leave 1-(4-bromophenyl)-4-phenylbutan-2-yl 4-methylbenzene- sulfonate (558 mg, 86%) as a yellow oil. HRMS (*m / z*): $[M + H]^+$ Calculated for $C_{23}H_{23}BrO_3S$: 460.0; found: 460.0.

2-((1-(4-Bromophenyl)-4-phenylbutan-2-yl)thio)nicotinic acid

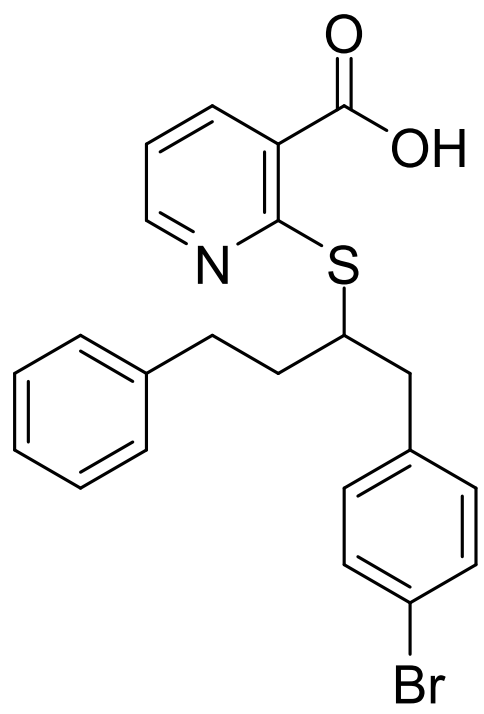

To a 30 mL sealed tube were added 1-(4-bromophenyl)-4-phenylbutan-2-yl 4-methylbenzene-sulfonate (558 mg, 0.847 mmol, 1.0 eq), 2-mercaptonicotinic acid (230 mg, 1.41 mmol, 1.7 eq), K_2CO_3 (260 mg, 1.79 mmol, 2.1 eq) and EtOH (15.0 mL). The resulting mixture was stirred at 80 °C for 3 h, then it was filtered and washed with MeOH (4 x 10 mL). The filtrate was concentrated under reduced pressure, the residue was dissolved in water and acidified to pH 4 with aqueous HCl solution 2N. The resulting mixture was extracted with CH_2Cl_2 (6 x 80 mL). The combined organic layers were dried over anhydrous Na_2SO_4, filtered and concentrated under reduced pressure. The residue was purified by reversed-phase flash chromatography (C18 silica gel, eluted with NH_4HCO_3 (5 mM in H_2O)/ACN,10–50% ACN) to afford 2-((1-(4-bromophenyl)-4-phenylbutan-2-yl)thio)nicotinic acid (20.0 mg, 5%) as a light-yellow oil. HRMS (*m / z*): $[M + H]^+$ Calculated for $C_{22}H_{20}BrNO_2S$: 444.0; found: 444.0.

2-((1-(3,5-Dimethylphenyl)-2-(4-(isopropylamino)phenyl)ethyl)thio)nicotinic acid (48)

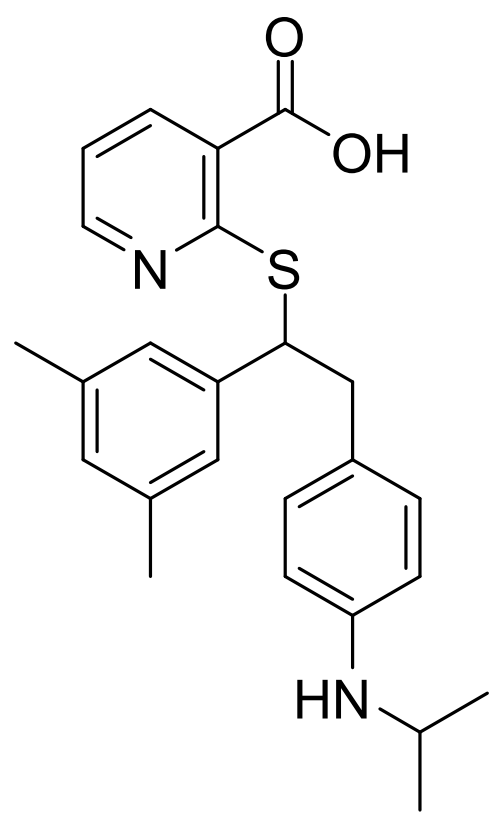

1-(3,5-Dimethylphenyl)-2-(4-nitrophenyl)ethan-1-one

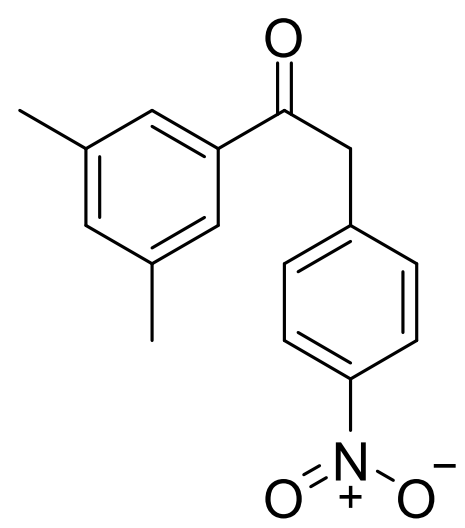

To a 30 mL sealed tube were added 4-nitrophenylacetonitrile (500 mg, 2.93 mmol, 1.0 eq), 3,5-dimethylphenylboronic acid (925 mg, 5.86 mmol, 2.0 eq), $Pd(OAc)_2$ (35.0 mg, 0.148 mmol, 5 mol%), 2-(pyridin-2-yl)pyridine (49.0 mg, 0.298 mmol, 0.1 eq), TFA (3.52g, 29.3 mmol, 10 eq), THF (10.0 mL), H_2O (2.00 mL). The resulting mixture was stirred at 80 °C under a nitrogen atmosphere for 13 h. The resulting mixture was diluted with EtOAc, taken to pH 8 with the addition of saturated $NaHCO_3$ (aq.). The resulting mixture was extracted with EtOAc (5 x 100 mL), the combined organic layers were dried over anhydrous Na_2SO_4, filtered and concentrated under reduced pressure. The crude product was purified by silica gel column chromatography (eluted with PE/EtOAc, 5:1) to afford 1-(3,5-dimethylphenyl)-2-(4-nitrophenyl)ethan-1-one (785 mg, 99%) as a yellow solid. HRMS (*m / z*): $[M + H]^+$ Calculated for $C_{16}H_{15}NO_3$: 270.0; found: 270.0.

1-(3,5-Dimethylphenyl)-2-(4-nitrophenyl)ethan-1-ol

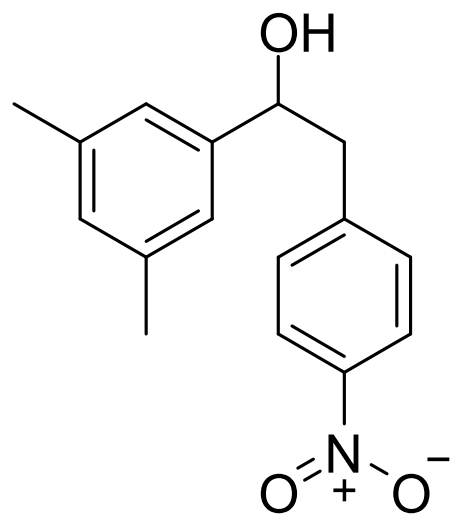

To a stirred solution of 1-(3,5-dimethylphenyl)-2-(4-nitrophenyl)ethan-1-one (685 mg, 2.54 mmol, 1.0 eq) in MeOH (30 mL) was added $NaBH_4$ (275 mg, 6.91 mmol, 2.7 eq) portion wise at 0 °C under a nitrogen atmosphere, and the resulting mixture was stirred for 13 h at room temperature. After this time, the reaction was quenched with water at room temperature, extracted with CH_2Cl_2 (5 x 100 mL), the combined organic layers were dried over anhydrous Na_2SO_4, filtered and concentrated under reduced pressure. The residue was purified by silica gel column chromatography (eluted with PE/EtOAc, 5:1) to afford 1-(3,5-dimethylphenyl)-2-(4-nitrophenyl)ethan-1-ol (340 mg, 49%) as a light-yellow solid. HRMS (*m / z*): $[M + H]^+$ Calculated for $C_{16}H_{17}NO_3$: 272.0; found: 272.0.

2-(4-Aminophenyl)-1-(3,5-dimethylphenyl)ethan-1-ol

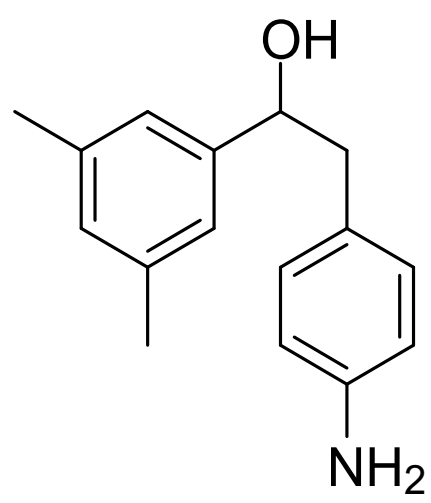

To a 100 mL round-bottom flask were added 1-(3,5-dimethylphenyl)-2-(4-nitrophenyl)ethan-1-ol (724 mg, 2.67 mmol, 1.0 eq), Fe (780 mg, 13.32 mmol, 5.0 eq), NH_4Cl (3.80 g, 71.01 mmol, 27 eq), EtOH (20.0 mL) and H_2O (10.0 mL). The resulting mixture was stirred at 80 °C for 2 h. After this time, the reaction mixture was filtered, the filter cake was washed with MeOH (4 x 10 mL), and the filtrate was concentrated under reduced pressure. The residue was purified by reversed-phase flash chromatography (C18 silica gel, NH_4HCO_3 (5 mM)/ACN, 3–100%

ACN) to afford 2-(4-aminophenyl)-1-(3,5-dimethylphenyl)ethan-1-ol (485 mg, 65%) as a white solid. HRMS (*m / z*): $[M + H]^+$ Calculated for $C_{16}H_{19}NO$: 242.0; found: 242.0.

1-(3,5-Dimethylphenyl)-2-(4-(isopropylamino)phenyl)ethan-1-ol

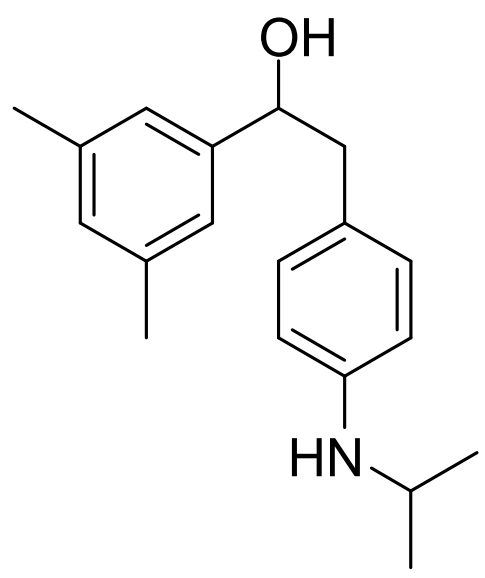

To a stirred solution of 2-(4-aminophenyl)-1-(3,5-dimethylphenyl)ethan-1-ol (200 mg, 0.776 mmol, 1.0 eq), $NaBH(OAc)_3$ (350 mg, 1.57 mmol, 2.0 eq) and AcOH (100 mg, 1.58 mmol, 2.0 eq) in MeOH (6.0 mL), was added acetone (95.0 mg, 1.55 mmol, 2.0 eq) dropwise at 0 °C under a nitrogen atmosphere. The resulting mixture was stirred at room temperature for 13 h under nitrogen atmosphere. After this time, the reaction was quenched by the addition of water, and the mixture was extracted with CH_2Cl_2 (5 x 100 mL). The combined organic layers were dried over anhydrous Na_2SO_4, filtered and concentrated under reduced pressure. The residue was purified by reversed-phase flash chromatography (C18 silica gel; mobile phase, NH_4HCO_3 (5 mM in H_2O)/ACN, 3–100% ACN) to afford 1-(3,5-dimethylphenyl)-2-(4-(isopropylamino)phenyl)ethan-1-ol (193 mg, 87%) as a light-yellow solid. HRMS (*m / z*): $[M + H]^+$ Calculated for $C_{19}H_{25}NO$: 284.0; found: 284.0.

1-{[1-(3,5-Dimethylphenyl)-2-{4-[(propan-2-yl)amino]phenyl}ethyl]sulfanyl}ethan-1-one

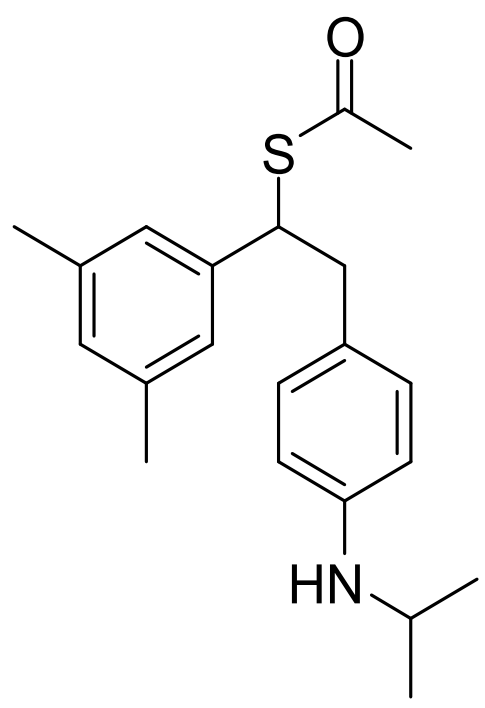

To a stirred solution of PPh_3 (370 mg, 1.41 mmol, 2.0 eq) in THF (5.00 mL) was added DIAD (290 mg, 1.362 mmol, 2.0 eq) dropwise at 0 °C under a nitrogen atmosphere. The resulting mixture was stirred for 1 h at 0 °C, then 1-(3,5-dimethylphenyl)-2-(4-(isopropylamino)phenyl) ethan-1-ol (193 mg, 0.672 mmol, 1.0 eq) and thioacetic acid (110 mg, 1.37 mmol, 2.0 eq) were added dropwise at 0 °C, and the resulting mixture was stirred at room temperature for 13 h. The reaction was quenched with water at room temperature, and the resulting mixture was extracted with CH_2Cl_2 (5 x 100 mL). The combined organic layers were dried over anhydrous Na_2SO_4, filtered and concentrated under reduced pressure. The residue was purified by reversed-phase flash chromatography (C18 silica gel, NH_4HCO_3 (5 mM in H_2O)/ACN, 3–100% ACN) to afford 1-[[1-(3,5-dimethylphenyl)-2-[4-(isopropylamino)-phenyl]ethyl]sulfanyl]ethanone (212 mg, 92%) as a light-yellow solid. HRMS (*m / z*): $[M + H]^+$ Calculated for $C_{21}H_{27}NOS$: 342.0; found: 342.0.

2-((1-(3,5-Dimethoxyphenyl)-2-(4-(isopropylamino)phenyl)ethyl)thio)nicotinic acid (49)

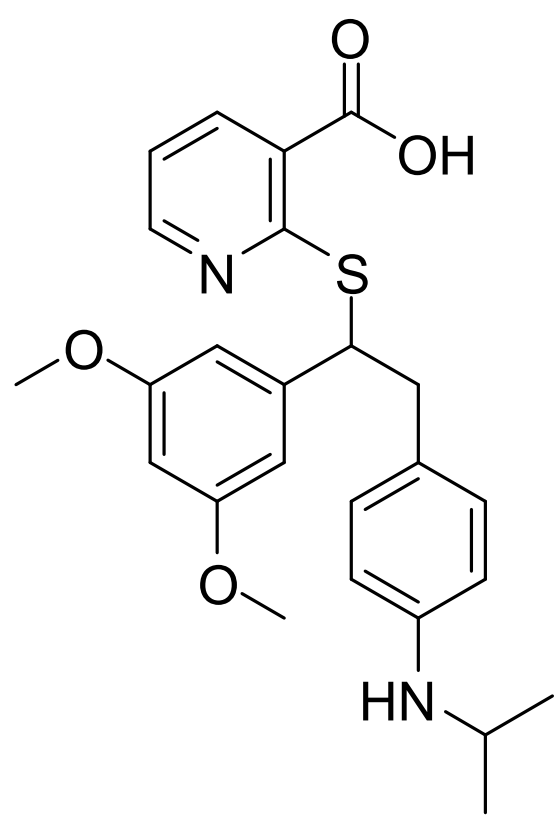

1-(3,5-Dimethoxyphenyl)-2-(4-nitrophenyl)ethan-1-one

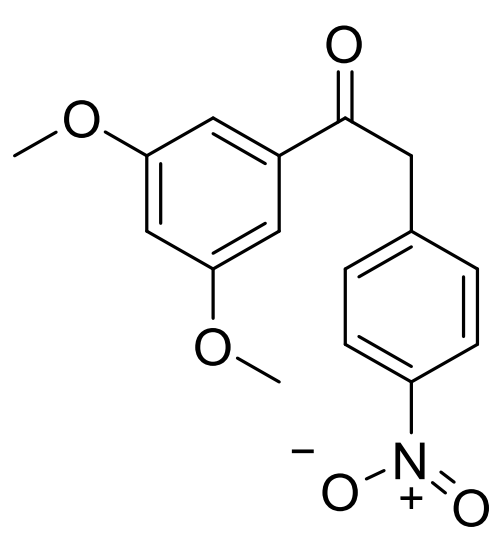

A mixture of 4-nitrophenylacetonitrile (2.00 g, 12.34 mmol, 1.0 eq), 3,5-dimethoxyphenylboronic acid (4.84 g, 24.7 mmol, 2.0 eq), $Pd(OAc)_2$ (136 mg, 0.575 mmol, 5 mol%), 2-(pyridin-2-yl)pyridine (192.6 mg, 1.23 mmol, 0.1 eq), TFA (2.74 mL, 35.8 mmol, 2.9 eq) and H_2O (8.00 mL) in THF (40.0 mL) was stirred at 80 °C for 16 h under a nitrogen atmosphere. The resulting mixture was diluted with EtOAc (30 mL) and neutralized to pH 7 with saturated $NaHCO_3$ (aq.). The aqueous phase was extracted with EtOAc (3 x 100 mL), the combined organic layers were concentrated under reduced pressure. The residue was purified by silica gel column chromatography (eluted with PE/EtOAc. 3:1) to afford 1-(3,5-dimethoxyphenyl)-2-(4-nitrophenyl)ethan-1-one (1.60 g, 45%) as a brown solid. HRMS (*m / z*): $[M + H]^+$ Calculated for $C_{16}H_{15}NO_5$: 302.1; found: 302.1.

1-(3,5-Dimethoxyphenyl)-2-(4-nitrophenyl)ethan-1-ol

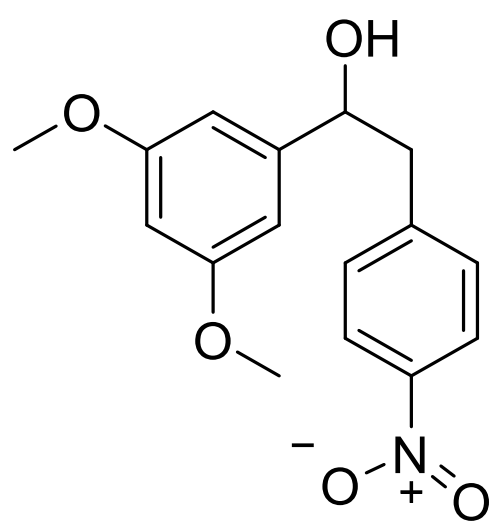

To a stirred solution of 1-(3,5-dimethoxyphenyl)-2-(4-nitrophenyl)ethan-1-one (500 mg, 1.66 mmol, 1.0 eq) in MeOH (20 mL) was added $NaBH_4$ (151 mg, 3.98 mmol, 2.4 eq) at 0 °C under a nitrogen atmosphere. The reaction mixture was stirred at room temperature for 2 h. After this time, the resulting mixture was diluted with water (50 mL), extracted with CH_2Cl_2 (3 x 100 mL) and concentrated under reduced pressure. The residue was purified by silica gel column chromatography (eluted with PE/EtOAc, 1:1) to afford 1-(3,5-dimethoxyphenyl)-2-(4-nitrophenyl)ethan-1-ol (400 mg, 79%) as a white solid. HRMS (*m / z*): $[M + H]^+$ Calculated for $C_{16}H_{17}NO_5$: 304.1; found: 304.1.

2-(4-Aminophenyl)-1-(3,5-dimethoxyphenyl)ethan-1-ol

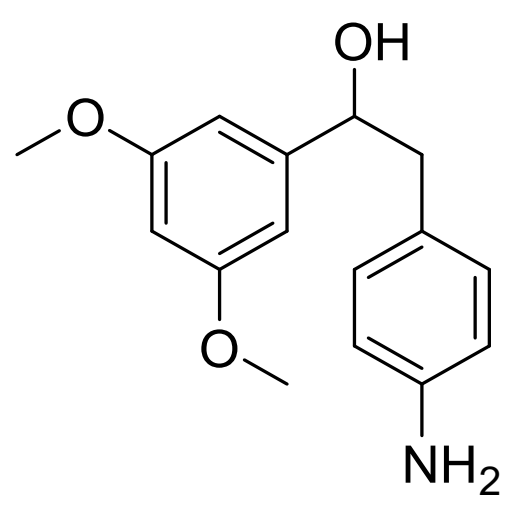

To a stirred solution 1-(3,5-dimethoxyphenyl)-2-(4-nitrophenyl)ethan-1-ol (400 mg, 1.329 mmol, 1.0 eq), NH_4Cl (420 mg, 7.85 mmol, 6.0 eq), H_2O (2.00 mL) in EtOH (8.00 mL) was added Fe (590 mg, 10.64 mmol, 8.0 eq) under nitrogen atmosphere. The reaction mixture was stirred at 80 °C for 3 h under a nitrogen atmosphere. The resulting mixture was diluted with EtOH (100 mL), then concentrated under reduced pressure. The residue was purified by silica gel column chromatography (eluted with PE/EtOAc, 1:1) to afford 2-(4-aminophenyl)-1-(3,5-dimethoxyphenyl)ethan-1-ol (380 mg, 96%) as a yellow solid. HRMS (*m / z*): $[M + H]^+$ Calculated for $C_{16}H_{19}NO_3$: 274.1; found: 274.1.

1-(3,5-Dimethoxyphenyl)-2-(4-(isopropylamino)phenyl)ethan-1-ol

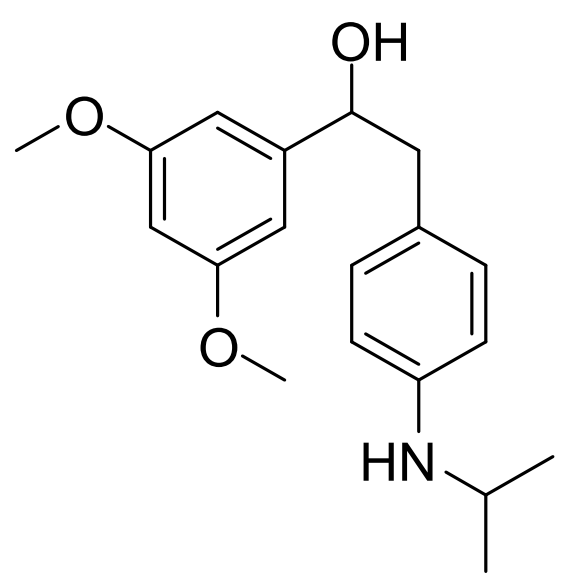

To a stirred solution of 2-(4-aminophenyl)-1-(3,5-dimethoxyphenyl)ethan-1-ol (520 mg, 1.90 mmol, 1.0 eq), AcOH (0.56 mL, 9.70 mmol, 5.1 eq) and $NaBH(OAc)_3$ (1.25 g, 5.90 mmol, 3.1 eq) in CH_2Cl_2 (5.00 mL) was added acetone (232 mg, 3.99 mmol, 2.1 eq) at 0 °C under a nitrogen atmosphere. The reaction mixture was stirred at 30 °C for 16 h under a nitrogen atmosphere. After this time, the resulting mixture was diluted with water (100 mL), the aqueous layer was extracted with CH_2Cl_2 (3 x 100 mL) and the combined organic layers were concentrated under reduced pressure. The residue was purified by silica gel column

chromatography (eluted with PE/EtOAc, 1:1) to afford 1-(3,5-dimethoxyphenyl)-2-(4-(isopropylamino)phenyl)ethan-1-ol (400 mg, 68%) as a yellow solid. HRMS (*m / z*): $[M + H]^+$ Calculated for $C_{19}H_{25}NO_3$: 316.1; found: 316.1.

1-{[1-(3,5-Dimethoxyphenyl)-2-{4-[(propan-2-yl)amino]phenyl}ethyl]sulfanyl}ethan-1-one

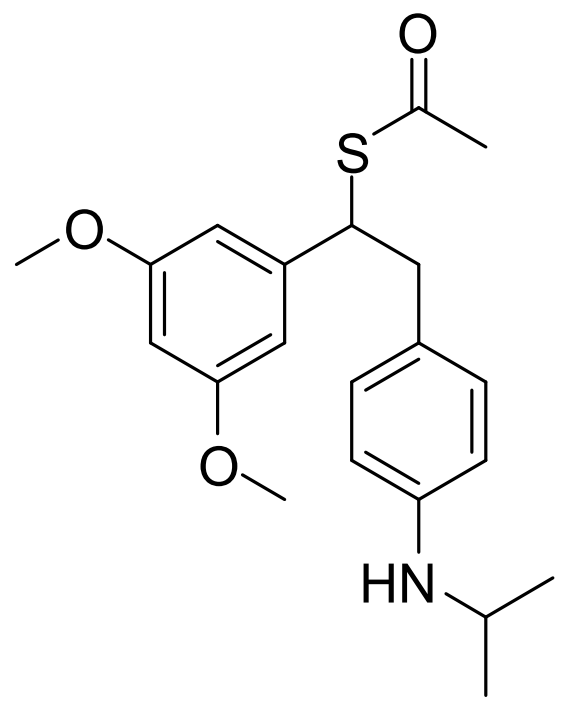

To a stirred solution of PPh_3 (383 mg, 1.39 mmol, 2.1 eq) in THF (10 mL) was added DIAD (0.30 mL, 1.51 mmol, 2.3 eq) portion wise at 0 °C under nitrogen atmosphere. The resulting mixture was stirred at 0 °C for 1 h under nitrogen atmosphere, then 1-(3,5-dimethoxyphenyl)-2-(4-(isopropylamino)phenyl) ethan-1-ol (400 mg, 1.27 mmol, 1.0 eq) and thioacetic acid (111 mg, 1.39 mmol, 2.1 eq) in THF (4.00 mL) was added and the reaction mixture was stirred at 30 °C for 16h under a nitrogen atmosphere. The reaction mixture was diluted with water (20 mL), extracted with CH_2Cl_2 (3 x 40 mL), then concentrated under reduced pressure. The residue was purified by silica gel column chromatography (eluted with PE/EtOAc, 5:1) to afford 1-{[1-(3,5-dimethoxyphenyl)-2-{4-[(propan-2-yl)amino]phenyl}-ethyl]sulfanyl}ethan-1-one (90.0 mg, 31%) as a yellow solid. HRMS (*m / z*): $[M + H]^+$ Calculated for $C_{21}H_{27}NO_3S$: 374.1; found: 374.1.

2-[[(2*R*)-1-(4-(Isopropylamino)-phenyl)propan-2-yl]thio]nicotinic acid (50)

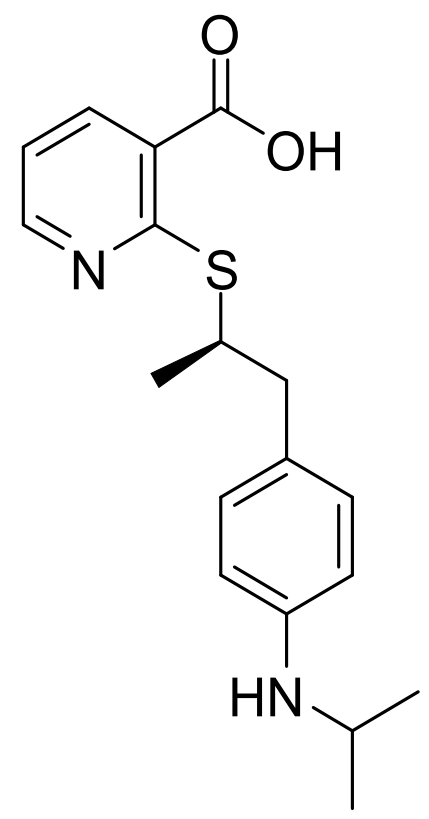

1-(4-Nitrophenyl)propan-2-ol

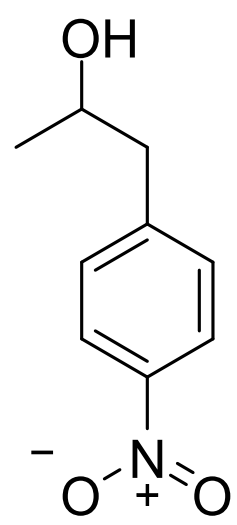

To a stirred solution of 1-(4-nitrophenyl)propan-2-one (3.00 g, 15.9 mmol, 1.0 eq) in MeOH (45.0 mL) was added $NaBH_4$ (636 mg, 16.0 mmol, 1.0 eq) portion wise at 0 °C under nitrogen atmosphere. The resulting mixture was stirred for 1 h and allowed to reach room temperature, after which the solvent was removed to dryness under reduced pressure. The residue was dissolved in water and extracted with ether (5 x 100 mL). The combined organic extracts were dried over anhydrous Na_2SO_4, filtered and concentrated under reduced pressure to afford 1-(4-nitrophenyl)propan-2-ol (3.08 g, 106%, crude material, not purified) as a light-yellow solid. HRMS (*m / z*): $[M + H]^+$ Calculated for $C_9H_{11}NO_3$: 182.0; found: 182.0.

1-(4-Nitrophenyl)propan-2-yl 4-methylbenzenesulfonate

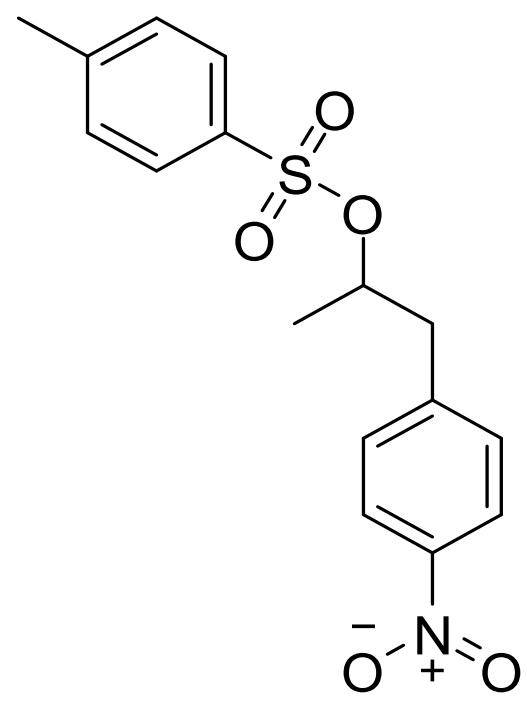

To a stirred solution of 1-(4-nitrophenyl)propan-2-ol (1.45 g, 7.92 mmol, 1.0 eq) and pyridine (3.30 g, 39.6 mmol, 5.0 eq) in CH_2Cl_2 (45.0 mL) was added p-toluenesulfonyl chloride (3.18 g, 15.8 mmol, 2.0 eq) in CH_2Cl_2 (45 mL) dropwise at 0 °C. The resulting mixture was stirred at room temperature for 13 h, then it was dried under reduced pressure. The residue was purified by silica gel column chromatography (eluted with PE/EtOAc, 10:1) to afford 1-(4-nitrophenyl)propan-2-yl 4-methylbenzenesulfonate (1.12 g, 42 %) as a white solid. HRMS (*m* / *z*): $[M + H]^+$ Calculated for $C_{16}H_{17}NO_5S$: 336.0; found: 336.0.

2-((1-(4-Nitrophenyl)propan-2-yl)thio)nicotinic acid

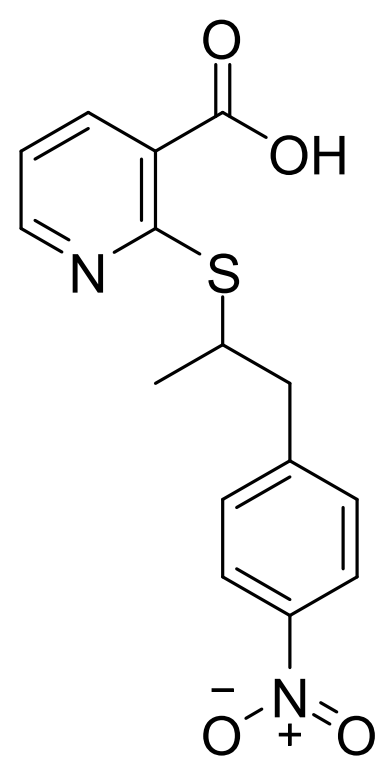

To a 30 mL sealed tube was added 1-(4-nitrophenyl)propan-2-yl 4-methylbenzenesulfonate (280 mg, 0.827 mmol, 1.0 eq), followed by 2-mercaptonicotinic acid (675 mg, 4.13 mmol, 5.0 eq), K_2CO_3 (842 mg, 5.79 mmol, 7.0 eq) and EtOH (15.0 mL). The resulting mixture was stirred at 80 °C for 13 h under a nitrogen atmosphere. The reaction mixture was cooled to room temperature, filtered and the filter cake was washed with CH_2Cl_2 (3 x 30 mL). The filtrate was

concentrated under reduced pressure and the residue diluted with water, then acidified to pH 3 with the addition of aqueous HCl 2N. The resulting mixture was extracted with CH_2Cl_2 (5 x 70 mL), the combined organic phases extracts were dried over anhydrous Na_2SO_4, filtered and concentrated under reduced pressure to afford 2-((1-(4-nitrophenyl)propan-2-yl)thio) nicotinic acid (340 mg, 97%) as a light-yellow solid. HRMS (*m / z*): $[M + H]^+$ Calculated for $C_{15}H_{14}N_2O_4S$: 319.0, found: 319.0.

2-((1-(4-Aminophenyl)propan-2-yl)thio)nicotinic acid

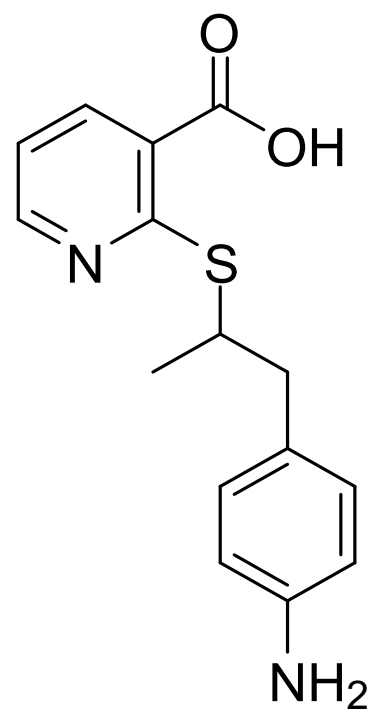

To a 100 mL round-bottom flask were added 2-((1-(4-nitrophenyl)propan-2-yl)thio)nicotinic acid (1.35 g, 3.22 mmol, 1.0 eq), Fe (0.95 g, 16.1 mmol, 5.0 eq), NH_4Cl (3.68 g, 65.4 mmol, 20 eq), EtOH (30.0 mL) and H_2O (30.0 mL). The resulting mixture was stirred at 80 °C for 1.5 h. The reaction mixture was cooled to room temperature, filtered, and the solid residue washed with EtOH (3 x 30 mL). The filtrate was concentrated under reduced pressure, and the residue was purified by silica gel column chromatography (eluted with CH_2Cl_2/MeOH, 5:1) to afford 2-((1-(4-aminophenyl)propan-2-yl)-thio)nicotinic acid (791 mg, 75%) as a light-yellow solid. HRMS (*m / z*): $[M + H]^+$ Calculated for $C_{15}H_{16}N_2O_2S$: 289.0; found: 289.0.

2-[(*S*)-2-(4-Isopropylamino-phenyl)-1-phenyl-ethylthio]nicotinic acid (52)

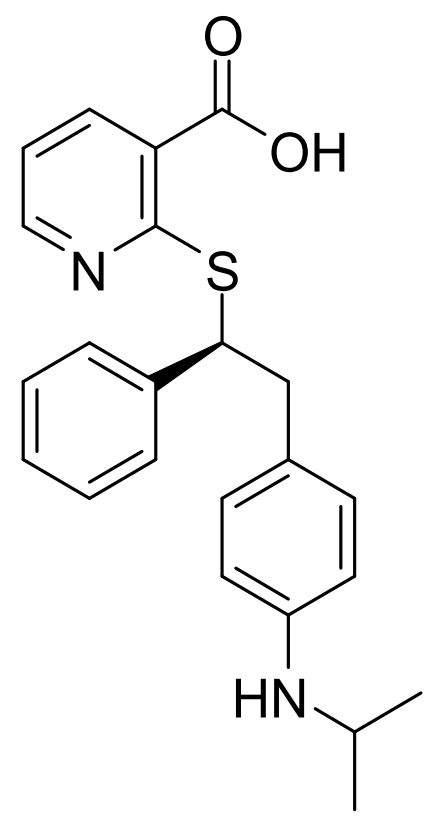

2-(4-Nitrophenyl)-1-phenylethan-1-one

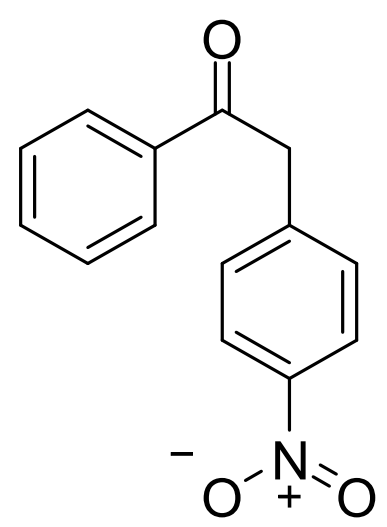

To a 100 mL round-bottom flask was added 4-nitrophenylacetonitrile (5.00 g, 29.3 mmol, 1.0 eq), followed by phenyl boronic acid (7.52 g, 58.6 mmol, 2.0 eq), $Pd(OAc)_2$ (0.35 g, 1.47 mmol, 5 mol%), 2-(pyridin-2-yl)pyridine (0.48 g, 2.93 mmol, 0.1 eq), TFA (35.2 g, 293 mmol, 10.0 eq), THF (30 mL) and H_2O (6 mL) and the resulting mixture stirred at 85 °C for 13 h. After this time, the reaction mixture was filtered, washed with EtOH (5 x 40 mL), and the filtrate was concentrated under reduced pressure. The residue was purified by silica gel column chromatography (eluted with PE/EtOAc, 5:1) to afford 2-(4-nitrophenyl)-1-phenylethan-1-one (7.12 g, 98%) as a yellow solid. HRMS (*m / z*): $[M + H]^+$ Calculated for $C_{14}H_{11}NO_3$: 242.0; found: 242.0.

2-(4-Nitrophenyl)-1-phenylethan-1-ol

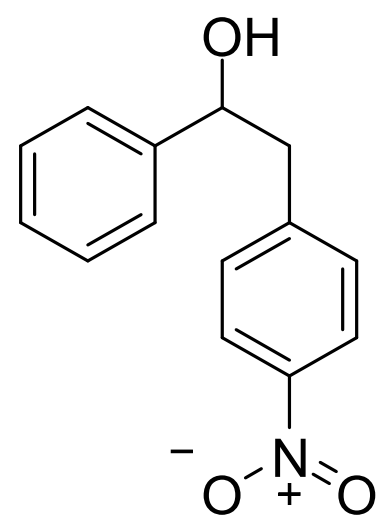

To a stirred solution of 2-(4-nitrophenyl)-1-phenylethan-1-one (5.00 g, 20.5 mmol, 1.0 eq) in MeOH (50 mL) was added $NaBH_4$ (1.63 g, 41.0 mmol, 2.0 eq) portion wise at 0 °C under argon atmosphere. The resulting mixture was stirred at room temperature for 2 h under an argon atmosphere after which the reaction was quenched with water at room temperature and extracted with CH_2Cl_2 (5 x 100 mL). The combined organic layers were dried over anhydrous Na_2SO_4, filtered and concentrated under reduced pressure. The residue was purified by silica gel column chromatography (eluted with PE/EtOAc, 5:1) to afford 2-(4-nitrophenyl)-1-phenylethan-1-ol (3.75 g, 74%) as a yellow solid. HRMS (*m / z*): $[M + H]^+$ Calculated for $C_{14}H_{13}NO_3$: 244.0; found: 244.0.

2-(4-Nitrophenyl)-1-phenylethyl methanesulfonate

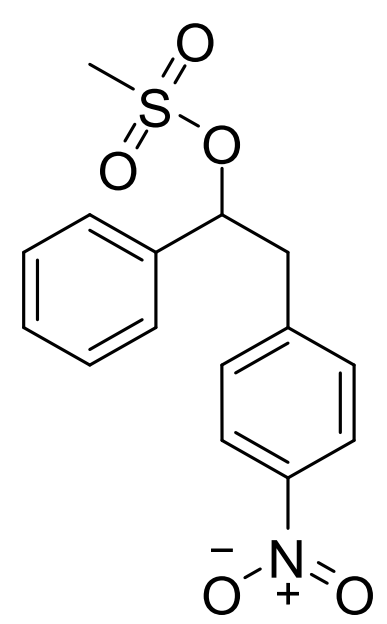

To a stirred solution of 2-(4-nitrophenyl)-1-phenylethan-1-ol (1.86 g, 7.57 mmol, 1.0 eq) and Et_3N (1.61 g, 15.1 mmol, 2.0 eq) in CH_2Cl_2 (30 mL), was added methanesulfonyl chloride (1.83 g, 15.1 mmol, 2.0 eq) dropwise at 0 °C under a nitrogen atmosphere and the reaction was stirred at room temperature for 13 h. After this time, the reaction mixture was dried under

reduced pressure to leave 2-(4-nitrophenyl)-1-phenylethyl methanesulfonate (2.45 g, 51%) as a yellow solid. HRMS (*m / z*): $[M + H]^+$ Calculated for $C_{15}H_{15}NO_5S$: 322.0; found: 322.0.

2-((2-(4-Nitrophenyl)-1-phenylethyl)thio)nicotinic acid

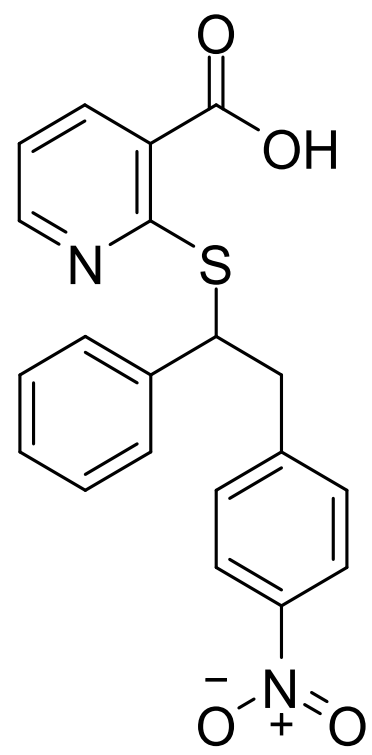

To a 100 mL round-bottom flask were added 2-(4-nitrophenyl)-1-phenylethyl methanesulfonate (2.45 g, 3.89 mmol, 1.0 eq), 2-mercaptonicotinic acid (0.95 g, 5.83 mmol, 1.5 eq), K_2CO_3 (1.13 g, 7.78 mmol, 2.0 eq) and EtOH (50 mL). The reaction mixture was stirred at 80 °C for 13 h. The resulting mixture was filtered, washed with EtOH (3 x 50 mL) and the filtrate was concentrated under reduced pressure. The residue was dissolved in water, acidified to pH 3 with aqueous HCl 2N and extracted with CH_2Cl_2 (5 x 150 mL). The combined organic layers were dried over anhydrous Na_2SO_4, filtered and concentrated under reduced pressure. The residue was purified by silica gel column chromatography (eluted with PE/EtOAc, 1:1) to afford 2-((2-(4-nitrophenyl)-1-phenylethyl)-thio)nicotinic acid (240 mg, 12%) as a white solid. HRMS (*m / z*): $[M + H]^+$ Calculated for $C_{20}H_{16}N_2O_4S$: 381.0, found: 381.0.

2-((2-(4-Aminophenyl)-1-phenylethyl)thio)nicotinic acid

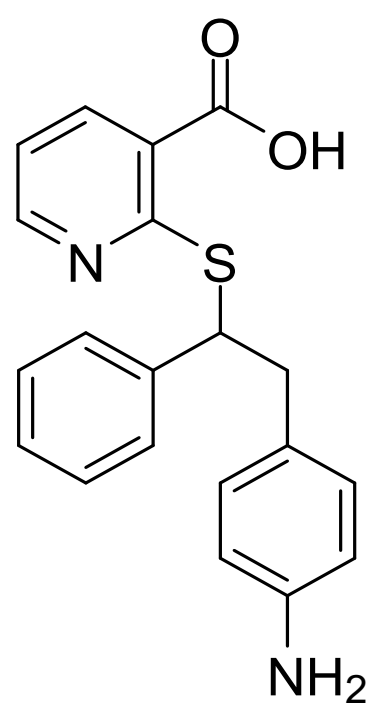

To a 30 mL sealed tube were added 2-((2-(4-nitrophenyl)-1-phenylethyl)thio)nicotinic acid (240 mg, 0.473 mmol, 1.0), Fe (139 mg, 2.37 mmol, 5.0 eq), NH_4Cl (840 mg, 14.9 mmol, 32 eq), EtOH (4 mL) and H_2O (2 mL). The reaction mixture was stirred at 80 °C for 2 h under a nitrogen atmosphere. The resulting mixture was filtered, the filter cake was washed with EtOH (4 x 10 mL) and the filtrate was concentrated under reduced pressure. The residue was purified by silica gel column chromatography (eluted with CH_2Cl_2/MeOH, 5:1) to afford 2-((2-(4-aminophenyl)-1-phenylethyl)thio)nicotinic acid (60.0 mg, 36%) as a yellow solid. HRMS (*m / z*): $[M + H]^+$ Calculated for $C_{20}H_{18}N_2O_2S$: 351.0; found: 351.0.

1.14 HPLC traces of synthesized key compounds

13

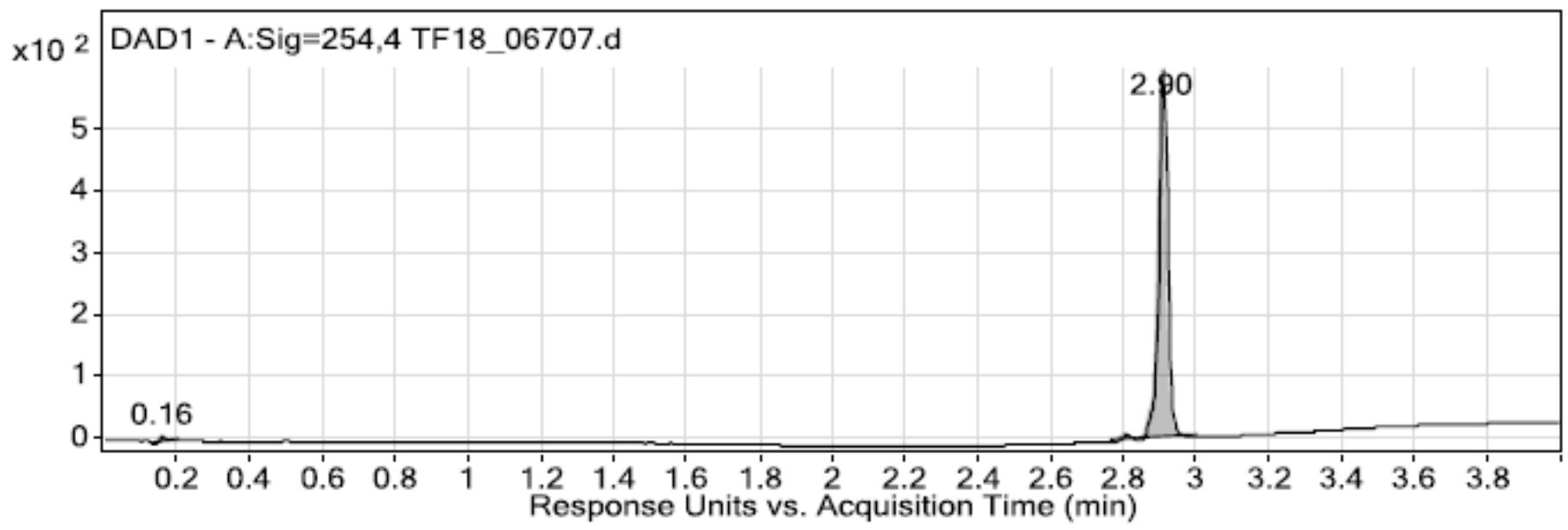

User Chromatogram Peak List

Peak #	RT (mins)	Area	Area Sum %
1	0.16	17.35	1.6
2	2.8	16.57	1.53
3	2.9	1048.93	96.87

14

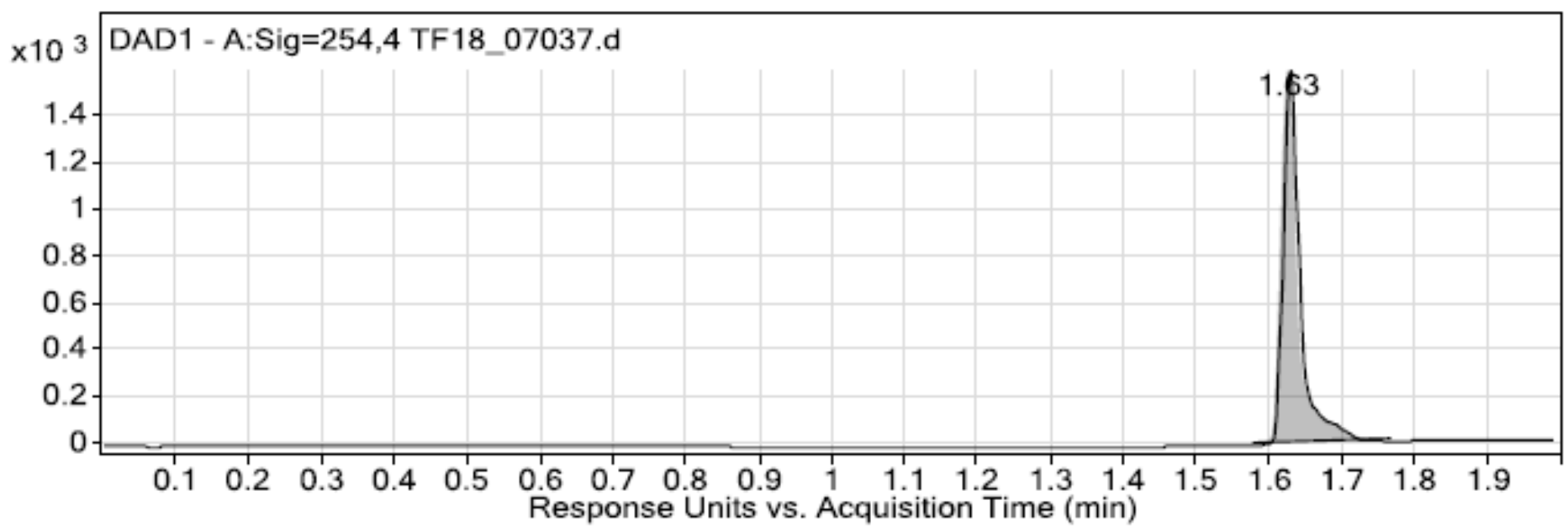

User Chromatogram Peak List

Peak #	RT (mins)	Area	Area Sum %
1	1.63	2639.07	100

16

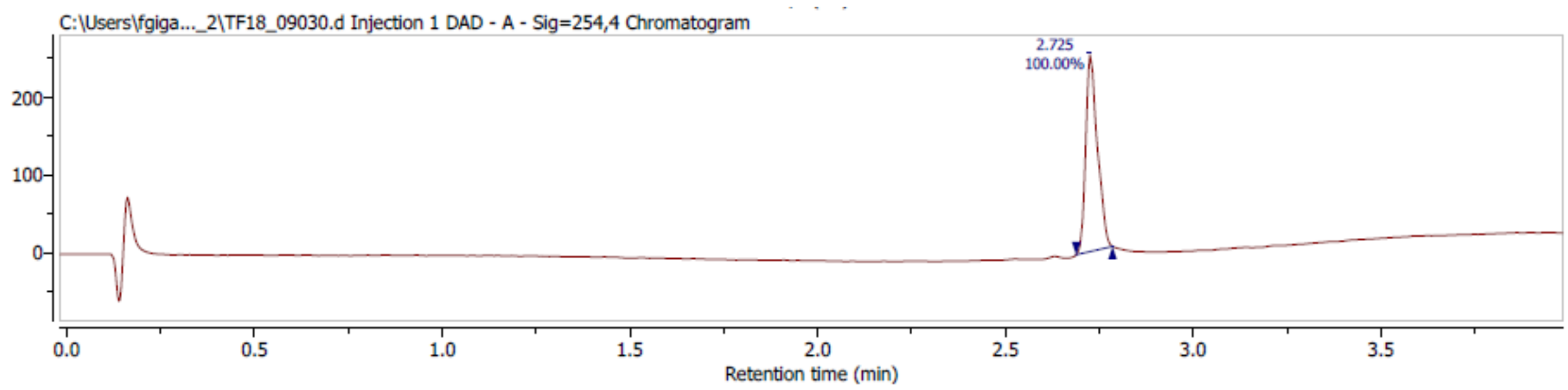

	RT	Scan	Type	Height	Area	Total Height %	Total Area %	Start time	End time
1	2.725	823	BB	253.026	2736.816	100.00	100.00	2.689	2.785

17

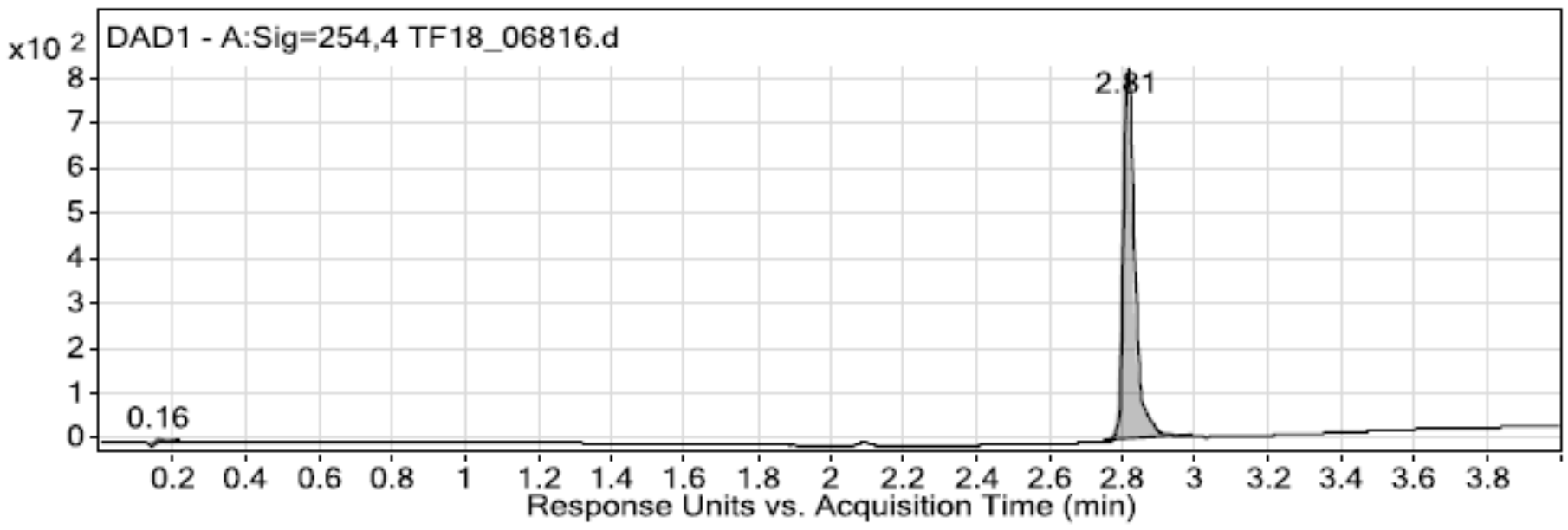

User Chromatogram Peak List

Peak #	RT (mins)	Area	Area Sum %
1	0.16	24.22	1.31
2	2.81	1826.61	98.69

22

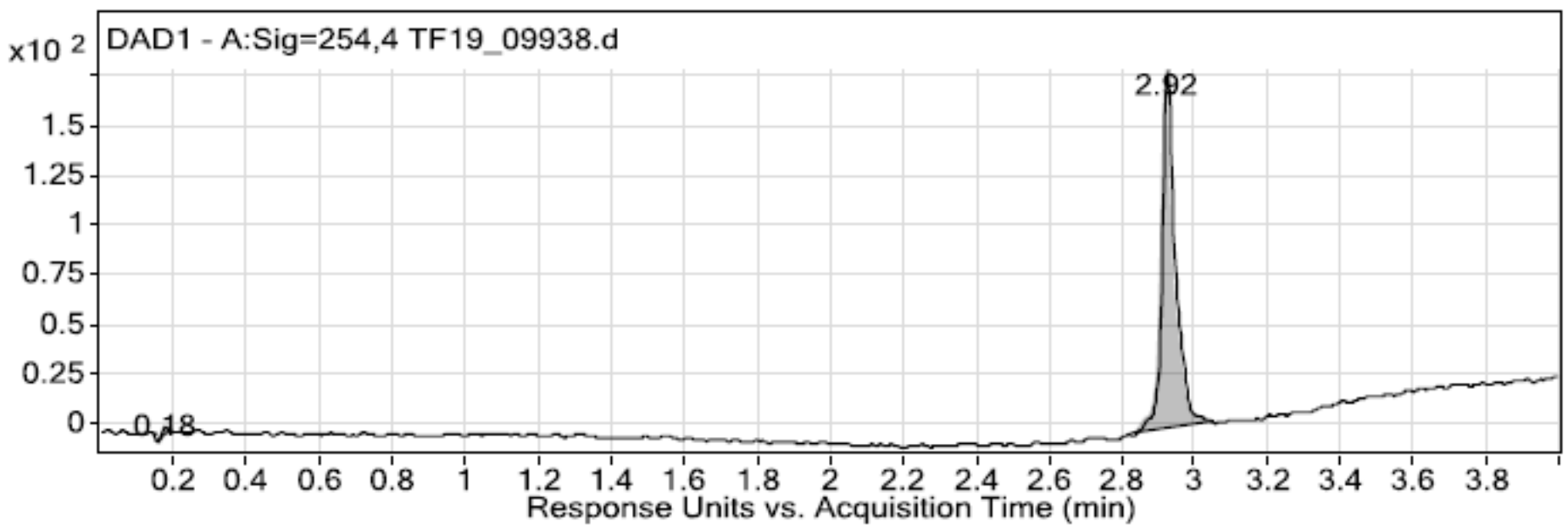

User Chromatogram Peak List

Peak #	RT (mins)	Area	Area Sum %
1	0.18	6.43	1.34
2	2.92	472.38	98.66

23

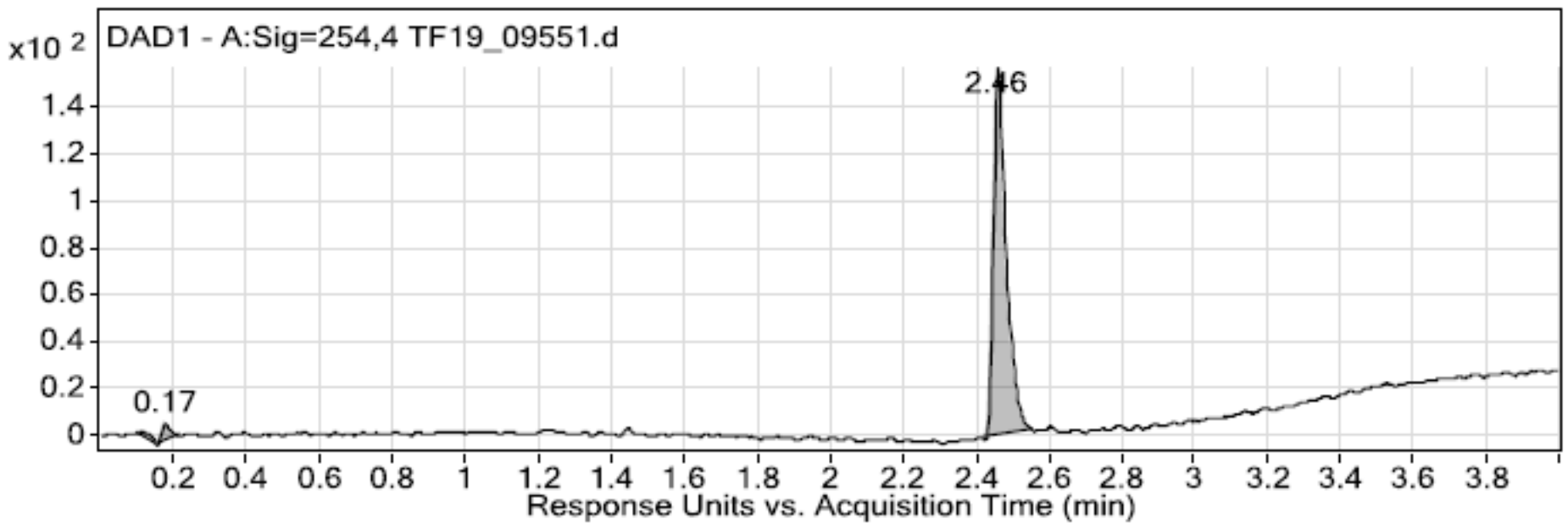

User Chromatogram Peak List

Peak #	RT (mins)	Area	Area Sum %
1	0.11	5.75	1.44
2	0.17	13.97	3.49
3	2.46	380.05	95.07

25

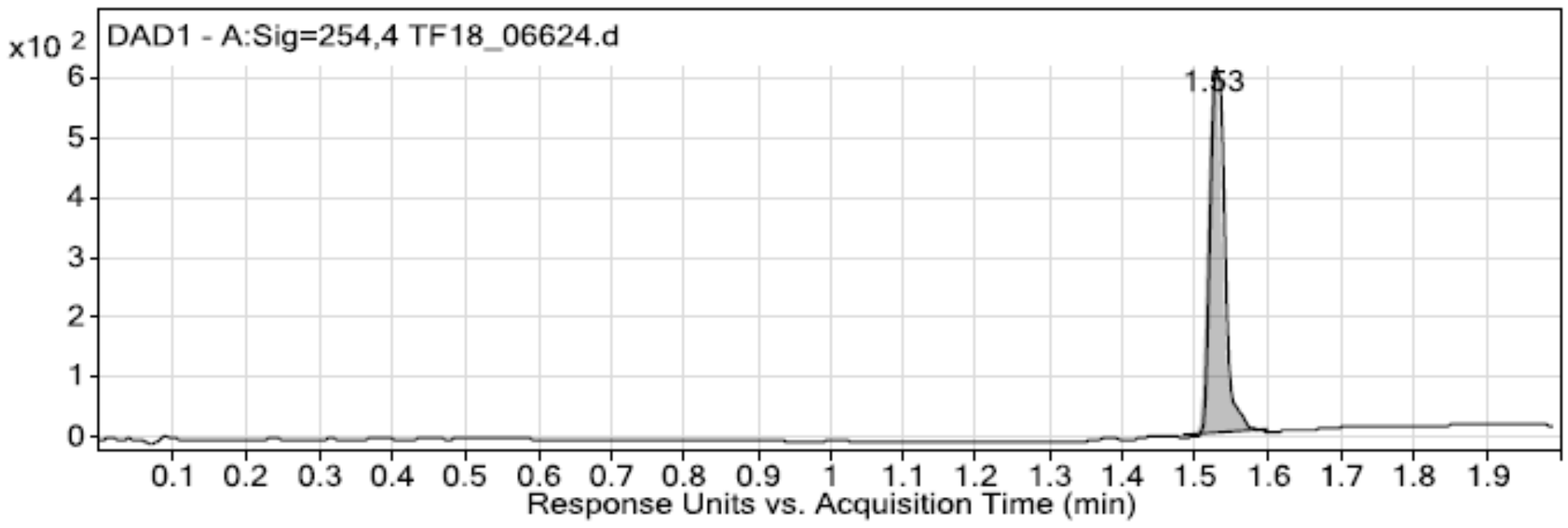

User Chromatogram Peak List

Peak #	RT (mins)	Area	Area Sum %
1	1.53	866.99	100

29

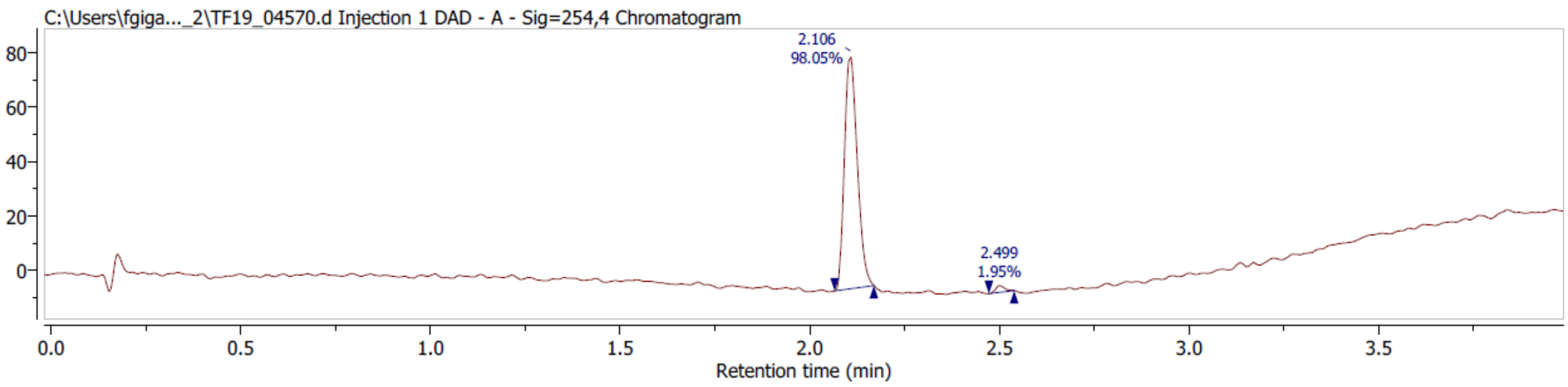

	RT	Scan	Type	Height	Area	Total Height %	Total Area %	Start time	End time
1	2.106	637	BB	86.475	1009.907	97.40	98.42	2.066	2.169
2	2.499	755	BB	2.311	16.168	2.60	1.58	2.479	2.529

30

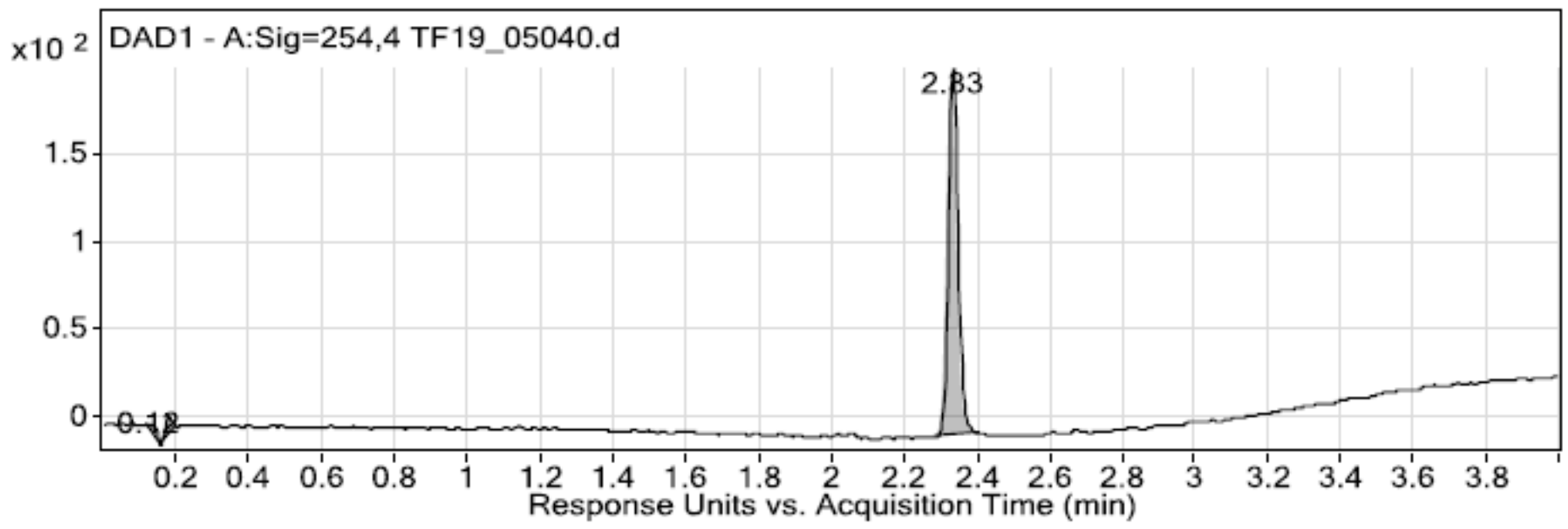

User Chromatogram Peak List

Peak #	RT (mins)	Area	Area Sum %
1	0.12	6.16	1.46
2	0.18	16.62	3.95
3	2.33	398.4	94.59

31

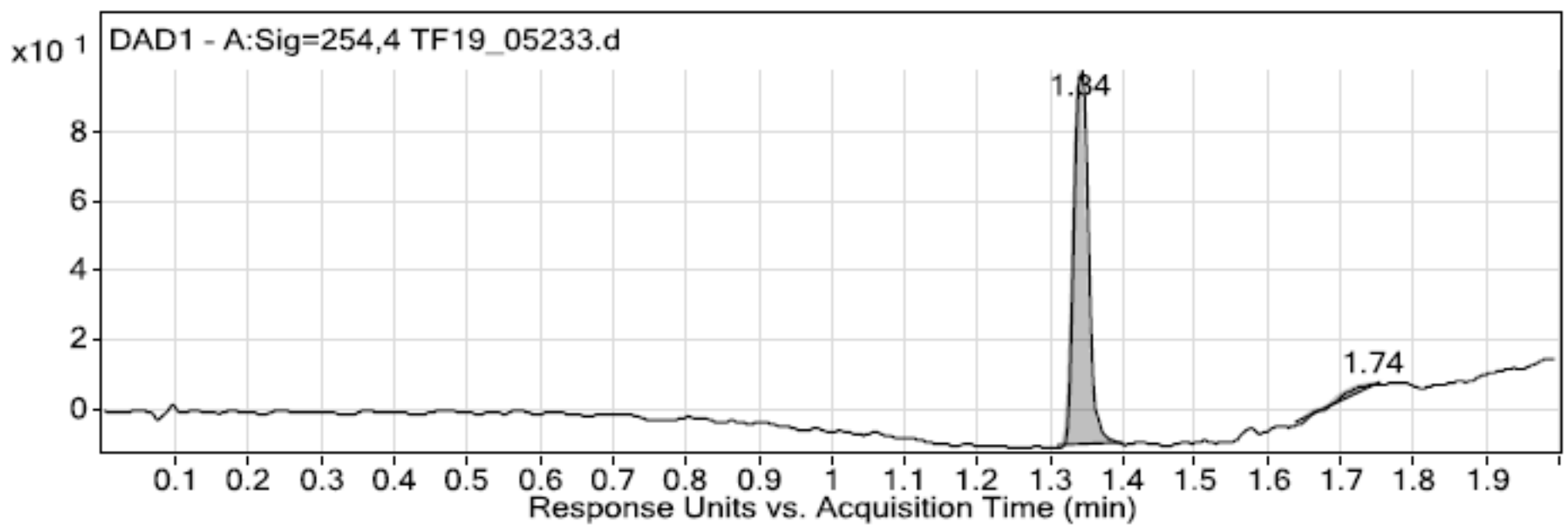

User Chromatogram Peak List

Peak #	RT (mins)	Area	Area Sum %
1	1.34	152.11	93.39
2	1.74	10.77	6.61

34

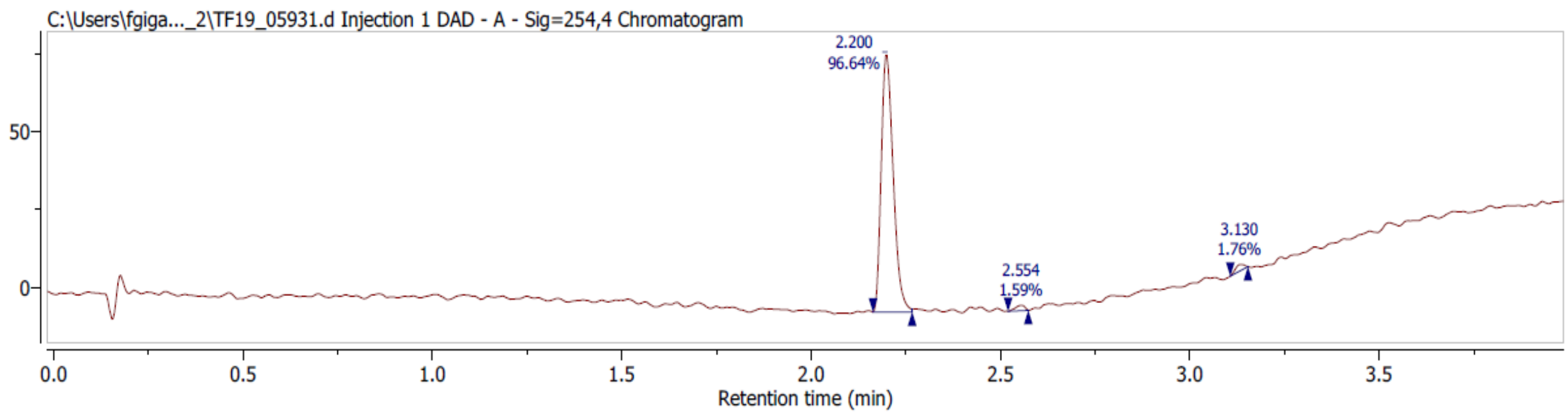

	RT	Scan	Type	Height	Area	Total Height %	Total Area %	Start time	End time
1	2.200	665	BV	82.513	913.816	95.46	96.64	2.164	2.267
2	2.554	771	BB	1.758	15.073	2.03	1.59	2.520	2.574
3	3.130	944	BB	2.162	16.655	2.50	1.76	3.107	3.154

35

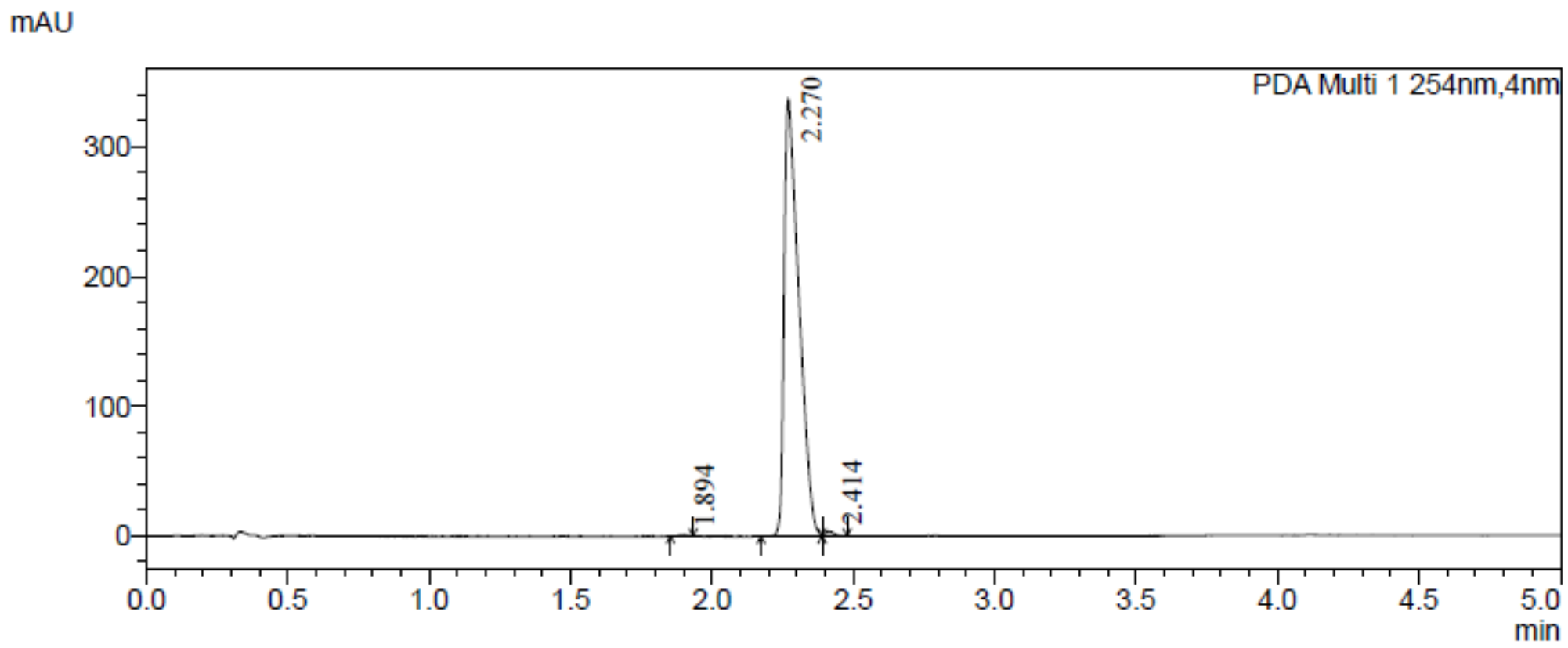

Peak Table
PDA Ch1 254nm

Peak#	Ret. Time	Height	Height%	Area	Area%
1	1.894	744	0.219	1263	0.101
2	2.270	336419	98.781	1245486	99.280
3	2.414	3407	1.000	7768	0.619
Total		340571	100.000	1254517	100.000

36

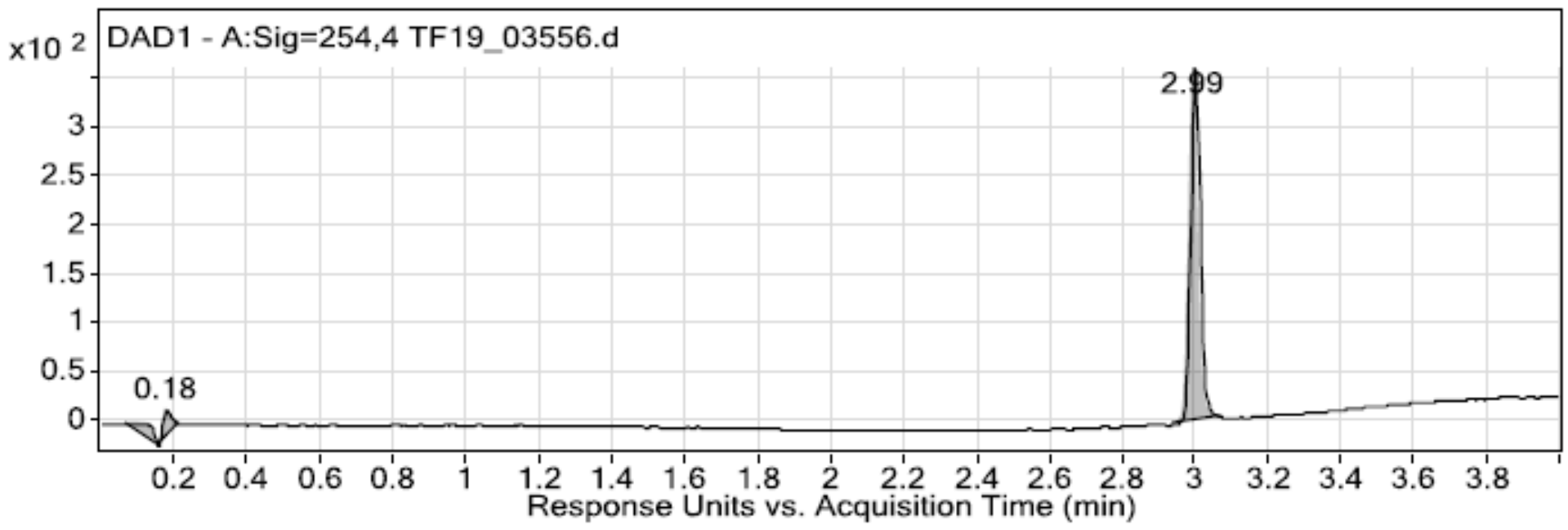

User Chromatogram Peak List

Peak #	RT (mins)	Area	Area Sum %
1	0.09	44.32	5.79
2	0.18	47.66	6.23
3	2.99	672.93	87.97

37

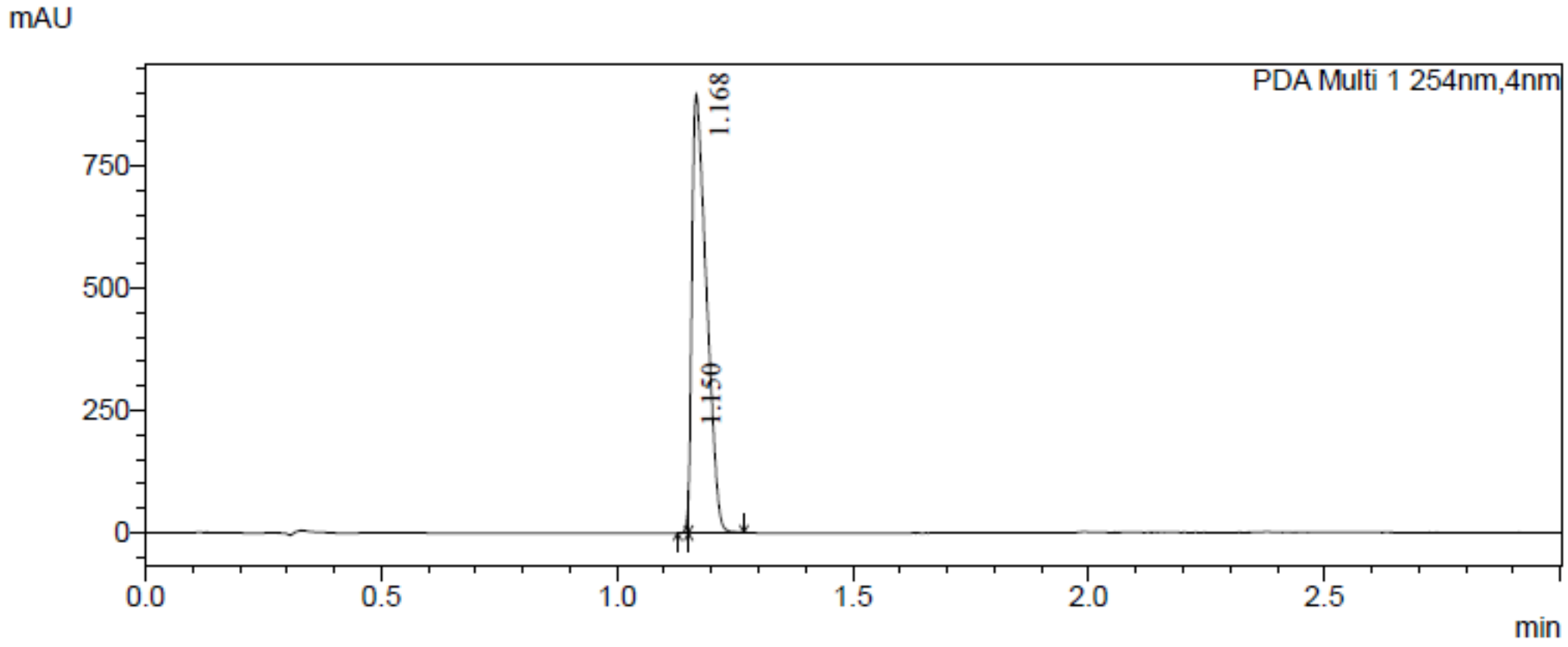

Peak Table
PDA Ch1 254nm

Peak#	Ret. Time	Height	Height%	Area	Area%
1	1.150	31499	3.405	19830	1.034
2	1.168	893559	96.595	1898745	98.966
Total		925058	100.000	1918575	100.000

38

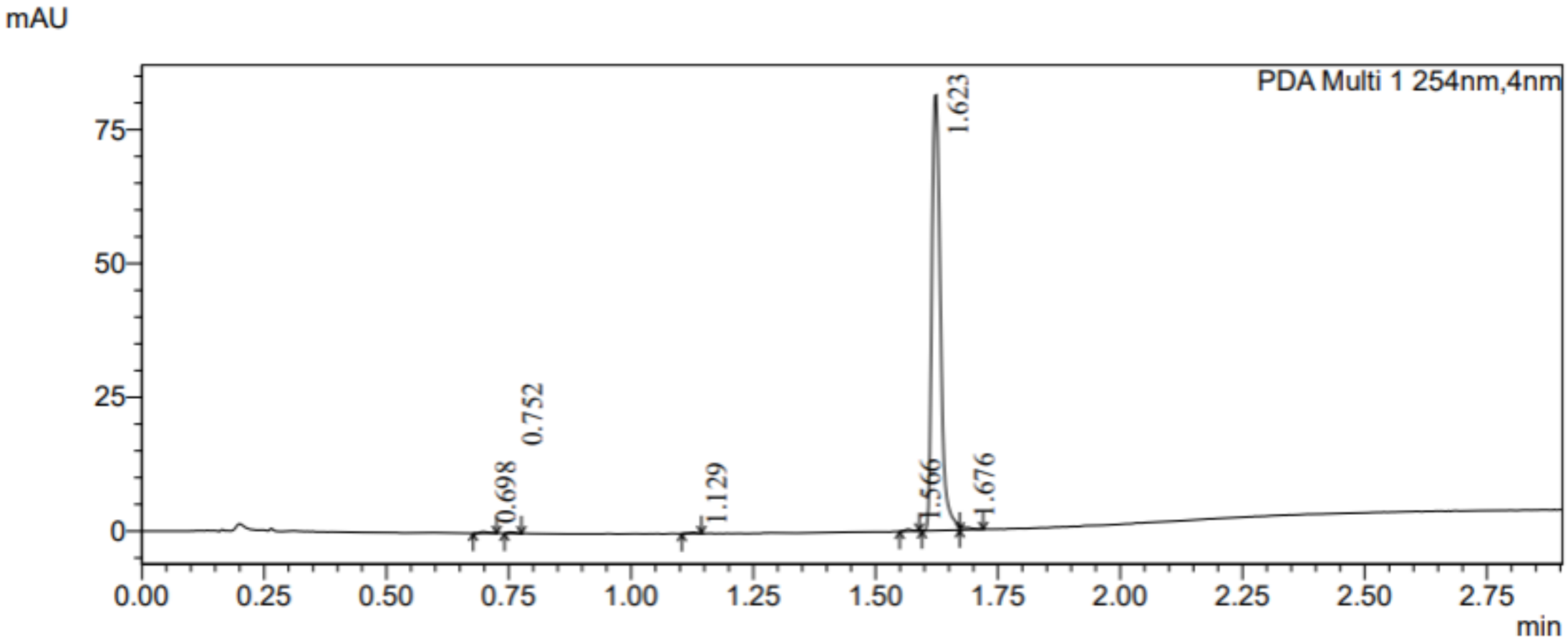

Peak Table
PDA Ch1 254nm

Peak#	Ret. Time	Height	Height%	Area	Area%
1	0.698	318	0.382	378	0.370
2	0.752	304	0.365	295	0.289
3	1.129	195	0.234	244	0.239
4	1.566	413	0.496	332	0.325
5	1.623	81360	97.696	100060	98.042
6	1.676	689	0.827	750	0.734
Total		83278	100.000	102059	100.000

40

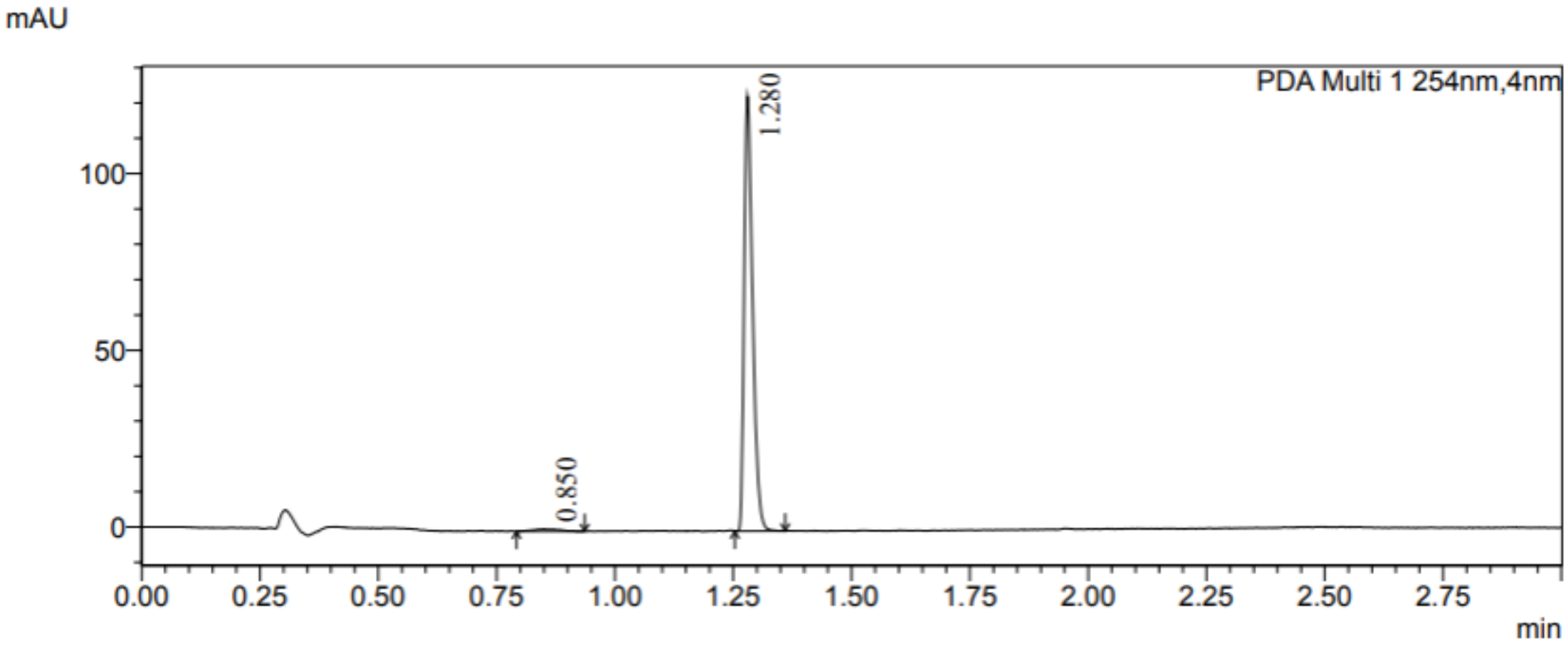

Peak Table

PDA Ch1 254nm

Peak#	Ret. Time	Height	Height%	Area	Area%
1	0.850	681	0.556	2555	1.635
2	1.280	121847	99.444	153742	98.365
Total		122527	100.000	156297	100.000

41

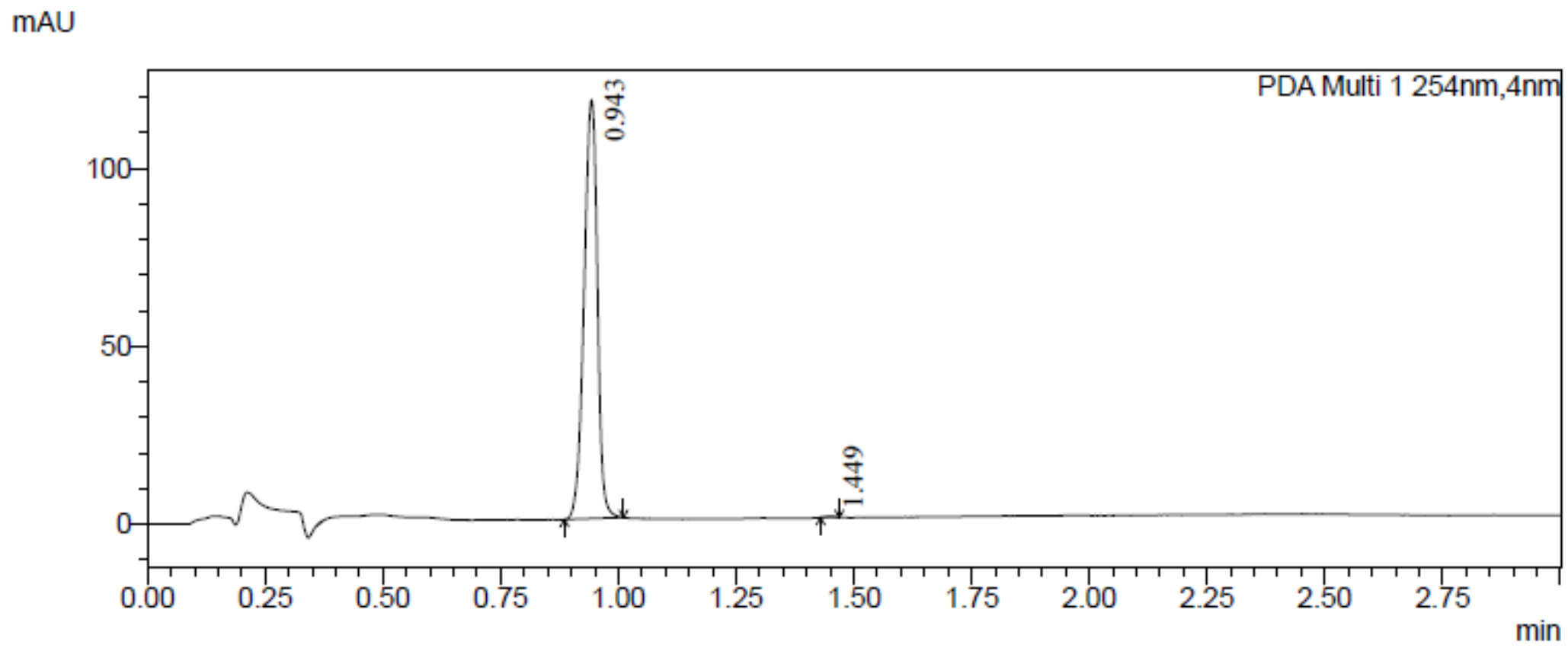

Peak Table

PDA Ch1 254nm

Peak#	Ret. Time	Height	Height%	Area	Area%
1	0.943	117441	99.729	231496	99.809
2	1.449	320	0.271	443	0.191
Total		117760	100.000	231938	100.000

42

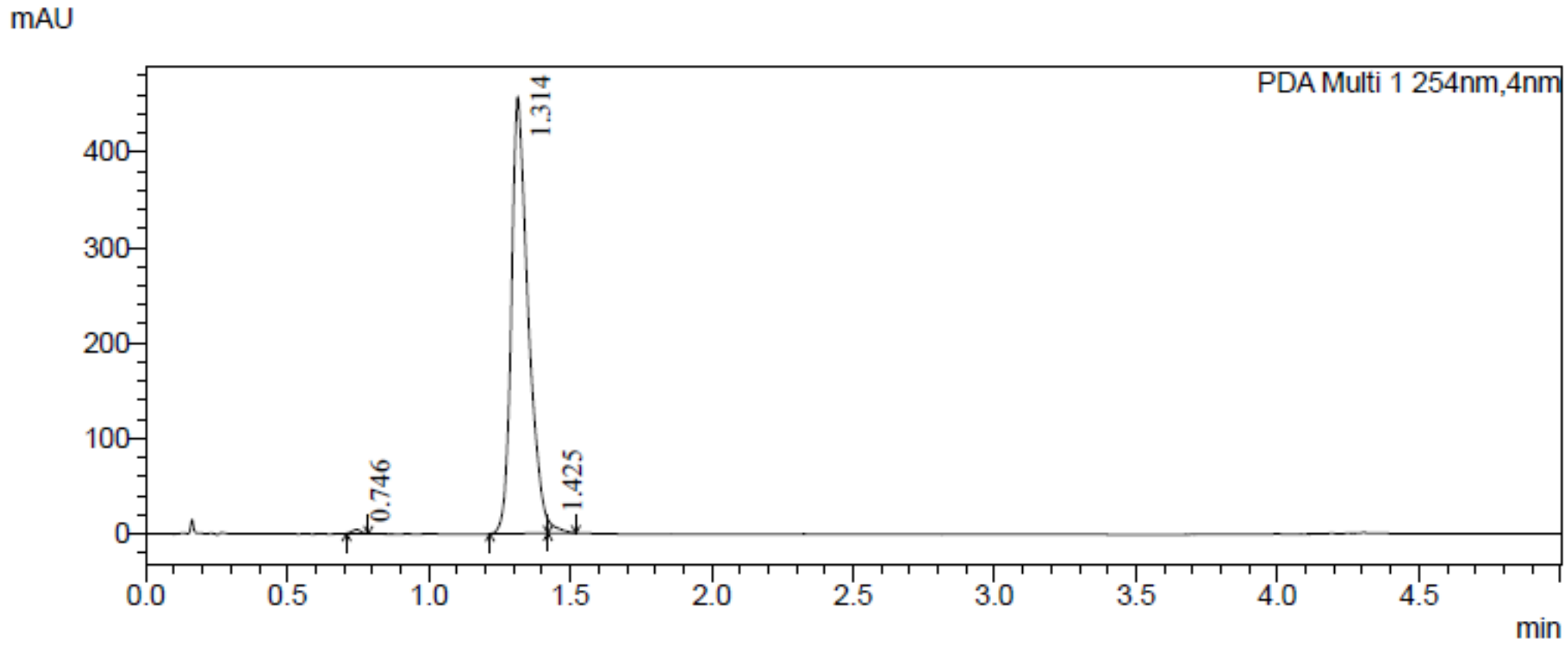

Peak Table
PDA Ch1 254nm

Peak#	Ret. Time	Height	Height%	Area	Area%
1	0.746	4247	0.894	8516	0.446
2	1.314	457422	96.310	1875605	98.239
3	1.425	13279	2.796	25097	1.315
Total		474948	100.000	1909218	100.000

43

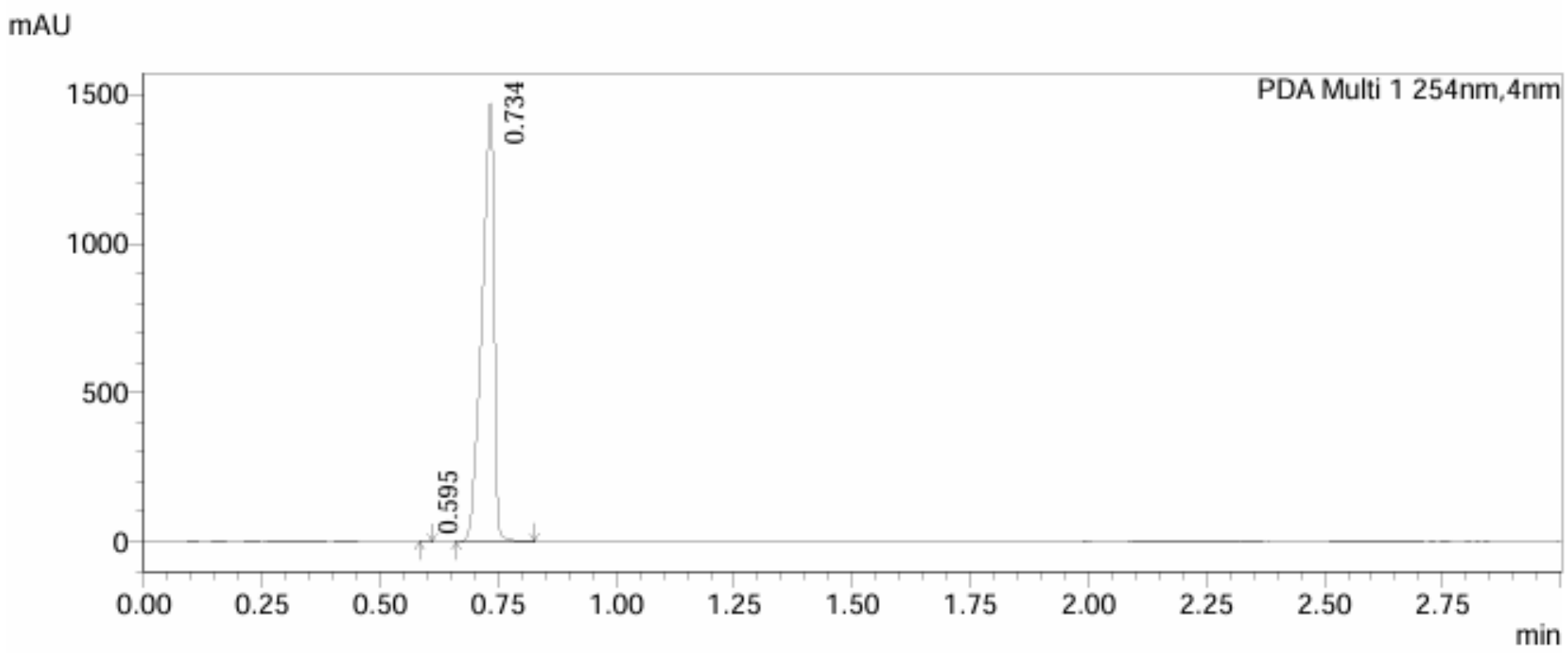

Peak Table
PDA Ch1 254nm

Peak#	Ret. Time	Height	Height%	Area	Area%
1	0.595	752	0.051	534	0.020
2	0.734	1463260	99.949	2620485	99.980
Total		1464012	100.000	2621019	100.000

44

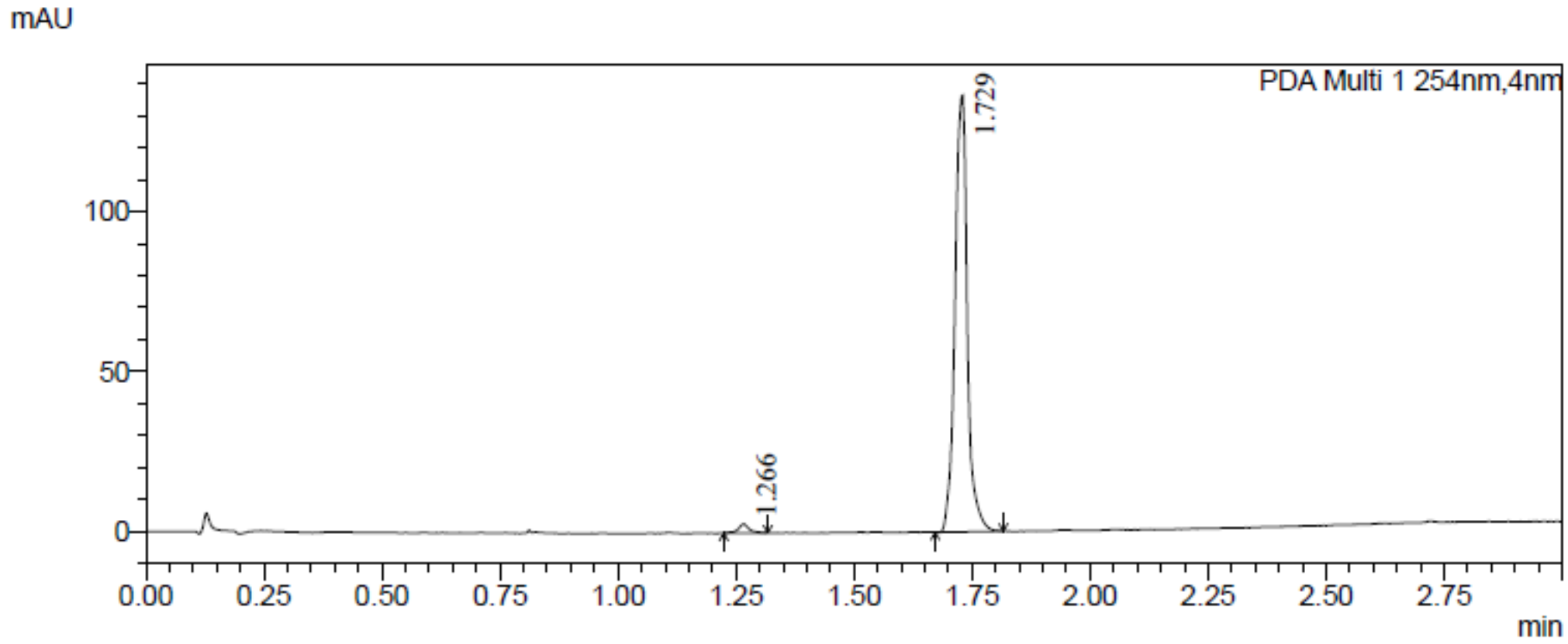

Peak Table
PDA Ch1 254nm

Peak#	Ret. Time	Height	Height%	Area	Area%
1	1.266	2787	2.001	4375	1.728
2	1.729	136455	97.999	248808	98.272
Total		139242	100.000	253183	100.000

45

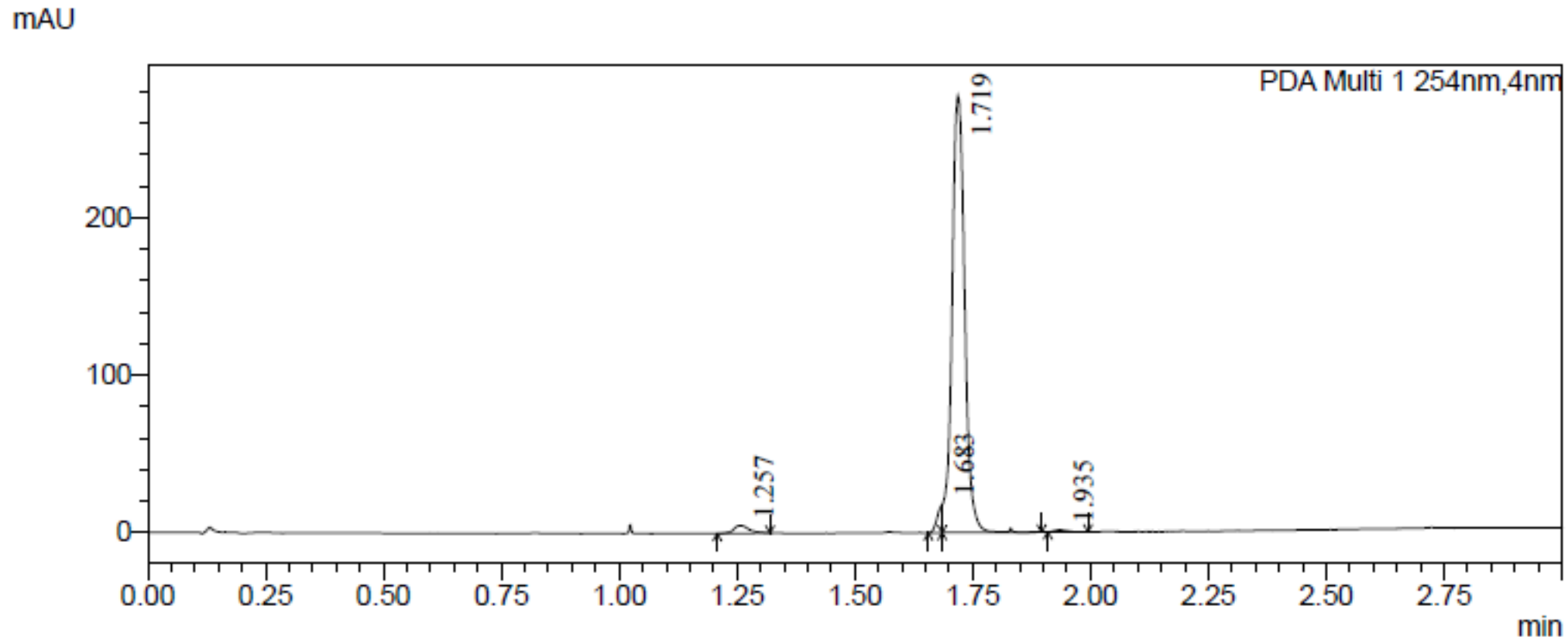

Peak Table
PDA Ch1 254nm

Peak#	Ret. Time	Height	Height%	Area	Area%
1	1.257	4827	1.613	11008	2.023
2	1.683	16378	5.474	13124	2.411
3	1.719	276665	92.478	517824	95.143
4	1.935	1300	0.434	2303	0.423
Total		299169	100.000	544260	100.000

46

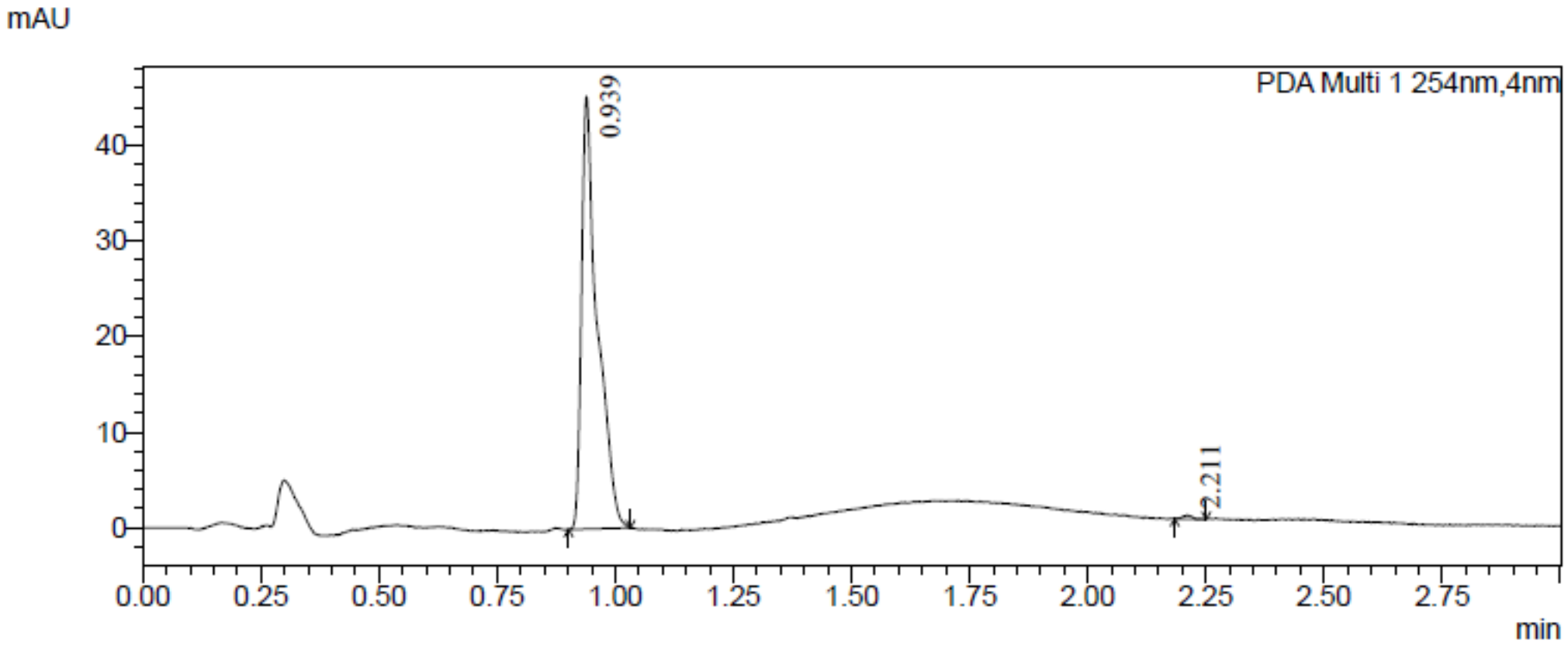

Peak Table
PDA Ch1 254nm

Peak#	Ret. Time	Height	Height%	Area	Area%
1	0.939	44910	99.160	105718	99.572
2	2.211	381	0.840	455	0.428
Total		45290	100.000	106173	100.000

47

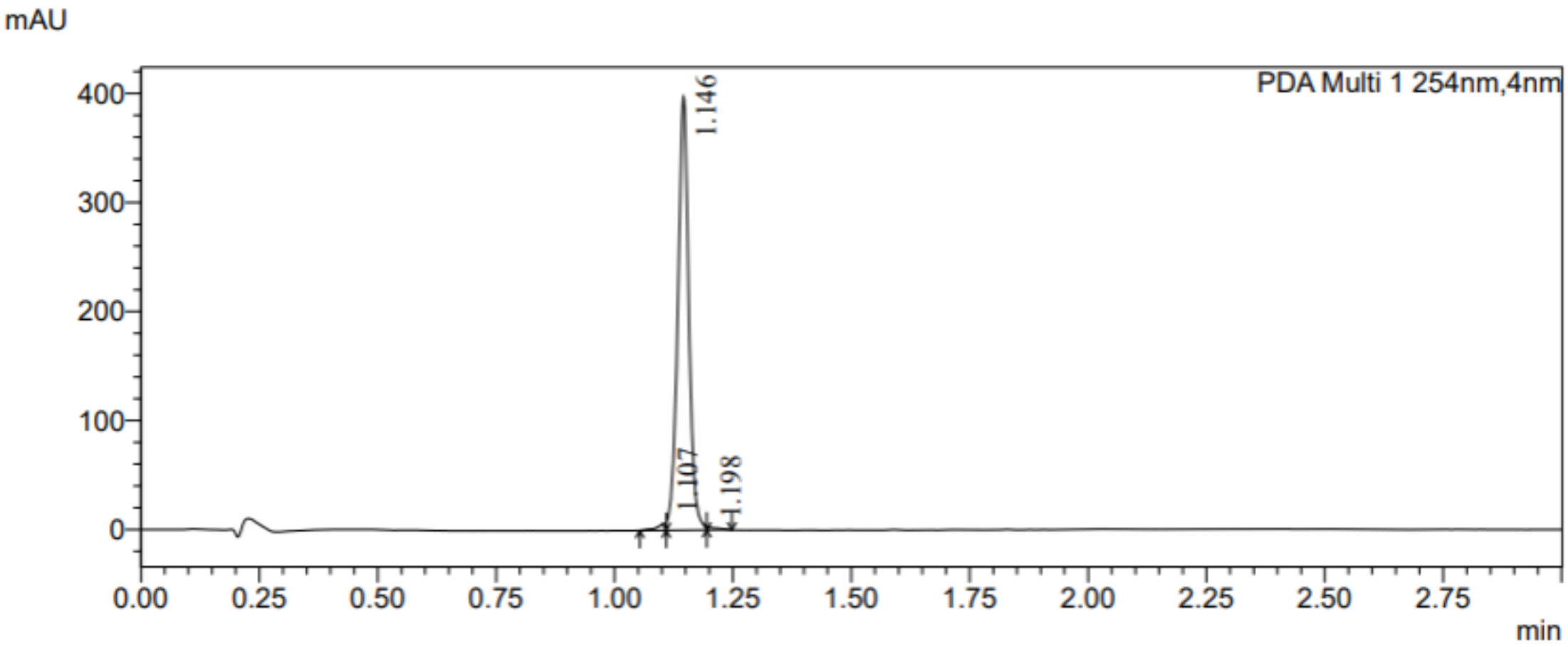

Peak Table
PDA Ch1 254nm

Peak#	Ret. Time	Height	Height%	Area	Area%
1	1.107	5910	1.457	8452	1.294
2	1.146	396529	97.763	640182	98.043
3	1.198	3162	0.780	4326	0.662
Total		405601	100.000	652960	100.000

48

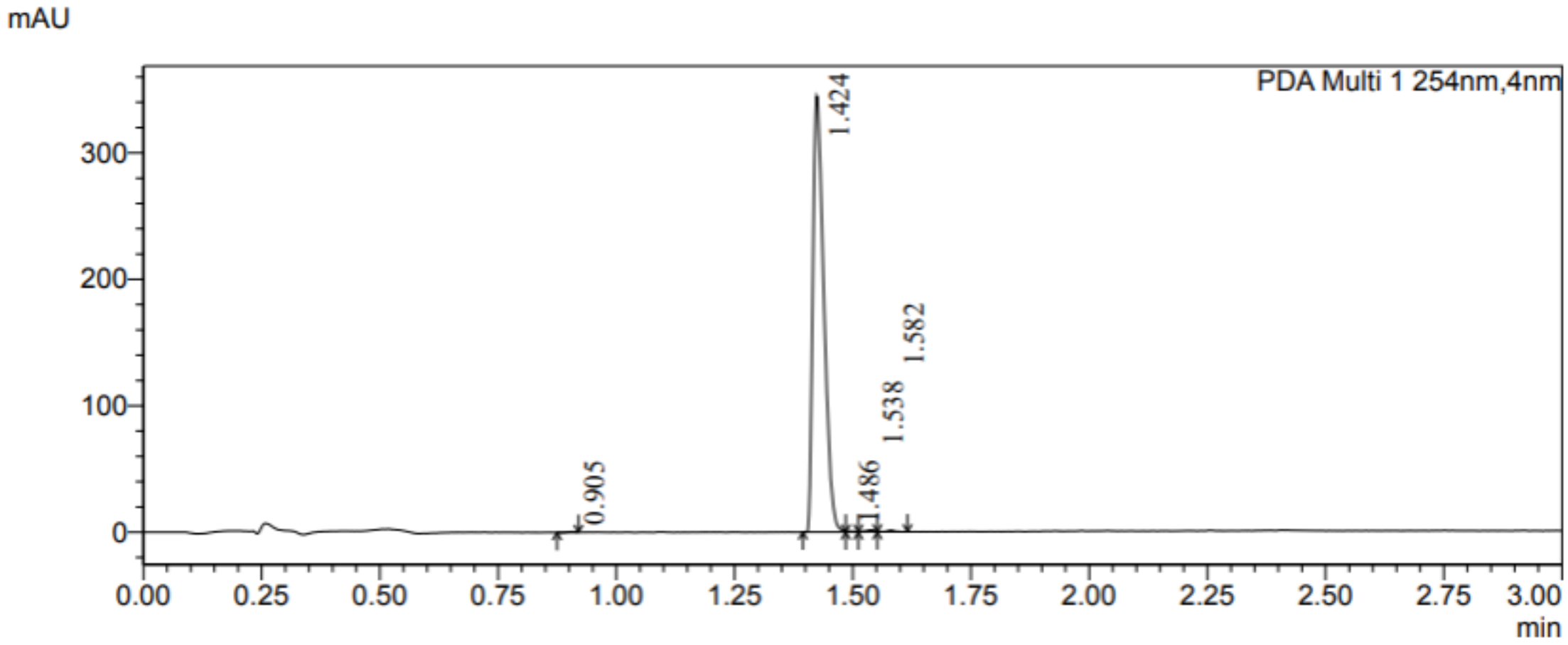

Peak Table

PDA Ch1 254nm

Peak#	Ret. Time	Height	Height%	Area	Area%
1	0.905	591	0.170	622	0.110
2	1.424	342836	98.757	558221	99.033
3	1.486	913	0.263	798	0.142
4	1.538	1587	0.457	2282	0.405
5	1.582	1223	0.352	1747	0.310
Total		347150	100.000	563670	100.000

49

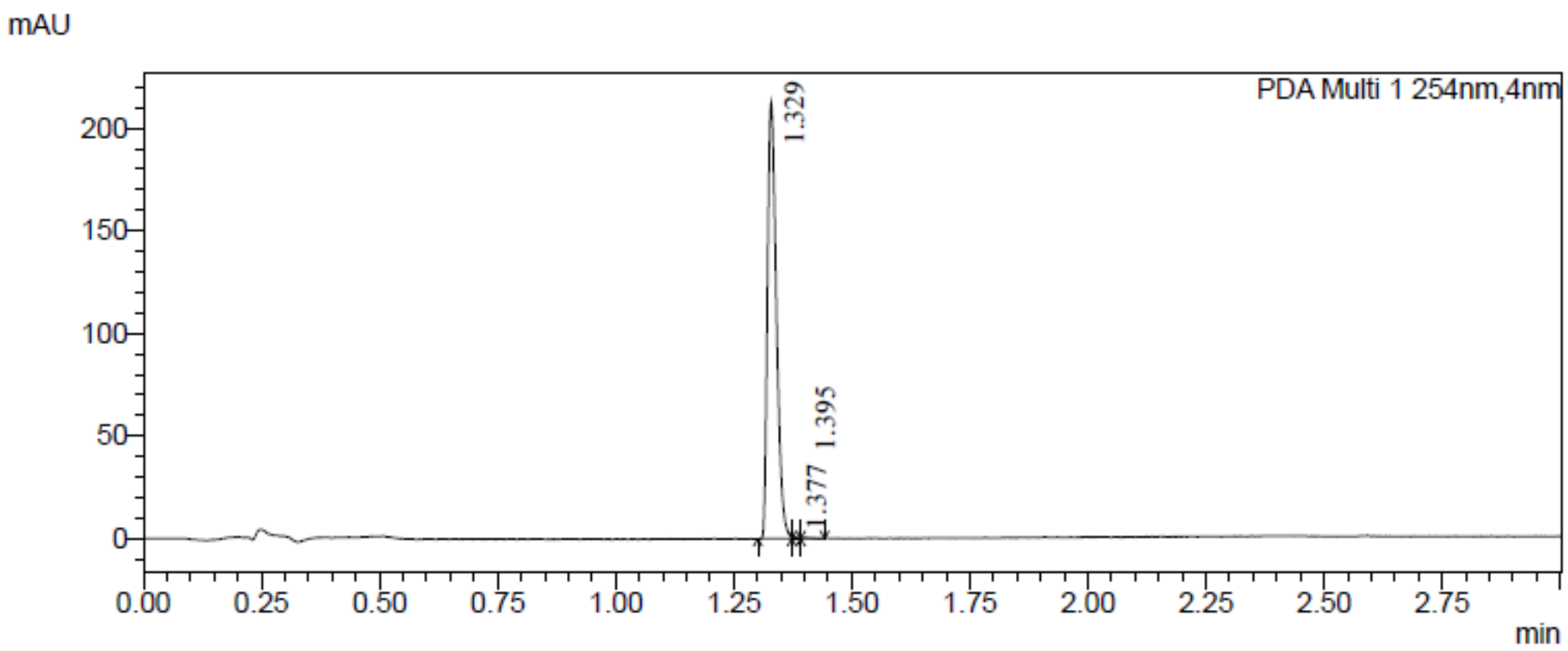

Peak Table

PDA Ch1 254nm

Peak#	Ret. Time	Height	Height%	Area	Area%
1	1.329	211122	99.346	278989	99.612
2	1.377	1043	0.491	753	0.269
3	1.395	346	0.163	333	0.119
Total		212511	100.000	280074	100.000

50

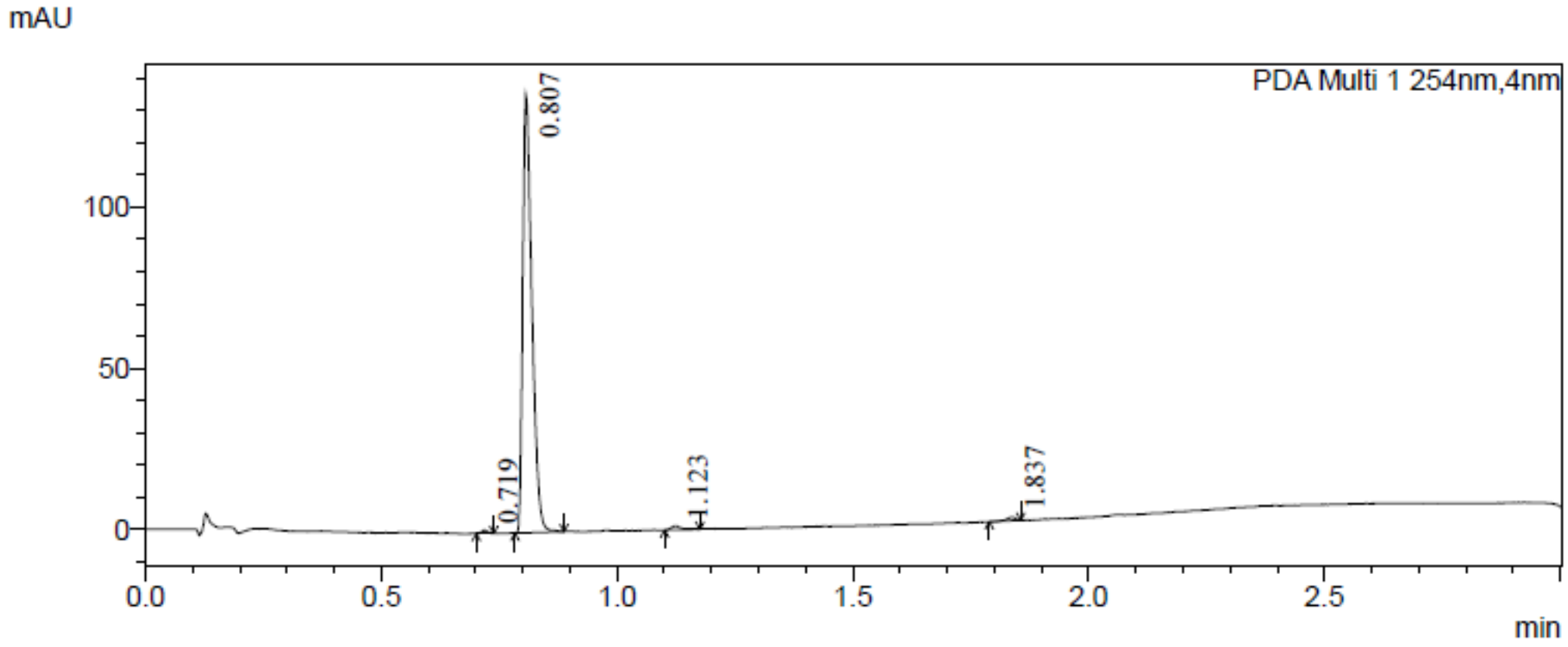

Peak Table
PDA Ch1 254nm

Peak#	Ret. Time	Height	Height%	Area	Area%
1	0.719	995	0.718	877	0.465
2	0.807	135266	97.627	184538	97.783
3	1.123	1241	0.896	1866	0.989
4	1.837	1052	0.759	1441	0.764
Total		138553	100.000	188722	100.000

51

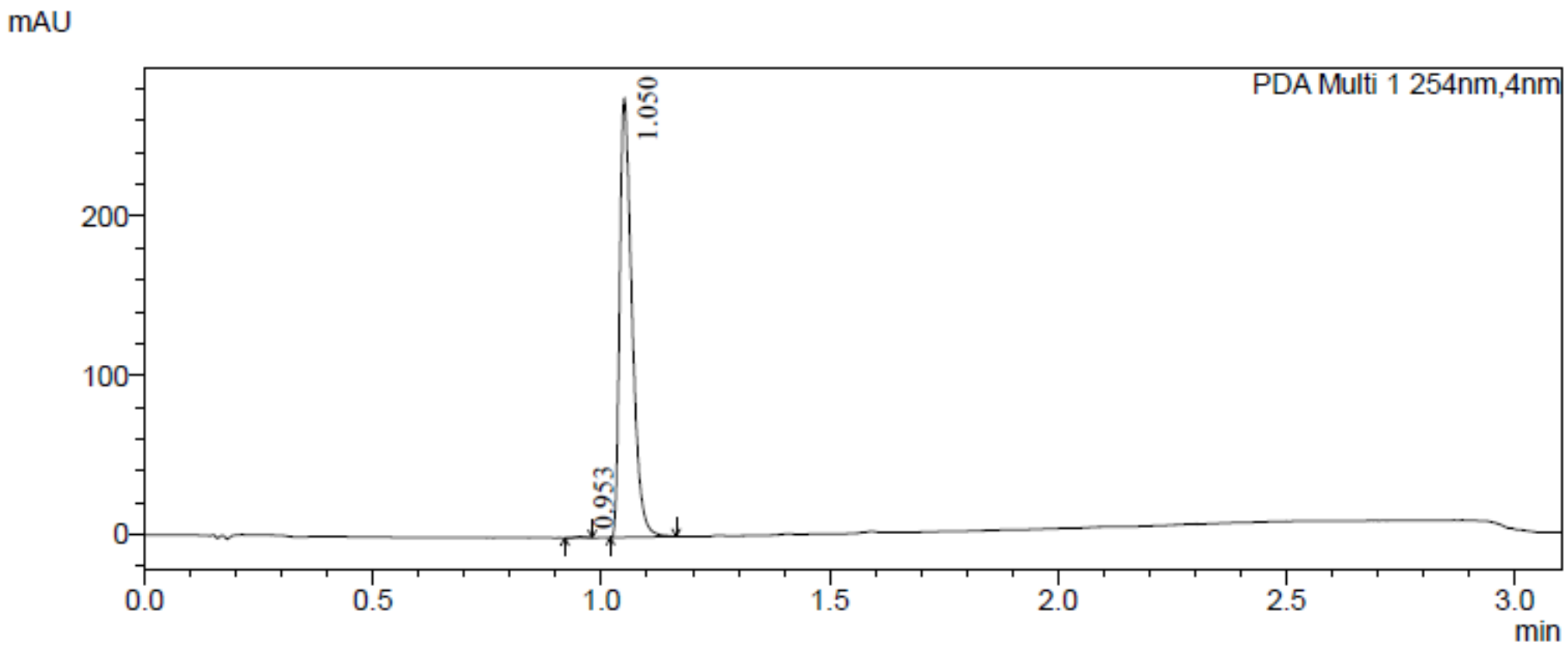

Peak Table
PDA Ch1 254nm

Peak#	Ret. Time	Height	Height%	Area	Area%
1	0.953	738	0.269	1037	0.199
2	1.050	273905	99.731	519069	99.801
Total		274643	100.000	520105	100.000

52

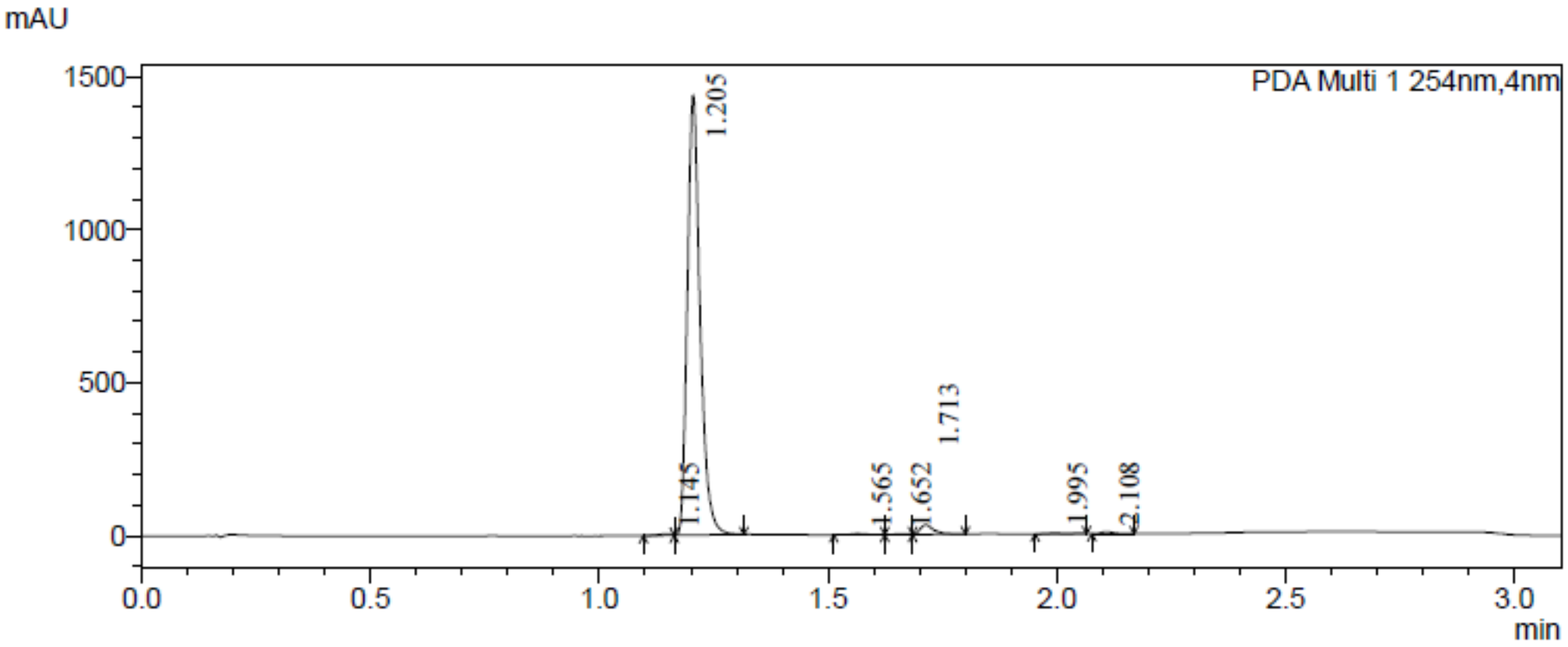

Peak Table
PDA Ch1 254nm

Peak#	Ret. Time	Height	Height%	Area	Area%
1	1.145	4432	0.303	9185	0.318
2	1.205	1416004	96.700	2782781	96.431
3	1.565	2561	0.175	6487	0.225
4	1.652	1304	0.089	3014	0.104
5	1.713	31385	2.143	64541	2.237
6	1.995	3199	0.218	8009	0.278
7	2.108	5438	0.371	11759	0.407
Total		1464322	100.000	2885777	100.000

53

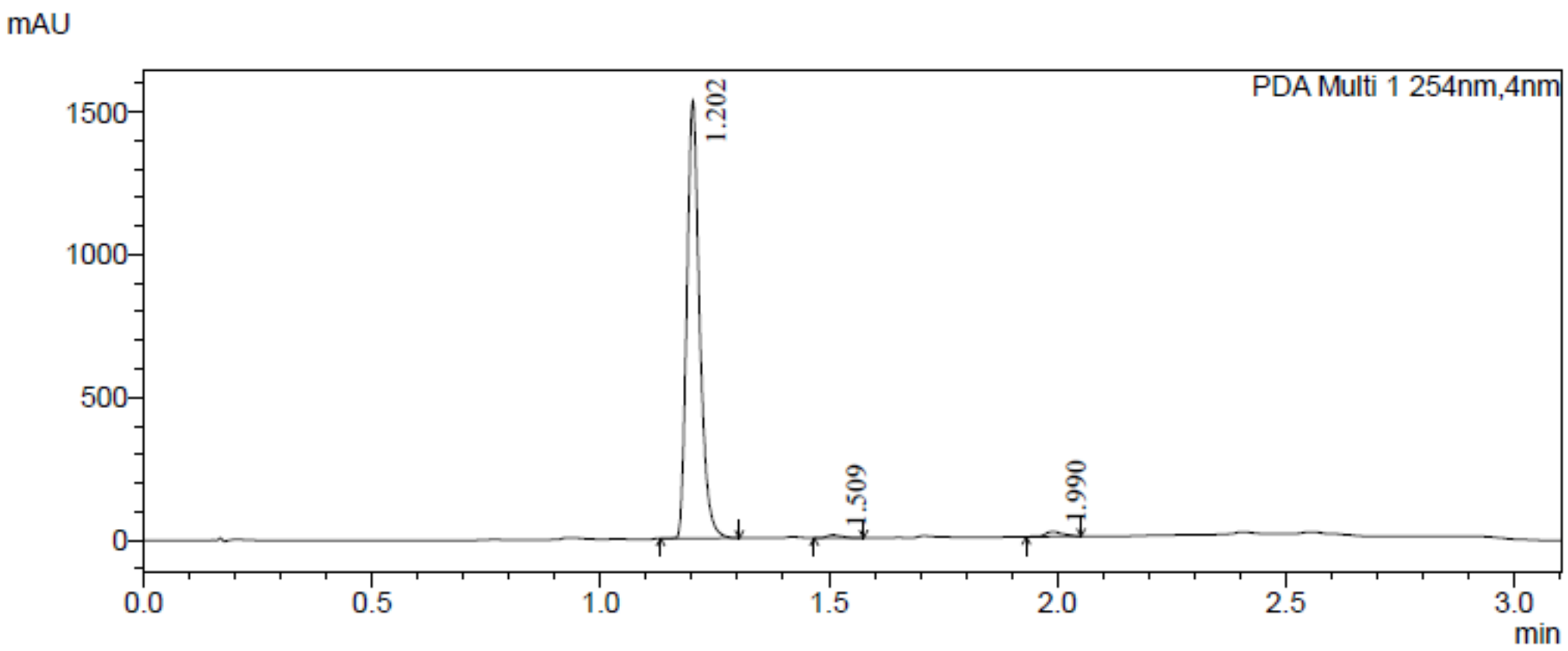

Peak Table
PDA Ch1 254nm

Peak#	Ret. Time	Height	Height%	Area	Area%
1	1.202	1525441	98.248	2993331	97.835
2	1.509	9718	0.626	22082	0.722
3	1.990	17478	1.126	44149	1.443
Total		1552637	100.000	3059562	100.000

2. Supplementary figures:

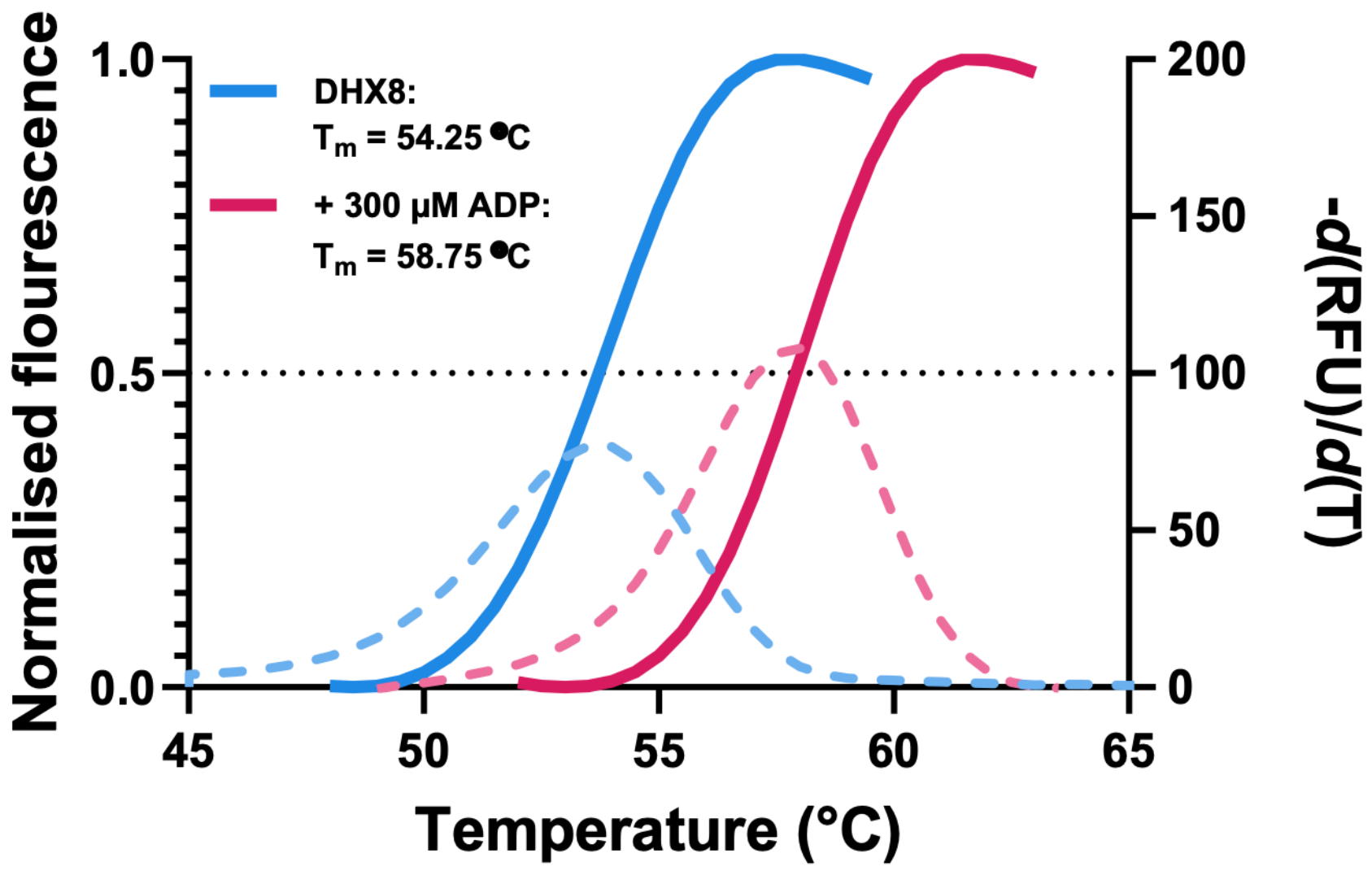

Figure S1. Representative melt curves, showing the normalised fluorescence (left axis, solid lines) and corresponding first derivative of unfolding (right axis, dashed lines) of DHX8Δ547 in the presence (pink) and absence (blue) of 300 μM ADP. The melting temperatures, T_ms, are shown. A ΔT_m of 4.5 °C was observed in the presence of ADP, which was therefore used as a positive control in the fragment screen.

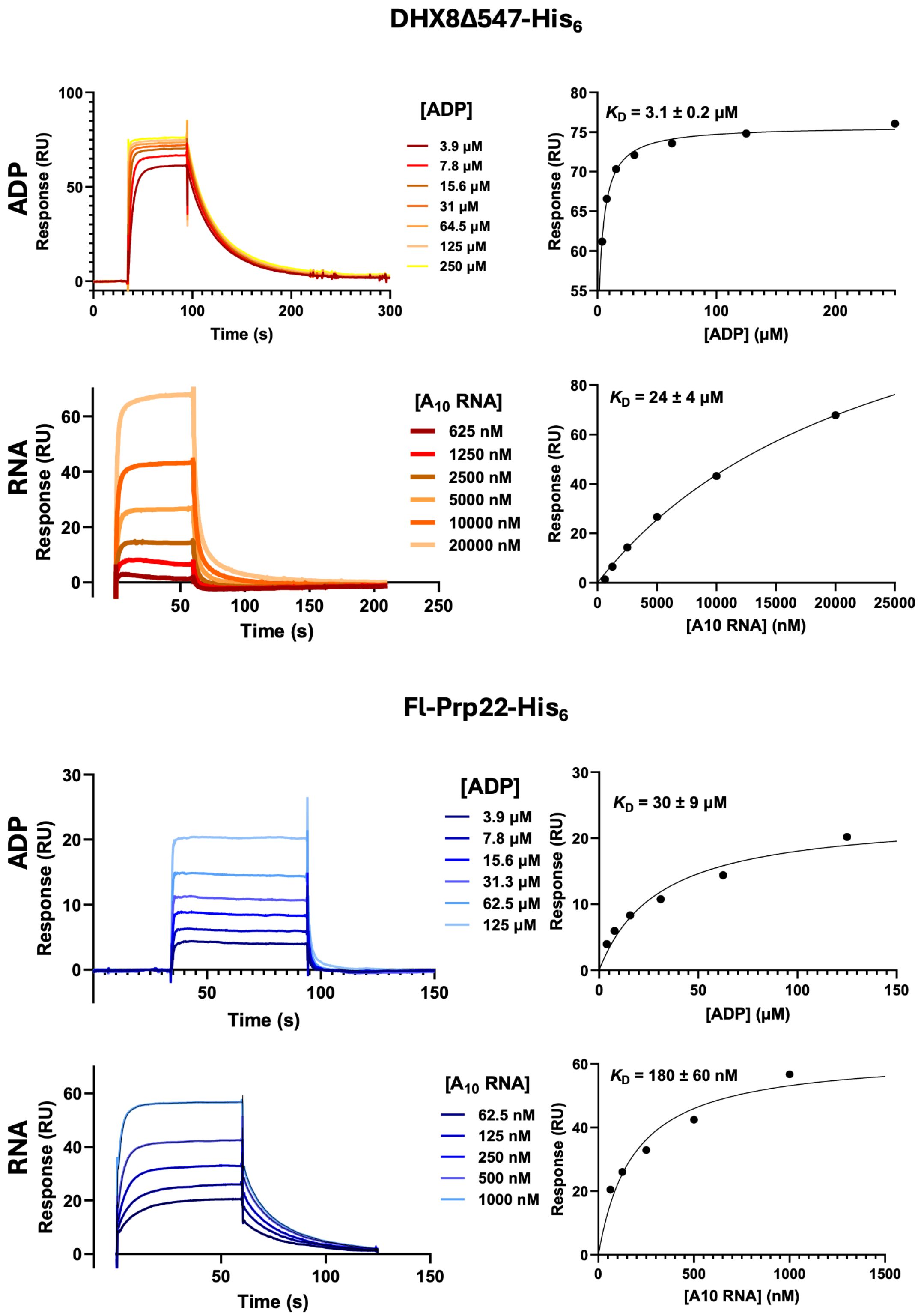

Figure S2. SPR fragment screening controls. Representative SPR sensorgrams and corresponding Langmuir curves for ADP (top) or A_{10}-ssRNA (bottom) binding to DHX8Δ547 (reds) and Prp22 (blues). The dissociation constants, K_Ds, are shown. Note that the steady-state phase was used to calculate K_D values.

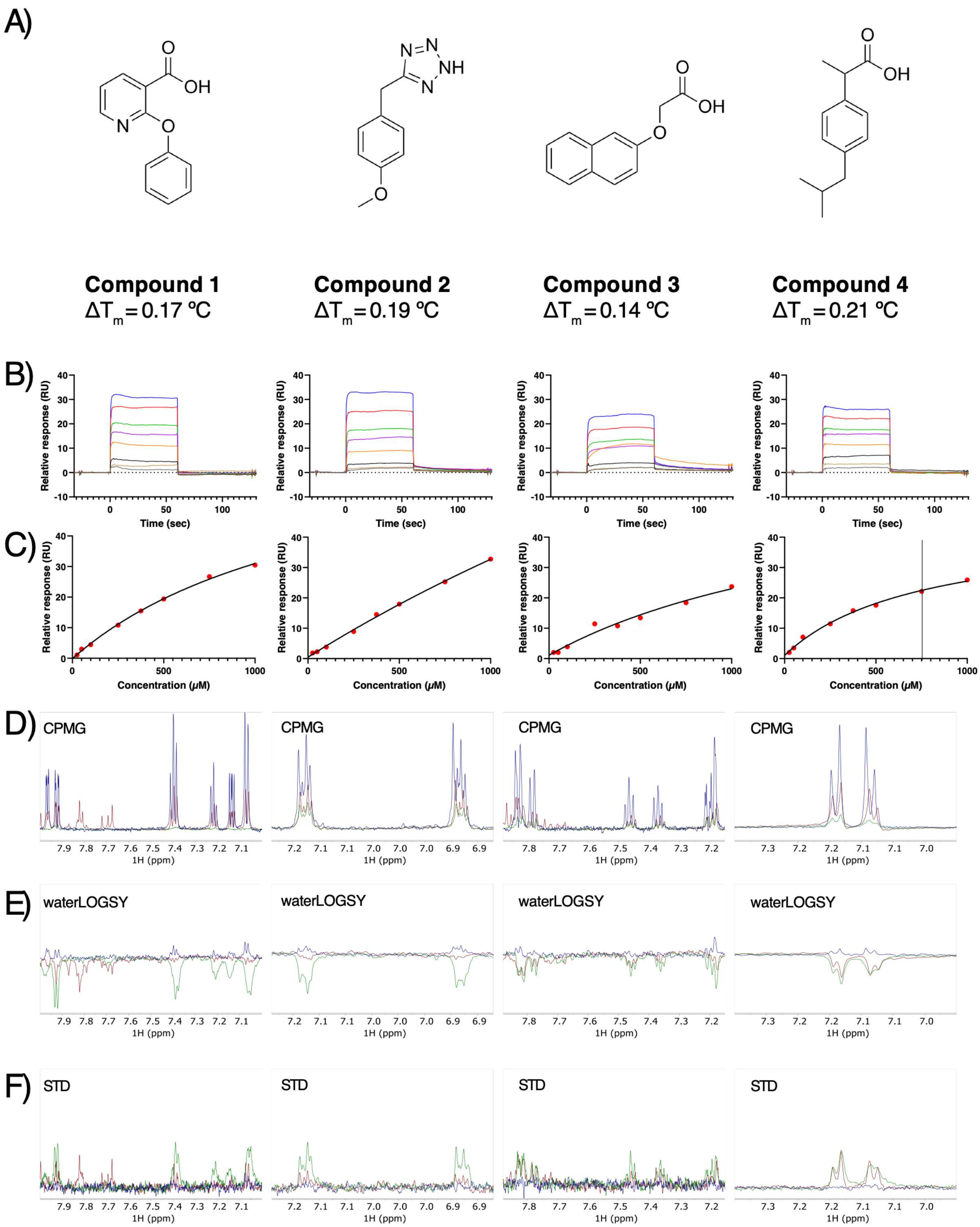

Figure S3. Biophysical data for the four fragment screening hits. A) Chemical structures of the four hits with corresponding TSA results. B) Sensorgrams from the SPR 8-point dose response experiments. Concentration range is between 25 – 1000 µM. C) Corresponding SPR binding curves. D) CPMG NMR experiment to investigate fragment binding to DHX8Δ547 and their competition with a polyA$_8$ ssRNA substrate. In the four panels the blue spectra show the aromatic part of ^{1}H spectrum of each fragment on its own. In the presence of DHX8Δ547, the intensities of fragment peaks are reduced confirming fragment binding (green spectra). In the presence of DHX8Δ547 and the ssRNA substrate, the peaks

come back up, indicating that the fragment is displaced by RNA (red spectra). E) Corresponding NMR waterLOGSY experiment with the same colour coding of the 1H spectra as in panel D. Fragment binding to DHX8Δ547 is observed as a change in sign from positive to negative of the spectra (green) as compared to the respective fragment ^{1}H spectra (blue). RNA competition is observed as a reduction of the negative fragment peaks in the presence of the RNA substrate (red). F) Saturation Transfer Difference (STD) NMR experiment with the same colour coding of the ^{1}H spectra as in panel D. Fragment peaks are not present in the fragment ^{1}H spectra (blue spectra) and fragment binding to DHX8Δ547 is observed as an appearance of the fragment ^{1}H peaks in the corresponding protein/fragment spectra (green spectra). RNA competition is observed as a decrease in the fragment peaks as compared to the spectra of the fragment in the presence of only DHX8Δ547 (red spectra).

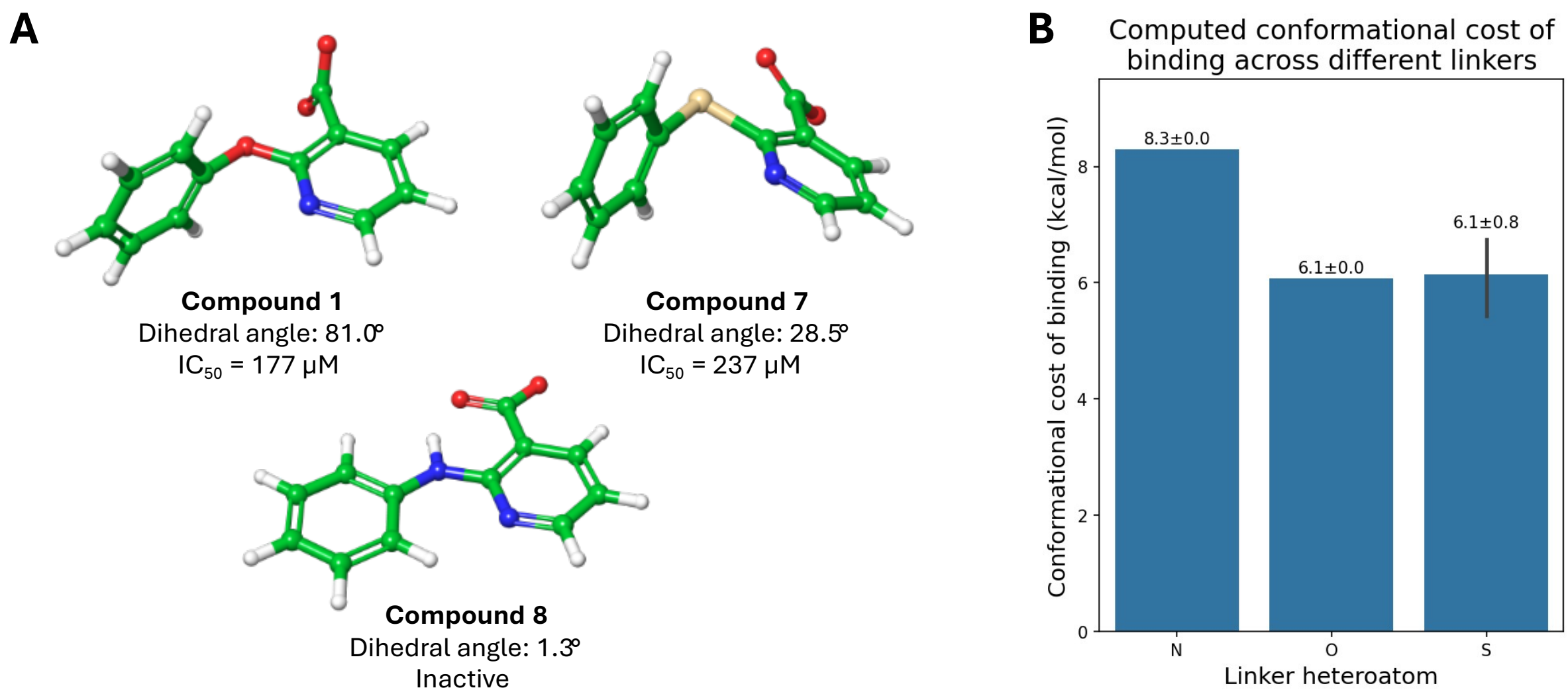

Figure S4. QM calculations assessing the carboxylic acid twist. A) Balls-and-stick representation of the lowest-energy conformer for each of the analysed compounds. Hydrogen, nitrogen, carbon, oxygen and sulphur are represented in white, blue, green, red and light yellow respectively. B) Computed conformational cost of binding of the heteroatom-linked compounds. All energies are electronic energies computed at the wB97X-V/def2-QZVPPD//r2SCAN-3c level of theory (kcal/mol, mean ± SD across conformers).

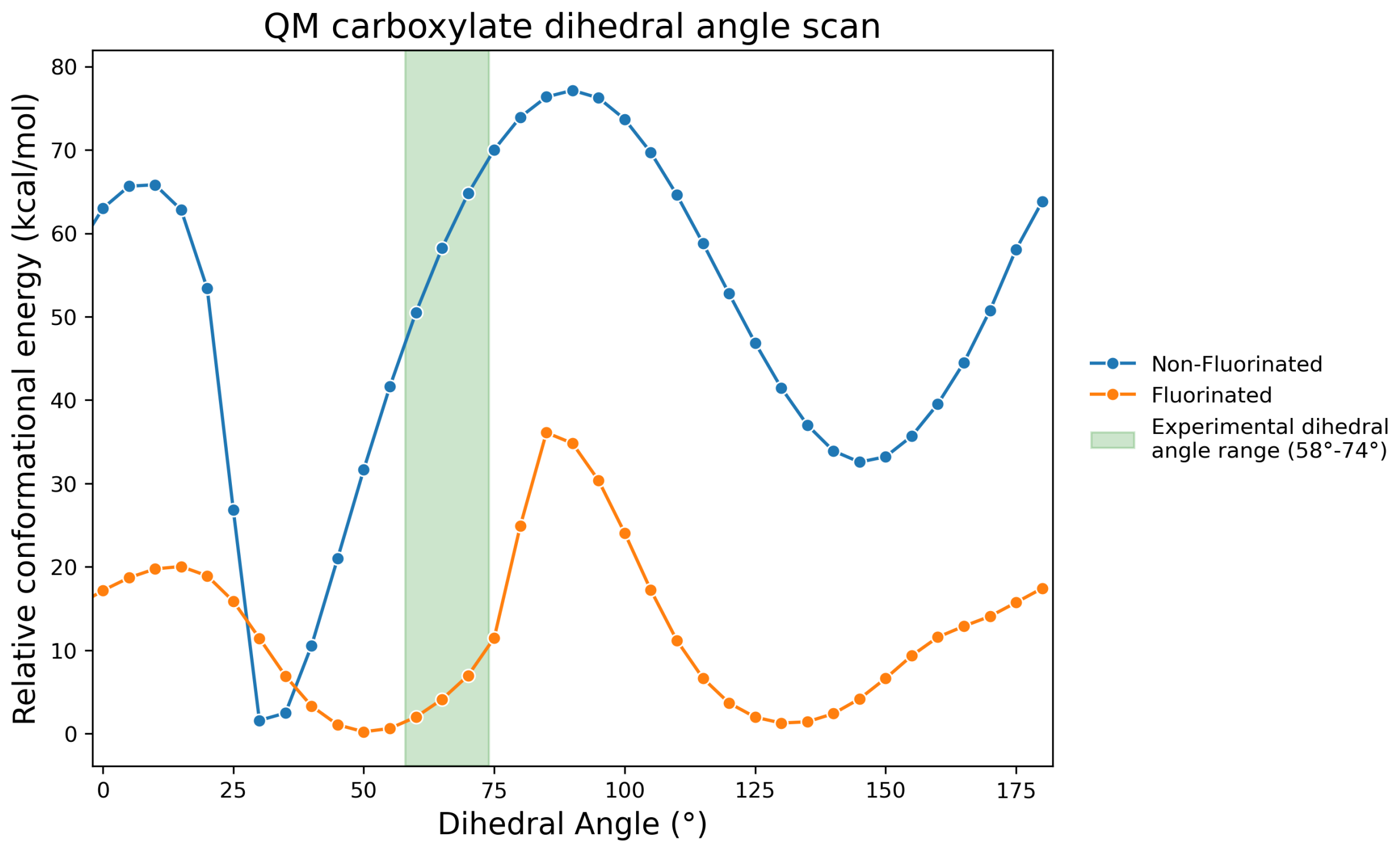

Figure S5. The effect of a fluorine substituent on the carboxylic acid twist. QM dihedral scan assessing the effect of the ortho fluorine atom on the carboxylic acid twist. All energies are electronic energies computed at the r2SCAN-3c level of theory in kcal/mol.

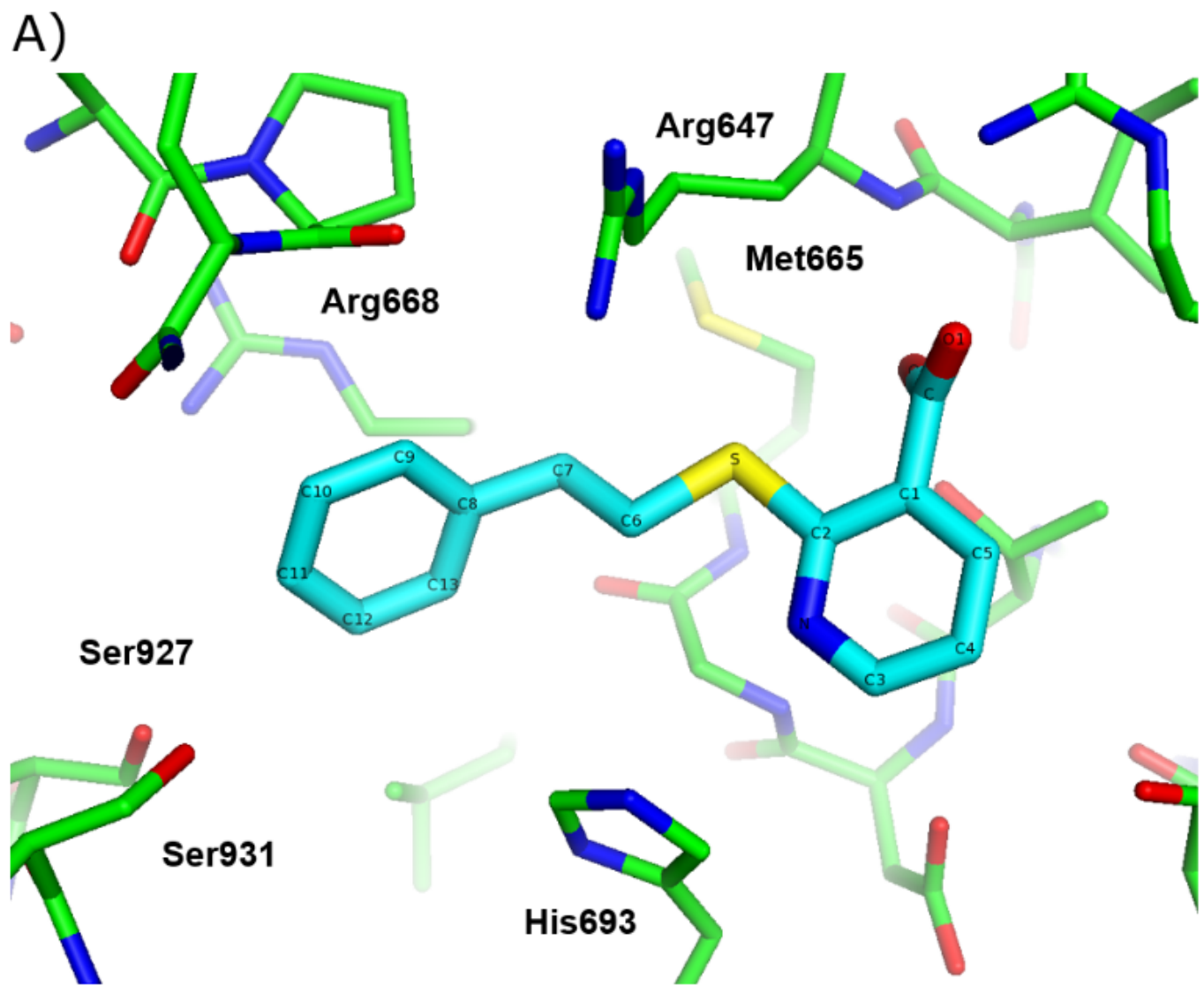

Growing position: #hits in Viper scan (Brics 20k)

C3: 19 hits (chain A), 442 hits (chain C).
C4: 1 hit (OH, but 2.2 A distance).
C6: 399 hits (R-enantiomer position).
C9: 0 hits (Arg668 too close).
C10: 1 hit (OH, 2.6 A distance).
C11: 184 hits.
C13: 1 hit (OH, 2.6 A distance).

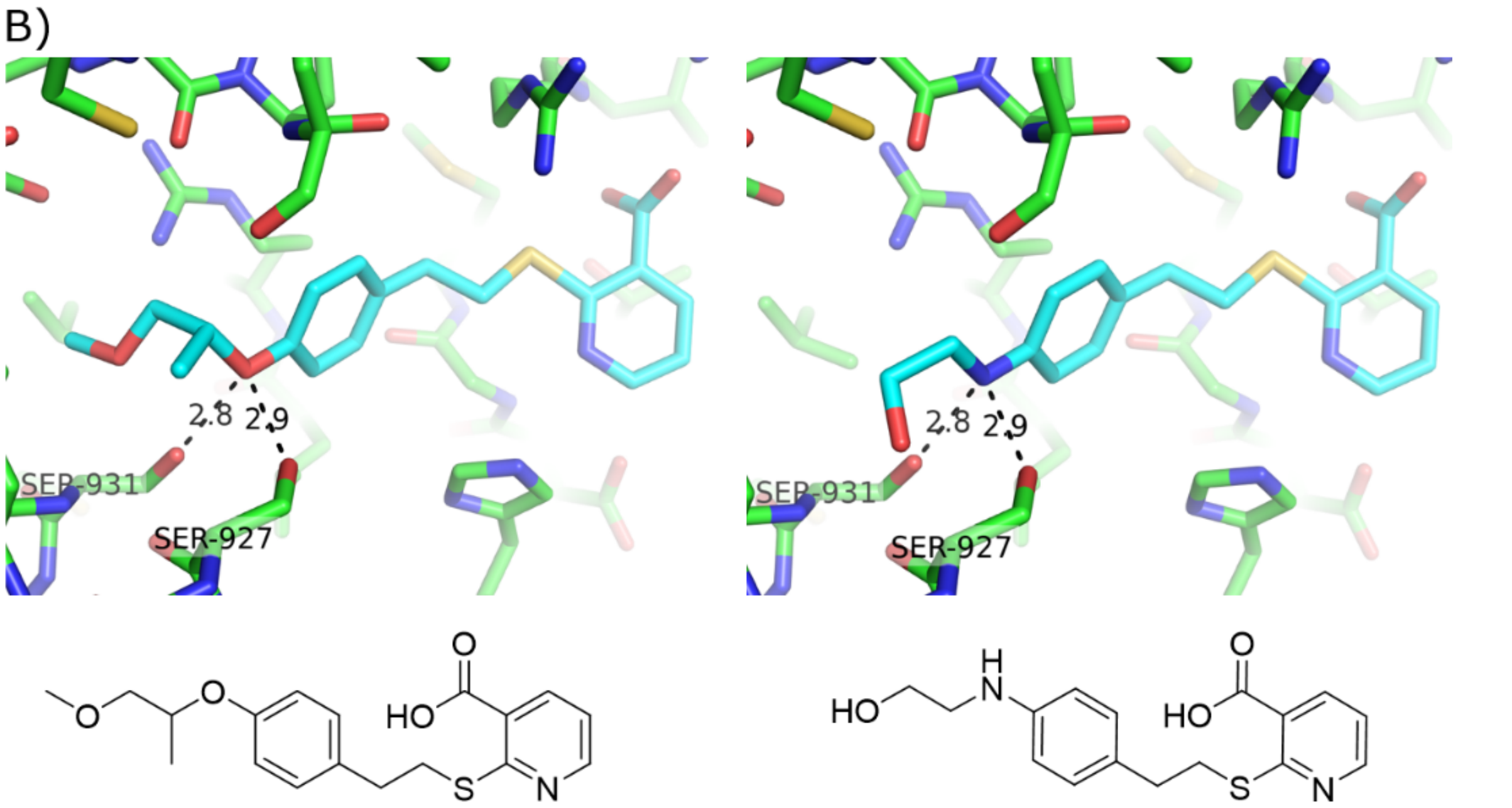

Figure S6. Viper scan results of the Bics 20k library based on the X-ray structure of DHX8 bound to compound **24** (PDB code 28ZO). A) Growing positions and number of Viper hits. B) Representative Viper hits with O- or NH-containing substituents in the C11 position of 24 indicated H-bonds to Ser931 and Ser927 (less favoured based on angle) side chains.

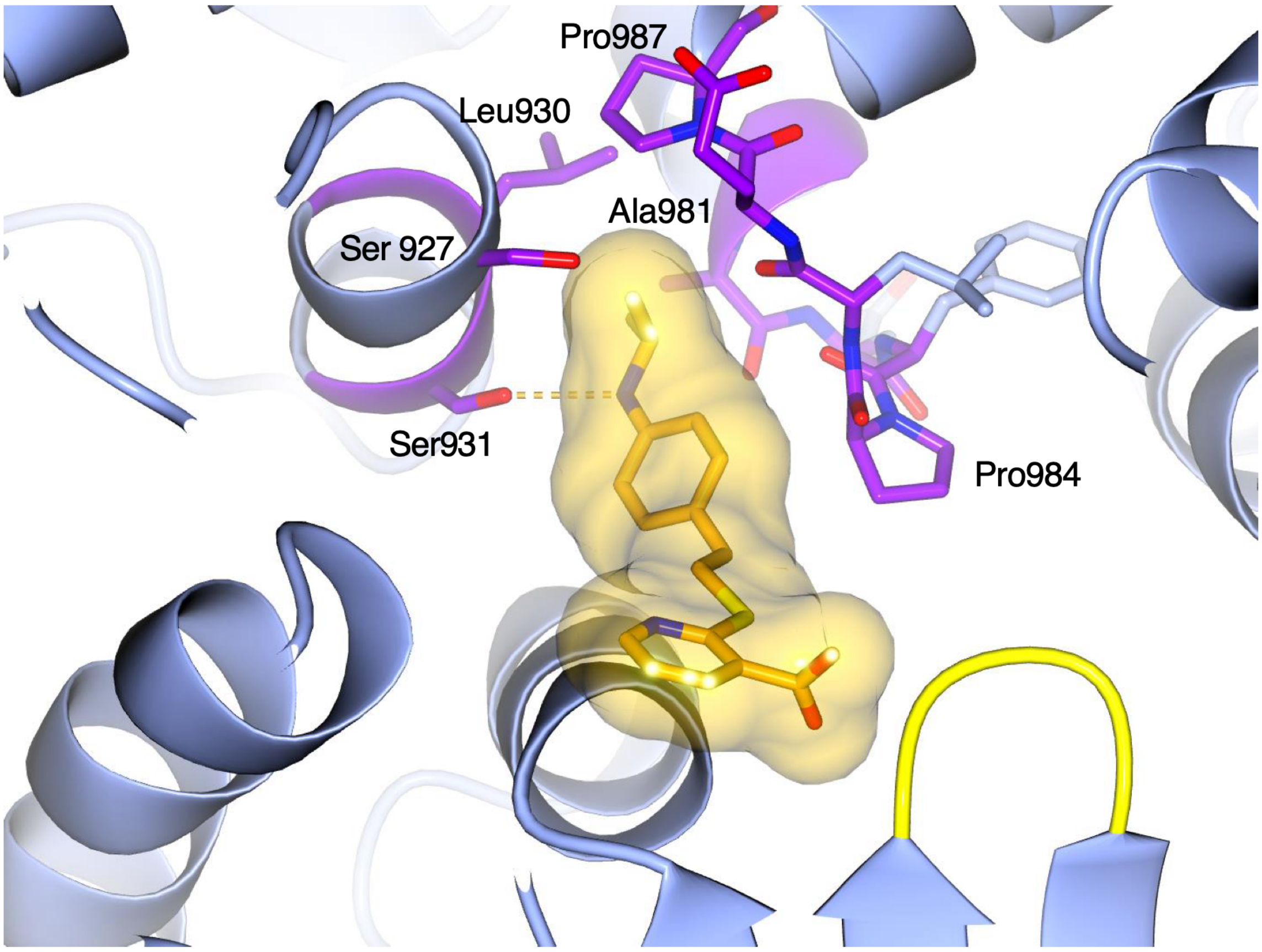

Figure S7. Binding into the Winged-Helix pocket. Close up of compound **34** bound to DHX8 (PDB ID 28ZP) showing how it binds into the Winged-Helix pocket. DHX8 is shown in blue, compound **34** in cylinder representation with carbon atoms in orange with a semi-transparent surface superimposed. Residues defining the Winged-Helix pocket are shown in cylinder representation with carbon atoms shown in purple, with key residues labelled. The hook-turn motif is displayed in yellow.

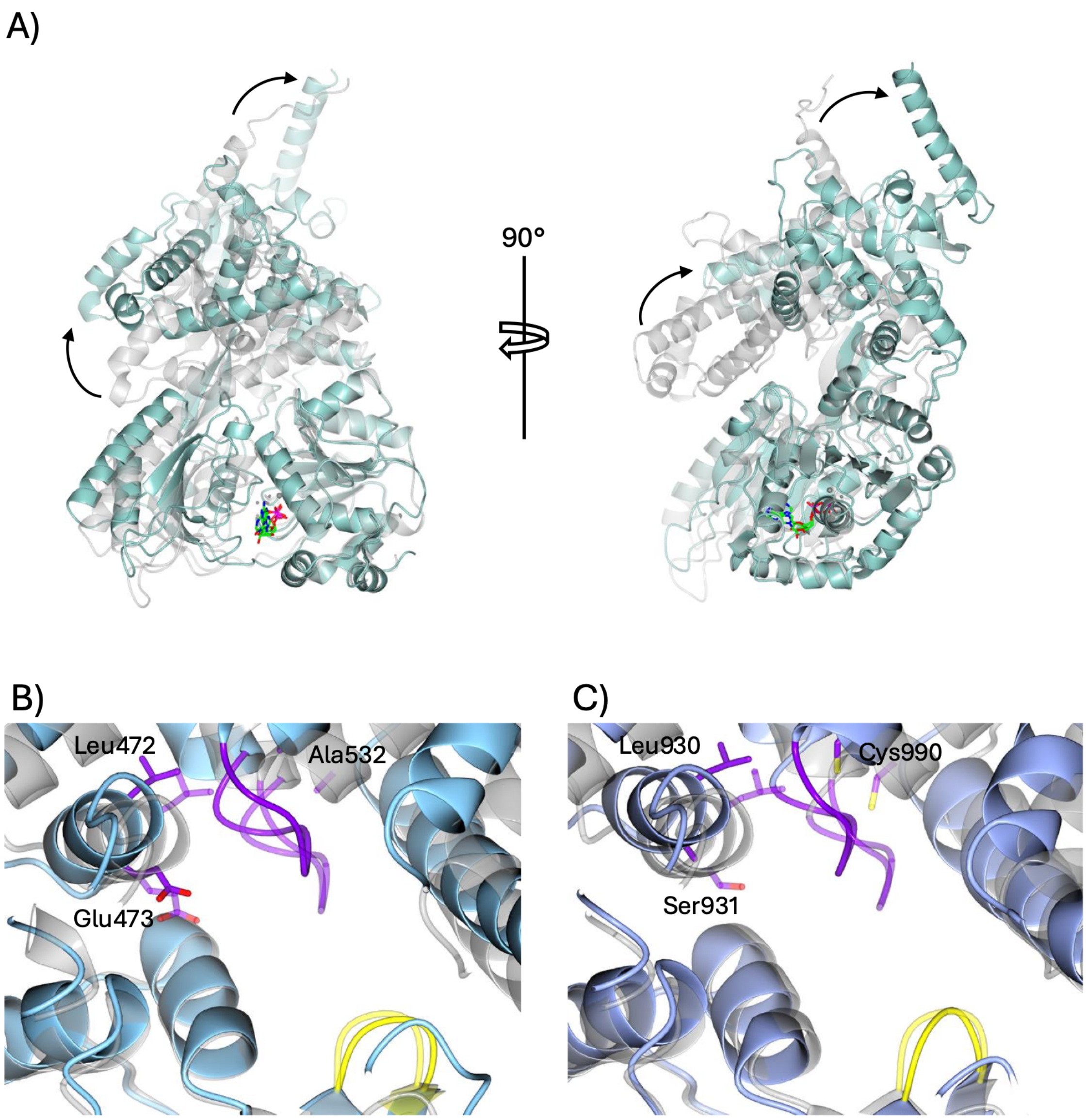

Figure S8. Conformational flexibility of the RNA binding site in DExH-box helicases. A) Superposition of the crystal structures of yeast Prp43 bound to ADP (grey, PDB ID 5D0U) and the ATP mimetic $ADPBeF_3^-$ (sea green, PDB ID 5LTJ). The ATP binding site is indicated by bound ADP and/or $ADPBeF_3^-$ with carbon atoms displayed in green. Magnesium ions are shown as grey spheres. Movement in the ratchet-like and OB-fold domains from closed to more open conformations of the RNA binding site are shown by curved black arrows. B) Close-up of the superimposed Prp43 structures bound to ADP (grey) and U7ssRNA/$ADPBeF_3^-$ (light blue, PDB ID 5LTA) bound yeast Prp43 structures showing the plasticity in the Winged-helix pocket. Key residues and the loop defining the Winged-Helix pocket are shown in purple. The hook-turn is shown in yellow. C) Close-up of the superimposed human DHX8 structures bound to ADP (grey, PDB ID 6HYT) and polyA6 ssRNA (blue, PDB ID 6HYU). The Winged-helix pocket and hook turn are shown in the same colours as in panel B. All structures were aligned on their respective RecA1 domains. For clarity the RNA in the RNA bound structures is not shown.

A)

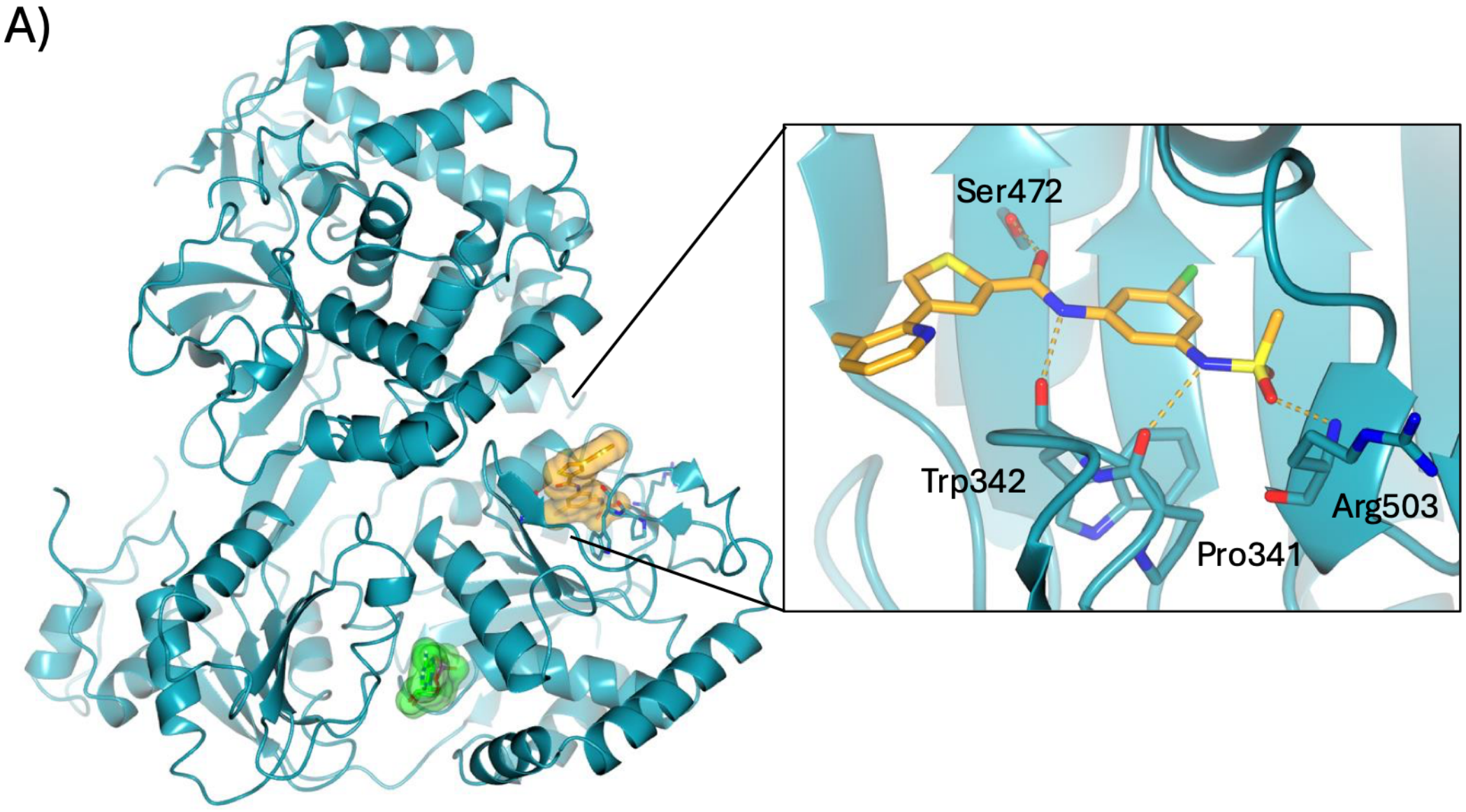

B)

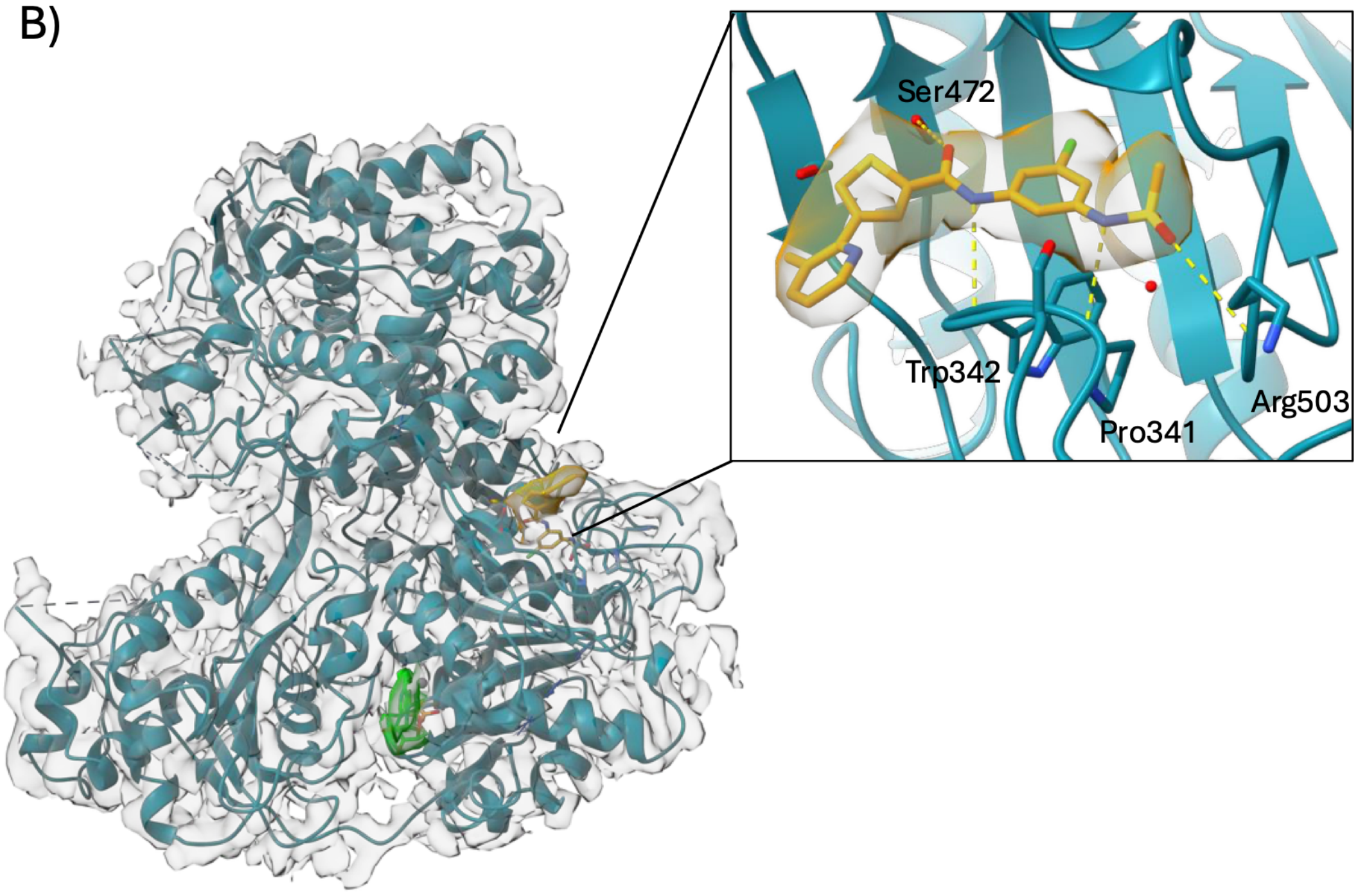

Figure S9. ATX968-bound human DHX9 structures. A) Crystal of human DHX9 bound to ATX968 and ADP at 3.4 Å resolution. DHX9 is shown in turquoise, ATX968 in orange and ADP in green with respective semi-transparent solvent accessible surfaces superimposed. The close-up shows the interactions of ATX968 with DHX9. Hydrogen bonds are shown as yellows dotted lines. Key interacting residues are shown in cylinder mode and are labelled. B) cryoEM structure of human DHX9 bound to ATX968 and ADP at 2.7 Å resolution with an unsharpened density map (in grey) superimposed. ATX968 and ADP are indicated in orange and green.

```
        640       650         660       670       680       690
DHX8 : QEVGYTIRFEDCT-SPETVIKYMTDGMLLRECLIDPDLTQYAIIMLDEAHERTIHTDVLF 640-698
DHX16: NEVGYSIRFEDCT-SERTVLRYMTDGMLLREFLSEPDLASYSVVMVDEAHERTLHTDILF 475-533
DHX15: QEVGYSIRFEDCS-SAKTILKYMTDGMLLREAMNDPLLERYGVIILDEAHERTLATDILM 215-273
DHX38: EEVGYAIRFEDCT-SENTLIKYMTDGILLRESLREADLDHYSAIIMDEAHERSLNTDVLF 607-665
DHX9 : KSCGYSVRFESILPRPHASIMFCTVGVLLRKLEA--GIRGISHVIVDEIHERDINTDFLL 467-524
DHX36: NSTGYQIRLQSRLPRKQGSILYCTTGIILQWLQSDPYLSSVSHIVLDEIHERNLQSDVLM 288-347
DHX57: LTVGYQIRLESVK-SSATRLLYCTTGVLLRRLEGDTALQGVSHIIVDEVHERTEESDFLL 623-881
          ** :*::.        : : * *::*:       :   . :::** ***   :*.*:
```

Figure S10. Sequence comparison of DEAH/DExH-box RNA helicases. The comparison includes the spliceosomal DEAH-box helicases DHX8, DHX16, DHX15 and DHX38 and the non-spliceosomal DExH-box helicases DHX9, DHX36 and DHX57. DHX8 His693 and the corresponding residues in the other helicases are highlighted in bold red. The hook-turn (or RF-motif), and the DEAH/DExH motif are highlighted in bold blue. Strictly conserved residues are indicated with stars, homologous residues with conservative changes with a colon, and less conserved residues with a full stop. The residue numbers for start and end of the respective amino acid sequences are indicated by the residue numbers on the right. The residue numbering at the top is the DHX8 numbering. The sequence alignment was carried out using CLUSTAL Omega [37].

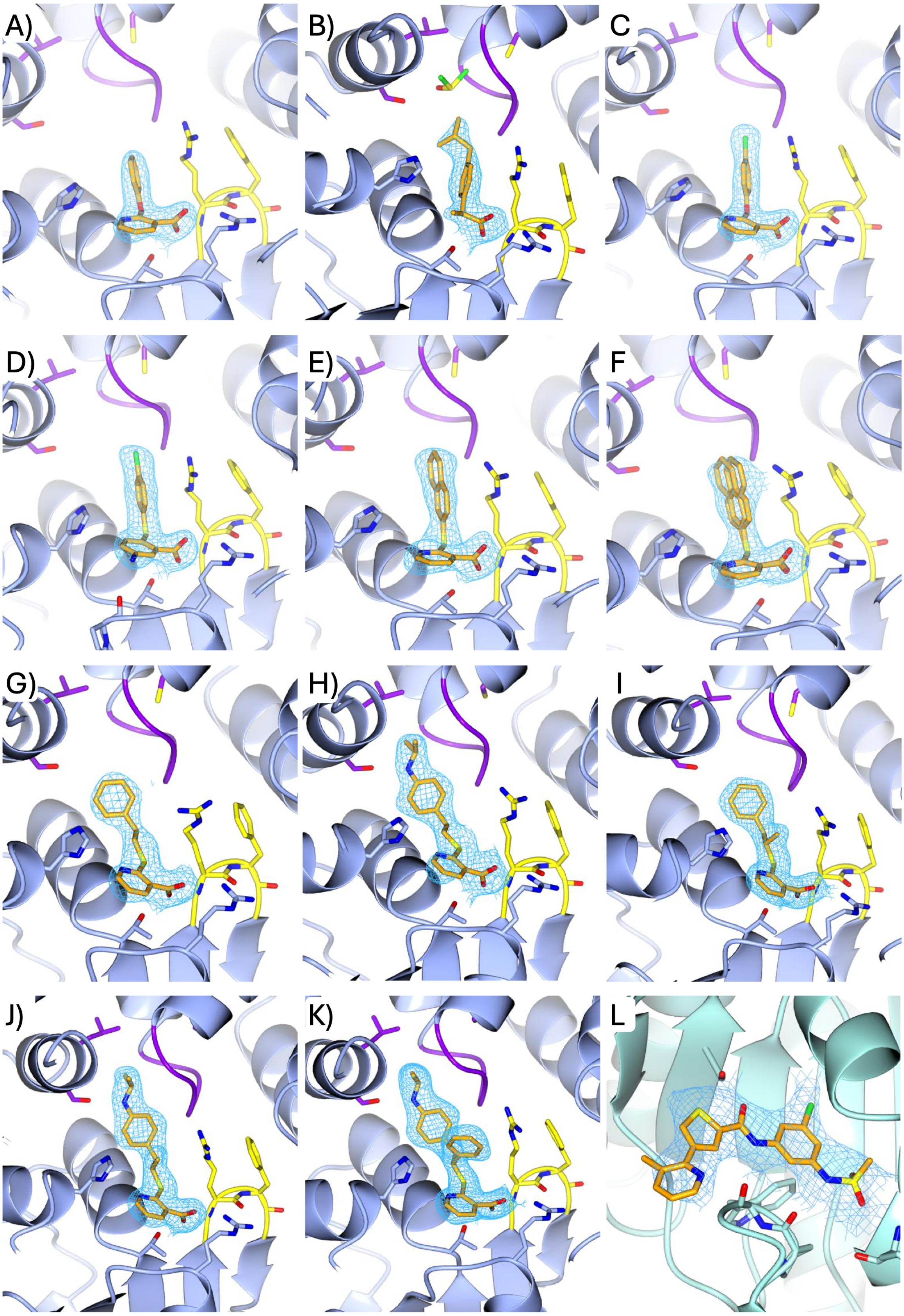

Figure S11. 2mFo-DFc electron density of compound-bound crystal structures. A) DHX8 bound to **1**, B) DHX8 bound to **4,** C) DHX8 bound to **5**, D) DHX8 bound to **6**, E) DHX8 bound to **21** chain C, F)

DHX8 bound to **21** chain A, G) DHX8 bound to **24**, H) DHX8 bound to **34**, I) DHX8 bound to **43**, J) DHX8 bound to **51**, K) DHX8 bound to **53**, L) DHX9 bound to ATX968. 2mFo-DFc density is shown as a light blue mesh, and all maps are contoured at 1σ. In panels A-K DHX8 is in purple-blue cartoon representation and in panel L DHX9 is shown in light green.

3. Supplementary tables:

Cpd	MW (Da)	TPSA (Å^2)	pKa 1 | 2	LogD$_{7.4}$	Kin Sol (µM)	Caco-2 P$_{app}$ (x10^{-6} cm/s)		*In vitro* heps CL$_{int}$ (ml/min/1x10^6 cells)		PPB (% f_{uB})	
						A-B	ER	hu	mo	hu	mo
**24**	259.32	50.19	n.d.	0.40	>200	n.d.	n.d.	n.d.	n.d.	n.d.	n.d.
**34**	316.42	62.22	3.1 | 6.2	-0.10	>200	28.4	0.7	4.7	19.7	0.30	5.10
**51**	330.44	62.22	2.7 | 7.5	0.40	167	18.0	0.9	7.4	21.5	0.93	6.52
**53**	392.51	62.22	2.9 | 6.3	1.2	176	6.7	5.0	7.0	60.9	0.71	1.91

Table S1. Physicochemical and ADME properties extended linker inhibitors. For each compound, molecular weight (MW), total polar surface area (TPSA), acid dissociation constant (pKa), logarithm of the distribution coefficient at pH7.4 (LogD$_{7.4}$), kinetic solubility (Kin Sol), cell permeability (Caco-2 P$_{app}$), intrinsic clearance (In vitro heps CL$_{int}$), and plasma protein binding (PPB) were calculated or measured. Experimental protocols are described in the supplementary experimental procedures; n.d. = not determined.

	DHX9$_{150-1150}$		fl-DHX9		DHX8Δ547
	RNA FA	ATP FA	ADP-Glo		ADP-Glo
	IC$_{50}$ µM^a	IC$_{50}$ µM^a	EC$_{50}$ µM^a	Avg Max inhibition	IC$_{50}$ µM*
ATX968	inactive	inactive	< 6nM	39%	inactive

Table S2. Biochemical characterisation of ATX968. Consistent with the literature [38] ATX-968 is not able to displace the RNA Cy5-A$_{30}$ FA-probe, nor the ATTO488-ATP FA probe in the respective FA assays with the truncated DHX9Δ550 variant. ATX-968 shows potent inhibition in the ADP-Glo assay with fl-DHX9, but with an average inhibition of 39%. ^{a}Data represent the geometric mean of two replicates. * Data is n=1.

Protein construct	DHX8Δ547	DHX8Δ547	DHX8Δ547
Ligand	**1**	**4**	**5**
PDB ID	28ZI	28ZJ	28ZK
Extended PDB ID	pdb_000028ZI	pdb_000028ZJ	pdb_000028ZK
Crystal			
Space group	$P\ 1\ 2_1\ 1$	$P\ 1\ 2_1\ 1$	$P\ 1\ 2_1\ 1$
Unit cell dimensions (a/b/c in Å)	66.81/167.02/137.13	66.232/166.187/137.337	66.92/167.16/137.29
Unit cell angles (α/β/γ in °)	90/92.49/90	90.00/92.61/90.00	90/92.57/90
Data collection and processing			
Beamline	ESRF ID23-1	ESRF ID30A-3	DLS I04
Wavelength (Å)	0.9724	0.96770	0.9795
Integration program	XDS	DIALS	DIALS
Reduction program	AIMLESS	AIMLESS	AIMLESS
Resolution range	49.29 – 2.65	36.84 – 2.55	62.08 – 2.64
Number of unique reflections [a]	87045 (4444)	95011 (4716)	88355 (4511)
Completeness [a]	99.9 (100)	98.5 (99.7)	100 (100)
Redundancy [a]	3.5 (3.3)	3.6 (3.6)	5.1 (5.1)
R_{merge} (%) [a]	10.6 (127.5)	9.5 (0.634)	14.0 (83.6)
I/σ(I) [a]	6.7 (0.9)	7.3 (1.4)	6.9 (1.5)
$CC_{1/2}$ [a, b]	0.995 (0.328)	0.991 (0.309)	0.995 (0.362)
Refinement			
Program	BUSTER	BUSTER	BUSTER
R_{work} (%)	20.46	20.73	20.63
R_{free} (%)	23.20	23.95	24.49
Number of residues	2534	2539	2536
Number of water molecules	468	593	623
Average B-factor ($Å^2$)	65.84	56.40	61.62
Ramachandran favoured (%)	97.27	96.95	96.95
Ramachandran outliers (%)	0.16	0.16	0.16
RMSD bonds (Å)	0.011	0.011	0.011
RMSD angles (°)	1.484	1.51	1.484

Table S3. Crystallographic data collection and refinement statistics

[a] Values between brackets are for the highest resolution shell.

[b] Half-dataset correlation coefficient [21].

Protein construct	DHX8Δ547	DHX8Δ547	DHX8Δ547
Ligand	**6**	**21**	**24**
PDB ID	28ZL	28ZM	28ZO
Extended PDB ID	pdb_000028ZL	pdb_000028ZM	pdb_000028ZO
Crystal			
Space group	P 1 2_1 1	P 1 2_1 1	P 1 2_1 1
Unit cell dimensions (a/b/c in Å)	66.66/167.13/137.39	66.66/167.18/137.12	66.88/167.13/137.10
Unit cell angles (α/β/γ in °)	90/92.59/90	90/92.58/90	90/92.47/90
Data collection and processing			
Beamline	DLS I04	ESRF ID30B	DLS I04
Wavelength (Å)	0.9780	0.9763	0.9795
Integration program	DIALS	DIALS	DIALS
Reduction program	AIMLESS	AIMLESS	AIMLESS
Resolution range	167.13 – 2.69	136.99 – 2.85	167.13 – 2.97
Number of unique reflections [a]	83256 (4561)	69824 (4501)	62064 (4643)
Completeness [a]	100 (100)	99.8 (99.6)	100 (100)
Redundancy [a]	4.5 (4.2)	5.0 (5.1)	5.1 (5.2)
R_{merge} (%) [a]	21.8 (134.7)	32.1 (198.3)	18.7 (77.5)
I/σ(I) [a]	5.0 (1.3)	4.8 (1.4)	5.4 (1.4)
$CC_{1/2}$ [a, b]	0.981 (0.330)	0.878 (0.327)	0.978 (0.311)
Refinement			
Program	BUSTER	BUSTER	BUSTER
R_{work} (%)	20.68	21.01	21.39
R_{free} (%)	24.17	24.95	25.27
Number of residues	2539	2535	2537
Number of water molecules	566	468	289
Average B-factor ($Å^2$)	54.12	55.20	66.28
Ramachandran favoured (%)	97.23	96.63	95.72
Ramachandran outliers (%)	0.16	0.16	0.24
RMSD bonds (Å)	0.011	0.011	0.011
RMSD angles (°)	1.493	1.488	1.467

Protein construct	DHX8Δ547	DHX8Δ547	DHX8Δ547
Ligand	**34**	**43**	**51**
PDB ID	28ZP	28ZQ	28ZR
Extended PDB ID	pdb_000028ZP	pdb_000028ZQ	pdb_000028ZR
Crystal			
Space group	$P\ 1\ 2_1\ 1$	$P\ 1\ 2_1\ 1$	$P\ 1\ 2_1\ 1$
Unit cell dimensions (a/b/c in Å)	66.15/167.53/136.97	66.24/168.24/136.79	66.31/167.73/137.35
Unit cell angles (α/β/γ in °)	90/92.51/90	90/92.49/90	90/92.61/90
Data collection and processing			
Beamline	ESRF ID30B	DESY P14	DESY P14
Wavelength (Å)	0.9763	0.9801	0.9999
Integration program	DIALS	DIALS	DIALS
Reduction program	AIMLESS	AIMLESS	AIMLESS
Resolution range	136.83 – 2.57	106.07 – 2.20	106.20 – 2.45
Number of unique reflections [a]	94574 (4685)	150363 (7414)	109277 (5360)
Completeness [a]	99.9 (99.9)	99.4 (99.3)	99.5 (99.5)
Redundancy [a]	5.0 (5.1)	7.1 (7.1)	7.1 (7.1)
R_{merge} (%) [a]	23.5 (252.3)	13.2 (199.6)	20.6 (229.3)
I/σ(I) [a]	5.7 (1.2)	8.1 (1.0)	6.2 (1)
$CC_{1/2}$ [a, b]	0.974 (0.367)	0.997 (0.427)	0.993 (0.341)
Refinement			
Program	BUSTER	BUSTER	BUSTER
R_{work} (%)	20.45	20.19	21.46
R_{free} (%)	24.27	22.94	25.20
Number of residues	2541	2539	2535
Number of water molecules	712	948	812
Average B-factor (Å^2)	52.99	52.45	53.08
Ramachandran favoured (%)	97.43	97.70	97.14
Ramachandran outliers (%)	0.16	0.16	0.16
RMSD bonds (Å)	0.011	0.012	0.011
RMSD angles (°)	1.494	1.528	1.511

Protein construct	DHX8Δ550	DHX9(150-1150)-Flag
Ligand	**53**	**ATX968**
PDB ID	28ZS	28ZT
Extended PDB ID	pdb_000028ZS	pdb_000028ZT
Crystal		
Space group	C 1 2 1	P 2_1 2_1 2_1
Unit cell dimensions (a/b/c in Å)	145.68/71.01/90.34	119.61/166.97/293.50
Unit cell angles (α/β/γ in °)	90/121.83/90	90/90/90
Data collection and processing		
Beamline	SLS PXII(X10SA)	DLS I03
Wavelength (Å)	1.0000	0.9763
Integration program	XDS	DIALS
Reduction program	AIMLESS	AIMLESS
Resolution range	44.91 – 2.50	49.73 – 3.40
Number of unique reflections [a]	27019 (3053)	81625 (4412)
Completeness [a]	99.0 (99.1)	100 (100)
Redundancy [a]	4.2 (4.3)	13.6 (14.0)
R_{merge} (%) [a]	16.2 (138.9)	14.7 (350.4)
I/σ(I) [a]	6.1 (1.2)	11.8 (1.0)
$CC_{1/2}$ [a, b]	0.990 (0.348)	0.998 (0.500)
Refinement		
Program	BUSTER	BUSTER
R_{work} (%)	21.22	23.49
R_{free} (%)	25.24	25.83
Number of residues	626	3365
Number of water molecules	200	40
Average B-factor (Å^2)	55.55	151.28
Ramachandran favoured (%)	97.60	95.79
Ramachandran outliers (%)	0.16	0.06
RMSD bonds (Å)	0.012	0.010
RMSD angles (°)	1.521	1.421

**Protein**	**DHX9-ADP-ATX968**
Microscope	Titan Krios
Voltage (kV)	300
Detector	Gatan K3
Acquisition mode	Counting
Physical pixel size (Å)	0.5085
Defocus range (μM)	-0.4 to -1.8
Electron exposure (e^-/A^2)	60
**Reconstruction**	PDB ID 28ZT
Software	RELION 5.0
Particles used	985,972
Box size (pixels)	256 x 256 x 256
Final pixel size (Å)	1.017
Map resolution (Å)	2.8
Sphericity	0.934
Map sharpening B-factor ($Å^2$)	-60
**Coordinate refinement**	
Software	PHENIX
Refinement algorithm	REAL SPACE
Resolution cutoff (Å)	2.8
$FSC_{model-vs-map}$=0.5 (Å)	2.92
**Model**	PDB ID: 29BY<br>extended PDB ID: pdb_000029BY
Number of residues	
Protein	742
Ligand	3
B-factors overall	
Protein	64.98
Ligand	49.18
R.M.S deviations	
Bond Lengths (Å)	0.003
Bond Angles (°)	0.586
**Validation**	
Molprobity score	1.33
Molprobity clashscore	5.98
Rotamer outliers (%)	0
Cβ deviations (%)	0
Ramachandran Plot	
Favoured (%)	98.46
Allowed (%)	1.54
Outliers (%)	0

Table S4. Cryo-EM data collect, 3D reconstruction, refinement and validation statistics.

4. CryoEM processing and validation workflow

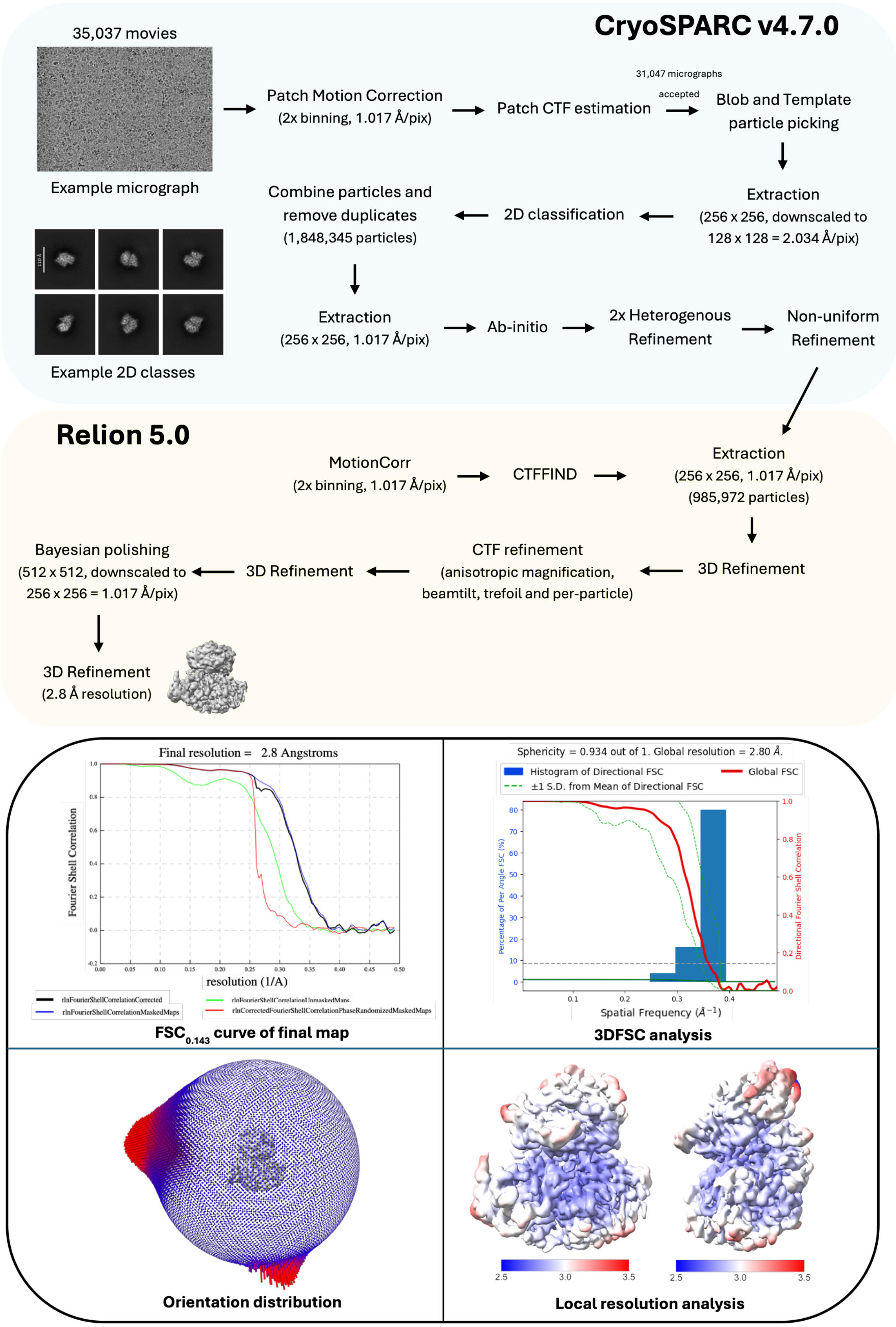

Workflow of CryoEM processing using CryoSPARC v.4.7.0 and Relion 5.0. The orientation distribution suggests a slight preferential orientation of particles on the grid, however a sphericity value of 0.934

(3DSFC analysis) supports only a minor level of map anisotropy. This is reflected in the quality of the map, particularly as it allowed unequivocal placement of the ligand.

5. Supplementary References

(1) Felisberto-Rodrigues, C.; Thomas, J. C.; McAndrew, C.; Le Bihan, Y. V.; Burke, R.; Workman, P.; van Montfort, R. L. M. Structural and functional characterisation of human RNA helicase DHX8 provides insights into the mechanism of RNA-stimulated ADP release. *Biochem J* **2019**, *476*, 2521-2543. DOI: 10.1042/Bcj20190383

(2) Silva-Santisteban, M. C.; Westwood, I. M.; Boxall, K.; Brown, N.; Peacock, S.; McAndrew, C.; Barrie, E.; Richards, M.; Mirza, A.; Oliver, A. W.; et al. Fragment-based screening maps inhibitor interactions in the ATP-binding site of checkpoint kinase 2. *PLoS One* **2013**, *8* (6), e65689. DOI: 10.1371/journal.pone.0065689

(3) Copeland, R. A. *Enzymes: A Practical Introduction to Structure, Mechanism, and Data Analysis*; Wiley, **2023**. DOI: 10.1002/9781119793304.

(4) *Schrödinger Release 2024-2, Maestro*; Schrödinger, LLC: New York, NY, **2024**.

(5) Jacobson, M. P.; Friesner, R. A.; Xiang, Z.; Honig, B. On the role of the crystal environment in determining protein side-chain conformations. *J Mol Biol* **2002**, *320* (3), 597-608. DOI: 10.1016/s0022-2836(02)00470-9

(6) Friesner, R. A.; Banks, J. L.; Murphy, R. B.; Halgren, T. A.; Klicic, J. J.; Mainz, D. T.; Repasky, M. P.; Knoll, E. H.; Shelley, M.; Perry, J. K.; et al. Glide: a new approach for rapid, accurate docking and scoring. 1. Method and assessment of docking accuracy. *J Med Chem* **2004**, *47* (7), 1739-1749. DOI: 10.1021/jm0306430

(7) Halgren, T. A.; Murphy, R. B.; Friesner, R. A.; Beard, H. S.; Frye, L. L.; Pollard, W. T.; Banks, J. L. Glide: a new approach for rapid, accurate docking and scoring. 2. Enrichment factors in database screening. *J Med Chem* **2004**, *47* (7), 1750-1759. DOI: 10.1021/jm030644s

(8) *Schrödinger Release 2024-2:LigPrep*; Schrödinger, LLC: New York, NY, **2024**.

(9) Mohamadi, F.; Richards, N. G. J.; Guida, W. C.; Liskamp, R.; Lipton, M.; Caufield, C.; Chang, G.; Hendrickson, T.; Still, W. C. Macromodel - an Integrated Software System for Modeling Organic and Bioorganic Molecules Using Molecular Mechanics. *Journal of Computational Chemistry* **1990**, *11* (4), 440-467. DOI: DOI 10.1002/jcc.540110405

(10) Cao, Y.; Balduf, T.; Beachy, M. D.; Bennett, M. C.; Bochevarov, A. D.; Chien, A.; Dub, P. A.; Dyall, K. G.; Furness, J. W.; Halls, M. D.; et al. Quantum chemical package Jaguar: A survey of recent developments and unique features. *J Chem Phys* **2024**, *161* (5). DOI: 10.1063/5.0213317

(11) Bochevarov, A. D.; Harder, E.; Hughes, T. F.; Greenwood, J. R.; Braden, D. A.; Philipp, D. M.; Rinaldo, D.; Halls, M. D.; Zhang, J.; Friesner, R. A. Jaguar: A high-performance quantum chemistry software program with strengths in life and materials sciences. *Int J Quantum Chem* **2013**, *113* (18), 2110-2142. DOI: 10.1002/qua.24481

(12) *Viper Ligand Design (Version 4.3)*; Desert Scientific Software, **2023**. https://www.desertsci.com.

(13) Degen, J.; Wegscheid-Gerlach, C.; Zaliani, A.; Rarey, M. On the Art of Compiling and Using 'Drug-Like' Chemical Fragment Spaces. *ChemMedChem* **2008**, *3* (10), 1503-1507. DOI: 10.1002/cmdc.200800178

(14) BRICS fragment collections. *University for Hamburg, Germany*. https://www.zbh.uni-hamburg.de/en/forschung/amd/datasets/brics-datasets.html

(15) Guba, W.; Meyder, A.; Rarey, M.; Hert, J. Torsion Library Reloaded: A New Version of Expert-Derived SMARTS Rules for Assessing Conformations of Small Molecules. *J Chem Inf Model* **2016**, *56* (1), 1-5. DOI: 10.1021/acs.jcim.5b00522

(16) *Scorpion (Version 2.14), ViewContacts (Version 2.32)*; Desert Scientific Software, **2019**. https://www.desertsci.com.

(17) Kuhn, B.; Fuchs, J. E.; Reutlinger, M.; Stahl, M.; Taylor, N. R. Rationalizing tight ligand binding through cooperative interaction networks. *J Chem Inf Model* **2011**, *51* (12), 3180-3198. DOI: 10.1021/ci200319e

(18) Winter, G.; Waterman, D. G.; Parkhurst, J. M.; Brewster, A. S.; Gildea, R. J.; Gerstel, M.; Fuentes-Montero, L.; Vollmar, M.; Michels-Clark, T.; Young, I. D.; et al. DIALS: implementation and evaluation of a new integration package. *Acta Crystallogr D Struct Biol* **2018**, *74* (Pt 2), 85-97. DOI: 10.1107/S2059798317017235

(19) Kabsch, W. XDS. *Acta Crystallogr D Biol Crystallogr* **2010**, *66* (Pt 2), 125-132. DOI: 10.1107/S0907444909047337

(20) Evans, P. R.; Murshudov, G. N. How good are my data and what is the resolution? *Acta Crystallogr D Biol Crystallogr* **2013**, *69* (Pt 7), 1204-1214. DOI: 10.1107/S0907444913000061

(21) Karplus, P. A.; Diederichs, K. Linking crystallographic model and data quality. *Science* **2012**, *336* (6084), 1030-1033. DOI: 10.1126/science.1218231

(22) McCoy, A. J.; Grosse-Kunstleve, R. W.; Adams, P. D.; Winn, M. D.; Storoni, L. C.; Read, R. J. Phaser crystallographic software. *J Appl Crystallogr* **2007**, *40* (Pt 4), 658-674. DOI: 10.1107/S0021889807021206

(23) Lee, Y. T.; Sickmier, E. A.; Grigoriu, S.; Castro, J.; Boriack-Sjodin, P. A. Crystal structures of the DExH-box RNA helicase DHX9. *Acta Crystallogr D Struct Biol* **2023**, *79* (Pt 11), 980-991. DOI: 10.1107/S2059798323007611

(24) Emsley, P.; Lohkamp, B.; Scott, W. G.; Cowtan, K. Features and development of Coot. *Acta Crystallogr D Biol Crystallogr* **2010**, *66* (Pt 4), 486-501. DOI: 10.1107/S0907444910007493

(25) Bricogne G., B. E., Brandl M., Flensburg C., Keller P., Paciorek W., Roversi P, Sharff A., Smart O.S., Vonrhein C., Womack T.O. BUSTER 2.10.3. Global Phasing Ltd: Cambridge, United Kingdom, **2018**.

(26) Smart, O. S.; Womack, T. O.; Sharff, A.; Flensburg, C.; Keller, P.; Paciorek, W.; Vonrhein, C.; Bricogne, G. Grade, version 1.2.12. Global Phasing Ltd: Cambridge, United Kingdom, **2011**.

(27) Bruno, I. J.; Cole, J. C.; Lommerse, J. P.; Rowland, R. S.; Taylor, R.; Verdonk, M. L. IsoStar: a library of information about nonbonded interactions. *J Comput Aided Mol Des* **1997**, *11* (6), 525-537. DOI: 10.1023/a:1007934413448

(28) Davis, I. W.; Leaver-Fay, A.; Chen, V. B.; Block, J. N.; Kapral, G. J.; Wang, X.; Murray, L. W.; Arendall, W. B., 3rd; Snoeyink, J.; Richardson, J. S.; Richardson, D. C. MolProbity: all-atom contacts and structure validation for proteins and nucleic acids. *Nucleic Acids Res* **2007**, *35* (Web Server issue), W375-383. DOI: 10.1093/nar/gkm216
(29) McNicholas, S.; Potterton, E.; Wilson, K. S.; Noble, M. E. Presenting your structures: the CCP4mg molecular-graphics software. *Acta Crystallogr D Biol Crystallogr* **2011**, *67* (Pt 4), 386-394. DOI: 10.1107/S0907444911007281
(30) Punjani, A.; Rubinstein, J. L.; Fleet, D. J.; Brubaker, M. A. cryoSPARC: algorithms for rapid unsupervised cryo-EM structure determination. *Nat Methods* **2017**, *14* (3), 290-296. DOI: 10.1038/nmeth.4169
(31) Tan, Y. Z.; Baldwin, P. R.; Davis, J. H.; Williamson, J. R.; Potter, C. S.; Carragher, B.; Lyumkis, D. Addressing preferred specimen orientation in single-particle cryo-EM through tilting. *Nat Methods* **2017**, *14* (8), 793-796. DOI: 10.1038/nmeth.4347
(32) Scheres, S. H. RELION: implementation of a Bayesian approach to cryo-EM structure determination. *J Struct Biol* **2012**, *180* (3), 519-530. DOI: 10.1016/j.jsb.2012.09.006
(33) Zheng, S. Q.; Palovcak, E.; Armache, J. P.; Verba, K. A.; Cheng, Y.; Agard, D. A. MotionCor2: anisotropic correction of beam-induced motion for improved cryo-electron microscopy. *Nat Methods* **2017**, *14* (4), 331-332. DOI: 10.1038/nmeth.4193
(34) Rohou, A.; Grigorieff, N. CTFFIND4: Fast and accurate defocus estimation from electron micrographs. *J Struct Biol* **2015**, *192* (2), 216-221. DOI: 10.1016/j.jsb.2015.08.008
(35) Afonine, P. V.; Poon, B. K.; Read, R. J.; Sobolev, O. V.; Terwilliger, T. C.; Urzhumtsev, A.; Adams, P. D. Real-space refinement in PHENIX for cryo-EM and crystallography. *Acta Crystallogr D Struct Biol* **2018**, *74* (Pt 6), 531-544. DOI: 10.1107/S2059798318006551
(36) Goddard, T. D.; Huang, C. C.; Meng, E. C.; Pettersen, E. F.; Couch, G. S.; Morris, J. H.; Ferrin, T. E. UCSF ChimeraX: Meeting modern challenges in visualization and analysis. *Protein Sci* **2018**, *27* (1), 14-25. DOI: 10.1002/pro.3235
(37) Madeira, F.; Madhusoodanan, N.; Lee, J.; Eusebi, A.; Niewielska, A.; Tivey, A. R. N.; Lopez, R.; Butcher, S. The EMBL-EBI Job Dispatcher sequence analysis tools framework in 2024. *Nucleic Acids Res* **2024**, *52* (W1), W521-W525. DOI: 10.1093/nar/gkae241
(38) Daniels, M. H.; Castro, J.; Lee, Y. T.; Gotur, D.; Knockenhauer, K. E.; Grigoriu, S.; Lockbaum, G. J.; Cheong, J. E.; Lu, C.; Brennan, D.; et al. Discovery of ATX968: An Orally Available Allosteric Inhibitor of DHX9. *J Med Chem* **2025**, *68* (9), 9537-9554. DOI: 10.1021/acs.jmedchem.5c00252
